# Myelin Sheath-Inspired Fluorocarbon Hydrophobic Bridges for Stable n‑Type Organic Mixed Ionic–Electronic Conductors

**DOI:** 10.1021/jacs.6c12196

**Published:** 2026-10-09

**Authors:** Wang Feng, Shaodan Cai, Chao Deng, Bei Zhang, Mengge Wu, Jianhua Chen, Antonio Facchetti

**Affiliations:** † Key Laboratory of Medicinal Chemistry for Natural Resource, Ministry of Education, School of Chemical Science and Technology, 12635 Yunnan University, Kunming 650091, China; ∥ Department of Materials, The Hong Kong University of Science and Technology, Hong Kong 999077, China; ‡ School of Materials Science and Engineering, 115724 Georgia Institute of Technology, Atlanta, Georgia 30332, United States; § Southwest United Graduate School, Kunming 650092, China

## Abstract

Operationally stable n-type organic mixed ionic–electronic conductors (OMIECs) remain a critical bottleneck for organic electrochemical transistors (OECTs) for application in bioelectronic and neuromorphic devices. However, several state-of-the-art n-type OMIECs suffer from performance decay and poor stability when operated in aqueous media. Inspired by myelin sheaths, we challenge the conventional “π-backbone + hydrophilic side-chain” design and propose a molecular architecture with fluorocarbon hydrophobic bridges to spatially segregate, at the molecular level, ionic and electronic pathways. By tuning the fluorocarbon bridge chain length in fluorocarbon-ethylene glycol chain substituents [−CH_2_(CF_2_)_*n*_CH_2_(OCH_2_CH_2_)_5_OMe = 2*n*FG; *n* = 1, 2, 3, 4, 6] of a DPP-CNTVT polymer series (2*n*FG-P), we balance electron transport, ion exchange, redox chemistry efficiency, and water incorporation. Extensive in situ spectroelectrochemistry, EIS, Raman, EQCM-D, AFM, and GIWAXS characterization reveals that tuning the length of the hydrophobic bridge enables good electron transport, suppresses excessive water incorporation, and enhances stability. Thus, the polymer with a mid-length bridge, 8FG-P (*n* = 4), achieves the greatest electron transport (μC* ∼ 157 F cm^–1^ V^–1^ s^–1^, μ ∼ 1.22 cm^2^ V^–1^ s^–1^), fastest response (τ_on_/τ_off_ = 2.6/1.5 ms), and retains >90% of the initial performance after 10,000 s of operation in water, a result independent of the polymer molecular weight. This strategy potentially expands OMIECs design principles and offers useful insights toward stable n-type OECT semiconductors.

## Graphical abstract

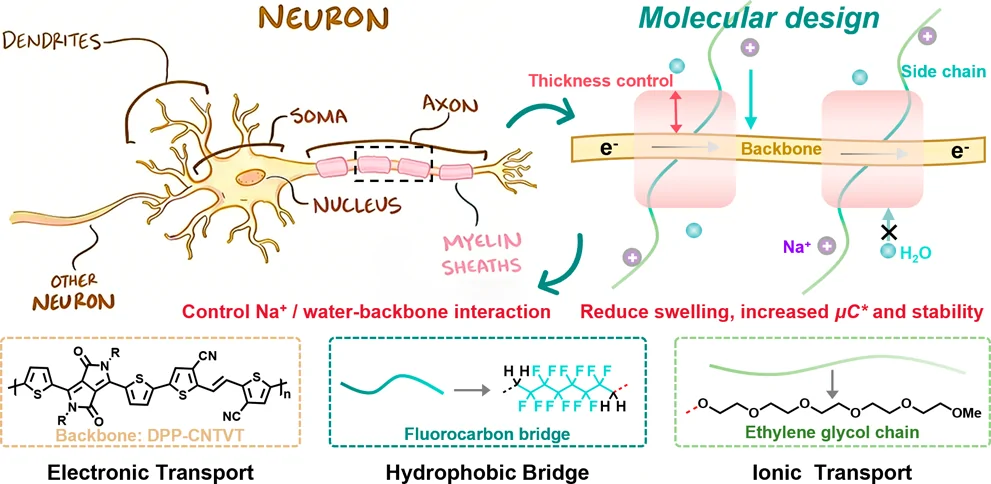

## 1 Introduction

Organic mixed ionic–electronic conductors (OMIECs) are the key semiconductor components of organic electrochemical transistors (OECTs), which are devices explored for novel bioelectronics and neuromorphic applications. In the bioelectronics area, real-time health monitoring enabled by flexible, wearable, and implantable devices has driven intense demand for high-performance OECTs as core signal-converting components.^1–3^ OMIECs stand out as ideal OECT channel materials due to their unique ability to transport both ions and electrons, efficiently transducing biological ionic signals into readable electronic outputs while offering excellent biocompatibility and mechanical conformability.^4–9^ These advantages position OECTs as leading candidates for next-generation bioelectronic devices.^10–15^ Crucially, n-type OMIECs are indispensable for advancing OECT systems for constructing complementary logic circuits^16,17^ and for inherent selectivity toward positively charged ions and biomolecules—the most prevalent biological analytes in physiological environments.^14,18,19^ However, real-world applications, especially for implanted devices targeting long-term operation in physiological environments, remain severely hindered by the poor operational stability of n-type OMIECs, representing a critical bottleneck for laboratory-to-clinic translation.^20–24^

With few notable exceptions,^25,26^ high-performance OMIECs typically follow the conventional molecular design of a π-conjugated backbone covalently tethered with hydrophilic oligoethylene glycol (OEG) side chains.^27–30^ While this molecular architecture facilitates ion exchange and efficient redox chemistry, the need for both ionic and electronic pathways creates an inherent vulnerability when operating these devices in water and physiological media: excessive water molecules penetrate into the polymer bulk, triggering severe degradation mechanisms. These include electron trapping,^31–33^ parasitic side reactions,^34,35^ disruption of crystalline domain connectivity via excessive swelling,^36,37^ and delamination from the electrode.^38,39^ These effects are particularly detrimental to n-type OECTs, where doped charges are inherently more sensitive to moisture and oxygen. Despite progress in improving stability via molecular weight control,^40,41^ energy-level tuning,^31,42^ crystallinity optimization,^43,44^ electrolyte engineering,^45–47^ or device encapsulation,^48,49^ such strategies rarely address the root cause of water-induced degradation. Traditional OMIEC designs cannot prevent water from deteriorating the charge-carrying backbone, leaving long-term stability unmet for practical implantable applications.

For functional systems requiring both reliable signal transmission and long-term stability, hydrophobic isolation represents an effective structural solution to minimize signal leakage while protecting active components from external environmental perturbations. Myelin sheaths are specialized, lipid-rich membranes that illustrate this structural principle, enveloping axons throughout the nervous system (Figure 1a).^50–52^ They fulfill two fundamental physiological roles: (1) Acting as highly efficient electrical insulators, they enable rapid and precise propagation of nerve impulses by minimizing signal attenuation and leakage; (2) Forming a dense, hydrophobic barrier, they shield axons from extrinsic environmental perturbations, thereby conferring long-term structural stability and functional reliability to the nervous system (Figure 1b).^53^ Nevertheless, ions can access the cell through ion channels at the Ranvier’s nodes, the gaps between myelin sheaths, to generate the action potential (Figure 1c).

**Figure 1:**
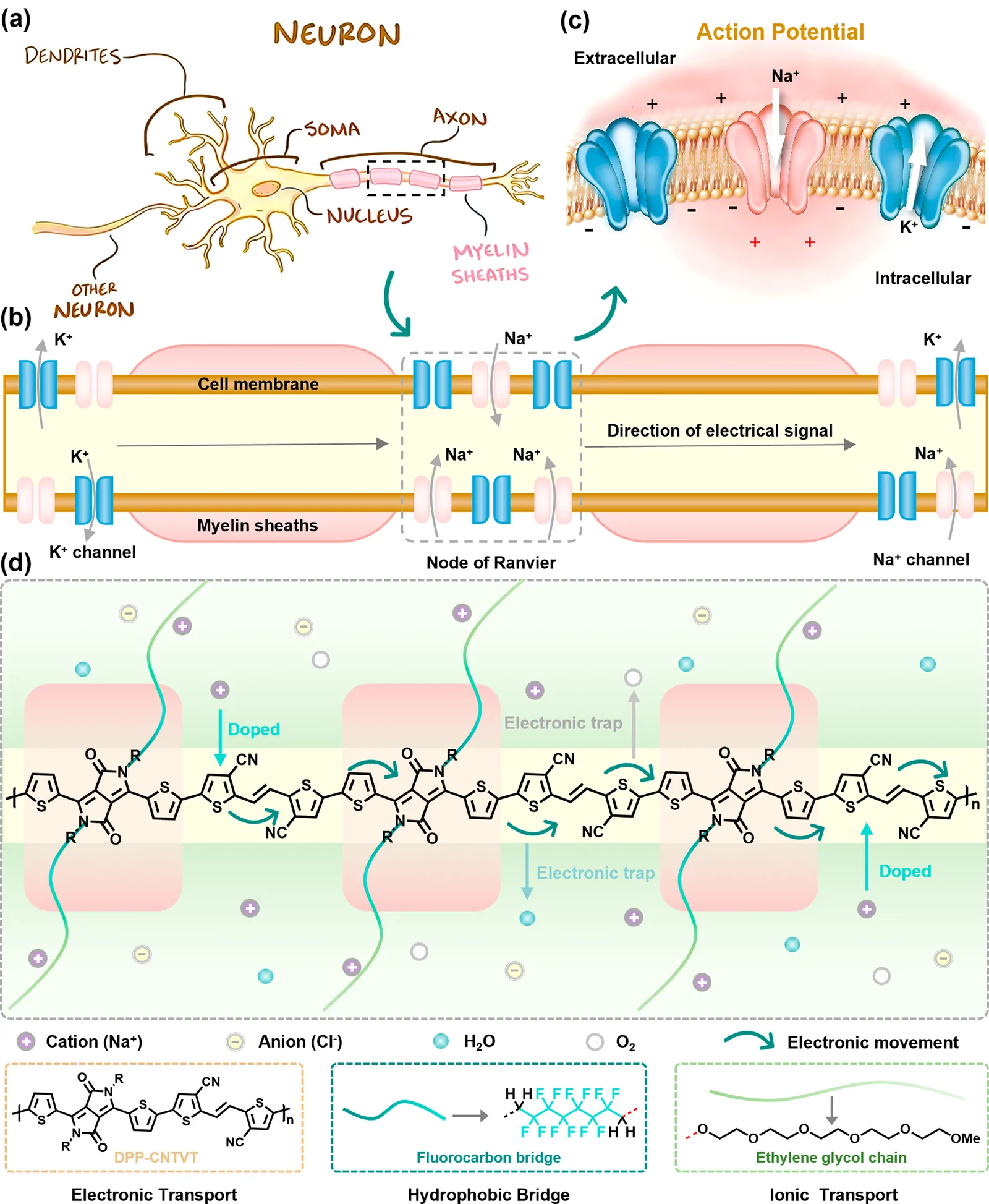
Schematic illustration of a neuron and the novel design strategy of fluorinated hydrophobic bridge barriers: (a) Schematic of myelin sheaths wrapping around a neuron’s axon. (b) The process of generating an action potential (nerve impulses). (c) Core functions of myelin sheath: electrical insulation and physical protection. (d) Molecular design and chemical structure of our fluorinated OMIECs.

Motivated by this protective mechanism and previous studies addressing stability in n-type semiconductors for OFETs,^32,54–56^ in this work we strategically insert a fluorocarbon hydrophobic bridge with different lengths [–(CF_2_)*_n_*– = 2*n*F, *n* = 1, 2, 3, 4, 6] between the ethylene glycol (G) hydrophilic ionic side chains and the electron-transporting DPP-CNTVT conjugated backbone, creating a spatially segregated “ionic channel–hydrophobic barrier–electronic channel” architecture (Figure 1d). This design aims at physically blocking excessive water penetration toward the π-backbone without disabling ion transport, which may offer a route toward mitigating water-induced instability at the molecular level. Thus, a series of five diketopyrrolopyrrole (DPP)-*co*-dicyanothenylenevinylene (CNTVT) polymers *N*-substituted with the fluorocarbon-oligoethylene glycol chain (2*n*FG), indicated as 2*n*FG-P (Figure 2a), is synthesized and characterized using a combination of electrochemical impedance spectroscopy (EIS), in situ spectroelectrochemistry (spectroe-chem), in situ Raman, electrochemical quartz crystal microbalance with dissipation monitoring (EQCM-D), atomic force microscopy (AFM), and grazing-incidence wide-angle X-ray scattering (GIWAXS). Our results indicate that a moderate-length fluorocarbon hydrophobic bridge (8FG) optimizes ion transport, redox efficiency, and π-packing while strongly suppressing water uptake and swelling. Molecular dynamics (MD) simulations further suggest that these bridges can modulate the distance among water, ions, and the conjugated backbone, weakening interactions between water and the backbone. When implemented in OECTs, 8FG-P and 12FG-P exhibit exceptional operational stability, retaining 93% and 97% of their performance after 10,000 s of continuous operation. In contrast, excessively short (4FG) or long (12FG) bridges compromise charge mobility and device performance. These results can offer molecular design insights towards stable, high-performance n-type OECTs and suggest a potential pathway for future implantable bioelectronic applications.

**Figure 2:**
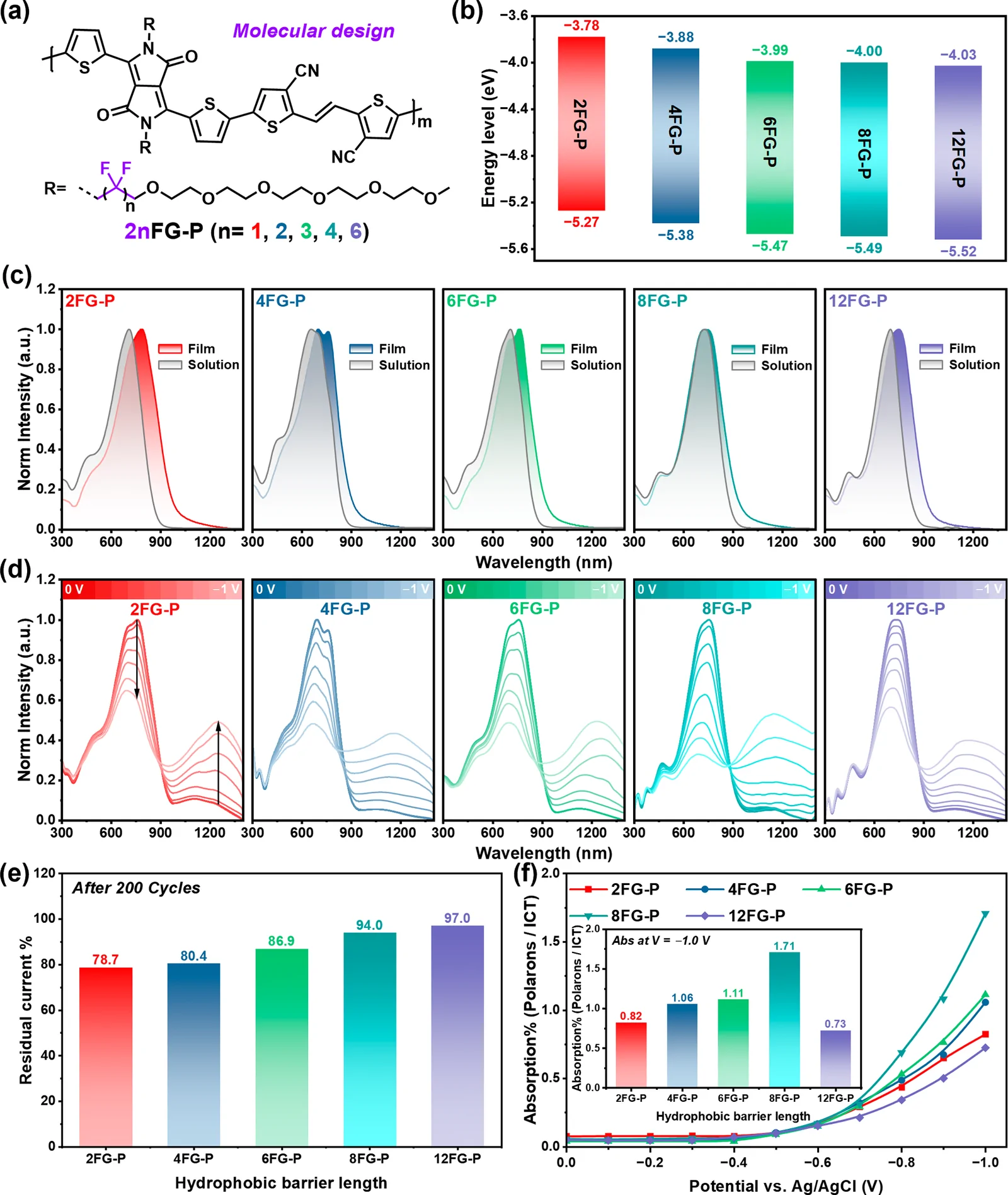
(a) Chemical structures of the synthesized polymers. (b) Energy level diagram. (c) Normalized optical absorption spectra in HFIP solution (gray line) and as thin films (colored line). (d) Spectroelectrochemistry (SEC): Potential-dependent UV/vis-NIR absorption spectra. (e) Residual current for the polymers at a potential of −1.0 V after 200 CV cycles in 0.1 M NaCl solution. (f) The ratio of the absorption intensity of polymer polarons and intramolecular charge transfer (ICT) at different biases.

## 2 Results and Discussion

### 2.1 Polymer Synthesis

Figure 2a reports the chemical structure of the new alternating poly­[2,5-bis­(1-2*n*-fluorocarbon-oligoethylene glycol-yl)-3,6-di­(thiophen-2-yl)­pyrrolo-[3,4-*c*]­pyrrole-1,4­(2*H*,5*H*)-dione-*alt*-(*E*)-1,2-bis­(3-cyanothiophen-2-yl)­ethene] (2*n*FG-DPP-CNTVT, abbreviated as 2*n*FG-P hereafter). This electron-deficient backbone was chosen to ensure high electron mobility and π-backbone planarity, which favor n-type charge transport.^57,58^ The synthetic route to 2*n*FG-P is illustrated in Scheme S1. Briefly, pentaethylene glycol (A1) was first monotosylated to yield intermediate A3, which was then coupled with fluorinated dialcohols (A4) via a base-promoted etherification using sodium hydride under anhydrous conditions,^59,60^ affording amphiphilic OEG-fluorinated alcohol intermediates (A5). Subsequent activation of A5 as trifluoromethanesulfonate (OTf) derivatives (A7) provided highly reactive electrophiles, which proved critical for efficient *N*,*N*′-dialkylation of the DPP core (A8) to access side-chain-functionalized DPPs (A9). Control experiments revealed that conventional brominated fluoroalkyl halide precursors showed negligible reactivity with the DPP unit, underscoring the essential role of the OTf leaving group in achieving successful functionalization. Finally, the thiophene α-positions of A9 were selectively brominated using *N*-bromosuccinimide (NBS), delivering five dibrominated monomers (A10, 2*n*FG-DPP-Br, *n* = 1, 2, 3, 4, 6) for subsequent polymerization. Each monomer was copolymerized with 5,5′-bis­(tributylstannyl)-[2,2′-bithiophene]-3,3′-dicarbonitrile via palladium-catalyzed Stille cross-coupling polycondensation to afford the target 2*n*FG-P polymers. All polymers were purified by sequential Soxhlet extraction with methanol, acetone, *n*-hexane, and hexafluoroisopropanol (HFIP). The HFIP-soluble fractions were collected and analyzed by gel permeation chromatography (GPC) to determine molecular weights and dispersity. The number-average molecular weights (*M*_n_) and dispersity (*Đ*) are 24.6 kDa/1.5, 27.4 kDa/1.7, 36.8 kDa/1.6, 69.8 kDa/1.9, and 65.2 kDa/2.2, for 2FG-P, 4FG-P, 6FG-P, 8FG-P, and 12FG-P, respectively (Table 1).

**Table 1:** Molecular Mass, Electrochemical Properties, and Optical Parameters of the Polymers

Polymer	*M*_n_ [kDa]^a^	*Đ*	*E*_HOMO_^CV^ [eV]^b^	*E*_LUMO_^CV^ [eV]^b^	*E*_g_^CV^ [eV]^b^	λ_max_^sol^ [nm]^c^	λ_max_^film^ [nm]^c^	Δλ_max_ [nm]^d^	λ_onset_^film^ [nm]^e^
2FG-P	24.6	1.5	–5.27	–3.78	1.49	708	785	77	951
4FG-P	27.4	1.7	–5.38	–3.88	1.50	700	759	59	918
6FG-P	36.8	1.6	–5.47	–3.99	1.48	707	763	56	916
8FG-P	69.8	1.9	–5.49	–4.00	1.49	727	736	9	915
12FG-P	65.2	2.2	–5.52	–4.03	1.49	698	748	50	912

a Determined by GPC using HFIP as an eluent.

b Estimated from the oxidation and reduction onsets of the CV plots.

c Recorded in HFIP solutions and the corresponding films on glass substrates.

d Δλ_max_ =λ_max_^film^ – λ_max_^sol^.

e Determined from the onset of the optical absorption spectra in the films.

### 2.2 Geometry and Electronic Structure

Density functional theory (DFT) calculations at the B3LYP/6–31G­(d,p) level were performed to investigate the geometry and electronic structures of the fluorinated OMIECs, including optimized backbone conformations, highest occupied molecular orbital (HOMO) and lowest unoccupied molecular orbital (LUMO) energy levels, and orbital topologies (Figures S1, S2).^61–63^ All polymers exhibit a highly planar conjugated backbone, which favors extended orbital delocalization and efficient long-range electron transport along the polymer backbone. Electrochemical redox properties were evaluated by cyclic voltammetry (CV) using ferrocene/ferrocenium (Fc/Fc^+^) as the internal standard (Figure S3, Table S1). All OMIECs display well-defined, reversible oxidation and reduction peaks, indicating robust electrochemical redox activity. The HOMO/LUMO energy levels of 2FG-P, 4FG-P, 6FG-P, 8FG-P, and 12FG-P are −5.27/–3.78 eV, −5.38/–3.88 eV, −5.47/–3.99 eV, −5.49/–4.00 eV, and −5.52/–4.03 eV, respectively, with nearly identical band gaps (Figure 2b, Table 1). With increasing fluorocarbon hydrophobic bridge length, both HOMO and LUMO levels gradually decrease, mainly due to the strong and additive electron-withdrawing effect of fluorine atoms.^32^ Importantly, 8FG-P and 12FG-P exhibit a deep LUMO level below −4.0 eV, which not only reduces the energy barrier for efficient electron injection and electrochemical doping but also may enhance the operational stability of n-type OECTs in aqueous environments by suppressing parasitic redox reactions and water-induced degradation.^31,64^ This is corroborated by evaluating the electrochemical stability by 200 consecutive CV cycles at a scan rate of 100 mV s^–1^ (Figures 2e and S4). After continuous cycling, 2FG-P, 4FG-P, and 6FG-P exhibit obvious current decay, retaining ∼79%, ∼80%, and ∼87% of their original current, respectively. In sharp contrast, 8FG-P and 12FG-P exhibit greater stability, retaining ∼94% and ∼97% of their current output. These results indicate that moderately long fluorocarbon hydrophobic bridges effectively suppress structural degradation and side reactions, endowing the n-type OMIECs with remarkable electrochemical robustness in aqueous environments.

### 2.3 Optical and In Situ Spectroelectrochemistry

The ultraviolet-visible-near-infrared (UV–vis–NIR) absorption spectra of 2*n*FG-P in HFIP solution and as thin films are shown in Figure 2c, with key optical parameters summarized in Table 1. All polymers exhibit similar solution-state absorption profiles, with a maximum absorption peak (λ_max_^sol^) at ∼700 nm, with the notable exception of 8FG-P, which is at 727 nm, indicating enhanced backbone planarity and pre-aggregation.^65^ In the solid state, all films display a red-shifted λ_max_ relative to solutions, arising from enhanced intermolecular π–π stacking and interchain aggregation. The solution-to-film red shift (Δλ_max_) follows the order: 2FG-P (77 nm) > 4FG-P (59 nm) > 6FG-P (56 nm) > 12FG-P (50 nm) > 8FG-P (9 nm). The minimal red shift of 9 nm for 8FG-P further suggests pre-aggregation in solution, as indicated by concentration-dependent and variable-temperature UV–vis–NIR absorption spectra (Figure S5), and this pre-aggregation may favor rapid and stable solid-state packing during film deposition.^66^ Furthermore, upon increasing fluorocarbon bridge length, the film absorption onsets (λ_onset_) exhibit a gradual blue shift from 951 to 912 nm. Short fluorocarbon segments (2FG) promote long-range ordered π–π stacking and low-energy optical transitions, whereas longer bridges (4-12FG) moderate extended stacking, rendering the absorption onset dominated by intrinsic backbone conjugation.^67,68^ In situ UV–vis–NIR spectroelectrochemistry was performed to probe ion uptake and electrochemical reduction in 0.1 M aqueous NaCl under cathodic biases from 0 to −1.0 V (Figure 2d). Upon an increased negative potential, the π–π* absorption (350–550 nm) and intramolecular charge-transfer (ICT) band (550–900 nm) gradually decrease, while the intensity of the electron polaron absorption peaks (900–1400 nm) continuously increases.^69,70^ Polymers 2FG-P, 4FG-P, and 6FG-P initiate reduction at −0.5 V, with larger absorbance changes at longer bridge lengths. In contrast, 8FG-P and 12FG-P start reduction at −0.4 V, consistent with the lower reduction potentials observed by CV. To quantitatively evaluate the doping efficiency of the polymers, we calculated the intensity ratio of polaron absorption to intrinsic ICT absorption under varying reductive potentials. A higher polaron/ICT ratio corresponds to a higher population of charged polymer chains.^71^ As shown in Figure 2f, 8FG-P achieves the maximum polaron/ICT ratio of 1.71 at −1.0 V, which is substantially higher than those of its counterparts across the series. This result indicates that the optimized fluorocarbon bridge structure endows 8FG-P with superior electrochemical doping efficiency, which is a prerequisite for high-performance OECTs.

### 2.4 In Situ Raman Spectroscopy

In situ Raman spectroscopy using a 532 nm excitation laser was employed to monitor the evolution of key vibrational modes during electrochemical reduction (Figure S6), providing structural information upon changes in substitution and supporting trends in OECT performance (*vide infra*). Vibrational assignments for the DPP-CNTVT backbone were based on DFT simulation (Figure S7) and previous reports.^72,73^ As shown in Figure 3a,b, peaks i, ii, and iii correspond to C–N, intra-ring C–C, and C=C stretching of the DPP acceptor unit; Peaks iv and v arise from the intra-ring C–C and inter-ring C–C vibrations of the thiophene donor; Peaks vi and vii are assigned to C=C stretching of cyano-substituted thiophene and the π-bridge C=C vibration in the CNTVT, respectively. Lorentzian fitting of the experimental spectra in Figure 3c was used to deconvolve overlapping bands. Notably, the backbone structural perturbations that govern charge transport properties are strongly correlated with these rotationally flexible vibrational modes, which show high sensitivity to the dihedral angle variations between adjacent monomer units.^74,75^ Consequently, the position and line shape of these Raman bands serve as robust spectroscopic indicators for evaluating intramolecular conformational order in the solid state. Thus, the vibrational band centered at 1400–1450 cm^–1^ appears as a broad peak in 2FG-P and progressively narrows with increasing fluorocarbon bridge length, indicating the coexistence of ordered (highlighted in blue) and disordered (highlighted in green) conformational phases along the polymer chains.^76,77^ As the fluorocarbon bridge lengthens, the band gradually narrows, indicating enhanced supramolecular assembly, restricted conformational freedom, and improved backbone planarity. A slight blue shift of the C–N peak (≤5 cm^–1^) indicates minor DPP unit distortion, but this does not compromise overall backbone planarization. Longer substituents also tune the electron-withdrawing character of DPP, further modulating intramolecular charge transfer (ICT) between donor and acceptor segments.

**Figure 3:**
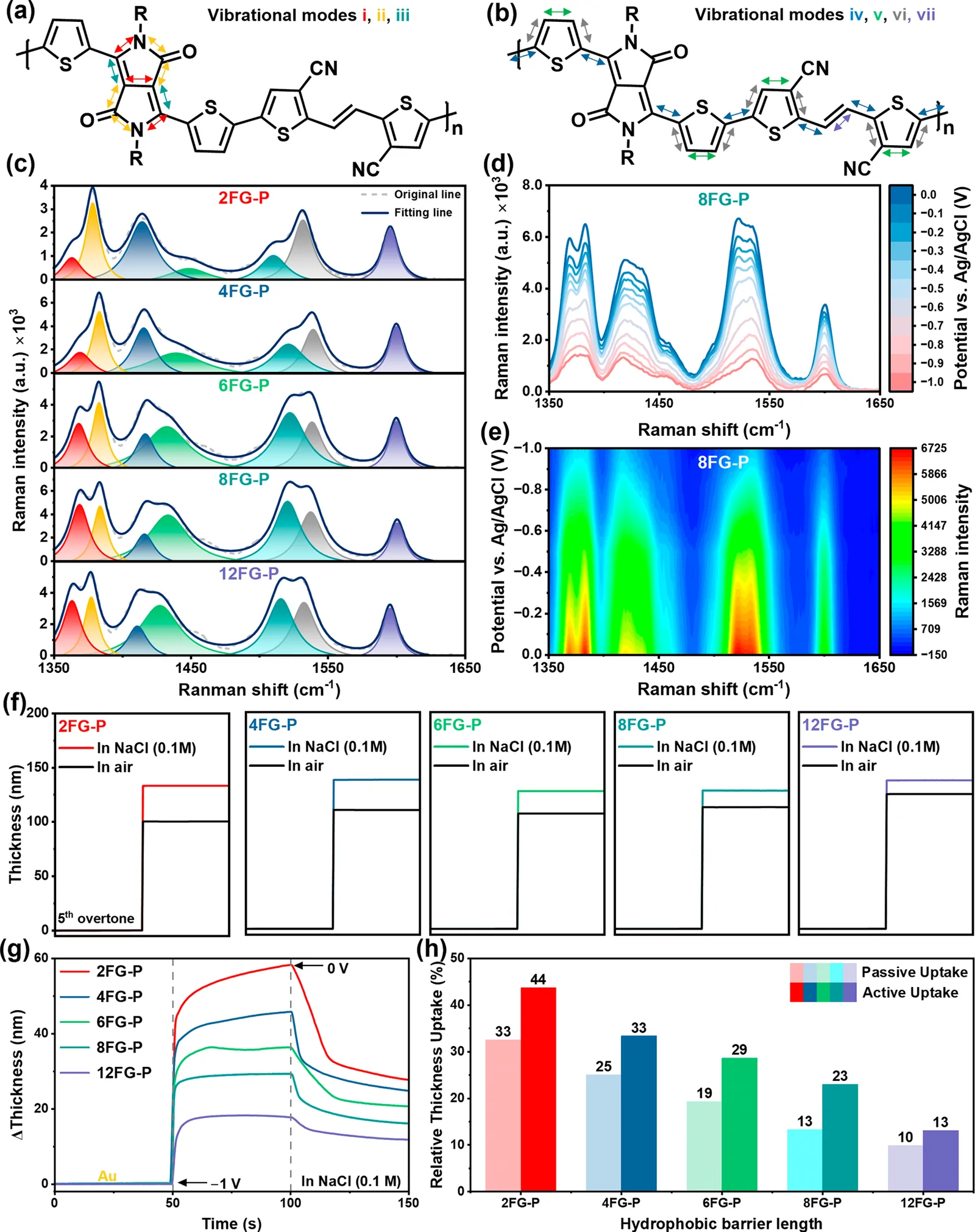
(a) The primary vibrational modes characteristic of the DPP structure. (b) The primary vibrational modes characteristic of the thiophene structure and its derivatives. (c) Raman spectra of the 2*n*FG-P polymer thin films in air, with a single-component band deconvolution based on Lorentzian fitting. (d) The in situ electrochemical Raman measurement of 8FG-P under a constant reduction bias voltage of 0 to −1 V. (e) 2D contour images of in situ Raman spectra of 8FG-P. (f) Calculated thickness shifts for 2*n*FG-P, recorded in air and 0.1 M NaCl (aq.) (g) Relative active thickness uptake for 2*n*FG-P polymer films, recorded in 0.1 M NaCl (aq.) when a 50 s doping pulse at *V* = −1.0 V vs *V*_OC_ is applied (Fitted by a Kelvin–Voigt model). (h) Passive and active swelling in 0.1 M NaCl (aq.) calculated from EQCM-D measurements.

In situ Raman tracking during electrochemical doping is shown in Figures 3d and S8. Upon application of a negative bias, hydrated sodium ions gradually infiltrate the polymer thin film, resulting in the depletion of neutral species and the concurrent generation of polarons, with the Raman spectral intensity decreasing (Figure S9a). Specifically, the relative intensity ratio of the two C–C peaks (*I*_v_/*I*_iv_), reflects changes in the π-electron distribution across the polymer backbone and the thiophene donor units. Upon increasing the negative voltage, 8FG-P shows a more prominent rise in the relative intensity of the thiophene-associated C–C mode v (∼32%) (Figure S9b), alongside a ∼3 cm^–1^ red shift of the corresponding peak (Figure S9c). These results indicate that the accumulation of π-electrons near the donor units becomes more evident during electron polaron formation.^78–80^ To further visualize the dynamic structural evolution of the molecule during doping, a potential-Raman shift heatmap was constructed (Figures 3e and S10). Analytical results reveal that 8FG-P exhibits the fastest decay rate in the intensities of its conjugated backbone characteristic Raman peaks. A pronounced intensity drop is observed at a reduction potential of −0.4 V, significantly exceeding the attenuation magnitudes observed for 2FG-P, 4FG-P, 6FG-P, and 12FG-P under identical electrochemical conditions. At the fully doped state (−1.0 V), 8FG-P displays the lowest residual intensity among all analogues, with complete spectral quenching of all characteristic vibrational modes. This observation suggests substantial structural reorganization within both donor and acceptor units, accompanied by extensive delocalization of negative polarons across the entire conjugated backbone. Such structural evolution directly indicates that the 8FG-P thin film features lower ionic diffusion resistance, more thorough ion penetration, and more uniform doping.

### 2.5 Electrochemical Quartz Crystal Microbalance with Dissipation Monitoring (EQCM-D)

Electrochemical quartz crystal microbalance with dissipation monitoring (EQCM-D) was employed to quantify the passive and active swelling of polymer films, revealing how fluorocarbon length regulates water exchange and structural stability in aqueous electrolytes. This technique allows real-time tracking of thickness and mass changes during electrolyte exposure and electrochemical doping, providing direct insight into solvent–polymer and ion-polymer interactions.^81,82^ Film thicknesses were measured in the dry state and after reaching swelling equilibrium in a 0.1 M NaCl aqueous solution (Figure 3f). Passive swelling arises solely from water penetration without applied bias. Increasing fluorocarbon bridge length strongly suppresses water uptake: 2FG-P shows the largest swelling (∼33%), which systematically decreases to ∼25% (4FG-P), ∼19% (6FG-P), ∼13% (8FG-P), and ∼10% (12FG-P) (Figure 3h, light bars). This trend indicates that longer fluorocarbon bridges progressively suppress hydration of the polymer bulk. Active swelling/deswelling during electrochemical reduction was examined upon doping (−1.0 V vs *V*_OC_) and dedoping (0 V vs *V*_OC_) cycles after equilibration.^38,83^ As shown in Figure 3g, upon electrochemical doping, all films undergo further thickness increase due to Na^+^ and additional water insertion. Active swelling also decreases monotonically with the bridge length, falling from ∼44% to ∼13% (Figure 3h, dark bars), closely mirroring the trend observed for passive swelling. Notably, 8FG-P reaches swelling and doping equilibrium in ∼6 s vs ∼8–∼27 s for the other polymers, indicating rapid yet controlled ion penetration. Complementary contact angle (Figure S11) and dielectric constant measurements of the pristine films (Figure S12) further support these trends: longer fluorocarbon bridges increase hydrophobicity (CA from 69° to 85°) and lower the dielectric constant (ε from 4.5 to 2.9). Collectively, these results demonstrate that the fluorocarbon hydrophobic barrier layer reduces excessive water uptake while preserving sufficient ion permeability for efficient doping.^84,85^

Unlike conventional glycolated OMIECs, which typically suffer from severe swelling and disrupted packing (Table S2), 8FG-P achieves an optimal balance: moderate water uptake enables full electrochemical doping, while the fluorocarbon bridge maintains structural order and minimizes backbone disruption (*vide infra for detailed discussion*). This unique balance should preserve high electron mobility and mechanical robustness during OECT operation, which is critical for stable, high-performance n-type OECTs.

### 2.6 Molecular Dynamics (MD)

Molecular dynamics (MD) simulations were performed to elucidate the atomic-scale roles of fluorocarbon hydrophobic bridges in regulating interactions with Na^+^ ions, water molecules, and conjugated backbones. Simulations were conducted on multi-chain neutral models with varying fluorocarbon bridge lengths in explicit 0.1 M aqueous NaCl solution. During the simulations, full relaxation optimizations were conducted for the spatial distribution of polymer chains, the packing arrangement of side chains, and the positional coordination of dopant ions and water molecules, to characterize the solvation and interaction behavior in the amorphous region of polymer thin films. Full methodological details, including the parameters used for force field validation and calibration, are provided in the Supporting Information (SI). Figures 4a and S13 report a representative snapshot of the full periodic simulation system, containing the polymer chains, Na^+^ counterions, Cl^–^ ions, and explicit water molecules, with a magnified view visualizing the local coordination environment of Na^+^ around the polymer backbone as well as the spatial distribution of solvent and ionic species. Radial distribution function (RDF) analysis for 8FG-P (Figures 4b and S14, S15) reveals well-defined intermolecular coordination. Characteristic peaks for Na^+^–O (C=O, backbone) and Na^+^–O (PEG side chain) support that carbonyl groups and ether oxygen atoms serve as primary Na^+^ binding sites.^86,87^

**Figure 4:**
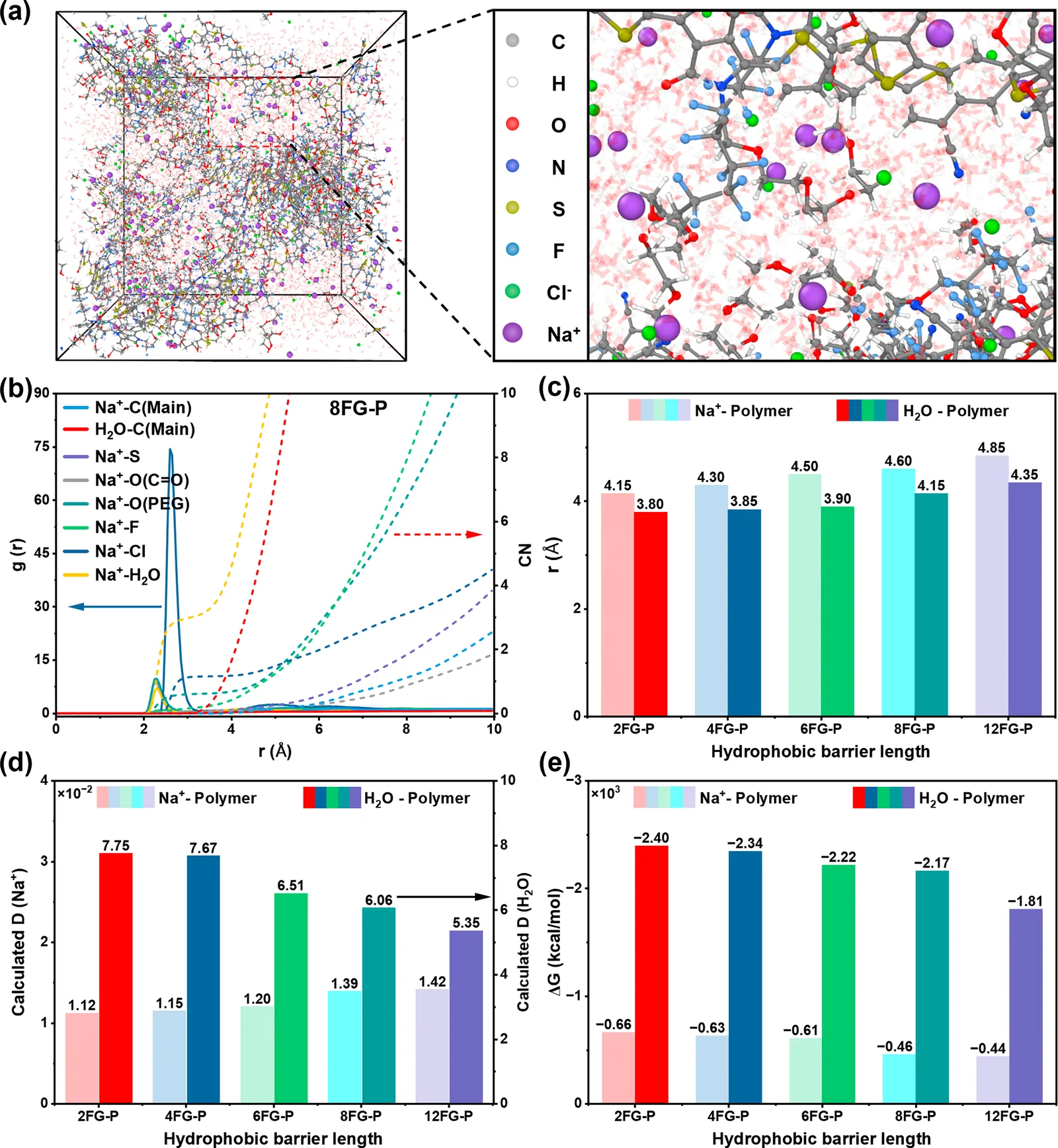
(a) Molecular dynamics (MD) simulation snapshots of 8FG-P in NaCl aqueous solution. (b) RDFs and the corresponding coordination numbers of 8FG-P. (c) Interaction distances of Na^+^ and water molecules with the polymer backbone in different lengths of fluorinated hydrophobic bridge models. (d) Diffusion coefficient (D) of Na^+^ and water molecules in different lengths of fluorinated hydrophobic bridge models. (e) Interaction energy of Na^+^ and water molecules with the polymer.

With increasing the fluorocarbon bridge length from 2FG to 12FG, the average distances from Na^+^ and H_2_O to the polymer backbone increase monotonically (Figure 4c), from 4.15 Å (Na^+^) and 3.80 Å (H_2_O) for 2FG-P to 4.85 Å (Na^+^) and 4.35 Å (H_2_O) for 12FG-P. These results indicate that the fluorocarbon bridge imposes strong hydrophobic repulsions, pushing ions and water away from the electronically active backbone and weakening Coulombic interactions.^88^ Diffusion coefficient (*D*) analysis further quantifies the dynamic behavior (Figures 4d and S16). Water diffusion decreases continuously from 7.75 × 10^–2^ Å^2^ ps^–1^ for 2FG-P to 5.35 × 10^–2^ Å^2^ ps^–1^ for 12FG-P, consistent with suppressed water penetration. Meanwhile, Na^+^ diffusivity increases from 1.12 × 10^–2^ Å^2^ ps^–1^ (2FG-P) to 1.42 × 10^–2^ Å^2^ ps^–1^ (12FG-P) because weaker backbone binding reduces ion trapping. Interaction energy (Δ*G*) calculations (Figures 4e and S17) support these trends: binding affinities for Na^+^ and H_2_O become progressively weaker with longer fluorocarbon bridges, in agreement with RDF and distance analyses.^47^

### 2.7 Film Crystallinity and Morphology

The influence of fluorocarbon hydrophobic bridges on film morphology and molecular packing was investigated by using atomic force microscopy (AFM) and two-dimensional grazing-incidence wide-angle X-ray scattering (2D-GIWAXS). As shown in Figures 5a and S18, all polymers form smooth and continuously thin films. The root-mean-square (RMS) roughness decreases from 1.92 nm (2FG-P) to 0.82 nm (8FG-P) and slightly rebounds to 1.01 nm for 12FG-P. Upon electrochemical doping (Figure S19), the changes in RMS roughness follow a similar trend: 2FG-P shows the largest increase (+0.46 nm), whereas 8FG-P and 12FG-P exhibit only minor increases of 0.09 and 0.08 nm, respectively. These results indicate that longer fluorocarbon bridges suppress the morphological and structural reconstruction during doping.

**Figure 5:**
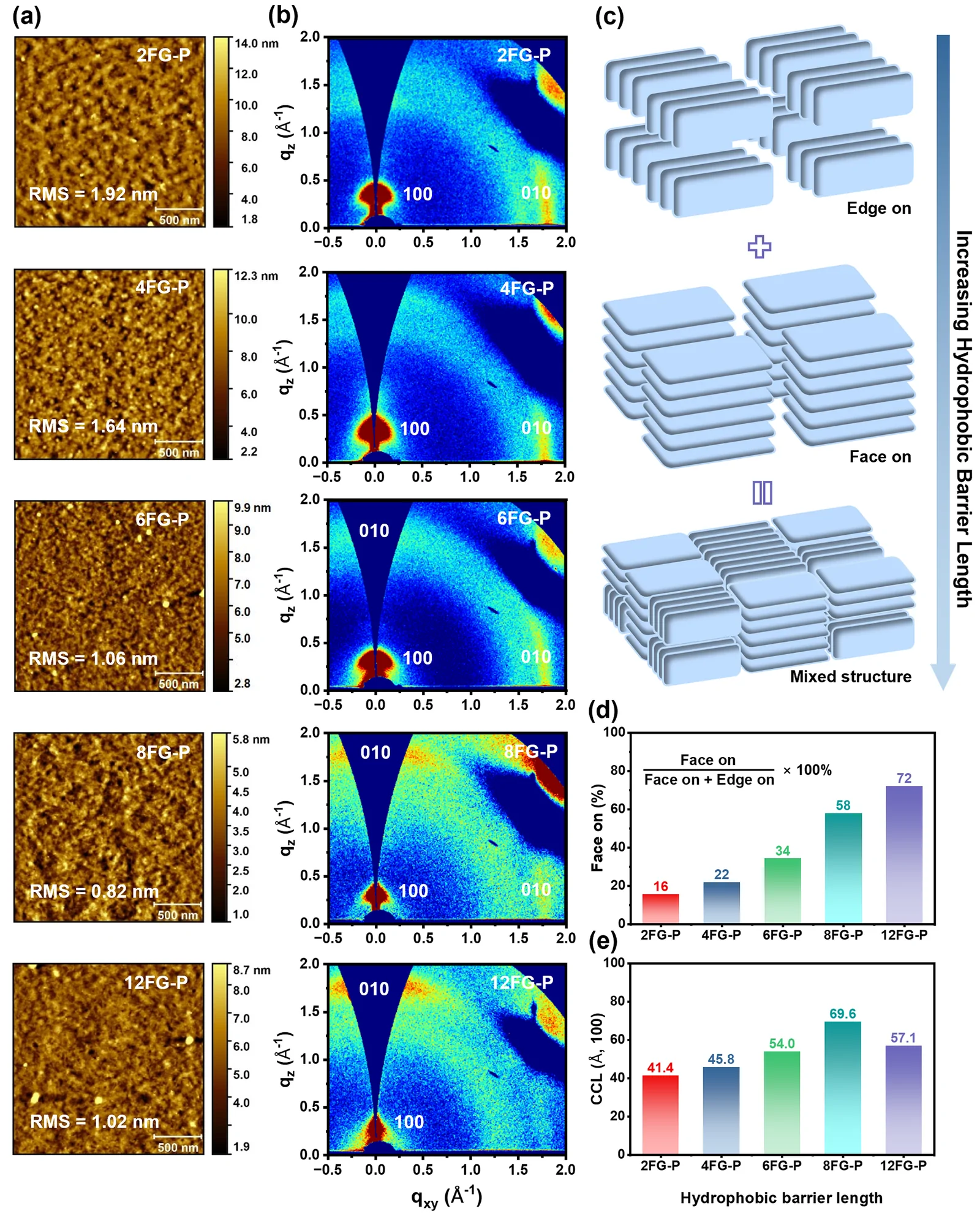
(a) AFM height images of 2*n*FG-P films. (b) 2D-GIWAXS images of 2*n*FG-P films. (c) Schematic illustration of the effect of increased length of the fluorinated hydrophobic bridge on the microstructure. (d) The ratio of the face-on and components of polymer thin films. (e) Correlation of crystal coherence length (CCL, 100) with the length of the fluorinated hydrophobic bridge.

2D-GIWAXS measurements reveal distinct evolution in molecular orientation and packing with increasing the fluorocarbon bridge length (Figures 5b and S20). Polymers with short chains (2FG-P, 4FG-P, 6FG-P) adopt a predominant edge-on texture, characterized by a strong (100) lamellar scattering in the out-of-plane (OOP) direction and an intense (010) π–π stacking reflection in the in-plane (IP) direction. In sharp contrast, 8FG-P and 12FG-P exhibit an additional (010) π–π stacking signal in the OOP direction, accompanied by weakened IP scattering (Figure 5c). This result indicates a transition toward a mixed face-on/edge-on orientation, which is favorable for three-dimensional (3D) charge transport in OECTs. To accurately evaluate this orientation difference quantitatively, we obtained pole figures along the azimuthal angle of the (010) reflection and extracted the integrated (010) peak area (Figure S21) to analyze the relative proportions of these two orientations (Table S3) following literature precedents.^89,90^ As shown in Figure 5d, with the increase in the length of the fluorocarbon hydrophobic bridge, the proportion of face-on orientation in the polymers increases from 16% for 2FG-P to 72% for 12FG-P, while the proportion of edge-on orientation decreases correspondingly from 84% to 28%. Crystal coherence lengths (CCL) calculated via the Scherrer equation further quantify crystalline ordering (Figure 5e, Table S4). The (100) lamellar CCL increases continuously from 41.4 Å (2FG-P) to 69.6 Å (8FG-P), indicating enhanced long-range lamellar ordering driven by stronger fluorine–fluorine interactions.^91,92^ However, the CCL drops to 57.1 Å for 12FG-P, suggesting that excessive fluorination hinders chain assembly. Simultaneously, the (010) π-π CCL decreases gradually with increasing (CF_2_)*_n_* length from 45.1 Å (2FG-P) to 28.7 Å (12FG-P).

Considering that electrochemical doping inevitably induces structural rearrangement and potential crystalline order degradation in OMIEC films, doped-state GIWAXS measurements were conducted to elucidate bridge-length-dependent microstructural evolution upon ion insertion (Figures S22, S23 and Table S4). Distinct structural disparities were observed across the fluorinated polymer series after electrochemical doping in 0.1 M NaCl electrolyte. All polymer films exhibited enlarged lamellar spacing following doping; Specifically, 2FG-P, 4FG-P, and 6FG-P displayed substantial lamellar expansion of 1.2 Å, 1.1 Å, and 0.9 Å, respectively. In stark comparison, 8FG-P and 12FG-P analogues exhibit far lower spacing increments of merely 0.4 Å and 0.3 Å, respectively. Such differences originate from the intercalation of hydrated ions and water molecules within interchain domains. Longer fluorocarbon bridges impart prominent hydrophobicity, which effectively restricts water penetration and suppresses excessive lamellar dilation.^88,93^ Interestingly, unlike the lamellar stacking, the π–π stacking distance even slightly contracts after doping. Quantitative analysis shows that the magnitude of π–π stacking contraction in this polymer series decreases progressively with increasing (CF_2_)*_n_* length, ranging from 0.08 Å to 0.04 Å (Table S4). Notably, in most polymers with OEG side chains, electrochemical doping induced severe film swelling that disrupts the tight π–π stacking, ultimately leading to reduction of charge transport.^36–39^ This subtle contraction is likely attributed to backbone doping/charging enhancing planar quinoid character, thus π–π interactions, while the cation is localized far from the backbone; however, enhanced intercalations cannot fully offset the redox-triggered decline in crystallinity.^94,95^ Furthermore, GIWAXS was used to quantitatively evaluate structural stability after doping. 8FG-P and 12FG-P retained well-preserved crystalline domains, with marginal CCL attenuation to 68.0/37.5 Å and 55.9/28.0 Å for CCL_(100)_/CCL_(010)_, respectively (Table S4). In contrast, short-bridge polymers suffered severe crystalline deterioration: 2FG-P, 4FG-P, and 6FG-P exhibited pronounced CCL degradation, with their post-doping CCL_(100)_/CCL_(010)_ declining to 36.1/38.0 Å, 42.4/39.1 Å, and 51.8/36.1 Å. These structural differences unambiguously verify that elongated fluorocarbon hydrophobic bridges constrain electrolyte-induced swelling, stabilize intrinsic crystalline frameworks, and mitigate doping-caused microstructural destruction. Collectively, these undoped and doped-state GIWAXS results offer microstructural insights that help rationalize the superior performance and operational stability of medium- and long-bridge polymers, particularly for the optimized 8FG-P system. Mechanistically, the larger CCL and more balanced face-on/edge-on orientation ratio of 8FG-P collectively extend 3D charge transport pathways and enhance mobility,^82,96,97^ while constrained swelling retains interchain packing to sustain operational stability, though this may result in decreased volumetric capacitance.

### 2.8 OECTs Device Fabrication and Evaluation

Planar organic electrochemical transistors (OECTs) were fabricated to evaluate the mixed ionic–electronic transport properties of the 2*n*FG-P polymer series (Figure S24). Devices were fabricated with Au source/drain electrodes (channel length *L* = 30 μm, width *W* = 500 μm), and polymer films were spin-coated directly from HFIP solution without post-treatment to preserve intrinsic behavior. Detailed device fabrication procedures are provided in the Experimental Section. All OECTs were tested in ambient conditions using 0.1 M NaCl as the electrolyte and Ag/AgCl as the gate electrode. Transfer and output curves confirm typical n-type accumulation-mode operation under positive gate bias (Figures 6a and S25, S26). Notably, the OECT transfer curves of all polymers show negligible hysteresis, hindering fast response kinetics. As shown in Figure 6b, the OECTs based on 8FG-P exhibit the highest maximum drain current (*I*_D,max_ = 1.02 mA) and peak transconductance (*g*_m,max_ = 4.29 mS), significantly outperforming 2-6FG-P and 12FG-P counterparts.

**Figure 6:**
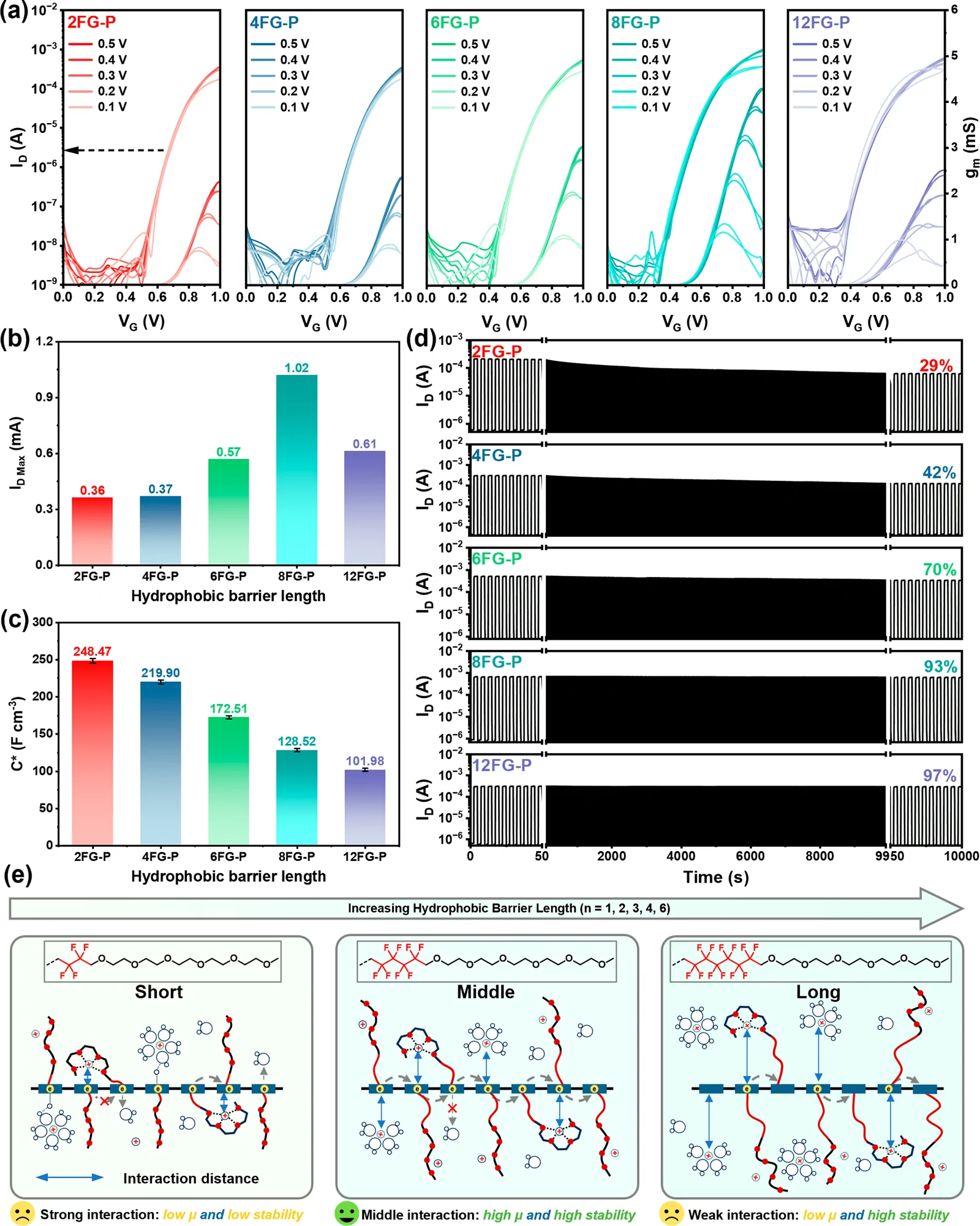
(a) Transfer and transconductance characteristics of 2*n*FG-P-based OECTs. (b) The maximum output current (*I*_D,max_) of 2*n*FG-P under 1.0 V bias. (c) Capacitance as a function of polymer volume for 2*n*FG-P under 1.0 V bias. (d) Long-term operational stability of the devices pulsed with a 1.0 V gate pulse 2.5 s in duration for 2*n*FG-P OECT polymer channels. (e) Schematic illustration of the interaction between Na^+^, water molecules, and the polymer backbone in fluorinated hydrophobic bridge models with varying lengths (short, middle, and long).

Furthermore, we calculated the product of electron mobility and volumetric capacitance (μC*) for each polymer using the equation: *g*_m_ = ∂*I*_D_/∂*V*_G_ = (*WdL* ^–1^)·μC*·|*V*_G_ – *V*_th_| (where *d* is the thickness of the active layer, and *V*_G_ is the gate voltage at *g*_m,max_).^98,99^ This parameter is theoretically independent of device geometry and bias, making it a critical indicator for benchmarking and comparing the performance of OMIECs. Among all polymers, 8FG-P exhibits the highest μC* of 156.71 F cm^–1^ V^–1^ s^–1^, while the μC* values of 2FG-P, 4FG-P, 6FG-P, and 12FG-P are 104.58 F cm^–1^ V^–1^ s^–1^, 112.42 F cm^–1^ V^–1^ s^–1^, 135.59 F cm^–1^ V^–1^ s^–1^, and 86.80 F cm^–1^ V^–1^ s^–1^, respectively (Table 2). These results demonstrate that 8FG-P possesses the optimal mixed ionic–electronic transport properties. Further benchmarking against literature-reported n-type OMIECs (Table S5) reveals that 8FG-P’s μC* is competitive with the top-performing counterparts reported to date.

**Table 2:** Average Polymer OECT Performance of 2*n*FG-P

Polymer	*d* [nm]^a^	*g*_m_ [mS]^b^	*V*_th_ [V]^c^	μC* [F cm^–1^ V^–1^ s^–1^]^d^	C* [F cm^–3^]^e^	μ [cm^2^ V^–1^ s^–1^]^f^	*I*_on_/*I*_off_	τ_on_/τ_off_ [ms]^g^
2FG-P	44.7 ± 1.1	2.26 ± 0.02	0.71 ± 0.008	104.58 ± 3.98	248.47 ± 3.19	0.42 ± 0.02	10^4^	3.8/2.6
4FG-P	41.8 ± 1.8	2.35 ± 0.07	0.70 ± 0.008	112.42 ± 6.61	219.90 ± 2.74	0.51 ± 0.03	10^4^	3.4/2.3
6FG-P	38.3 ± 2.0	3.03 ± 0.04	0.65 ± 0.007	135.59 ± 7.79	172.51 ± 2.08	0.79 ± 0.05	10^5^	2.8/2.2
8FG-P^h^	39.1 ± 1.4	4.29 ± 0.05	0.58 ± 0.006	156.71 ± 6.31	128.52 ± 2.26	1.22 ± 0.05	10^6^	2.6/1.5
12FG-P	40.5 ± 2.3	2.52 ± 0.06	0.57 ± 0.009	86.80 ± 5.65	101.98 ± 2.01	0.85 ± 0.06	10^4^	6.9/2.9

a The thickness of the OMIEC layer was measured by a step profilometer.

b Obtained from the transfer curve of OECTs (*g*_m_ = ∂*I*_D_/∂*V*_G_).

c Obtained from the transfer curve of the OECTs (|*I*_D_|^0.5^).

d Calculated from the formula: *g*_m_ = (*WdL* ^–1^)·μC*·|*V*_G_ – *V*_th_|.

e Obtained from EIS measurements: capacitance vs volume at −1.0 V bias.

f Calculated from μC* and the volumetric capacitance C*.

g OECTs switch on/off times were obtained from pulsed response time measurements.

h Device data of OECTs fabricated using HFIP as solvent differ from our previously reported data for OECTs prepared with chloroform.^102^

Electrochemical impedance spectroscopy (EIS) was used to decouple μ and C* (Figures 6c and S27–S29). Volumetric capacitance decreases systematically with increasing (CF_2_)*_n_* length, from 248.47 F cm^–3^ for 2FG-P to 101.98 F cm^–3^ for 12FG-P, owing to enhanced hydrophobicity. Based on the μC* obtained from transistor measurements and the C* values from EIS measurements, the electron mobility of each polymer was further determined. 8FG-P achieves the highest mobility of 1.22 cm^2^ V^–1^ s^–1^, far exceeding 2FG-P (0.42 cm^2^ V^–1^ s^–1^), 4FG-P (0.51 cm^2^ V^–1^ s^–1^), 6FG-P (0.79 cm^2^ V^–1^ s^–1^), and 12FG-P (0.85 cm^2^ V^–1^ s^–1^). Its mobility also rivals that of most state-of-the-art n-type OMIECs (Table S5). However, C* decreases with increasing fluorocarbon bridge length. Therefore, it is essential to optimize the balance between C* and μ rather than maximizing either metric individually to achieve favorable overall performance, an observation in line with recent literature.^100,101^

Additional key OECT metrics were systematically evaluated to quantify switching behavior, threshold characteristics, and operational stability. The average threshold voltage (*V*_th_) gradually decreases with increasing (CF_2_)*_n_* length: 2FG-P (0.71 V) > 4FG-P (0.70 V) > 6FG-P (0.65 V) > 8FG-P (0.58 V) >12FG-P (0.57 V) (Figure S30), indicating that deeper-lying LUMO levels and improved structural ordering facilitate easier electrochemical doping. In addition, the OECTs based on 8FG-P exhibit excellent switching properties with a higher *I*_on_/*I*_off_ ratio of more than ∼10^6^, notably higher than the 10^4^–10^5^ range observed for 2FG-P, 4FG-P, 6FG-P, and 12FG-P-based devices. Similarly, the OECTs based on 8FG-P exhibit the fastest response speed, with τ_on_/τ_off_ = 2.6/1.5 ms (Figure S31). By contrast, 2FG-P, 4FG-P, and 6FG-P-based devices show slower switching (3.8/2.6 ms, 3.4/2.3 ms, 2.6/1.5 ms), with the 12FG-P displaying the slowest response (6.9/2.9 ms). The moderate fluorocarbon bridge in 8FG-P accelerates Na^+^ diffusion by locally concentrating hydrated ions near the backbone, whereas the overly long and hydrophobic 12FG bridge impedes ion penetration and slows doping kinetics.

Long-term operational stability tests were performed on the five polymer-based OECT devices to investigate the effect of the fluorocarbon hydrophobic bridge length on device stability. The test conditions were as follows: a square-wave gate voltage (*V*_G_) ranging from 0 to 1.0 V with a pulse width of 2.5 s was applied for 10,000 s (a total of 2,000 cycles), while the drain-source voltage (*V*_D_) was kept constant at 0.5 V. As shown in Figure 6d, after continuous cycling, device current retention increases dramatically with fluorocarbon bridge length: 29% (2FG-P), 42% (4FG-P), 70% (6FG-P), 93% (8FG-P), and 97% (12FG-P). Note that lowering *V*_G_ to 0.9 V improves the long-term operational stability of 8FG-P, with 88% of the initial current retained after 50,000 s of on–off switch cycling (Figure S32). Implantable device deployment requires further optimization of the operational lifetime, which could be achieved by engineering the contacts, the device geometry, electrolyte composition, and biasing conditions.^33,103,104^ Comparing 8FG-P device data to those of n-type OMIECs functionalized with conventional OEG side chains indicates that very few of them exhibit similar cyclic stability; however, they show reduced device performance (Table S6). These results suggest that fluorocarbon hydrophobic bridges effectively suppress water-induced swelling, morphological disruption, and backbone degradation during repeated electrochemical switching.

Finally, since the molecular weights of the 2*n*FG-P series increase with increasing bridge length (see Table 1), we synthesized additional batches of 8FG-P and 12FG-P with lower molecular weights and comparable to those of 2FG-P, 4FG-P, and 6FG-P. Specifically, batches of 8FG-P medium (8FG-P-M_Mn_, *M*_n_ ∼ 39) and low (8FG-P-L_Mn_, *M*_n_ ∼ 21 kDa) and 12FG-P medium (12FG-P-M_Mn_, *M*_n_ ∼ 27) and low (12FG-P-L_Mn_, *M*_n_ ∼ 16 kDa) were obtained for comparative characterization (Figures S33, S34 and Table S7). As summarized in Table S7, the μC* values of 8FG-P-M_Mn_ and 8FG-P-L_Mn_ are 154.19 and 152.86 F cm^–1^ V^–1^ s^–1^, respectively, comparable to the value of 156.71 F cm^–1^ V^–1^ s^–1^ obtained for the high-molecular-weight 8FG-P. Similarly, the high-, middle-, and low-molecular-weight 12FG-P samples exhibit μC* values of 86.80, 85.25, and 87.71 F cm^–1^ V^–1^ s^–1^, respectively. Moreover, both middle- and low-molecular-weight 8FG-P and 12FG-P retain more than 90% operational stability (Figure S33) compared with their parent high-molecular-mass counterparts. Overall, these molecular-weight-matched samples exhibit OECT performance essentially identical to that of their higher-molecular-weight counterparts, ruling out molecular weight as a significant confounding factor. Therefore, the observed differences in charge transport, capacitive behavior, and operational stability across the 2*n*FG-P series are unlikely to arise from variations in molecular weight and instead can be attributed to differences in fluorocarbon bridge length.

As summarized in Figure 6e, the stable and high-performance OMIECs design we report here follows a moderate-interaction principle. Short fluorocarbon bridges (2-6FG) do not prevent excessive binding, ion trapping, and severe swelling, yet their mobility remains higher than that of the corresponding fluorocarbon bridge-free OMIECs (eg-DPP-CNTVT, 0.20 cm^2^ V^–1^ s^–1^).^102^ The overly long bridge (12FG) enhances hydrophobicity that suppresses water incorporation but prevents efficient redox chemistry. However, the medium-length fluorocarbon bridge (8FG) achieves an optimal performance balance: sufficiently weak non-specific binding enables fast ion transport, while controlled hydrophobicity suppresses swelling and preserves intermolecular backbone interactions upon ion exchange. This mechanism rationalizes the superior mobility and considerable stability observed experimentally in 8FG-P, offering a new design insight for next-generation n-type OMIECs.

## 3 Conclusions

In summary, inspired by the insulating and protective function of myelin sheaths in biological neural systems, we have proposed a general molecular design strategy based on a “ionic channel-hydrophobic barrier-electronic channel” architecture to improve the critical stability-performance trade-off in n-type OMIECs. By systematically varying the length of fluorocarbon hydrophobic bridges inserted between the glycolate ionic side chains and the π-conjugated backbone of DPP-CNTVT, we modulate ion–electron interactions, structural ordering, and aqueous stability.

Comprehensive experimental and computational studies reveal distinct structure-property relationships. Molecular dynamics simulations suggest that longer fluorocarbon bridges spatially separate ionic species from the conjugated backbone, weakening detrimental charge interactions and suppressing morphological disruption. In situ spectroelectrochemistry and in situ Raman measurements indicate that medium-length bridges (8FG) optimize ion transport kinetics and enable uniform polaron formation while maintaining efficient electrochemical doping. Structural analysis via GIWAXS further demonstrates that increasing the fluorocarbon bridge length promotes a favorable mixed face-on/edge-on three-dimensional (3D) packing morphology, facilitating isotropic charge transport. Concomitantly, EQCM-D and AFM characterizations show that longer fluorocarbon bridges enhance material hydrophobicity, reduce passive/active swelling, and minimize ion/water-induced degradation.

When implemented in OECT devices, the 2*n*FG-P series exhibits excellent n-type behavior. Notably, the 8FG-P derivative achieves an optimal balance: it delivers high electron mobility, superior μC*, fast switching speeds, and a high current on/off ratio exceeding 10^6^. Most critically, it exhibits exceptional operational stability, retaining 93% of its output current after 10,000 s (2,000 cycles) of continuous switching, which far exceeds the stability of shorter-barrier counterparts. This result is independent of the molecular mass of this polymer.

Overall, this work presents an alternative design strategy that draws inspiration from the hydrophobic isolation effect in myelin sheaths and spatially segregates ionic and electronic transport pathways, thereby enabling high-performance doping, efficient charge mobility, and stable operation in aqueous environments. The insights gained from this study may provide a novel molecular blueprint and preliminary design considerations for developing robust, high-performance n-type OMIECs, providing useful references for next-generation flexible and implantable bioelectronic devices.

## Supplementary Material



## Notes

Experimental section, DFT calculation, cyclic voltammetry, in situ Raman spectroscopy, contact angle measurement, dielectric constant, MD simulation, AFM measurement, grazing-incidence wide-angle X-ray scattering, OECT device, electrochemical impedance spectroscopy, molecular design and synthesis, NMR spectra (Figures S35–S82), mass spectra (Figures S83–S98), GPC trace (Figures S99–S107), and references (PDF)
