# Supplementary material for: Myelin Sheath-Inspired Fluorocarbon Hydrophobic Bridges for Stable n‑Type Organic Mixed Ionic–Electronic Conductors
Source: J Am Chem Soc. 2026 Oct 9;148(41):44454–69. doi: 10.1021/jacs.6c12196 (PMC13693380; doi:10.1021/jacs.6c12196)
Supplement: Supplementary file 1 [file ja6c12196_si_001.pdf]

Supplementary Information

Myelin Sheath-Inspired Fluorocarbon Hydrophobic Bridges for Stable n-Type Organic Mixed Ionic–Electronic Conductors

Wang Feng,[1] Shaodan Cai,[1] Chao Deng,[1] Bei Zhang,[1] Mengge Wu,[3] Jianhua Chen[*, 1, 4] and Antonio Facchetti[*, 2, 3]

1 Key Laboratory of Medicinal Chemistry for Natural Resource, Ministry of Education, School of Chemical Science and Technology, Yunnan University, Kunming 650091, China

2 Department of Materials, The Hong Kong University of Science and Technology, Hong Kong 999077, China

3 School of Materials Science and Engineering, Georgia Institute of Technology, Atlanta, Georgia 30332, United States

4 Southwest United Graduate School, Kunming 650092, China

***Corresponding authors.**

E-mail addresses: chenjianhua@ynu.edu.cn (J. Chen); afacchetti@ust.hk (A. Facchetti).

Contents:

1. **Experimental and Methods** 1
2. **DFT Calculations** 10
3. **Cyclic Voltammetry (CV)** 12
4. **Cyclic Voltammetry (CV) Stability** 13
5. **Variable-concentration and temperature UV-vis** 14
6. **In Situ Raman Spectroscopy** 15
7. **Contact Angle Measurement** 20
8. **Dielectric Constant** 20
9. **Comparison of the Swelling to the Previously Reported OMIECs** 21
10. **Molecular Dynamics (MD)** 23
11. **The Film Morphology and Microstructure** 27
12. **OECTs Fabrication Process** 34
13. **Transfer and Transconductance Curves** 35
14. **Output Curves** 36
15. **Electrochemical Impedance Spectroscopy** 37
16. **Threshold Voltage** 39
17. **Transient Profiles** 40
18. **Comparison of the n-Type OECTs Device Performance and Stability** 41
19. **The OECTs Performance of the Molecular Weight-Matched Samples** 48
20. **Molecular Design and Synthesis** 50
21. **NMR Spectra** 63
22. **High Resolution Mass Spectrometry** 87
23. **GPC Trace** 95
24. **References** 100

1. Experimental and Methods

1.1 Reagents and Materials Preparation: All reagents and materials used in this study were purchased directly from chemical suppliers, and all solvents were of ACS grade and purified via distillation to obtain anhydrous grade.

1.2 NMR characterization: ^{1}H NMR, ^{13}C NMR, and ^{19}F NMR spectra were recorded on a 400 MHz Bruker Avance spectrometer using deuterated $CDCl_3$ as solvent and tetramethylsilane (TMS) as an internal standard.

1.3 Mass spectrometry characterization: The mass spectra of the materials (except polymers) were recorded on Agilent 1100 HPLC-TOF high-performance liquid chromatography-mass spectrometry. Molecular weights of polymers were measured using an Agilent 1260 Infinity II GPC system. All polymers tested were prepared in hexafluoroisopropanol (HFIP) at a concentration of 2 mg/mL, filtered through a 0.22 μm polyvinylidene fluoride (PVDF) filter, and then injected into the autosampler at 30 ^{o}C. Hexafluoroisopropanol was used as the mobile phase, and polystyrene was employed as the reference material.

1.4 Density functional theory (DFT) calculation: Density functional theory (DFT) calculations were performed using three repeating units. To simplify the calculation process, methoxy groups were used to replace ethylene glycol side chains of the same length as the calculation model; meanwhile, the B3LYP/6-31G(d,p) calculation model was adopted to optimize the molecular structure. All visual images were processed using Gauss View and VMD (Visual Molecular Dynamics) software. Secondly, the Gaussian 16 software package was also used for Raman peak assignment and geometric structure analysis.[1] All simulations were conducted on isolated molecules in the gas phase, with geometry optimizations carried out at the B3LYP/6-31+G(d,p) level of theory. To optimize the calculation process, the model was constructed based on a repeating unit of DPP-CNTVT as the main chain structure, with the side chains replaced by methyl groups.[2] Vibrational frequencies were obtained from Raman spectral simulations, and peak assignments were visualized using Gauss View software. Raman spectra for the polaron state were also computed under identical conditions.

1.5 Optical absorption spectra: The Ultraviolet-Visible-near-infrared absorption spectrum of 2nFG-

P series OMIECs in HFIP solution and film were measured by Shimazu UV-3600i UV-Visible-near-infrared spectrophotometer. The optical band gaps of each material are calculated using the following formula:

$$E_g^{opt} = 1240/\lambda_{onset} \tag{1}$$

1.6 Cyclic Voltammetry (CV): Cyclic voltammetry (CV) measurements were performed on a CHI760E A21731 instrument with a three-electrode cell system in an N_2 saturated 0.1 M tetra-n-butylammonium hexafluorophosphate (n-Bu_4NPF6) solution in acetonitrile with a scanning rate of 100 mV/s at room temperature. An Ag/AgCl electrode, platinum wire, and material-coated glassy carbon electrode were used as the reference electrode (RE), counter electrode (CE), and working electrode (WE), respectively. The Ag/AgCl reference electrode was calibrated using a Fc/Fc^+ redox couple as an external standard, whose oxidation potential was set at −4.8 eV with respect to the zero-vacuum level. The HOMO and LUMO energy levels of polymer materials were calculated by the oxidation peak potential (E_{OX}) and reduction peak potential (E_{RE}), respectively.

$$E_{HOMO} = -(E_{OX} - E_{Fc/Fc^+} + 4.8)eV \tag{2}$$

$$E_{HOMO} = -(E_{RE} - E_{Fc/Fc^+} + 4.8)eV \tag{3}$$

$$(E_{Fc/Fc^+} = 0.45eV)$$

1.7 Cyclic Voltammetry (CV) Stability: Similar to the cyclic voltammetry (CV) measurement process, only the type of electrolyte was different. a 0.1 M deoxygenated sodium chloride solution was used as the electrolyte solution in the experiment. Similarly, the polymer material was coated on the surface of the working electrode, and 200 cyclic voltammetry measurements were performed in the potential range of 0.4 V to −1.0 V to observe the change rule of the reduction current of the polymer material with the increase of cycle number.

1.8 In Situ Spectroelectrochemistry: The polymer films were spin-coated onto an ITO-coated glass substrate and immersion it into a cuvette containing 0.1 M NaCl electrolyte solution, which was then placed in the cuvette together with an Ag/AgCl electrode. A KEITHLEY 2401 Source Meter was employed to in situ monitor the UV–vis−NIR spectra of the polymer films at various voltages, aiming to investigate the neutral and charged states of the films during the electrochemical doping process.

Ag/AgCl served as the negative electrode and ITO as the positive electrode. The spectral measurements were carried out using a Shimazu UV-3600i UV-visible near-infrared spectrophotometer, with the light beam passing through the cuvette that held the electrolyte aqueous solution and the polymer/ITO glass samples. For baseline correction, a background spectrum was measured using a cuvette containing electrolyte and an ITO glass slide prior to the formal experiment. Each UV-visible spectrum was acquired 30 seconds after a specific potential was applied, and the applied potentials ranged from 0 to −1.0 V.

1.9 In Situ Raman Spectroscopy: The polymer thin films were spin-coated onto the surface of silicon wafers evaporated with 3 nm chromium (Cr) and 60 nm gold (Au), and the silicon wafers were then immersed into an electrolytic cell filled with 0.1 M sodium chloride (NaCl) electrolyte solution. In the experiment, an Ag/AgCl electrode was inserted as the negative electrode, and the gold-plated silicon wafer spin-coated with the polymer material was used as the positive electrode. A KEITHLEY 2401 Source Meter was employed to in situ monitor the Raman spectra of the polymer thin films at various voltages. The Raman spectral measurements were performed using a Renishaw inVia confocal Raman microscope. A Raman scattering spectrum was collected 30 seconds after applying a specific potential, with the applied potential ranging from 0 V to −1.0 V.

1.10 Electrochemical Quartz Crystal Microbalance with Dissipation Monitoring (EQCM-D): E-QCMD is a microbalance that monitors in-situ fluctuations in the mass of an electroactive film coated on a piezoelectric quartz crystal while subjected to electrochemical reactions. The analyzer can monitor the frequency shifts and dissipation changes at the 1st, 3rd, 5th, 7th, 9th, and 11th overtones, which are indicative of the changes in sample mass and softness. Initially, measurements were performed on the bare sensor in air and subsequent to the addition of 0.1 M NaCl electrolyte into the cell cavity. A significant alteration in EQCM-D signals was observed upon the introduction of the electrolyte, which resulted from the density difference between the two media (air and electrolyte), leading to considerable changes in frequency (f) and energy dissipation (D). Following the acquisition of baseline EQCM-D signals for the bare sensor under dry (air) and wet (electrolyte) conditions, the measurement was halted, the sensor chip was removed, and the polymer was directly spin-coated onto the same sensor chip. Once the frequency (f) and dissipation (D) signals stabilized ($\Delta f < 1$ Hz h^{-1}), the EQCM-

D signals were re-recorded under dry conditions and in the electrolyte. Subsequently, the "stitch data" feature of Q-Soft software was utilized to calculate the differences in f and D signals of the sensor before and after polymer thin film deposition under dry and electrolyte environments. These differences were employed to determine the surface mass change (Δm) and effective thickness (d_{eff}) of the thin film in the dry and swollen states via the Sauerbrey equation:

$$\frac{\Delta m}{A} = -\Delta f_n \frac{\rho_q V_q}{2 f_0^2 n} \approx \frac{-\Delta f_n}{n} \approx 17.9 ng / cm^2 \tag{4}$$

$$d_{eff} = \frac{\Delta m}{\rho_{eff}} \tag{5}$$

Where Δf_n is the frequency shift of the nth overtone, A is the sensor's active area (0.7854 cm^2), ρ_q is the density of quartz, V_q is the shear wave velocity in quartz, f_0 is the fundamental frequency, and n is the overtone number. Film thickness (d_{eff}) was calculated by dividing the calculated areal mass by film density (ρ_{eff}). The swelling in this paper is calculated as the percentage change of volume relative to dry volume:

$$Swelling = \frac{V_{wet} - V_{dry}}{V_{dry}} \tag{6}$$

Electrochemical doping was performed using an Autolab PGstat128N potentiostat coupled with a QSense electrochemistry module (QEM 401). The integrated three-electrode setup comprised an Ag/AgCl reference electrode, a Pt counter electrode, and the polymer-coated Au QSensor acting as the working electrode.

In addition, thick or soft films deviate from rigid Sauerbrey-limit oscillation behavior and produce energy dissipation during resonance. These films show parallel viscous and elastic (viscoelastic) responses, well described by the Kelvin–Voigt element. The complex shear modulus G^* of this element is given in Eq.7, where G* is the complex shear modulus, μ is the elastic modulus (kg m^{-1} s^{-2}), η is the viscosity (kg m^{-1} s^{-1}), and f is the measurement frequency.

$$G^* = \mu + 2\Pi i f \eta \tag{7}$$

For thick viscoelastic films, mass changes were derived by fitting the complex shear modulus with frequency and dissipation shift data from at least three overtones. All modeling and analysis were

performed in Q-Tools and D-find software. The films that soften and absorb a large amount of water under the doping potential, we applied the Kelvin–Voigt viscoelastic (VSE) model using the 3th, 5th, and 7th overtones.[3]

1.10 Water Contact Angle: The polymer solutions were deposited onto the surface of the silicon wafer substrate, and the measurements were conducted using a sessile drop contact angle analysis system (SDC-350) to characterize the wettability of the polymers.

1.11 Dielectric Constant: The dielectric constant of the polymers was characterized using a parallel-plate capacitor configuration. Impedance spectroscopy measurements were conducted over a frequency range of 1000 Hz to 10 MHz using a Solartron 1260 Gain-Phase Analyzer. All tests were performed at room temperature under ambient air conditions. Commercially available ITO-coated glass substrates were utilized as the bottom electrodes. The substrates were cleaned sequentially with aqueous soap solution, rinsed with deionized water, followed by sonication in acetone and isopropyl alcohol, and subsequently dried in an oven and treated with ultraviolet-ozone (UVO). The thin films were deposited via spin-coating from hexafluoroisopropanol (HFIP) solutions under a nitrogen atmosphere. Top silver electrodes were thermally evaporated at a base pressure of approximately $\sim 10^{-4}$ Pa.

1.12 Molecular Dynamics (MD): All-atom molecular dynamics (MD) simulations were performed to investigate the film formation process and relevant physicochemical properties of five side-chain-functionalized polymers. First, the molecular structures of the five polymers were constructed. The full electronic wavefunction of oligomeric model compounds for each polymer was calculated via the GFN2-xTB method. The restrained electrostatic potential (RESP) atomic charges for the terminal segments and central repeating unit of each polymer were fitted using the Multiwfn software package, with the constraint of zero net charge for each restricted fitting region. After charge fitting, the all-atom force field topologies of the long-chain polymers were constructed, and the validated RESP charges were assigned to the corresponding atoms of each polymer chain. The initial periodic simulation cells containing the polymer chains and corresponding counterions were built via amorphous packing using the Packmol software. The force field topologies of all components in the simulation system were then assembled and integrated using the moltemplate software.

All MD simulations were performed using the Large-scale Atomic/Molecular Massively Parallel Simulator (LAMMPS) software package. First, energy minimization was carried out using the conjugate gradient algorithm, with convergence criteria set to an energy tolerance of 1.0×10^{-6} kcal/mol and a force tolerance of 1.0×10^{-8} kcal/(mol·Å) to eliminate unreasonable atomic contacts and high-energy local structures. After optimization, a 100 ps pre-equilibration simulation was performed in the canonical (NVT) ensemble at 300 K with an integration time step of 1 fs. During this pre-equilibration stage, the simulation box was uniformly compressed along the x, y, and z axes to a final dimension of 70 Å per side, to induce initial aggregation of the polymer chains and avoid unphysical dissociation of the system prior to the annealing process. After volume adjustment, an initial 100 ps relaxation simulation was conducted in the isothermal-isobaric (NPT) ensemble at 300 K and 1 atm, to contract the simulation box to the order of magnitude of its equilibrium volume. A simulated annealing protocol was subsequently applied as follows: (1) the system was heated from 300 K to 600 K over 100 ps in the NVT ensemble; (2) the system was maintained at 600 K for 1 ns to ensure sufficient mixing and conformational relaxation of all components; (3) the temperature was gradually reduced from 600 K to 400 K over 1 ns. Upon completion of the annealing procedure, the system was slowly cooled from 400 K to 300 K over 2 ns in the NPT ensemble, with the pressure held constant at 1 atm throughout the cooling process. For all annealing and cooling stages, the Nosé-Hoover thermostat was employed for temperature control with a damping constant of 100 fs, and isotropic pressure coupling was implemented via the Nosé-Hoover barostat with a damping constant of 1000 fs. After the full annealing and cooling cycle, a second energy minimization was performed to eliminate residual local stress in the system. A final 2 ns equilibrium simulation was then carried out in the NPT ensemble at 300 K and 1 atm, to ensure the system reached full convergence in terms of total energy, volume, and density.

The simulation trajectory was visualized using post-processing software. The latter 1 ns of the 2 ns equilibrium trajectory was used for statistical analysis of atomic distances, positions, and interaction energies. Key physicochemical properties of the system were extracted, including radial distribution function (RDF) and coordination number (CN), mean squared displacement (MSD) and corresponding self-diffusion coefficients, intermolecular interaction energies, and the number density distribution of each component along the spatial axes.

The limitations of MD simulation: MD simulations were performed on neutral polymer chains

and that the results primarily describe the effects of fluorination on side chain hydration, ion partitioning, and backbone packing. These structural and solvation trends are qualitatively preserved upon doping and provide a foundation for understanding the stability differences observed across the series. A quantitative description of polaron stabilization, however, would require charged polymer models incorporating explicit electronic structure,[4] which remains technically challenging at the fully atomistic MD scale.

1.13 Atomic Force Microscopy (AFM): The surface morphology and roughness of the film were obtained by scanning the surface with a Bruker Dimension Edge atomic force microscope (AFM) in the point-contact mode. The polymer films were prepared by dissolving them in hexafluoroisopropanol and then spin-coating them onto a Si substrate.

1.14 Grazing incidence wide-angle X-ray scattering (GIWAXS): GIWAXS measurements were conducted at the BSRF-1W1A diffuse X-ray scattering station of the Shanghai Synchrotron Radiation Light Source (SSRF). GIWAXS samples were prepared by spin-coating of the 2nFG-P series OMIECs on Si and Au-coated Si substrates. For the "doped" samples, the spin-coated Au-coated Si substrates were immersed in a 0.1 M NaCl solution for electrochemical doping, while Au-coated Si substrates as the positive electrode and Ag/AgCl acts as the negative electrode. A voltage bias (–1.0 V) was applied for 1 minute, followed by blow-drying of the samples. The wavelength of the monochromatic light source of the light source was 1.54 Å. The GIWAXS sample stage is equipped with a motorized stage for the fine alignment of the sample, and the incidence angle of the X-ray beam was set to 0.1^{o} for the neat and blend films. The data were recorded by using the 2D image plate detector of Eiger 2M from Dectris, Switzerland. and the X-ray irradiation time was 5 - 30 s, dependent on the saturation level of the detector. Diffraction angles were calibrated using a CeO_2 standard (cubic, a = b = c = 5.4117 Å, and $\alpha = \beta = \gamma = 90°$). CCLs were calculated according to the following Scherrer equation:

$$CCL = 2\pi K/\Delta q \tag{7}$$

In this equation, CCL is the crystal coherence length, K is a shape factor (0.89), and Δq is the FWHM of a diffraction peak.

1.15 Electrochemical Impedance Spectroscopy (EIS): EIS measurements were performed using the same three-electrode setup as that for cyclic voltammetry. A layer of 3 nm Cr and 60 nm Au were

sequentially deposited on glass substrates via vacuum evaporation to serve as gold electrodes. The polymer solution in hexafluoroisopropanol (HFIP) was spin-coated onto the gold electrodes. The gold electrodes were then encapsulated with SU-8 photoresist, leaving only the active channels exposed. The polymer-coated gold electrode served as the working electrode (WE), platinum wire as the counter electrode (CE), Ag/AgCl electrode as the reference electrode (RE), and 0.1 M NaCl solution as the electrolyte. EIS spectra were recorded over a frequency range of 100 kHz to 1 Hz with a bias voltage of –1.0 V. Through the software EC-Lab view, the results of the Bode diagram or Nyquist diagram are fitted to the equivalent circuit, namely the Randall circuit (*Rs (Rp||C)*). Using the following formula to calculate the effective capacitance (C) and volume capacitance (C*) of the measured impedance at 1 Hz:

$$C = 1/(2\pi f\,|Z|) \tag{8}$$

$$C^{*} = C/(d \times A) \tag{9}$$

where *f* is frequency and |Z| is the magnitude of the imaginary part of the impedance. A is the area of the gold electrode (0.01 μm^2, 0.05 μm^2,and 0.1 μm^2), and measured film thickness (d) with a step meter to determine its volume capacitance (C*).

1.16 Device Fabrication and Characterization: The fabrication process of the OECTs is shown in Figure S20. OECTs were fabricated in layers on Si/SiO_2 wafers that pre-cleaned with isopropyl alcohol. First the source and drain electrode, comprising 3 nm of Cr and 60 nm of Au was deposited by thermal evaporation through a shadow mask (at a rate of approximately 0.1 Å s^{-1} and1.0 Å s^{-1}, respectively), with a channel size of W/L = 500 μm/30 μm. Then, the 2nFG-P series OMIEC solutions in HFIP (15 mg/mL) were spin-coated. Note 2FG-P, 4FG-P, and 6FG-P solution were spun at 2000 rpm while 8FG-P and 12FG-P solution is deposited at 3000 rpm to achieve an optimized OMIECs film thickness. Finally, in the channel region, a 1-2 μL drop of 0.1 M NaCl electrolyte was dispensed, with an Ag/AgCl electrode serving as the OECTs gate electrode inserted into the droplet. All OECT parameters were calculated using a minimum of five independent devices. The reported uncertainty corresponds to one standard deviation across at least 5 independent devices per sample, accounting for variations in film thickness, device fabrication, and parameter extraction.

1.17 OECT Stability Test: OECTs stability testing was conducted using a 0.1 M NaCl solution prepared with Milli-Q water (18.2 MΩ·cm) under ambient air condition, without any degassing treatment. Used a 50 μL PDMS small chamber printed by 3D technology as the storage tank for the electrolyte. The long-term operational stability test was performed either with a gate voltage pulse width of 2.5 s (on) / 5 s (off) at V_G = 0 /1.0 V and V_D = 0.5 V over the course of 10000 seconds.

2. DFT Calculations

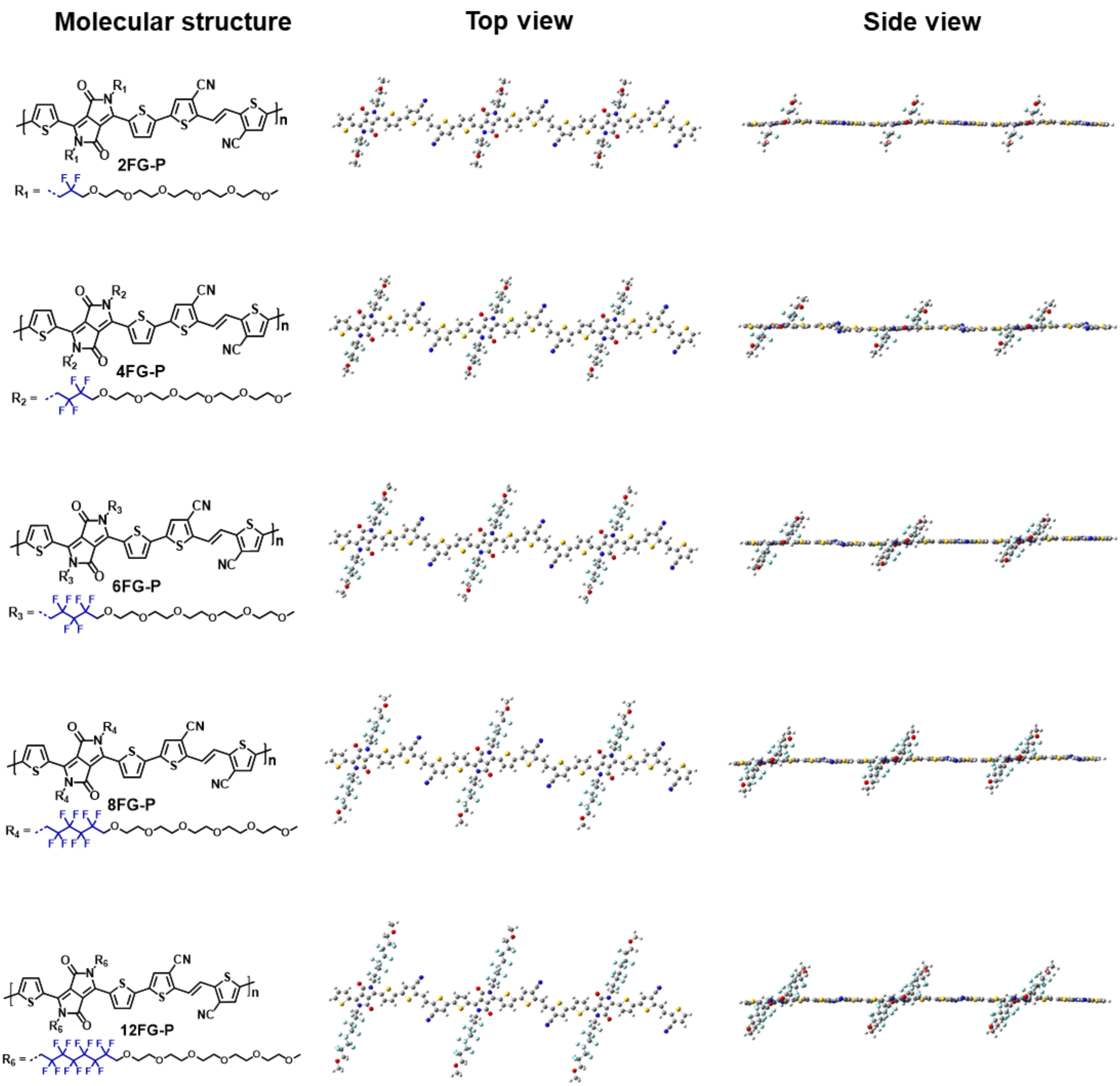

Figure S1. Molecular model after DFT calculation optimization.

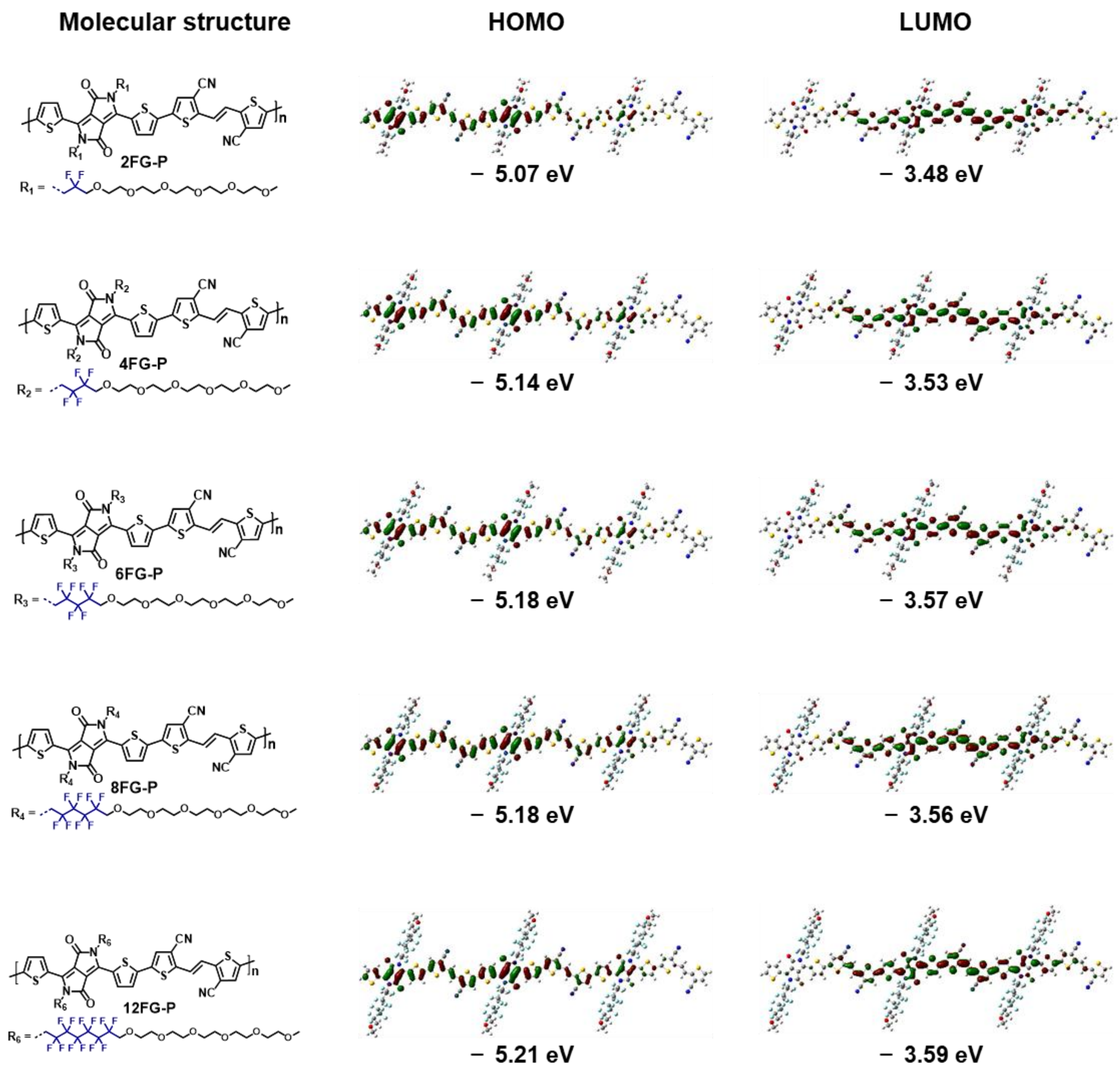

Figure S2. Frontier molecular orbitals and calculated HOMO/LUMO energy levels.

3. Cyclic Voltammetry (CV)

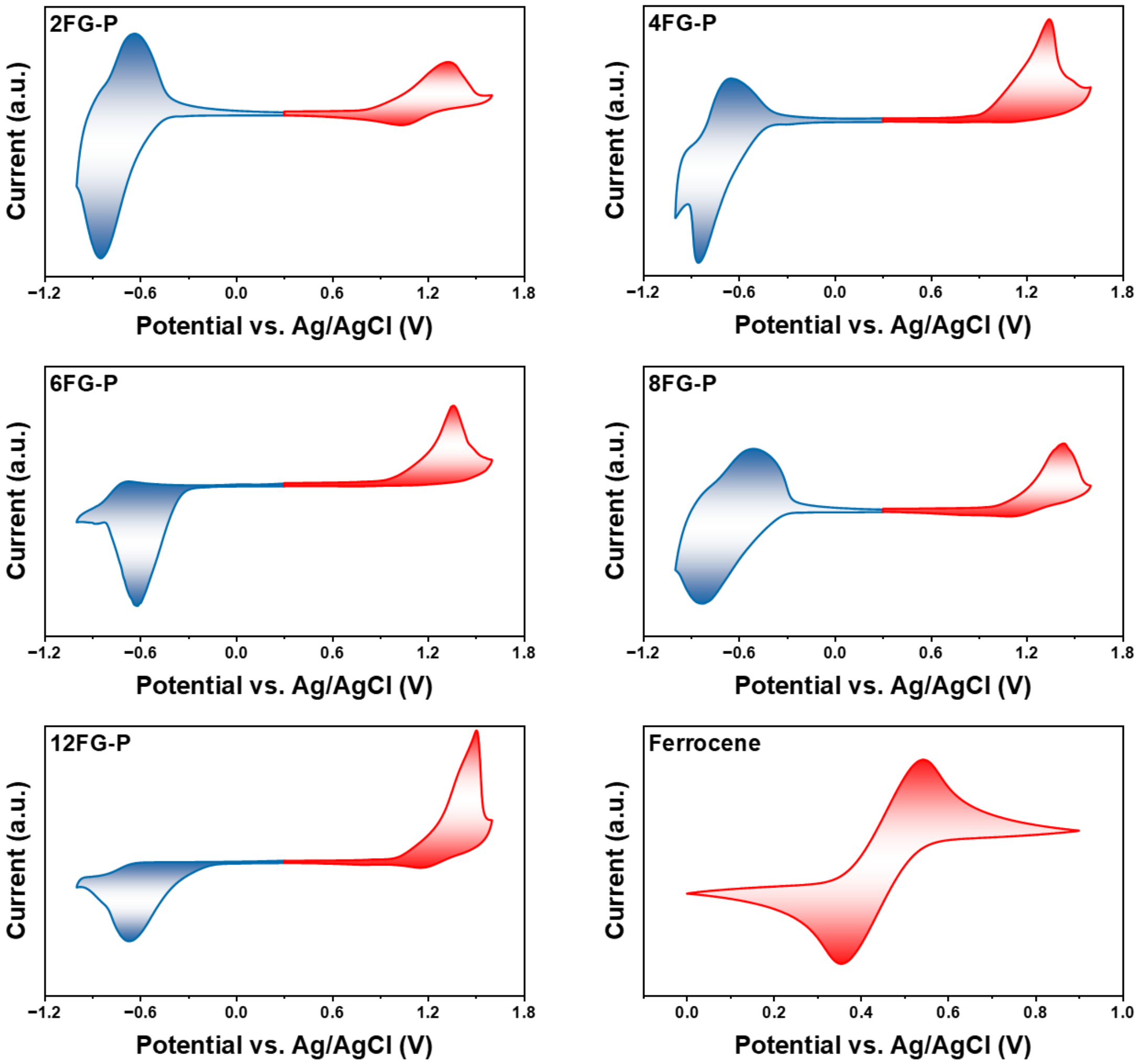

Figure S3. Cyclic voltammetry curves of the films.

Table S1 The oxidation potential and reduction potential from the CV curve, as well as the calculated HOMO and LUMO energy levels and energy bands

Polymer	E_{OX}^{onset} (V)	E_{RE}^{onset} (V)	E_{HOMO} (eV)	E_{LUMO} (eV)	E_g^{ele} (eV)
2FG-P	0.92	−0.57	−5.27	−3.78	1.49
4FG-P	1.03	−0.47	−5.38	−3.88	1.50
6FG-P	1.12	−0.36	−5.47	−3.99	1.48
8FG-P	1.14	−0.35	−5.49	−4.00	1.49
12FG-P	1.17	−0.32	−5.52	−4.03	1.49

4. Cyclic Voltammetry (CV) Stability

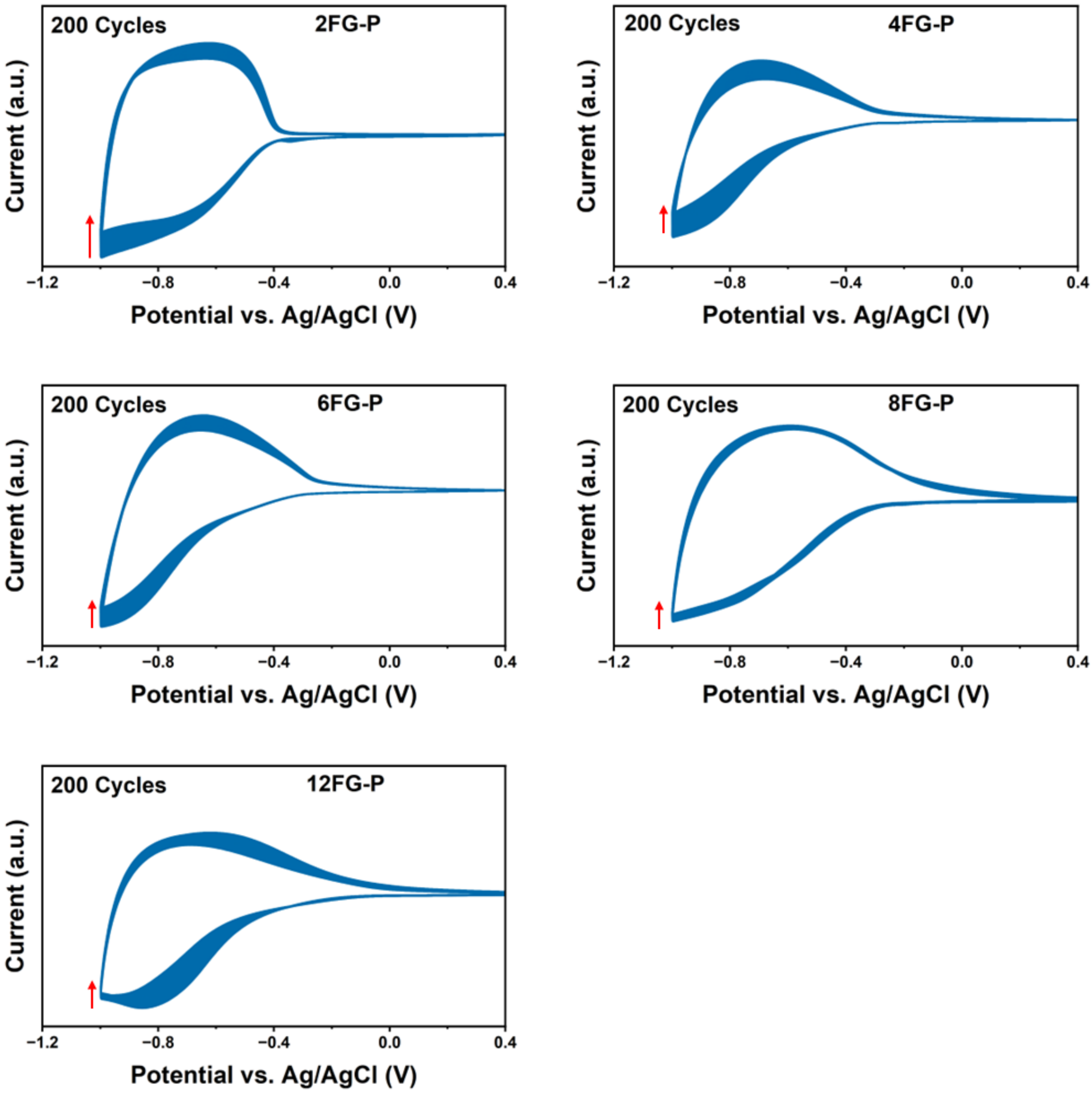

Figure S4. Investigate the electrochemical doping stability of 2nFG-P (n = 1, 2, 3, 4, and 6) through 200 cyclic voltammetry cycles with NaCl Solution (0.1 M).

5. Variable-concentration and temperature UV-vis

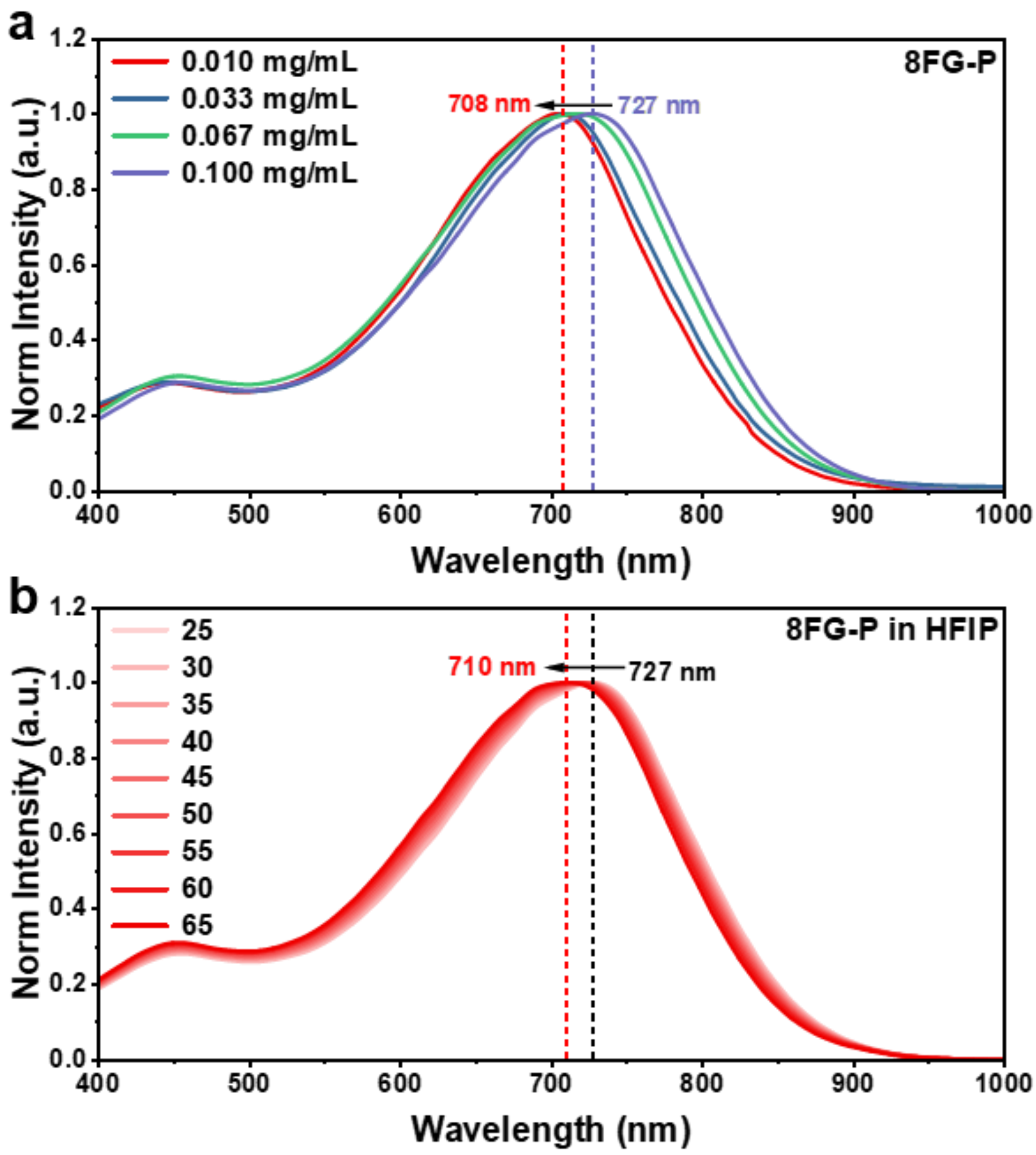

Figure S5. (a) Concentration-dependent UV–vis absorption spectra of 8FG-P in HFIP solution. (b) Variable-temperature UV–vis absorption spectra of 8FG-P in HFIP, confirming temperature-induced disaggregation behavior.

6. In Situ Raman Spectroscopy

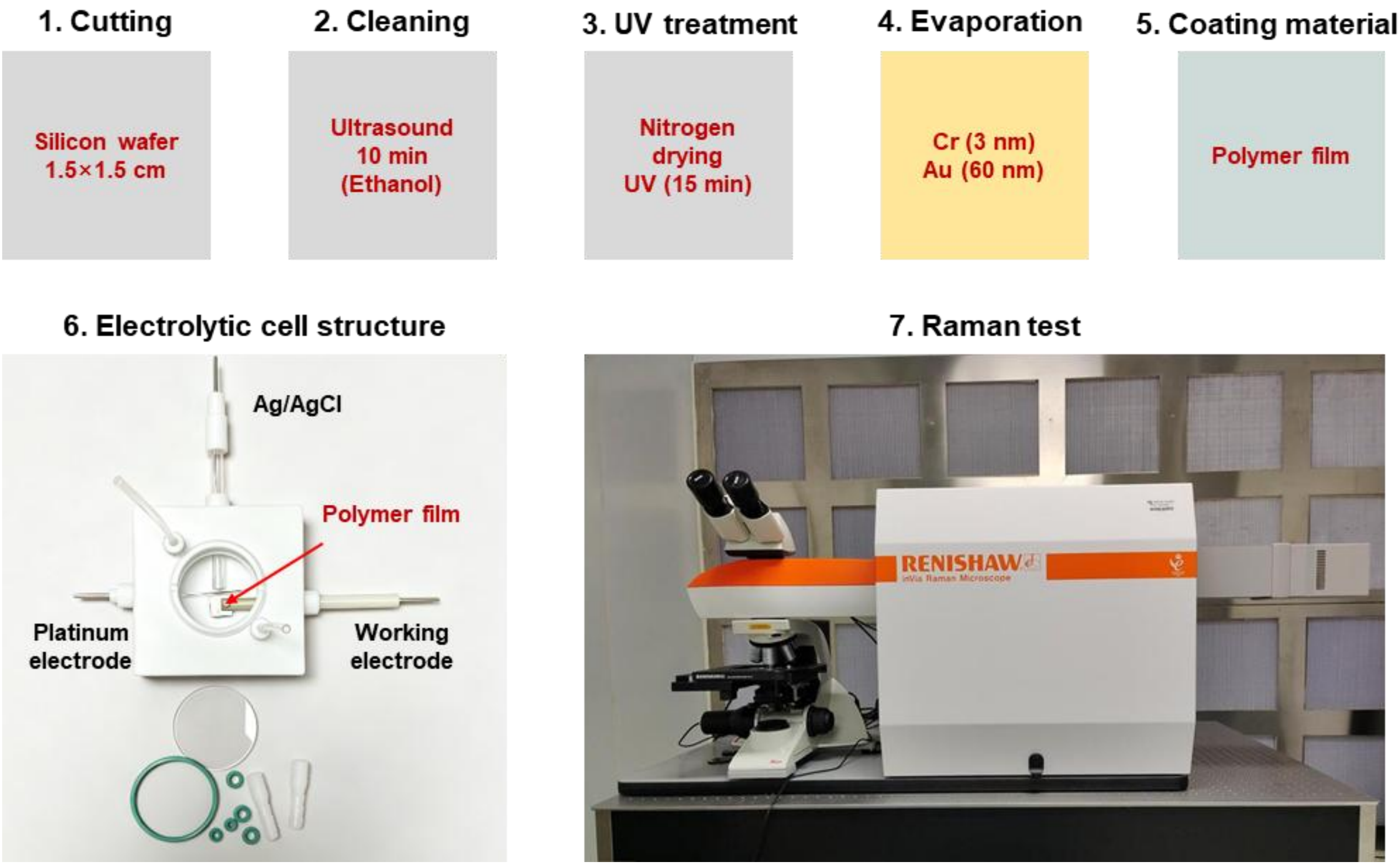

Figure S6. Flowchart of the device preparation process for Raman testing.

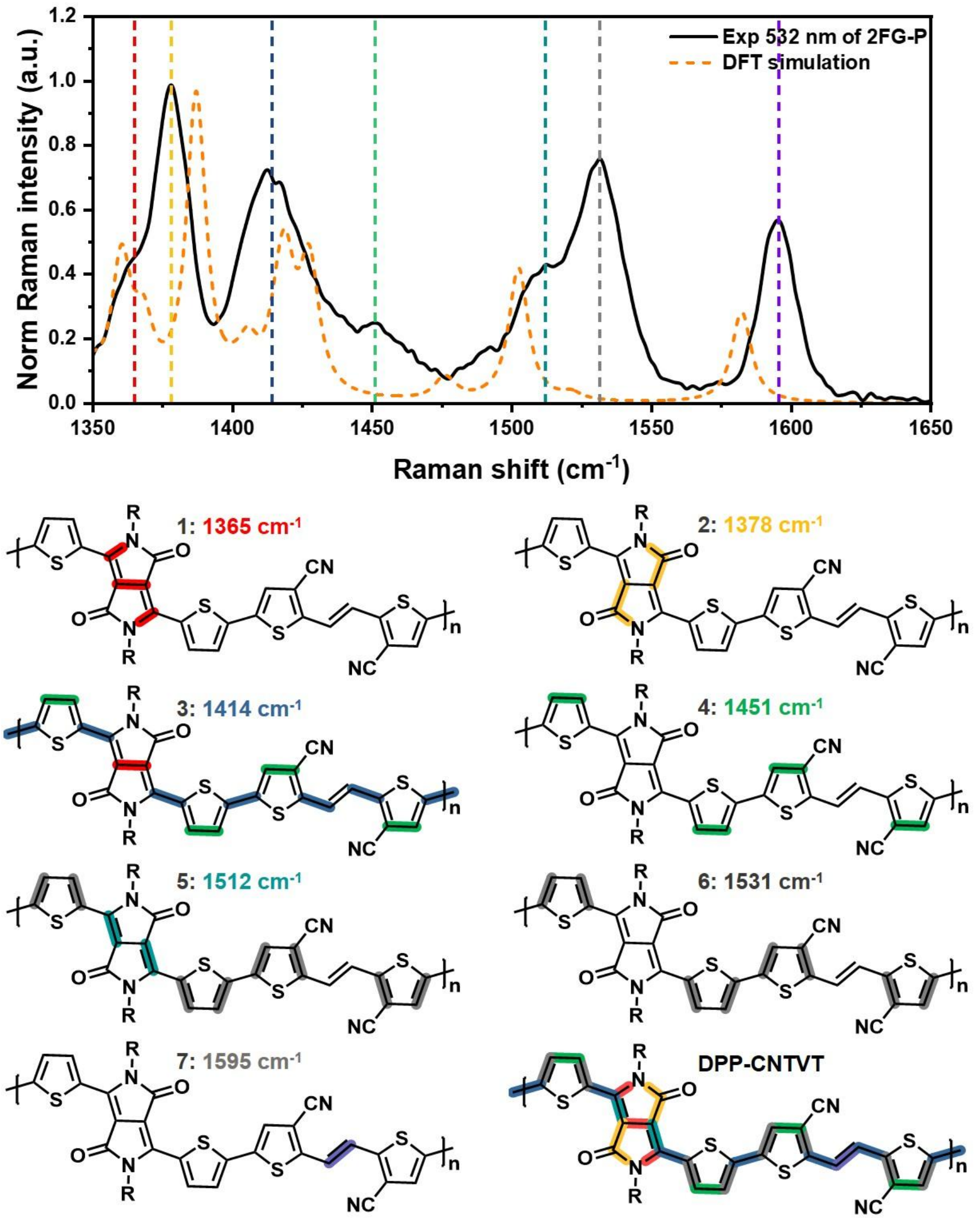

Peak	Raman shift (cm^{-1})	Vibrational modes (DPP-CNTVT)
1 = i	1365	DPP C-C$_{intra}$ and N-C
2 = ii	1378	DPP C-C$_{Carboxide}$ and N-C$_{Carboxide}$
3 = iv	1414	All backbone C-C
4 = v	1451	T C-C
5 = iii	1512	DPP and T C=C
6 = vi	1531	T C=C
7= vii	1595	V C=C

Figure S7. Vibrational peak assignment for DPP-CNTVT based on DFT-simulated Raman spectra.

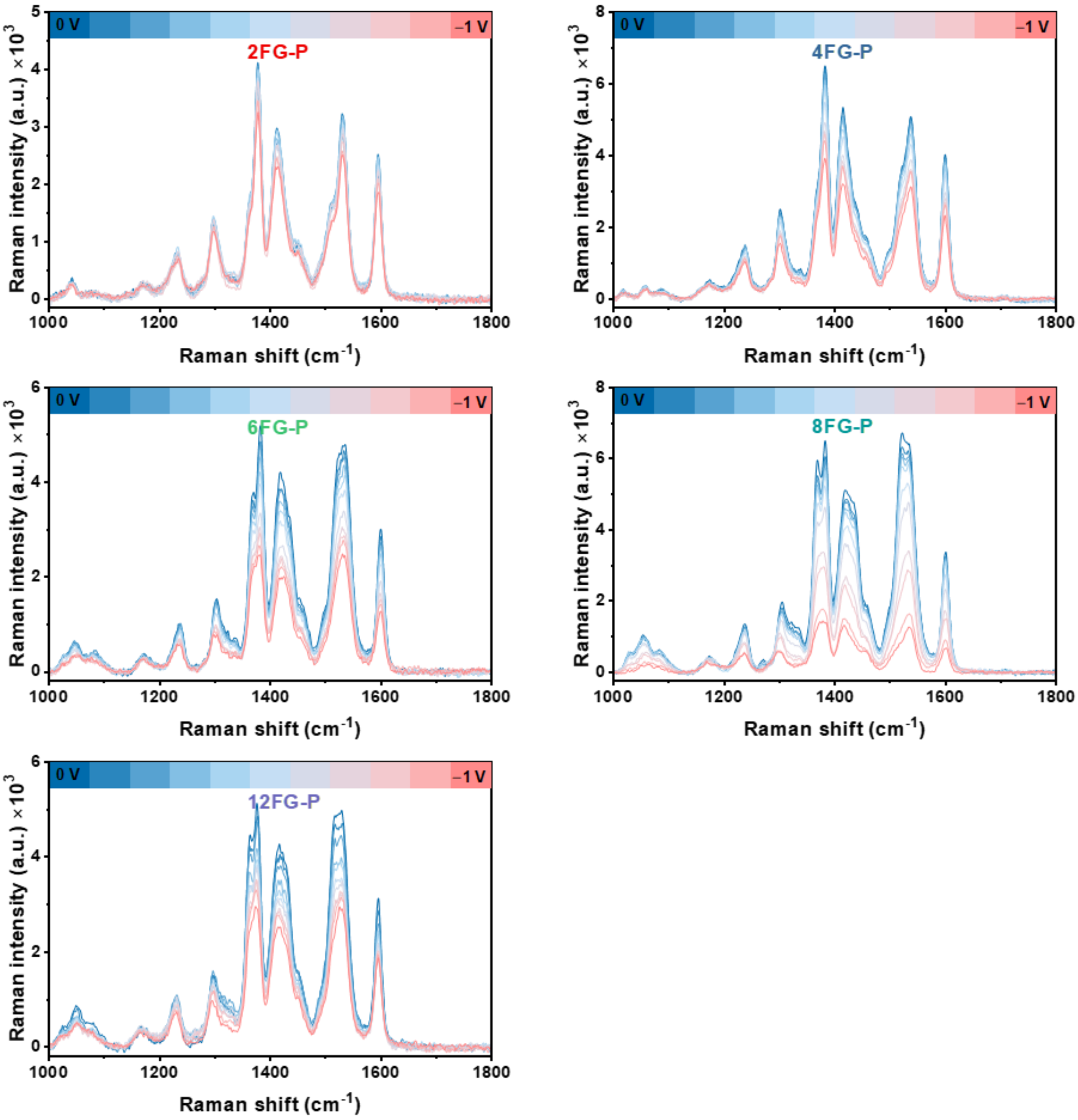

Figure S8. In-situ Raman Spectroscopy of 2nFG-P (n = 1, 2, 3, 4, and 6)

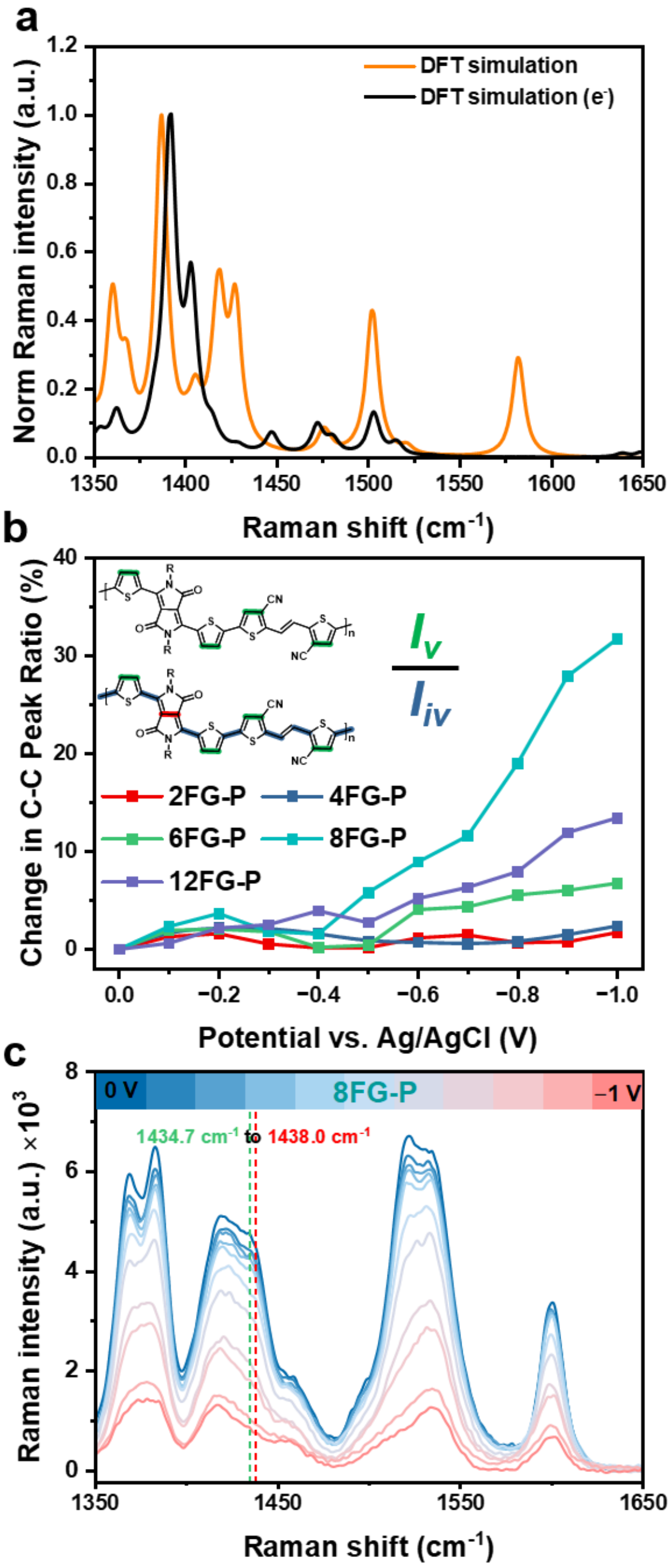

Figure S9. (a) DFT-calculated Raman spectra of DPP-CNTVT in the neutral state and singly reduced polaron state (-1). (b) Change in C-C peak intensity ratio Iv/Iiv reflecting redistribution of π-electrons along the conjugated backbone and within thiophene donor units. (c) Shift of peak v during polaron formation in 8FG-P.

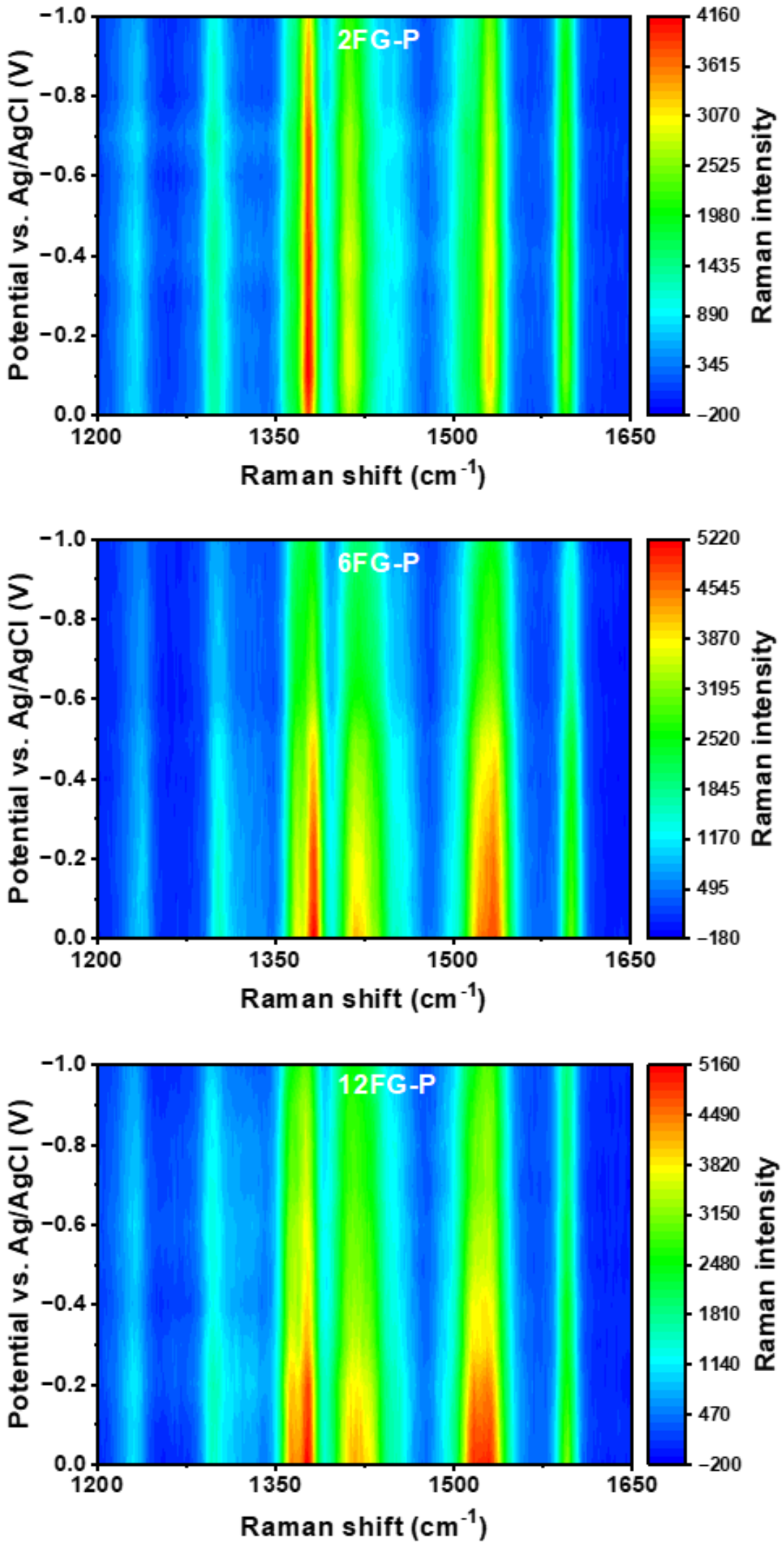

Figure S10. The potential-Raman shift heatmap of 2nFG-P (n = 1, 2, 3, 4, and 6).

7. Contact Angle Measurement

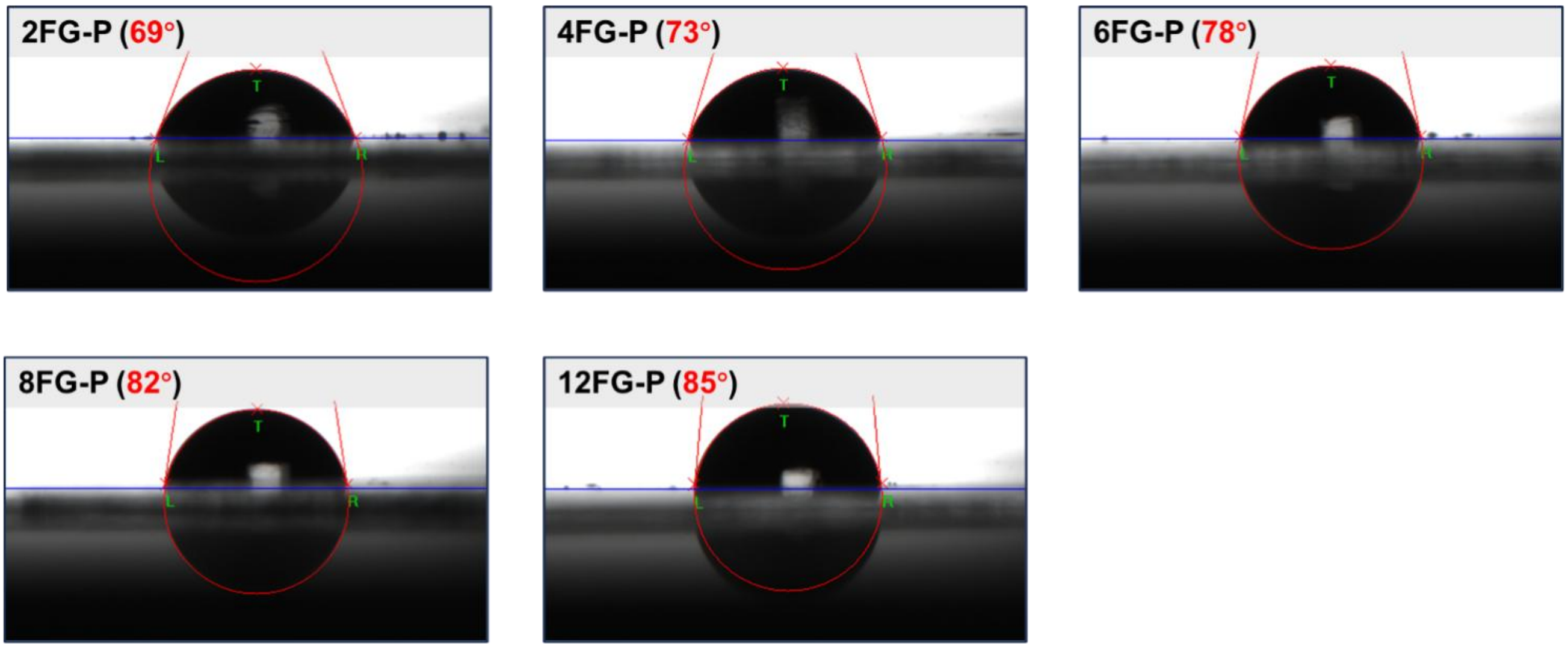

Figure S11. The water contacts angle of 2nFG-P (n = 1, 2, 3, 4, and 6).

8. Dielectric Constant

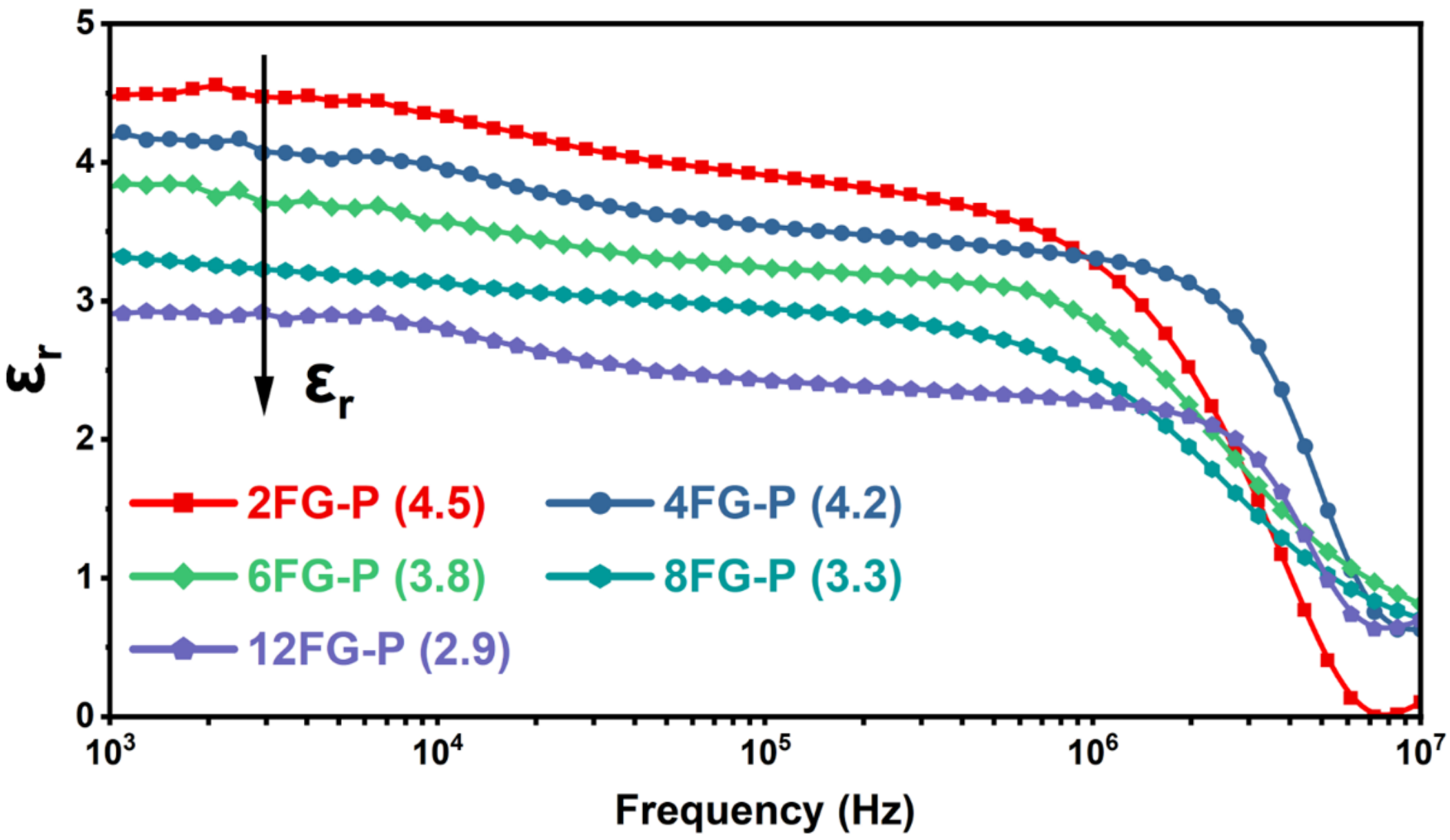

Figure S12. The dielectric constant of 2nFG-P (n = 1, 2, 3, 4, and 6).

9. Comparison of the Swelling to the Previously Reported OMIECs

Table S2 The swelling of OMIECs with traditional ethylene glycol side chains

Polymer	Passive swelling	Active swelling	ref
**PNPD**	61.4 %	6.3 %	5
**P(gTDPP2FT)**	113 %	1.3 %	
**PANDA**	153 %	\	
**pgBTTT**	16%	46%	6
**p(g2T-TT)**	12%	65%	
**p(1g-NDI-T2)**	0.7%	~50%	7
**p(2g-NDI-T2)**	5.8%	~175%	
**p(3g-NDI-T2)**	76.5%	\	
**p(4g-NDI-T2)**	>+100%	\	
**g-0%**	2%	~5%	8
**g-50%**	~7%	~10%	
**g-75%**	~9%	~33%	
**g-100%**	12%	86%	
**2g**	50%	110%	
**P(NDITEG-T)**	328%	\	9
**P(NDIDTEG-T)**	160%	\	
**P(NDIDEG-T)**	36%	\	
**P(NDIMTEG-T)**	22%	\	
**P(g3T2)**	~50%	249%	10
**P(g2T2-g4T2)**	~50%	168%	
**P(g1T2-g5T2)**	~50%	10%	
**P(g0T2-g6T2)**	~50%	4%	
**NDI-T2-L**	~35%	~180%	11
**NDI-T2-B**	~170%	~280%	

Polymer	Passive swelling	Active swelling	ref
**NDI-T-L**	~10%	~130%	
**NDI-T-B**	95%	~80%	
**TVTCN-L**	0.08%	4.7%	
**TVTCN-B**	~35%	45.3%	
**3gTT-3gT**	27%	87%	
**4gTT-2gT**	14%	70%	12
**5gTT-1gT**	90%	114%	
**p(gNDI-gT2)**	~50%	~320%	13
**p([100:0]NDI-g3T2)**	\	343%	
**p([90:10]NDI-g3T2)**	\	87%	14
**p([75:25]NDI-g3T2)**	\	11%	
**P75**	37%	7%	15
**P90**	38%	14%	16
**P100**	~72%	~85%	17
**g-(100%)**	~27%	~95%	18
**eg-DPP-CNTVT**	21.5%	44.1%	19
**2FG-P**	33%	44%	
**4FG-P**	25%	33%	
**6FG-P**	19%	29%	This work
**8FG-P**	13%	23%	
**12FG-P**	10%	13%	

10. Molecular Dynamics (MD)

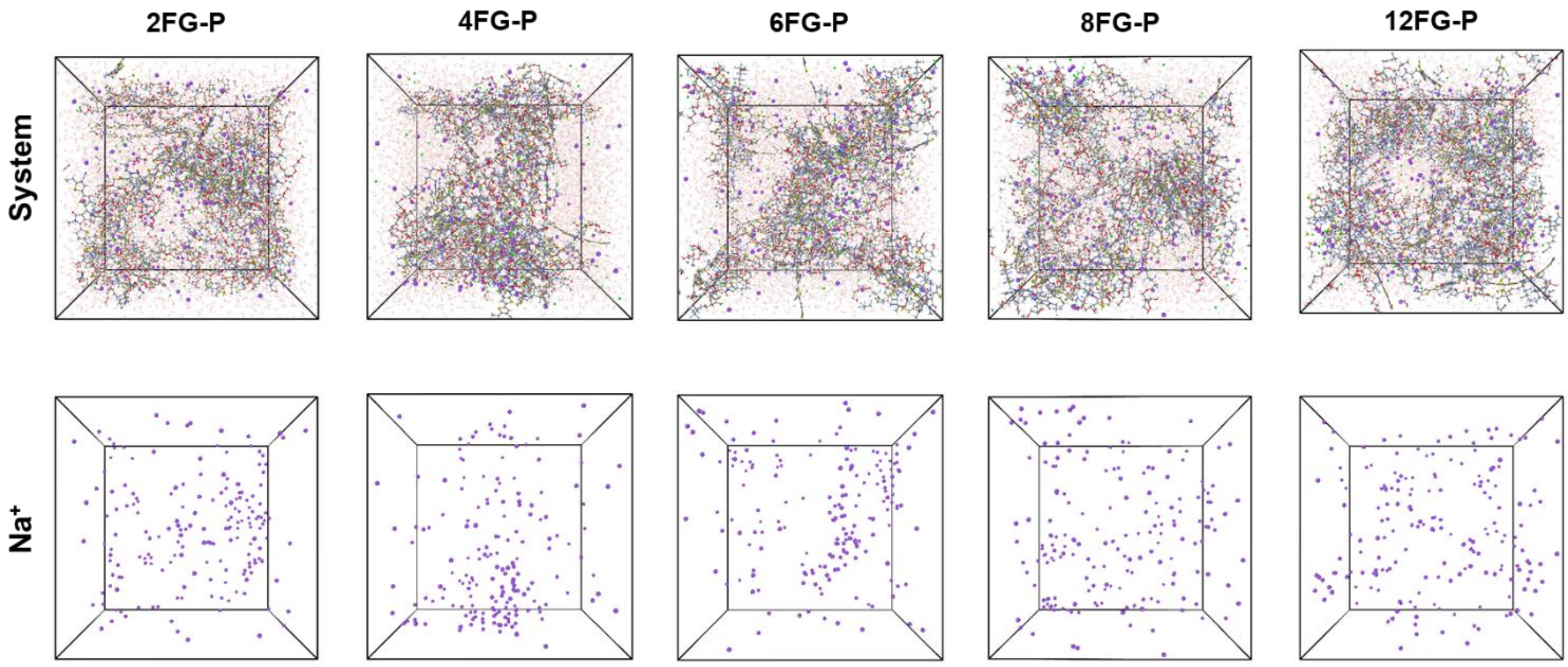

Figure S13. Molecular dynamics (MD) simulation snapshots of 2nFG-P (n = 1, 2, 3, 4, and 6).

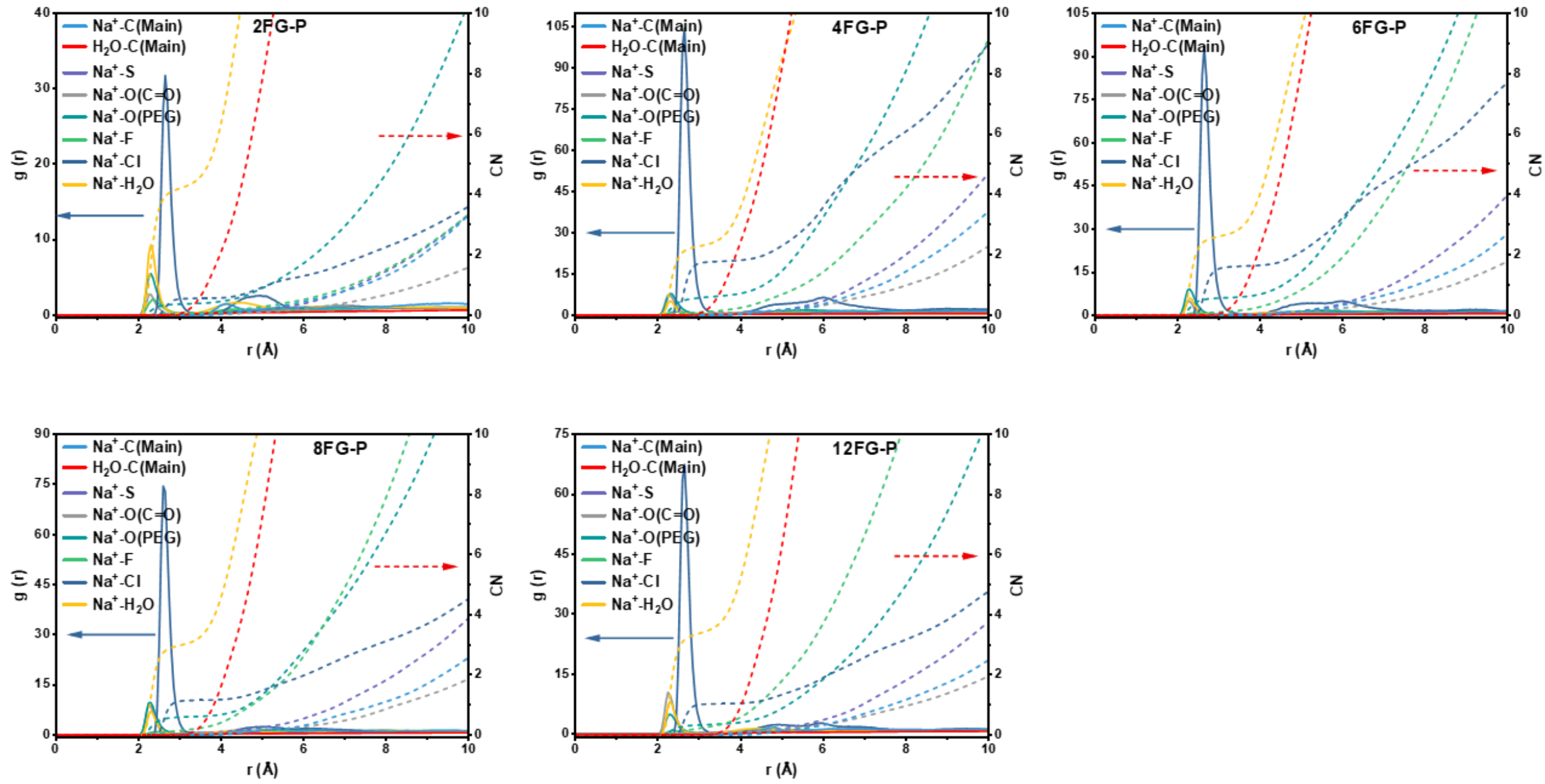

Figure S14. RDFs and the corresponding coordination numbers of 2nFG-P (n = 1, 2, 3, 4, and 6).

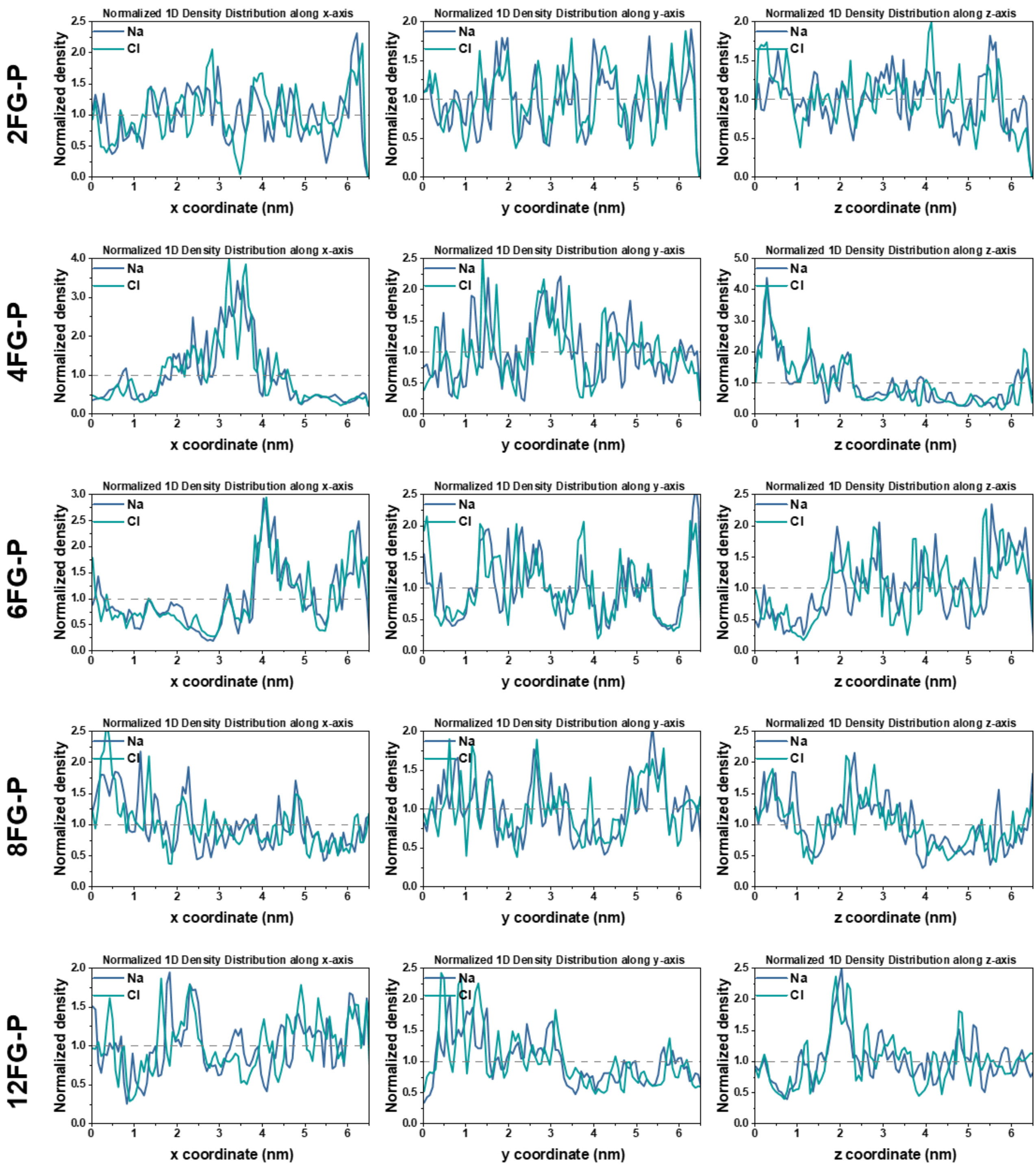

Figure S15. The number density distribution and normalized relative concentration distribution along the x, y, and z directions respectively.

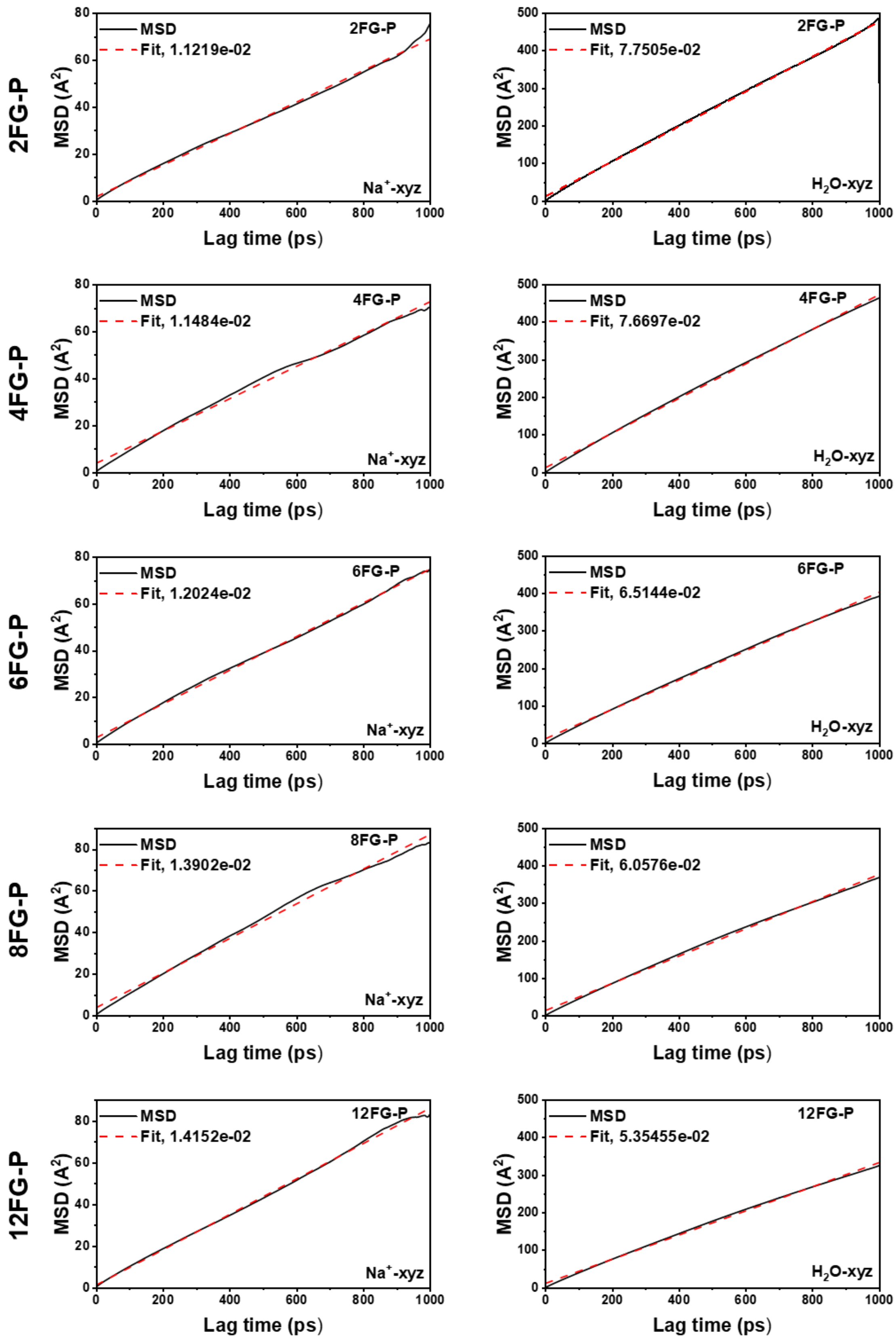

Figure S16. Diffusion coefficient (D) of Na^+ and water molecules in different lengths of fluorinated hydrophobic barrier models.

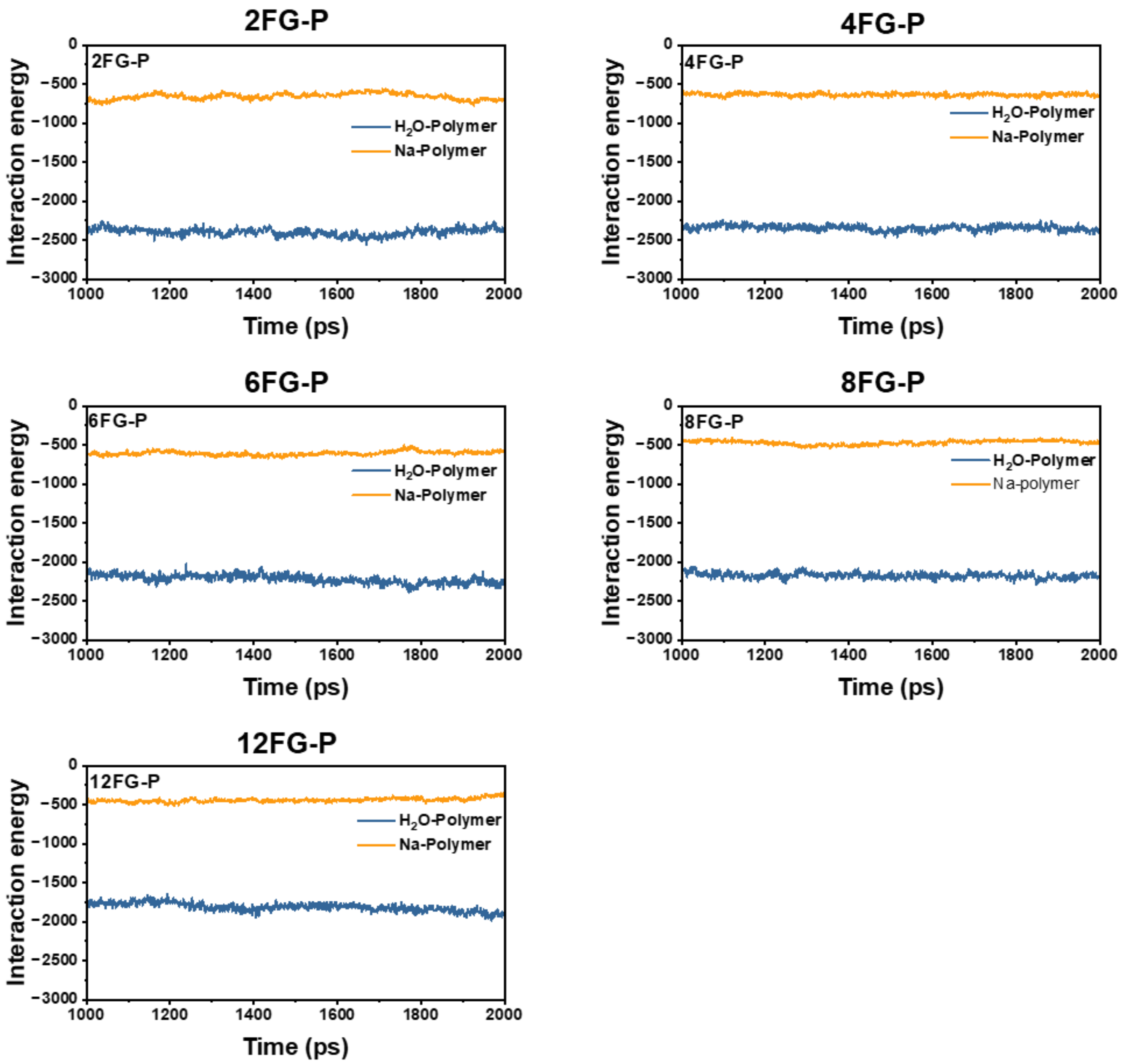

Figure S17. Interaction energy of Na^+ and water molecules with the polymer.

11.The Film Morphology and Microstructure

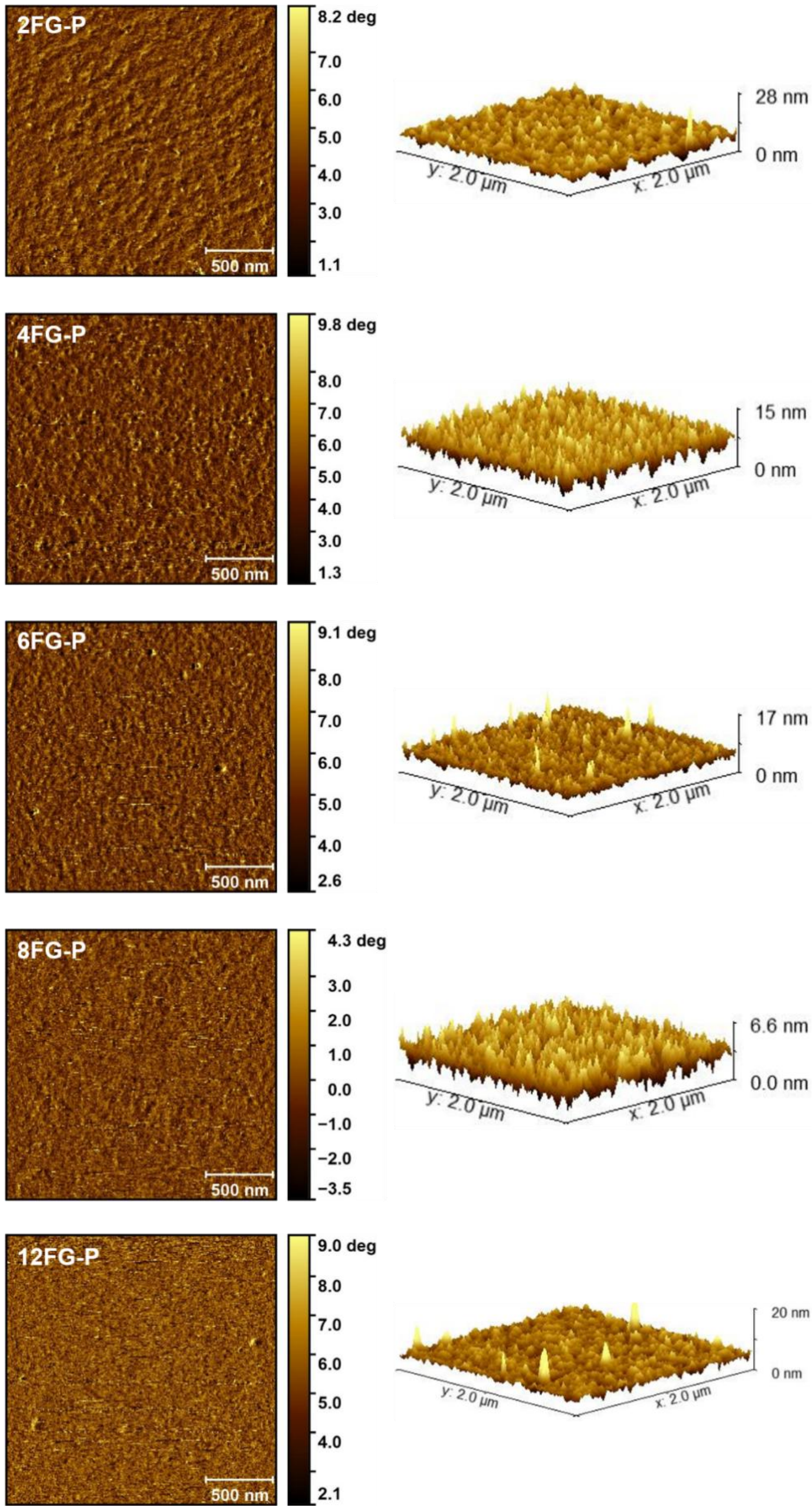

Figure S18. Phase and 3D height-mode AFM images (2 μm ×2 μm).

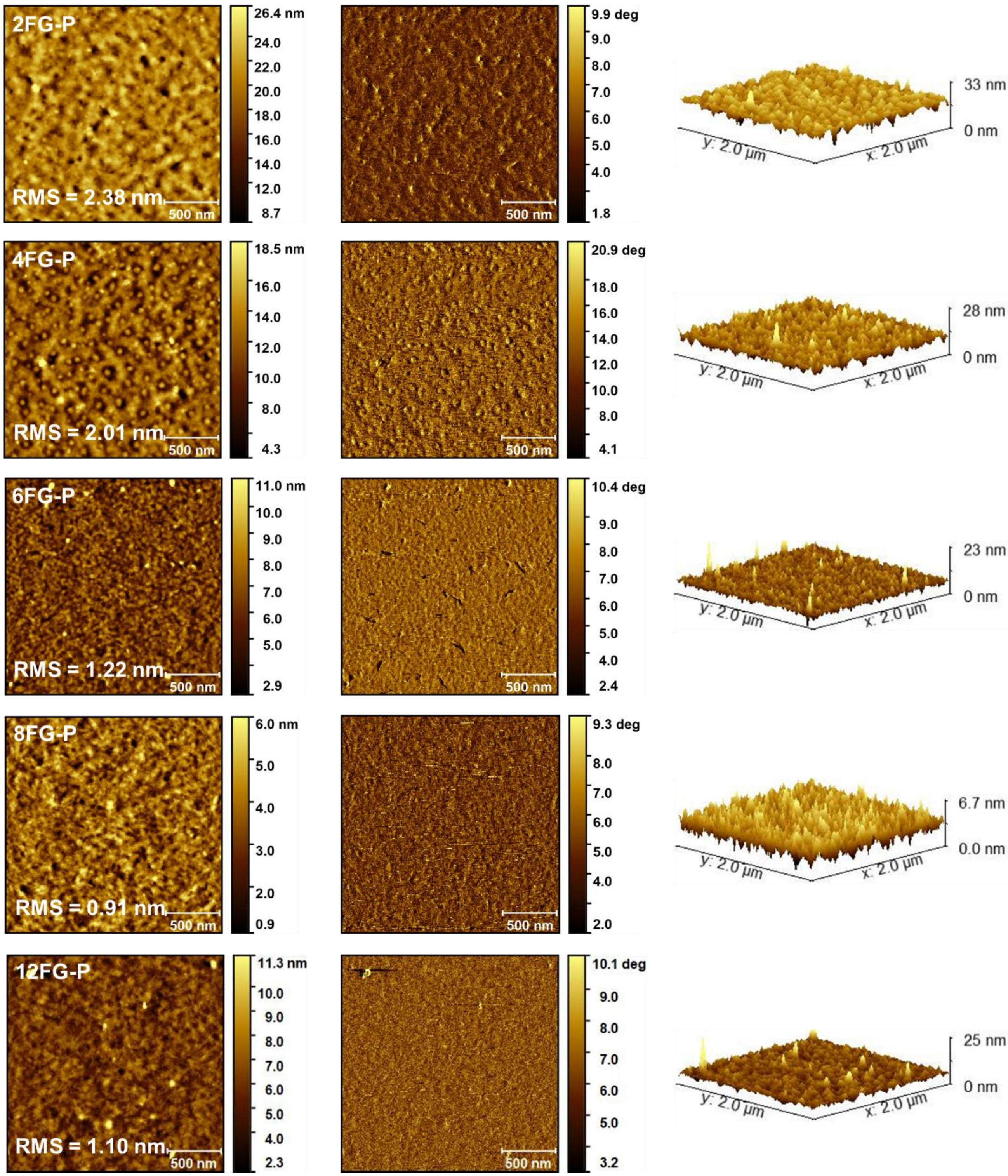

Figure S19. AFM height images, phase, and 3D-images after electrochemical doping.

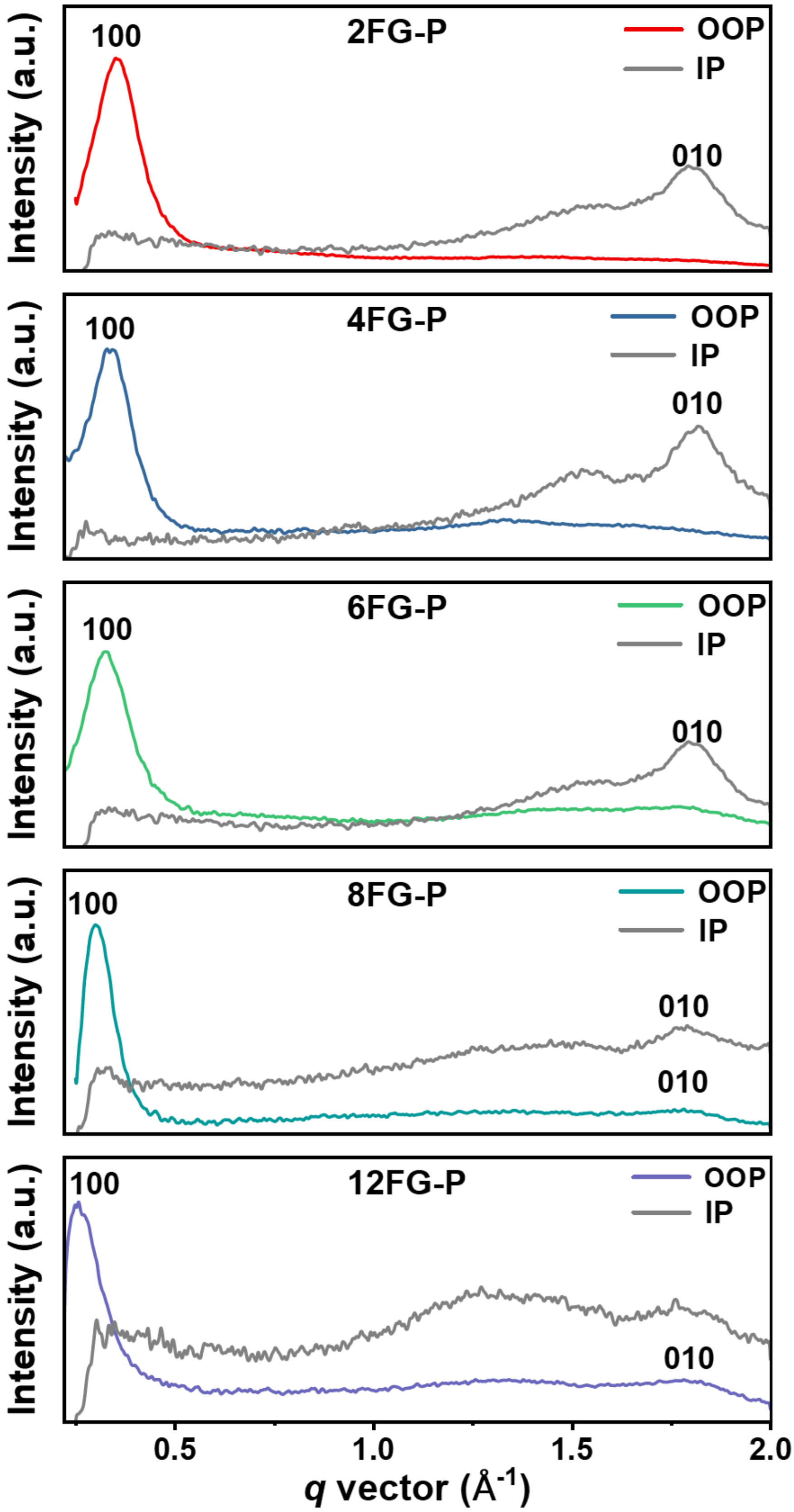

Figure S20. Out-of-plane (OOP) and in-plane (IP) linecut profiles of GIWAXS patterns.

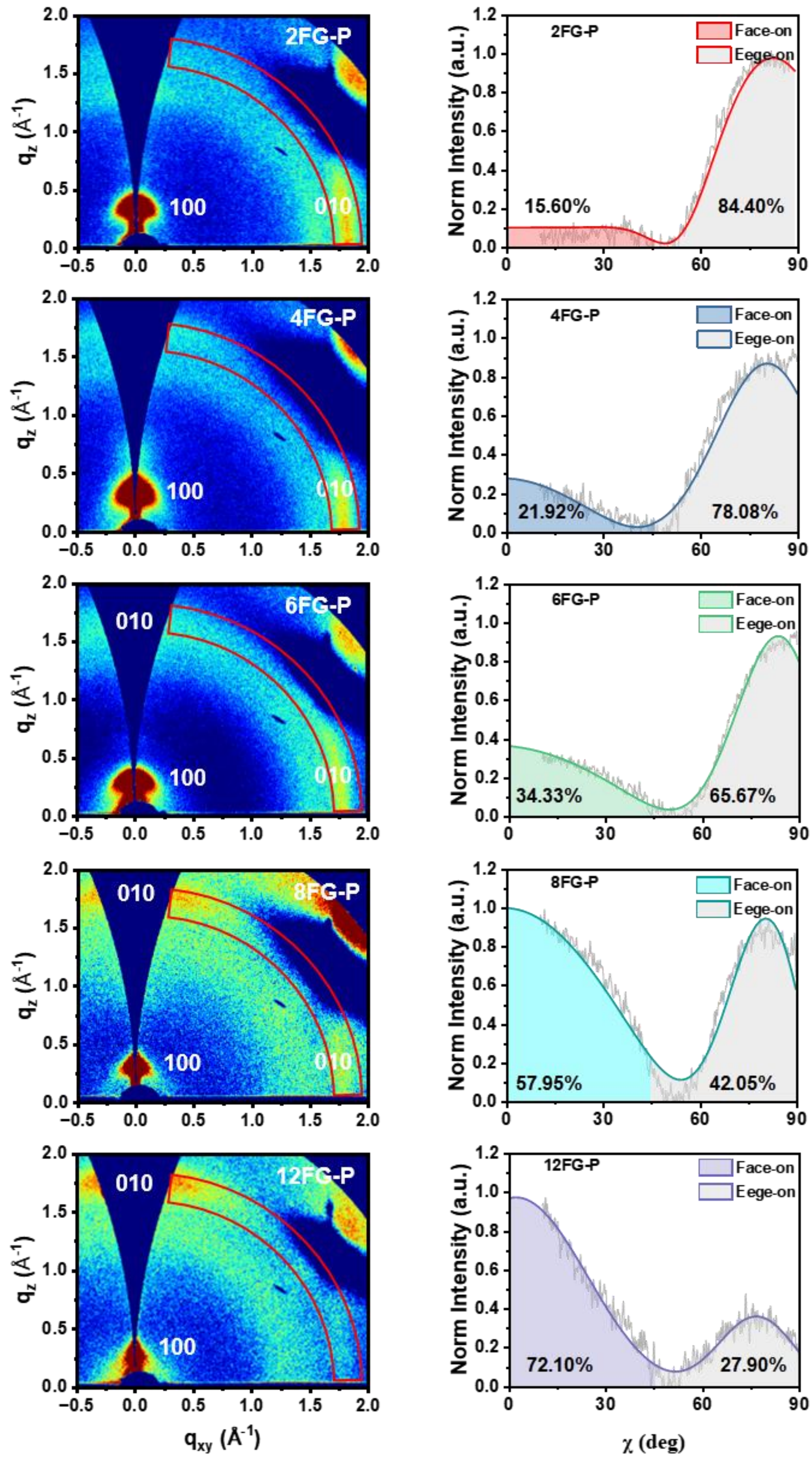

Figure S21. The pole figure of the integration along the azimuthal angle of the (010) peak of 2nFG-P (n= 1, 2, 3, 4, and 6).

Table S3 The proportions and average values of face-on and edge-on for the indicated polymers on the 010 crystal planes

Polymer	Integral area			Ratio	
	Face-on	Edeg-on	Total area	Face-on	Edeg-on
2FG-P	4.40	23.80	28.20	15.60%	84.40%
2FG-P	6.60	23.51	30.11	21.92%	78.08%
6FG-P	10.65	20.37	31.02	34.33%	65.67%
8FG-P	35.55	17.16	52.71	57.95%	42.05%
12FG-P	25.71	9.95	35.66	72.10%	27.90%

Table S4 Detailed crystal structure parameter of 2nFG-P (n= 1, 2, 3, 4, and 6).

Polymer	Doping state	Lamellar stacking						π - π stacking					
		q ($Å^{-1}$)	d (100) Å	Δd (Å)	FWHM (Å)	CCL (Å)	ΔCCL (Å)	q ($Å^{-1}$)	d (010) Å	Δd (Å)	FWHM (Å)	CCL (Å)	ΔCCL (Å)
2FG-P	Pristine	0.383	16.405	1.244	0.135	41.422	-5.345	1.798	3.495	-0.084	0.124	45.097	-7.056
	Doped	0.356	17.649		0.155	36.078		1.843	3.410		0.147	38.041	
4FG-P	Pristine	0.352	17.850	1.132	0.122	45.836	-3.472	1.793	3.504	-0.061	0.127	44.032	-4.927
	Doped	0.331	18.982		0.132	42.364		1.825	3.443		0.143	39.105	
6FG-P	Pristine	0.327	19.215	0.924	0.104	53.977	-2.199	1.786	3.518	-0.051	0.140	39.943	-3.865
	Doped	0.312	20.138		0.108	51.778		1.813	3.467		0.155	36.078	
8FG-P	Pristine	0.304	20.668	0.416	0.080	69.639	-1.610	1.780	3.530	-0.039	0.145	38.489	-0.958
	Doped	0.298	21.371		0.082	68.030		1.800	3.491		0.149	37.530	
12FG-P	Pristine	0.261	24.073	0.280	0.098	57.062	-1.141	1.775	3.540	-0.035	0.195	28.677	-0.717
	Doped	0.258	24.640		0.100	55.920		1.793	3.525		0.200	27.960	

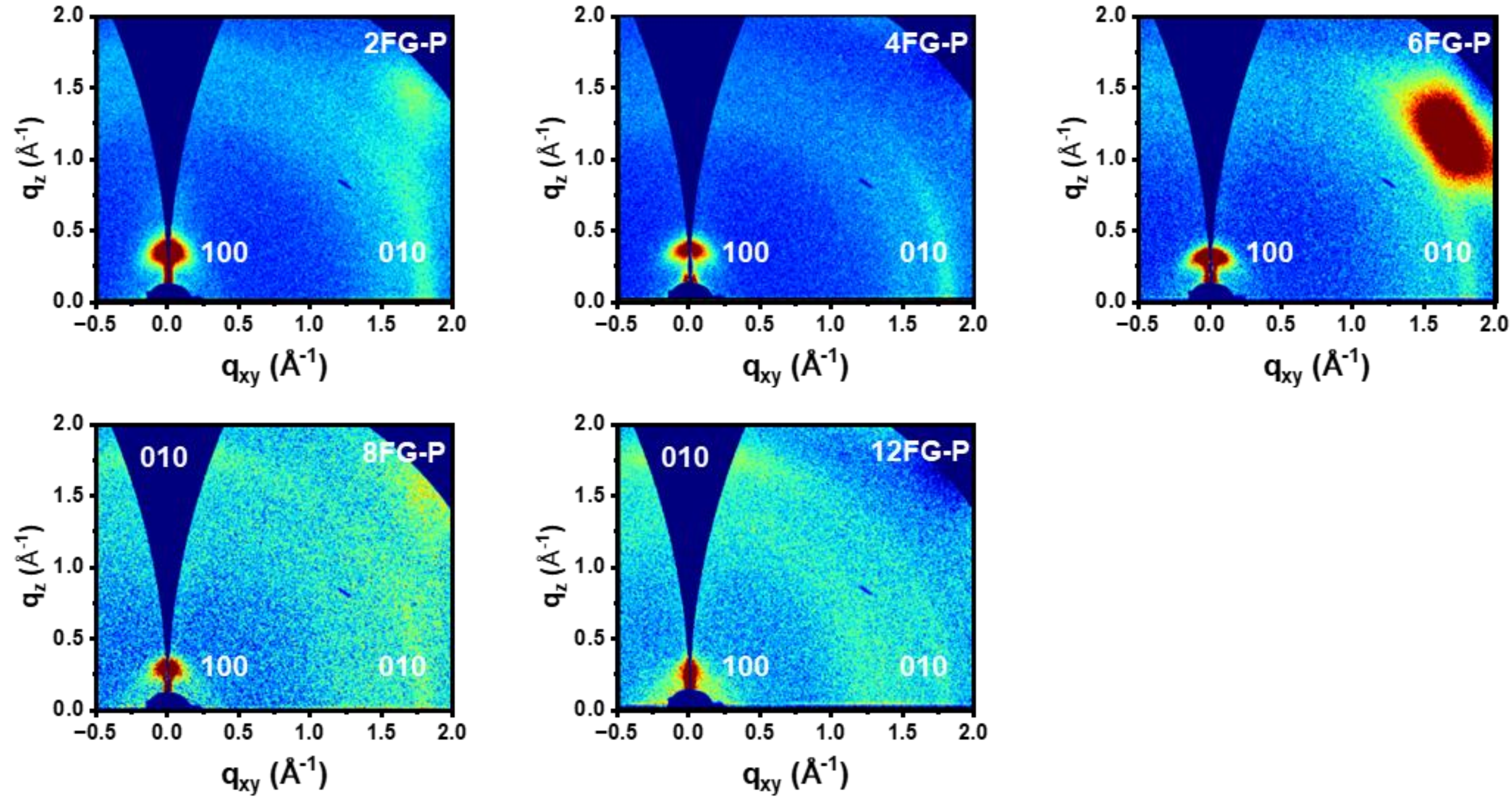

Figure S22. Doped 2D-GIWAXS images of 2nFG-P (n= 1, 2, 3, 4, and 6). "Doped" stands for the films on the silicon substrate (with a 60 nm thick layer of gold) that are reduced by voltage bias for 1 min and blow-dried.

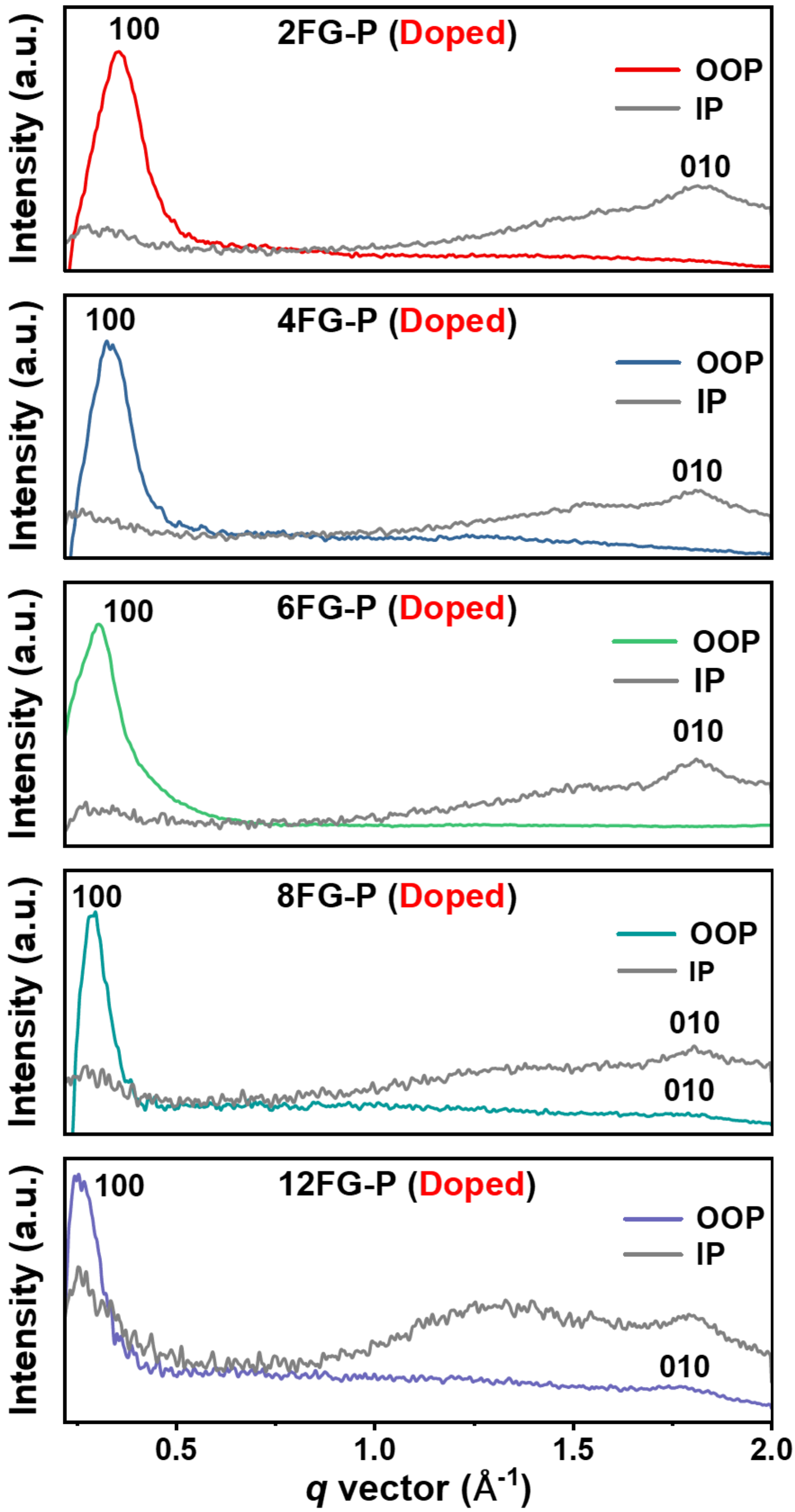

Figure S23. Out-of-plane (OOP) and in-plane (IP) linecut profiles of GIWAXS patterns for the indicated doped polymers.

12.OECTs Fabrication Process

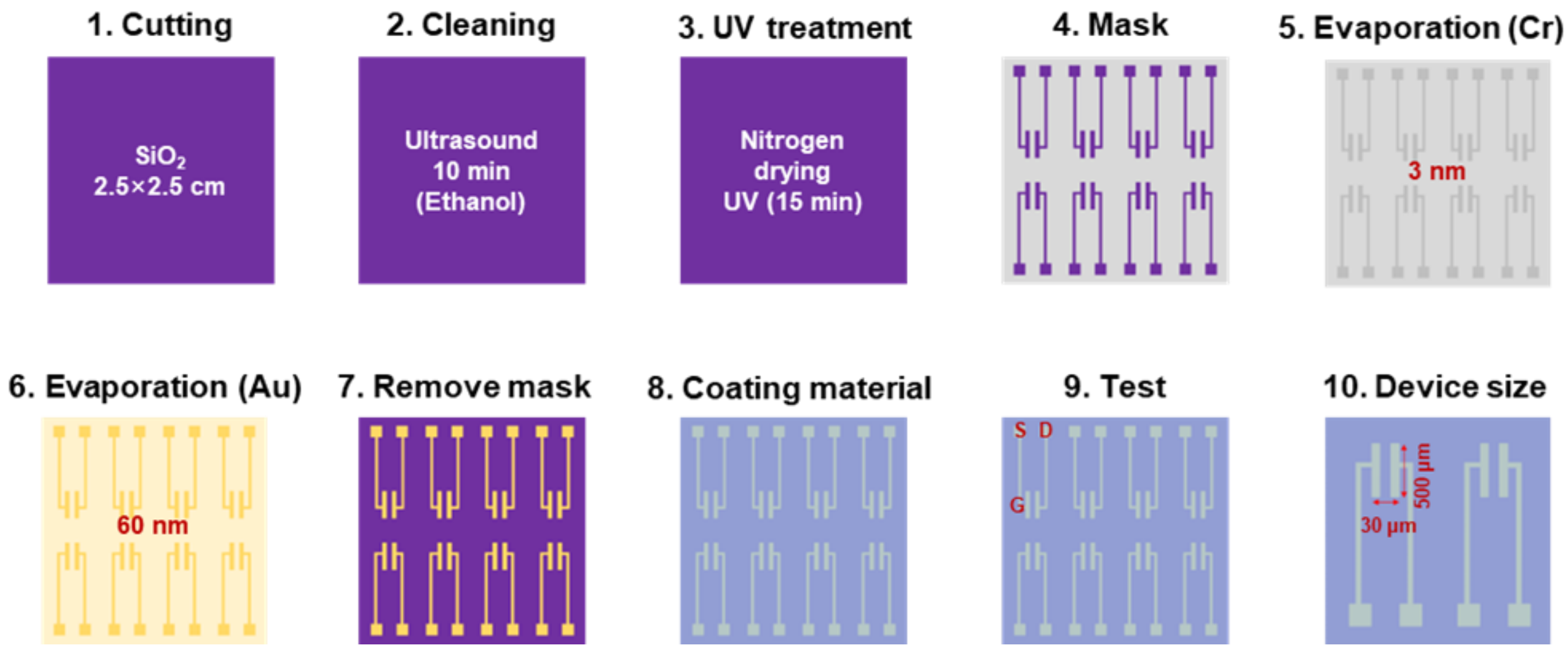

Figure S24. Schematic diagram of the planar OECT fabrication process.

13. Transfer and Transconductance Curves

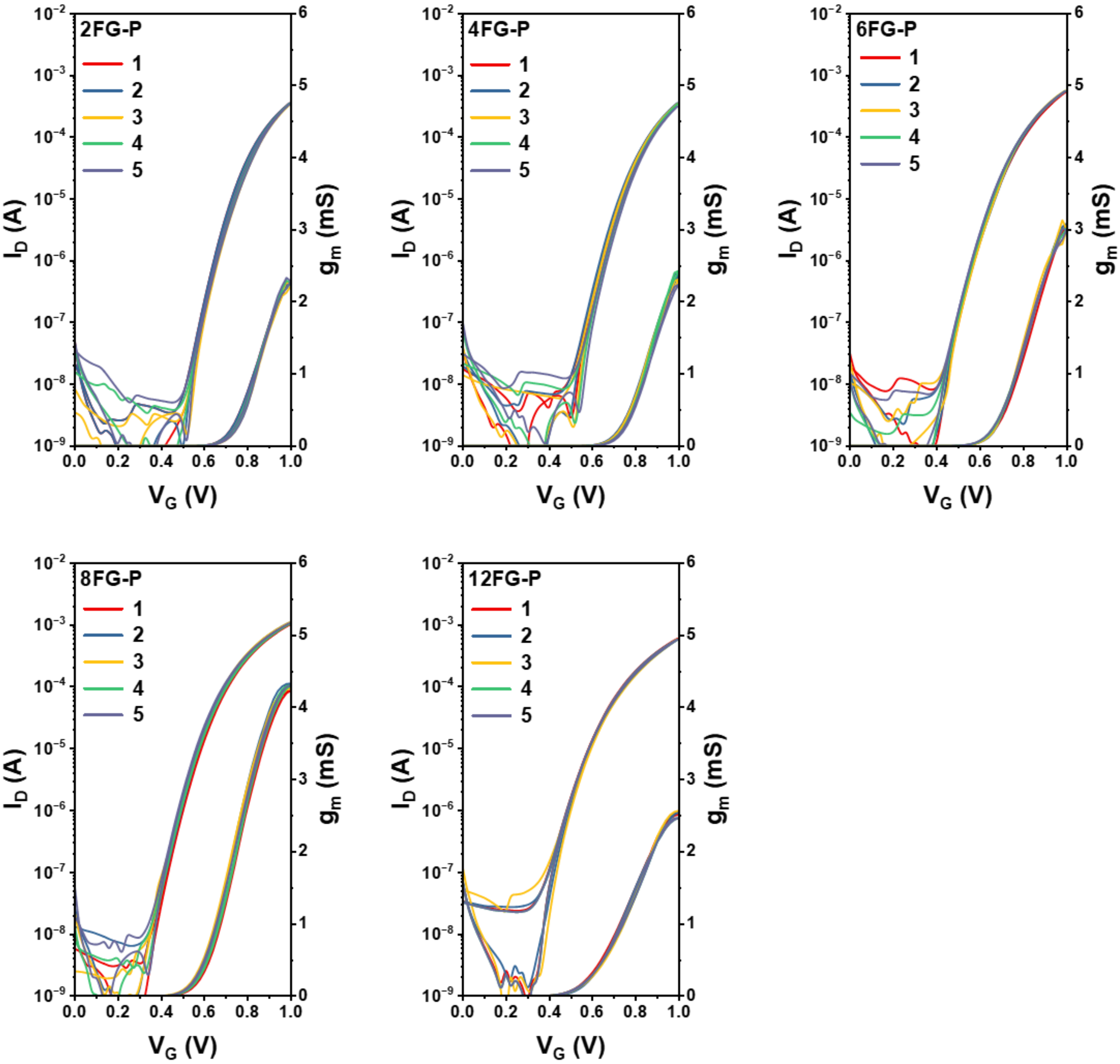

Figure S25. Representative transfer and transconductance plots for five devices.

14.Output Curves

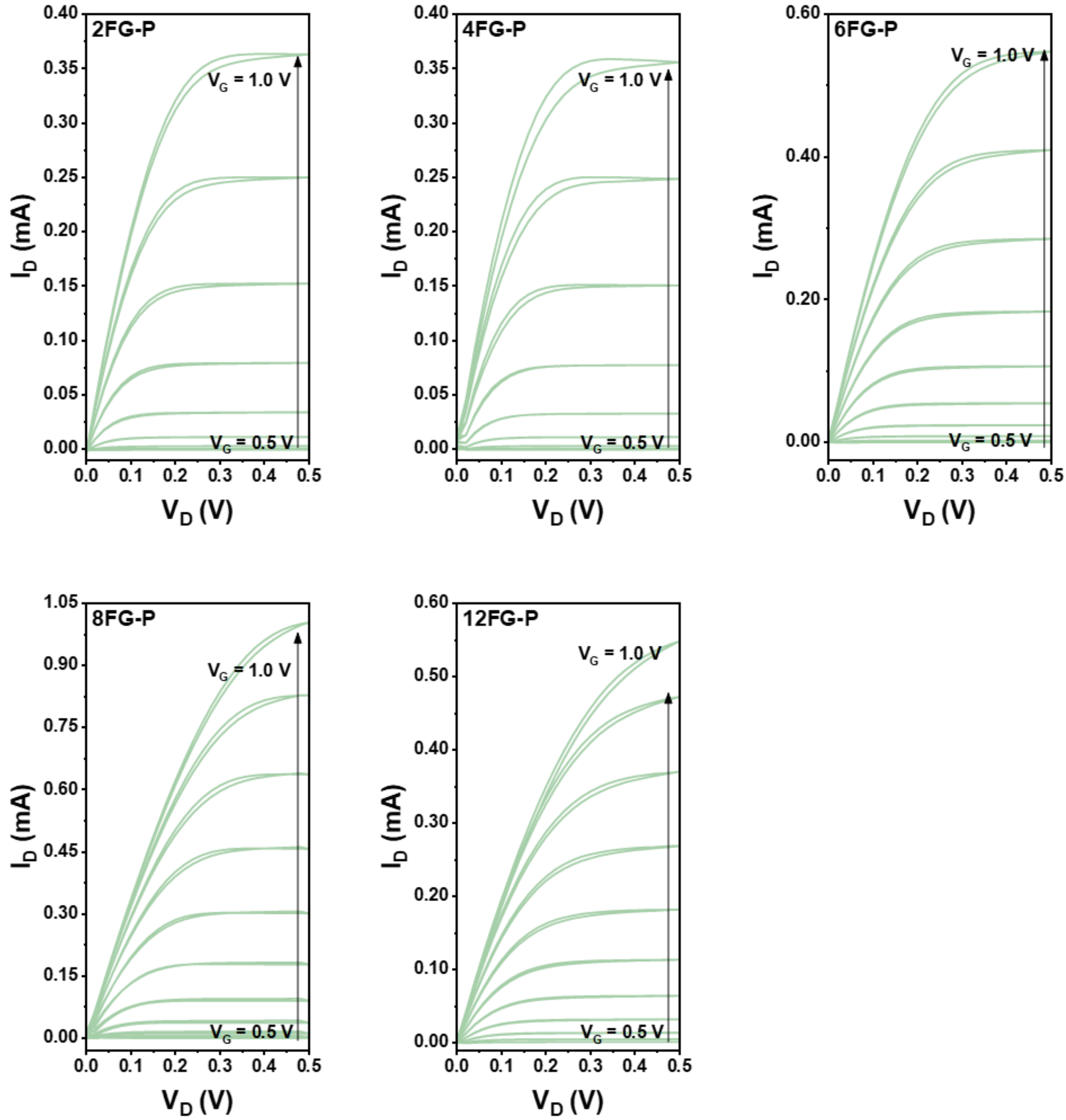

Figure S26. Output curves of 2nFG-P (n = 1, 2, 3, 4, and 6).

15.Electrochemical Impedance Spectroscopy

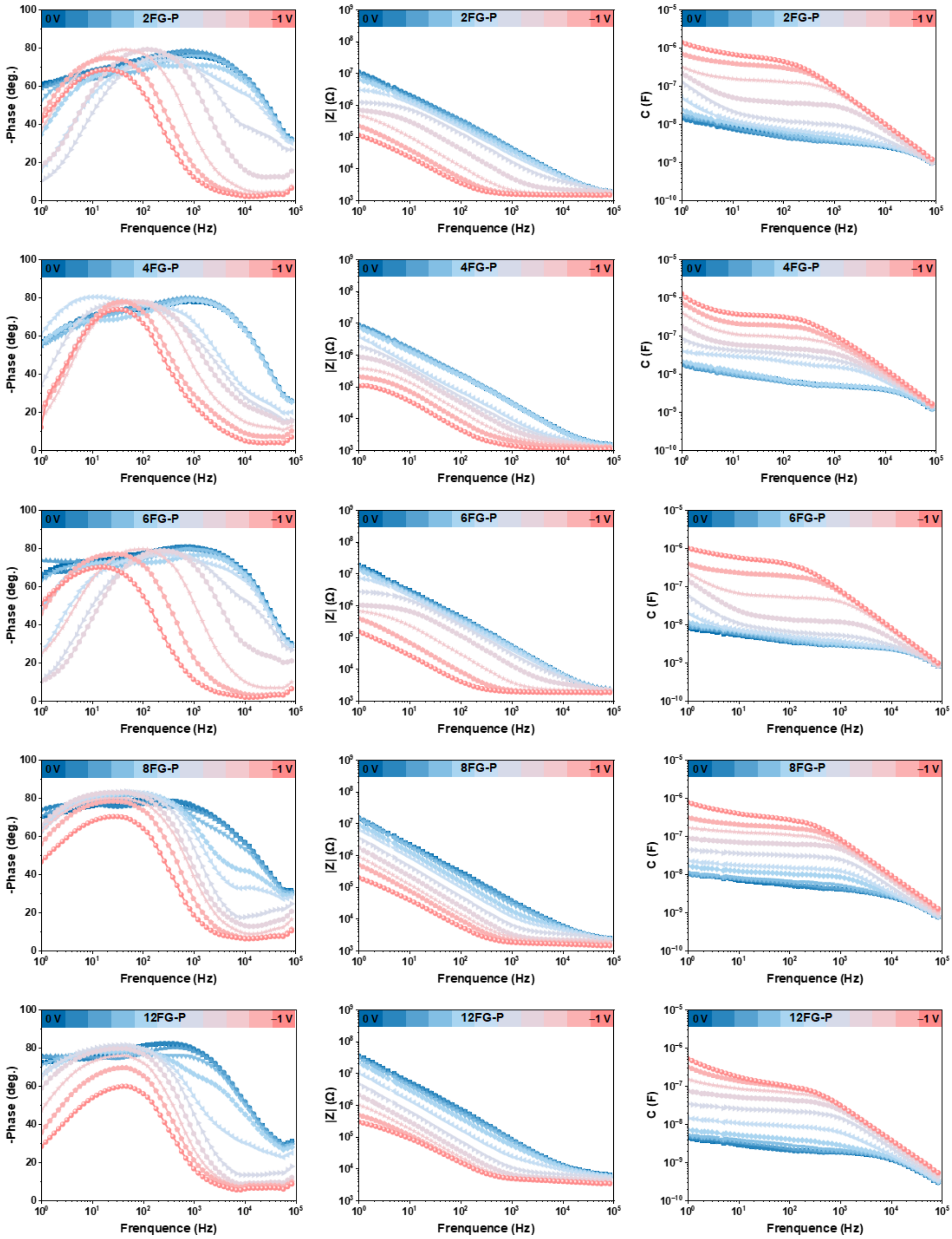

Figure S27. Electrochemical impedance spectroscopy of phase plots, impedance, and capacitance calibration for the indicated polymers.

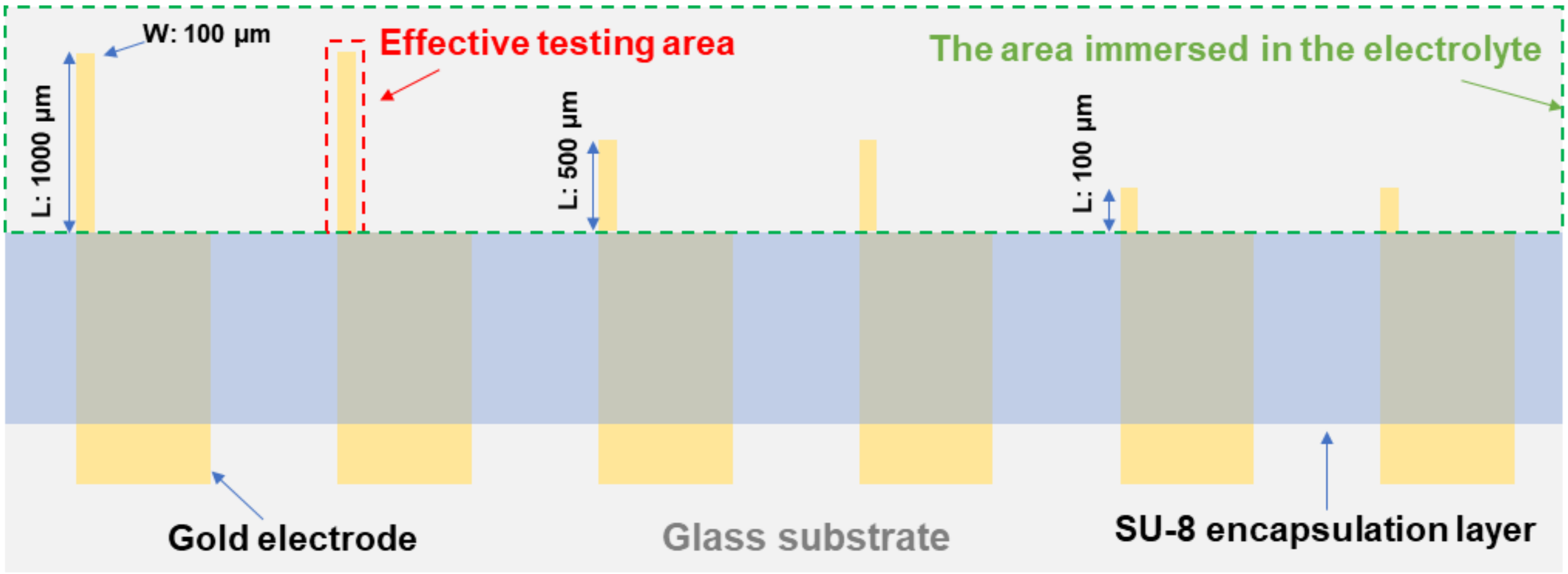

Figure S28. Schematic diagram of the electrode structure used for volume capacitance testing.

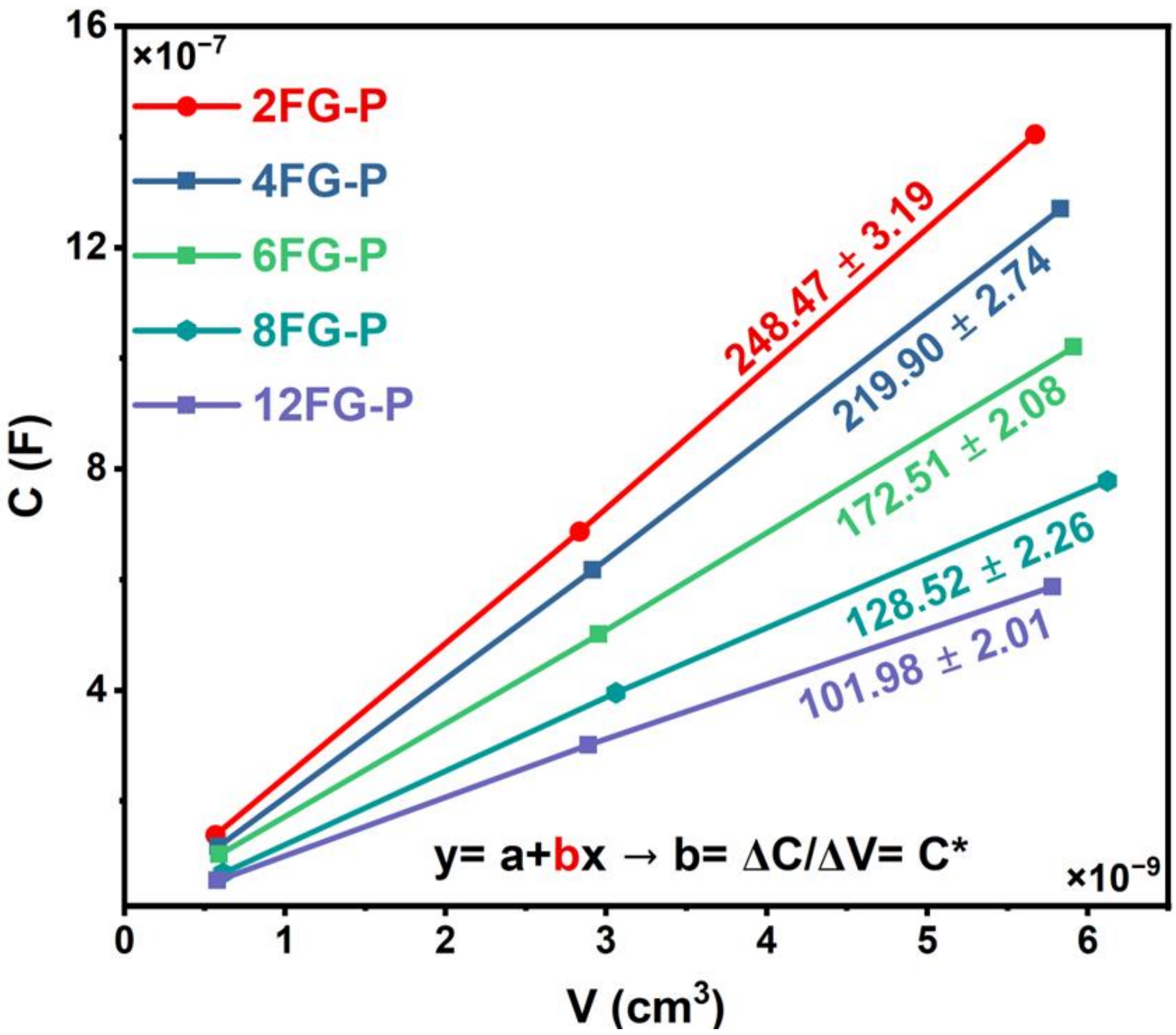

Figure S29. The capacitance obtained from tests with different electrode sizes and the corresponding fitting curves.

16. Threshold Voltage

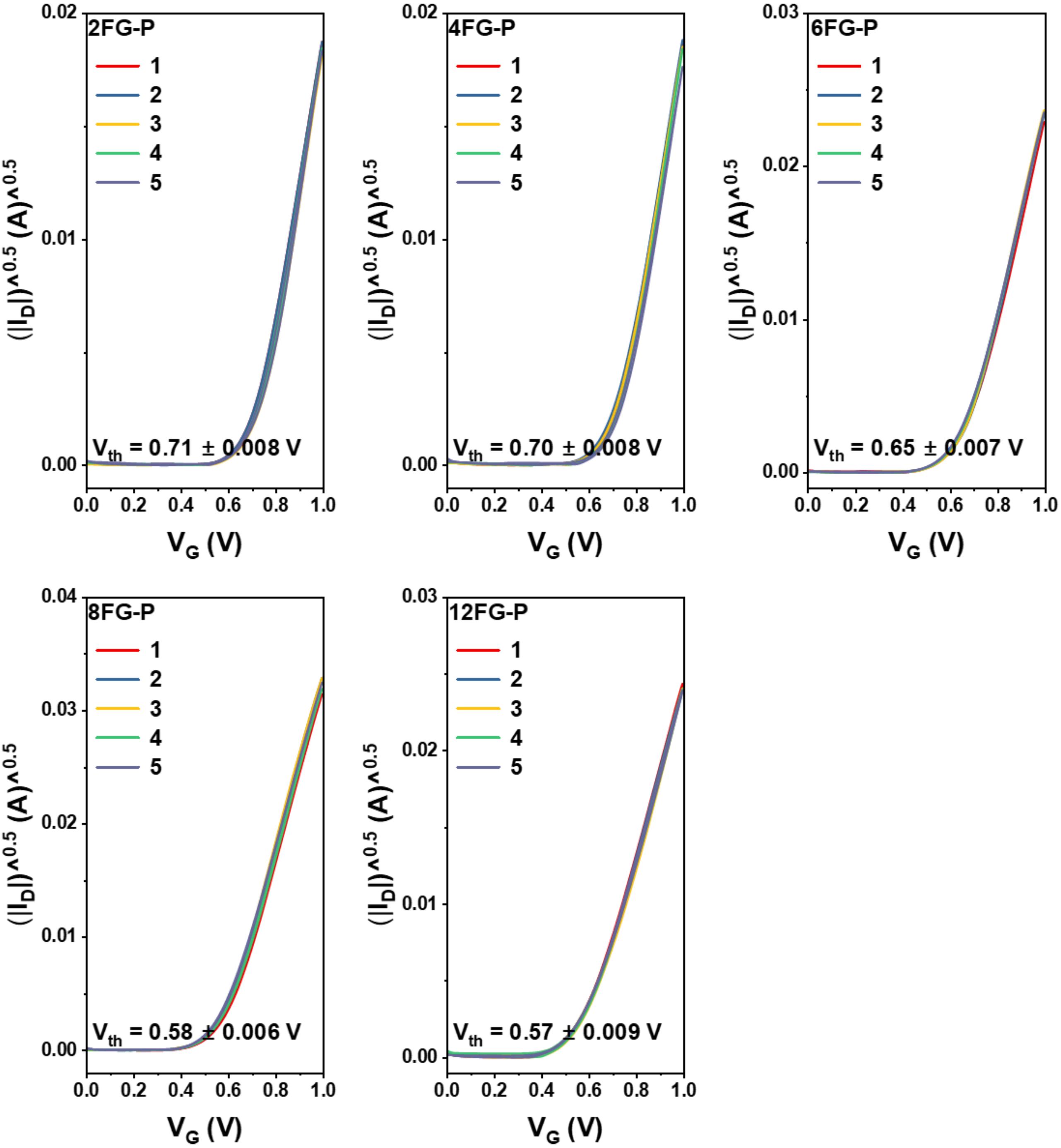

Figure S30. The average threshold voltage (V_{th}) of the indicated OECTs.

17. Transient Profiles

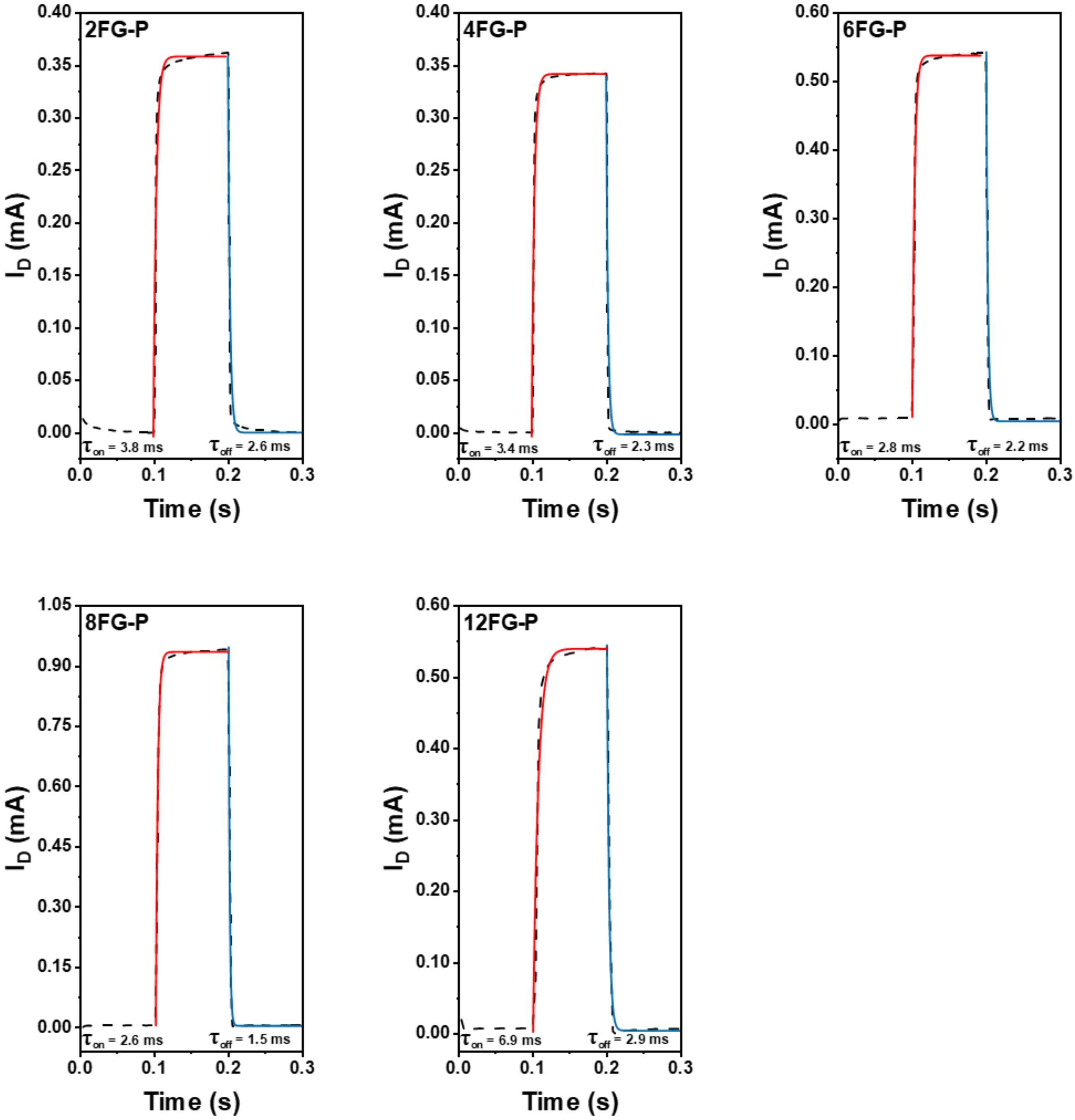

Figure S31. Transient profiles of 2nFG-P (n = 1, 2, 3, 4, and 6) based planar OECT with NaCl (0.1 M) as electrolyte. The response time was obtained from the drain voltage was at 0.5 V, and the gate voltage at 0/1.0 V.

18.Comparison of the n-Type OECTs Device Performance and Stability

Table S5. Comparison of the device performance of the OECTs developed in this work and the other n-type OECTs.

Polymer	$g_{m, norm}$ (S cm^{-1})	μC^* (F cm^{-1} V^{-1} s^{-1})	C^* (F cm^{-3})	μ (cm^2 V^{-1} s^{-1})	V_{th} (V)	ref
**f-BTI2g-TVT**	0.27	1.5	110	0.14	0.90	20
**f-BTI2g-TVTCN**	12.8	41.3	170	0.24	0.68	
**f-BTI2g-TVTF**	22.6	90.2	222	0.41	0.75	21
**f-BTI2g-TVTCl**	3.97	18.9	149	0.13	0.79	
**f-BTI2TEG-FT**	4.6	15.2	52	0.03	0.53	22
**f-BTI2TEG-T**	0.27	2.3	443	0.04	0.68	
**PBTI2g-DTz**	2.44	12.53	72	0.17	0.80	
**PBTI2g-DPP**	4.16	26.39	203	0.13	0.78	23
**PBTI2g-BTI**	13.71	63.79	255	0.25	0.78	
**BDOPV-TVT**	0.59	2.50	7.33	0.34	0.35	
**BDOPV-CNTVT**	0.77	3.10	14.84	0.21	0.15	24
**FBDOPV-CNTVT**	1.58	6.13	33.44	0.18	0.0075	
**f-BTI2g-SVSCN**	48.3	150.9	347.5	0.36	0.70	25
**f-BseI2g-SVSCN**	71.4	191.2	387.2	0.48	0.68	
**PBTI1g-DTCN**	1.51	9.81	283.3	0.04	0.83	
**PBTI2g-DTCN**	60.2	287.8	325.4	0.84	0.76	26
**PBTI3g-DTCN**	16.9	59.2	368.8	0.19	0.72	
**p(C-V)**	1.8	14.89	126.75	0.117	0.17	27
**p(C2F-V)**	25.67	107.56	117.96	0.912	0.02	
**p(C2F-z)**	25.9	84.65	81	1.04	0.07	28
**AIG-BT**	0.029	0.12	83.5	1.4×10^{-3}	0.55	29

Polymer	$g_{m, norm}$ (S cm^{-1})	μC^* (F cm^{-1} V^{-1} s^{-1})	C^* (F cm^{-3})	μ (cm^2 V^{-1} s^{-1})	V_{th} (V)	ref
**gAIID-T**	0.0147	0.09	43.0	2.0×10^{-3}	0.59	30
**gAIID-2FT**	0.94	4.09	99.8	0.049	0.45	
**PgNaN**	0.212	0.662	100	0.652	0.37	31
**PgNgN**	0.007	0.037	239	0.046	0.21	
**AIG-ff2T**	0.0637	0.40	96.7	0.004	0.61	32
**P-AQM2I-2FT**	0.0057	0.070	161	4.35×10^{-4}	0.50	
**P-AQM2I-TT**	0.0124	0.058	216	2.69×10^{-4}	0.42	33
**P-AQM2I-2T**	0.0604	0.45	216	2.08×10^{-3}	0.52	
**P(gPzDPP-2T)**	0.48	0.22	134	1.6×10^{-3}	0.56	34
**P(gPzDPP-CT2)**	0.82	1.72	91	1.9×10^{-2}	0.32	
**P(DPP-TDP)**	1.31	7.62	68.58	0.11	0.575	35
**P(gTDPP2FT)**	15.3	54.8	156	0.35	0.64	36
**gTzDPP-C6**	1.51	4.69	135	0.035	0.41	
**gTzDPP-C8**	6.03	22.9	357	0.064	0.51	37
**gTzDPP-C12**	4.21	13.5	241	0.056	0.53	
**PANDA**	14.7	39.6	181	0.22	0.44	5
**PANDA2**	19.2	51.4	197	0.26	0.47	
**Pg3Tz-2-DPP**	4.8	14.2	243.6	0.058	0.31	
**Pg3Tz-5-DPP**	8.6	22.4	246.5	0.091	0.35	
**Pg4Tz-5-DPP**	18.6	47.2	253.2	0.186	0.30	38
**Pg5Tz-5-DPP**	30.8	92.5	243.3	0.380	0.31	
**Pg6Tz-5-DPP**	7.5	23.6	246.2	0.096	0.29	
**Pg7Tz-5-DPP**	4.9	16.6	252.8	0.066	0.30	
**p(gNDI-gT2)**	0.11	0.0397	397	1.0×10^{-5}	0.28	39
**p(C3-gNDI-gT2)**	0.34	0.13	72	9.2×10^{-4}	0.25	40

Polymer	$g_{m, norm}$ (S cm^{-1})	μC^* (F cm^{-1} V^{-1} s^{-1})	C^* (F cm^{-3})	μ (cm^2 V^{-1} s^{-1})	V_{th} (V)	ref
**p(C6-gNDI-gT2)**	0.37	0.16	59	6.3×10^{-4}	0.37	
**p(C$_2$-T2)**	0.40	0.20	492	3.97×10^{-4}	0.27	
**p(C$_4$-T2)**	0.63	0.30	158	1.90×10^{-3}	0.24	
**p(C$_6$-T2)**	2.28	1.29	272	4.74×10^{-3}	0.30	41
**p(C$_8$-T2)**	0.15	0.13	342	3.76×10^{-4}	0.37	
**p(C$_4$-T2-C$_2$-EG)**	0.02	0.01	200	4.99×10^{-5}	0.30	
**p(C$_4$-T2-C$_4$-EG)**	0.01	0.006	116	5.34×10^{-5}	0.33	
**P(NDITEG-T)**	0.031	0.04	181.7	0.24×10^{-3}	0.26	
**P(NDIDTEG-T)**	0.135	0.21	232.7	0.94×10^{-3}	0.18	42
**P(NDIDEG-T)**	0.359	0.51	240.1	2.12×10^{-3}	0.15	
**P(NDIMTEG-T)**	0.378	0.56	250.8	2.23×10^{-3}	0.18	
**gNDI-EDBT**	0.003	0.02	50.5	3.96×10^{-4}	0.52	
**gNDI-BT**	0.019	0.09	158.3	5.69×10^{-4}	0.30	43
**gNDI-FBT**	0.036	0.12	196.6	6.10×10^{-4}	0.19	
***o*-CNgTVT-2FT**	17.4	100	193.3	0.517	0.79	44
***i*-CNgTVT-2FT**	2.2	10.2	214.2	0.048	0.78	
**CNg4T2-CNT2**	6.75	27.01	309.03	0.087	0.75	45
**P(PyV)**	54.08	120	485	0.25	0.55	46
**NDI-T2-L**	0.05	0.28	309	0.11×10^{-3}	0.16	
**NDI-T2-B**	0.01	0.06	291	0.13×10^{-3}	0.19	
**NDI-T-L**	0.34	1.61	289	4.66×10^{-3}	0.18	11
**NDI-T-B**	0.03	0.20	275	0.44×10^{-3}	0.18	
**TVTCN-L**	34.08	108.3	147	0.68	0.61	
**TVTCN-B**	35.35	113.2	152	0.63	0.60	
**p(gDPP-V-B05)**	68.5	360	291	1.24	0.75	

Polymer	$g_{m, norm}$ (S cm^{-1})	μC^* (F cm^{-1} V^{-1} s^{-1})	C^* (F cm^{-3})	μ (cm^2 V^{-1} s^{-1})	V_{th} (V)	ref
**p(gDPP-V-B20)**	54.8	293	275	1.07	0.80	[47]
**paNL**	12.7	46.9	115	0.41	0.39	[48]
**peNL**	28.4	136.2	227	0.60	0.21	
**gDPP-Tb$_0$**	10.4	39.7	169	0.24	0.74	[49]
**gDPP-Tb$_{0.1}$**	26.9	1188.0	203	0.59	0.77	
**2FG-P**	30.3	104.58	248.47	0.42	0.71	This work
**4FG-P**	33.7	112.42	219.90	0.51	0.70	
**6FG-P**	47.5	135.59	172.51	0.79	0.65	
**8FG-P**	65.8	156.71	128.52	1.22	0.58	
**12FG-P**	37.3	86.80	101.98	0.85	0.57	

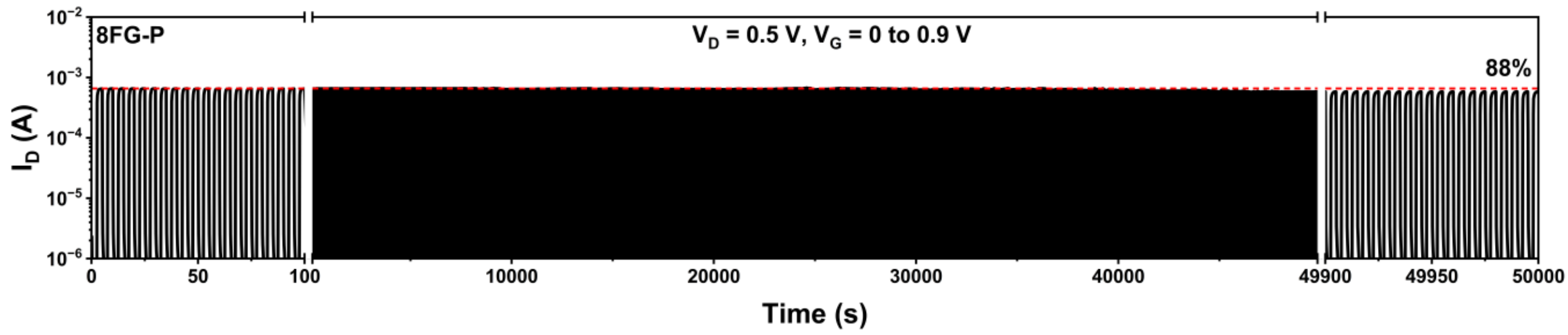

Figure 32. Operational stability of 8FG-P-based OECTs subjected to 10,000 on-off electrochemical cycles (50,000 s) at the V_D and V_G (V_G = 0.9V). The gate switching duration and interval time are both 2.5 s.

Table S6. Comparison of the device stability of the OECTs developed in this work and the other n-type OECTs.

Polymer	Cycles	Runing time (s)	Operational stability (%)	ref
**gTzDPP-C6**	75	900	70-80	
**gTzDPP-C8**	75	900	70-80	37
**gTzDPP-C12**	75	900	70-80	
**P(NDITEG-T)**	100	1000	＞100	
**P(NDIDTEG-T)**	100	1000	＞100	42
**P(NDIDEG-T)**	100	1000	＞100	
**P(NDIMTEG-T)**	100	1000	＞100	
**f-BTI2g-TVTF**	100	1000	91	21
**f-BTI2g-TVTCl**	100	1000	44	
**f-BTI2g-SVSCN**	180	1800	45	25
**f-BseI2g-SVSCN**	180	1800	75	
**p(N-T)**	1800	1800	63	
**p(C-T)**	900	1800	13	50
**P(C-2T)**	900	1800	72	
**AIG-ff2T**	180	1800	55	32
**P(DPP-TDP)**	900	1800	82	35

Polymer	Cycles	Runing time (s)	Operational stability (%)	ref
	900	1800	70.9	
	900	1800	65	
**P4gNDTI**	180	1800	94	51
**o-CNgTVT-2FT**	1000	2000	94	44
**i-CNgTVT-2FT**	1000	2000	48	
**P(gPzDPP-2T)**	100	2000	50	34
**P(gPzDPP-CT2)**	100	2000	50	
**PgNaN**	\	2000	＞75	31
**PBTI1g-DTCN**	200	2000	80	26
**PBTI2g-DTCN**	200	2000	93	
**PBTI3g-DTCN**	200	2000	76	
**BDOPV-CNTVT**	200	2000	87	24
**FBDOPV-CNTVT**	200	2000	85	
**PBTI2g-DTz**	1300	2000	40	23
**PBTI2g-DPP**	1300	2000	65	
**PBTI2g-BTI**	1300	2000	50	
**f-BTI2g-TVT**	240	2400	70	20
**f-BTI2g-TVTCN**	240	2400	75	
**CNg4T2-CNT2**	240	2400	＞80	45
**AIG-BT**	500	2500	＞90	29
**PANDA**	\	2500	89	5
**f-BTI2TEG-FT**	300	3000	70	22
**f-BTI2TEG-T**	300	3000	70	
**p(C-V)**	60	3600	9	27
**p(C2F-V)**	60	3600	77	
**p(C2F-z)**	180	3600	93	28
**gAIID-T**	600	3600	12	30

Polymer	Cycles	Runing time (s)	Operational stability (%)	ref
**P(gTDPP2FT)**	60	3600	54	[36]
**gNDI-EDBT**	360	3600	100	
**gNDI-BT**	360	3600	100	[43]
**gNDI-FBT**	360	3600	100	
**gNDI-V**	600	3600	91	[52]
**gNDI-T**	600	3600	95	
**p(gNDI-gT2)**	720	7200	100	[39]
**gAIID-2FT**	1200	7200	100	[30]
**p(C3-gNDI-gT2)**	900	9000	119	[40]
**p(C6-gNDI-gT2)**	900	9000	100	
**S-DTFMCN**	1042	10416	96	[53]
**B-DTFMCN**	1042	10416	96	
**PNDI2TEG-2Tz**	1000	10000	~100	[54]
**2FG-P**	2000	10000	29	
**4FG-P**	2000	10000	42	
**6FG-P**	2000	10000	70	This work
**8FG-P**	2000	10000	93	
**12FG-P**	2000	10000	97	

19. The OECTs Performance of the Molecular Weight-Matched Samples

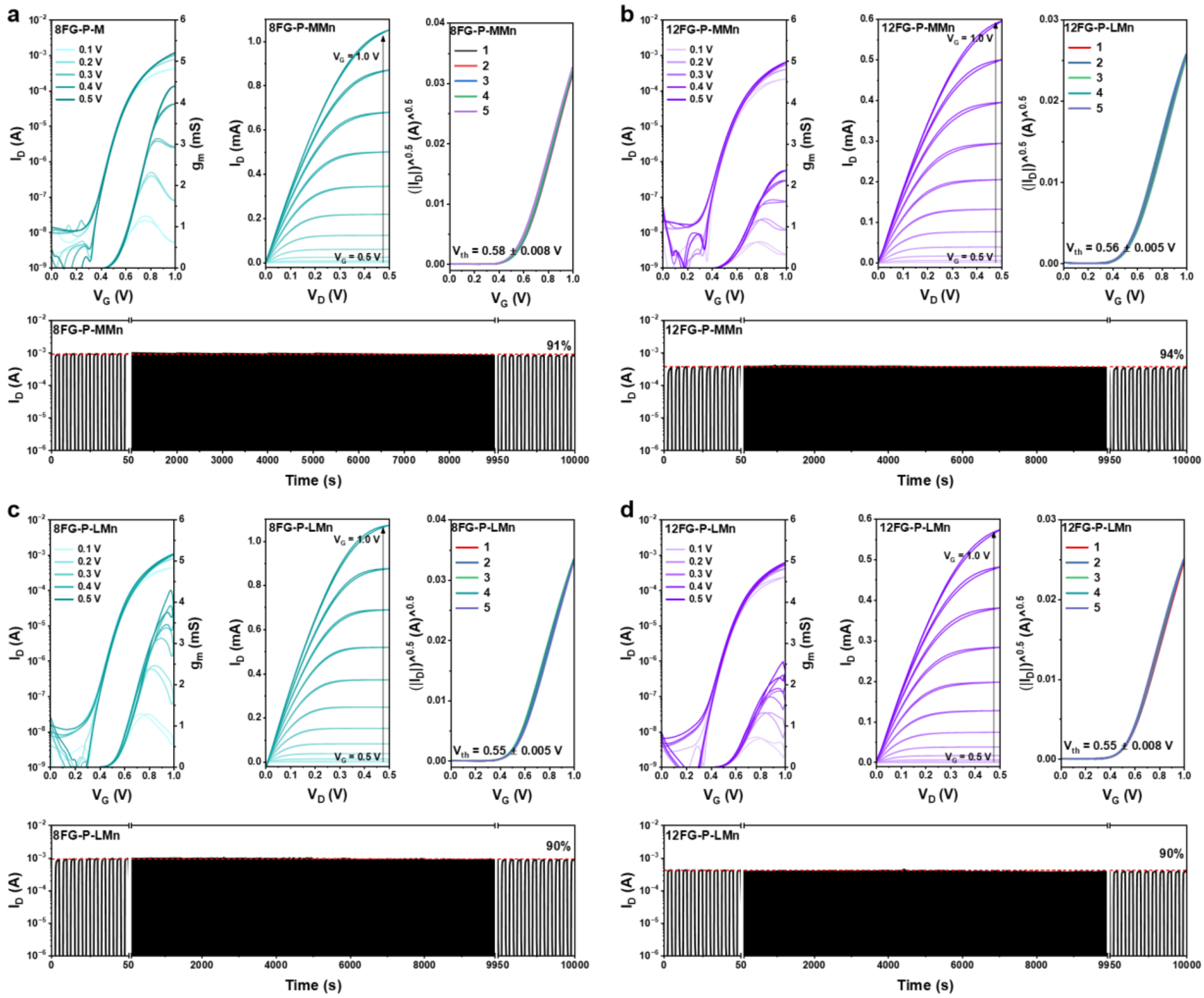

Figure 33. Transfer curves, output curves, threshold voltage (V_{th}), and long-term operational stability of OECTs based on middle- and low-molecular-weight batches of 8FG-P and 12FG-P.

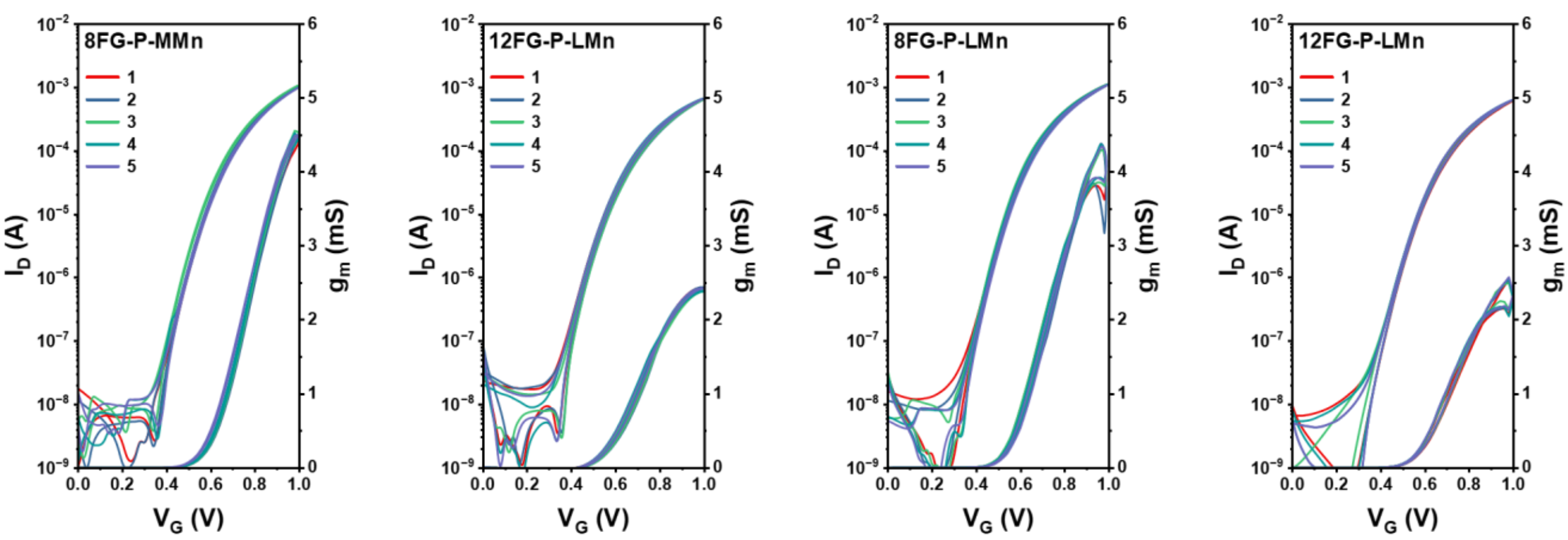

Figure 34. Representative transfer and transconductance curves for OECT devices fabricated from middle- and low-molecular-weight batches of 8FG-P and 12FG-P.

Table S7. Summary of key OECT parameters for 2nFG-P.

Polymer	Mn [kDa]/Đ	d [nm]	g_m [mS]	V_{th} [V]	μC^* [F cm^{-1} V^{-1} s^{-1}]
2FG-P	24.6 / 1.5	44.7 ± 1.1	2.26 ± 0.02	0.71 ± 0.008	104.58 ± 3.98
4FG-P	27.4 / 1.7	41.8 ± 1.8	2.35 ± 0.07	0.70 ± 0.008	112.42 ± 6.61
6FG-P	36.8 / 1.6	38.3 ± 2.0	3.03 ± 0.04	0.65 ± 0.007	135.59 ± 7.79
8FG-P	69.8 / 1.9	39.1 ± 1.4	4.29 ± 0.05	0.58 ± 0.006	156.71 ± 6.31
12FG-P	65.2 / 2.2	40.5 ± 2.3	2.52 ± 0.06	0.57 ± 0.009	86.80 ± 5.65
8FG-P-MMn	39.8 / 1.9	41.5 ± 1.8	4.48 ± 0.05	0.58 ± 0.007	154.19 ± 7.37
12FG-P-MMn	27.2 / 1.5	38.7 ± 1.3	2.42 ± 0.02	0.56 ± 0.005	85.25 ± 3.10
8FG-P-LMn	21.3 / 1.8	39.7 ± 1.3	4.35 ± 0.04	0.55 ± 0.005	152.86 ± 5.47
12FG-P-LMn	15.6 / 1.3	40.4 ± 1.6	2.54 ± 0.03	0.55 ± 0.009	87.71 ± 4.03

20.Molecular Design and Synthesis

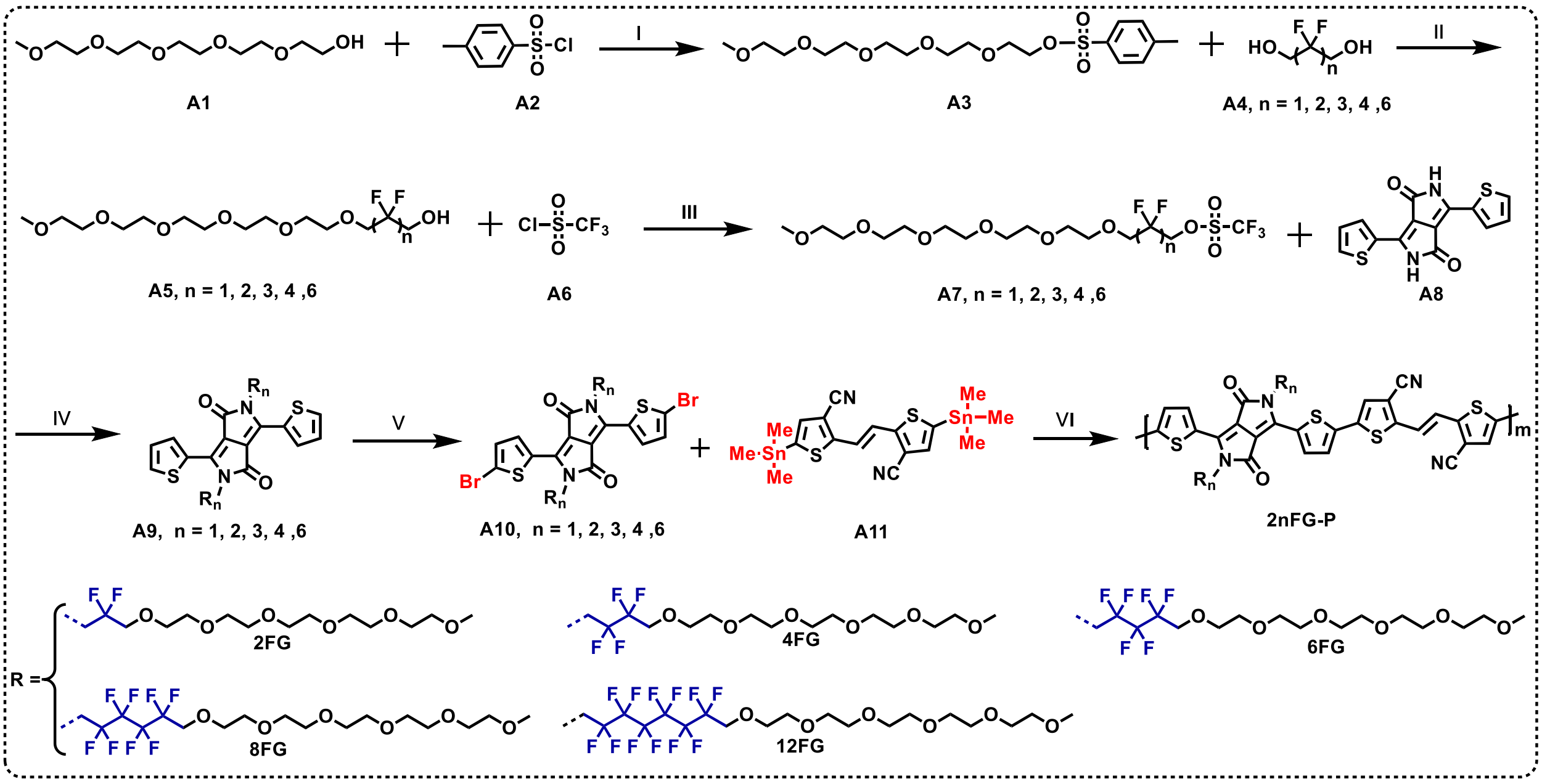

Reagents and conditions: **I:** NaOH, THF, 0 °C to RT; **II:** NaH, Dry 1,4-Dioxane, RT to 90 °C; **III:** Et_3N, THF, 0 °C to RT; **IV:** K_2CO_3, Dry DMF, 85°C; **V:** NBS, $CHCl_3$, 0 °C; **X:** $Pd_2(dba)_3$, $P(o\text{-}Tol)_3$, Dry Chlorobenzene.

Scheme S1. Synthetic routes of 2nFG-P (n = 1, 2, 3, 4, and 6).

(1) Synthesis of 2,5,8,11,14-pentaoxahexadecan-16-yl-4-methylbenzenesulfonate (A3)

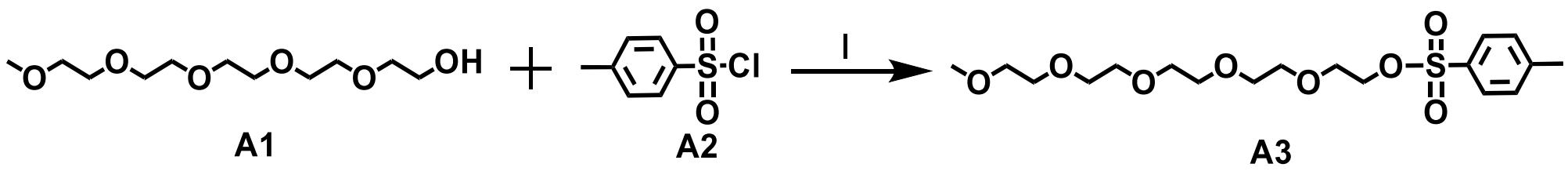

The compound was prepared following the synthetic protocol reported in Ref. [19].

(2) Synthesis of 19,19-difluoro-2,5,8,11,14,17-hexaoxatricosan-20-ol (A5-1).

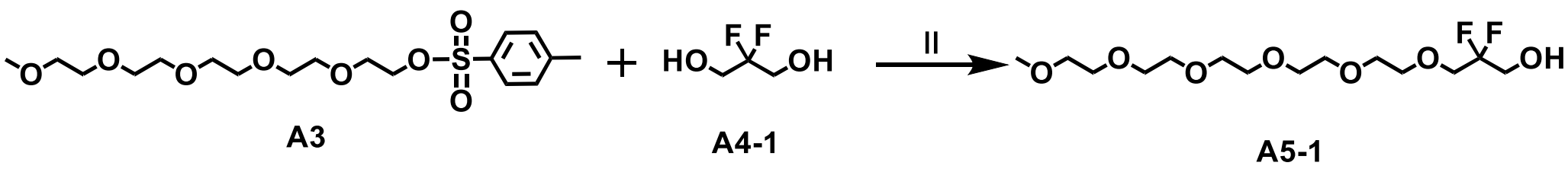

To a 500 mL three-necked round-bottom flask was added 2,2-difluoro-1,3-propanediol (A4-1, 15.00 g, 133.83 mmol, 1.50 eq). Then, 300 mL of anhydrous 1,4-dioxane was added, and the system was degassed by three vacuum-nitrogen purge cycles to remove air completely. 2,5,8,11,14-pentaoxahexadecan-16-yl-4-methylbenzenesulfonate (A3, 36.27 g, 89.22 mmol, 1.00 eq) was dissolved in 50 mL of anhydrous 1,4-dioxane, and the solution was transferred to a pressure-equalizing

dropping funnel. The dropping funnel was connected to the three-necked flask and sealed with a rubber stopper. Under a nitrogen atmosphere, 60% NaH powder (4.64 g, 115.99 mmol, 1.30 eq) was added to the three-necked flask in portions. The system was degassed again by three vacuum-nitrogen purge cycles, stirred at room temperature for 30 min, then heated to 90 °C and stirred at constant temperature for 3 h. The above sulfonate solution was added dropwise slowly via the pressure-equalizing dropping funnel. After the addition was complete, the reaction mixture was stirred overnight, and the reaction progress was monitored by thin-layer chromatography (TLC). After completion of the reaction, the reaction mixture was allowed to cool to room temperature naturally. Then, 12 mL of 2 mol/L HCl-ether solution was added slowly to quench the reaction, followed by stirring for 15 min. The solvent was removed by reduced pressure distillation, and the residue was redissolved in 250 mL of dichloromethane. The resulting white solid was removed by suction filtration, and the organic phase was collected and concentrated by reduced pressure distillation. Purification by column chromatography using ethyl acetate:petroleum ether = 2:1 (v/v) as the eluent afforded 12.67 g of a colorless transparent oil with a yield of 41.00%. ^{1}H NMR (400 MHz, $CDCl_3$) δ (ppm) = 3.82 (t1, J = 13.2 Hz, 2H), 3.80 (t2, J = 12.2 Hz, 2H), 3.73 – 3.69 (m, 2H), 3.66 – 3.60 (m, 16H), 3.54 – 3.51 (m, 2H), 3.39 (s, 1H), 3.35 (s, 3H).^{19}F NMR (376 MHz, $CDCl_3$) δ (ppm) = −114.36 (s, 2F). ^{13}C NMR (101 MHz, $CDCl_3$) δ (ppm) = 118.94 – 113.07 (m), 71.91, 71.60, 70.59, 70.57, 70.55, 70.52, 70.48, 70.15, 68.37, 68.09, 67.80, 60.83, 60.55, 60.28, 59.01. HR-ESI-MS (m/z): calculated: $C_{14}H_{28}F_2O_7$, $[M+H]^+$:347.1876; found: 347.1879.

(3) Synthesis of 19,19,20,20-tetrafluoro-2,5,8,11,14,17-hexaoxatricosan-21-ol (A5-2).

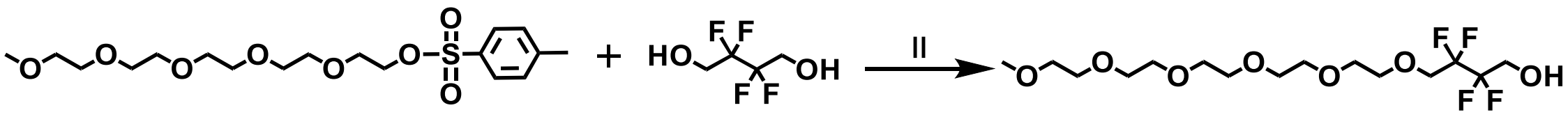

A3 A4-2 A5-2

Fluorine-substituted diol for the synthesis of compound A4-1 was replaced with 2,2,3,3-tetrafluoro-1,4-butanediol (A4-2) using the same molar stoichiometry. The rest of synthetic procedure was same as the compound 5a-1 (15.96g, 69.15% yield). ^{1}H NMR (400 MHz, $CDCl_3$) δ (ppm) = 3.95 (t1, J = 13.6 Hz, 2H), 3.95 (t1, J = 12.8 Hz, 2H), 3.77 (s, 1H), 3.74 – 3.70 (m, 2H), 3.67 – 3.55 (m, 16H), 3.51 (m, 2H), 3.33 (s, 3H). ^{19}F NMR (377 MHz, $CDCl_3$) δ (ppm) = −122.57 (t, J = 3.8Hz, 2F), −124.50 (t, J = 3.8Hz, 2F). ^{13}C NMR (101 MHz, $CDCl_3$) δ (ppm) = 118.94 – 113.07 (m), 71.91, 71.60, 70.59,

70.57, 70.55, 70.52, 70.48, 70.15, 68.37, 68.09, 67.80, 60.83, 60.55, 60.28, 59.01. HR-ESI-MS (m/z): calculated: $C_{15}H_{28}F_4O_7$, $[M+H]^+$: 397.1844; found: 397.1840.

(4) Synthesis of 19,19,20,20,21,21-hexafluoro-2,5,8,11,14,17-hexaoxatricosan-22-ol (A5-3).

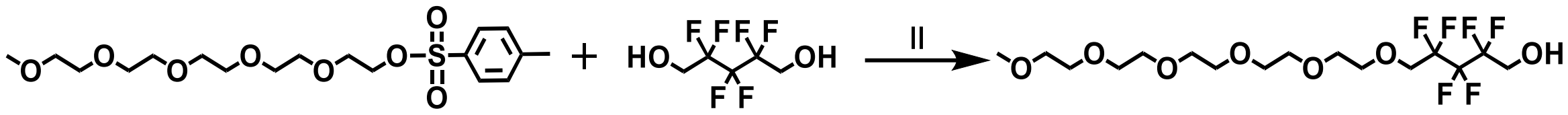

Fluorine-substituted diol for the synthesis of compound A4-1was replaced with 2,2,3,3,4,4-hexafluoro-1,5-pentanediol (A4-3) using the same molar stoichiometry. The rest of synthetic procedure was same as the compound 5a-1 (10.25g, 48.69% yield). ^{1}H NMR (400 MHz, $CDCl_3$) δ (ppm) = 4.01 (t1, *J* = 14.8 Hz, 2H), (t2, *J* = 14.4 Hz, 2H), 3.77 (s, 1H), 3.74 – 3.70 (m, 2H), 3.67 – 3.55 (m, 16H), 3.51 (m, 2H), 3.33 (s, 3H). ^{19}F NMR (376 MHz, $CDCl_3$-d) δ (ppm) = −120.18 (t, *J* = 8.8 Hz, 2F), −122.64 (t, *J* = 8.3 Hz, 2F), −126.84 (s, 2F). ^{13}C NMR (101 MHz, $CDCl_3$-d) δ (ppm) = 118.75 – 109.11 (m), 72.03, 71.49, 70.70, 70.68, 70.61, 70.57, 70.55, 70.49, 68.43, 68.18, 67.93, 60.58, 60.34, 60.10, 59.05. HR-ESI-MS (m/z): calculated: $C_{16}H_{28}F_6O_7$, $[M+H]^+$: 447.1812; found: 447.1812.

(5) Synthesis of 19,19,20,20,21,21,22,22-octafluoro-2,5,8,11,14,17-hexaoxatricosan-23-ol (A5-4).

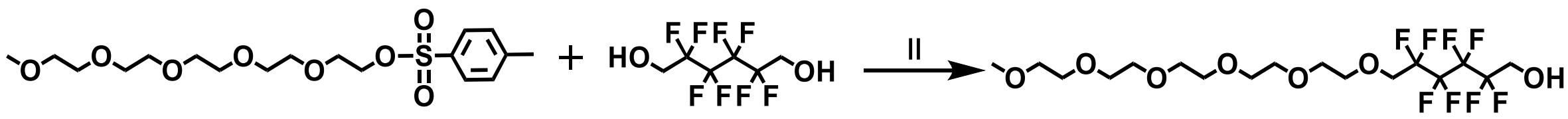

The compound was prepared following the synthetic protocol reported in Ref. [19].

(6) Synthesis of 19,19,20,20,21,21,22,22,23,23,24,24-dodecafluoro-2,5,8,11,14,17-hexaoxatricos-an-25-ol (A5-6).

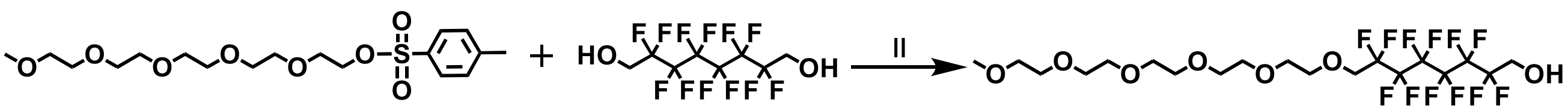

Fluorine-substituted diol for the synthesis of compound A4-1 was replaced with 2,2,3,3,4,4,5,5,6,6,7,7-dodecafluoro-1,8-octanediol (A4-5) using the same molar stoichiometry. The rest of synthetic procedure was same as the compound 5a-1 (7.50g, 70.00% yield). ^{1}H NMR (400 MHz, $CDCl_3$) δ (ppm) = 4.04 (t1, *J* = 12.0 Hz, 2H), 4.01 (t2, *J* = 15.8 Hz, 2H), 3.77–3.75 (m, 2H), 3.68 – 3.60 (m, 16H), 3.55 – 3.51 (m, 2H), 3.36 (s, 3H), 3.18 (s, 1H). ^{19}F NMR (376 MHz, $CDCl_3$) δ (ppm) = −119.89 (t, *J* = 12.8 Hz, 2F), −122.07 (m, 8F), −122.37 (m, 2F), −123.63 (m, 4F). ^{13}C NMR (101 MHz, $CDCl_3$) δ

(ppm) = 115.81-108.25 (m), 72.37, 71.94, 70.76, 70.70, 70.61-70.51 (m), 68.57, 68.32, 68.07, 60.62, 60.37, 60.12, 59.06. HR-ESI-MS (m/z): calculated: $C_{19}H_{28}F_{12}O_7$, $[M+NH_4]^+$: 614.1982; found: 614.1977.

(7) Synthesis of 19,19-difluoro-2,5,8,11,14,17-hexaoxatricosan-20-yl-trifluoromethanesulfonate (A7-1)

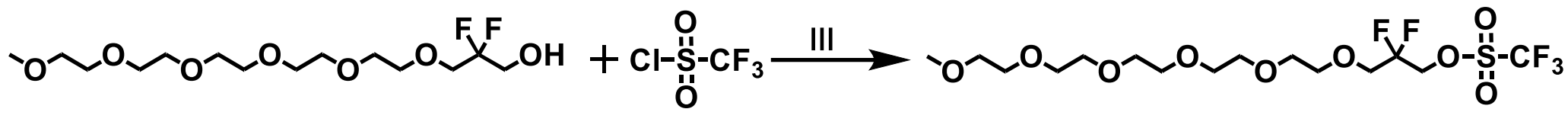

19,19-difluoro-2,5,8,11,14,17-hexaoxatricosan-20-ol (A5-1, 12.67 g, 36.58 mmol, 1.00 eq) was weighed into a 100 mL three-necked flask, sealed with a rubber stopper, and degassed by three vacuum-nitrogen purge cycles. 70 mL of anhydrous tetrahydrofuran was added via syringe, and the mixture was stirred at room temperature for 5 min. Triethylamine (8.20 g, 80.48 mmol, 2.20 eq) was added to the reaction system, stirred at room temperature for 15 min, then the three-necked flask was cooled to 0 °C in an ice-water bath. Trifluoromethanesulfonyl chloride (A6, 12.33 g, 73.16 mmol, 2.00 eq) was weighed and added dropwise slowly via syringe. The reaction was stirred at 0 °C for 3 h, then warmed to room temperature and stirred overnight, with progress monitored by TLC. After reaction completion, the reaction mixture was concentrated under reduced pressure, redissolved in 100 mL of diethyl ether, and suction-filtered to remove white insoluble solids. The organic phase was concentrated under reduced pressure and purified by column chromatography using ethyl acetate:petroleum ether = 1:1 (v/v) as eluent, affording 11.26 g of a colorless transparent oil with a yield of 64.34%. ^{1}H NMR (400 MHz, $CDCl_3$) δ (ppm) = 4.68 (t, *J* = 11.8 Hz, 2H), 3.84 (t, *J* = 12.1 Hz, 2H), 3.73 – 3.71 (m, 2H), 3.65 – 3.62 (m, 16H), 3.54 – 3.52 (m, 2H), 3.36 (s, 3H). ^{19}F NMR (376 MHz, $CDCl_3$) δ −74.30 (s, 3F), −113.77 (s, 2F). ^{13}C NMR (101 MHz, $CDCl_3$) δ 123.47 – 115.49 (m), 72.03, 71.70, 71.10, 70.77, 70.76, 70.70, 70.68, 70.65, 70.61, 70.44, 69.45, 69.15, 68.84, 59.13. HR-ESI-MS (m/z): calculated: $C_{15}H_{27}F_5O_9S$, $[M+H]^+$: 479.1369; found: 479.1373.

(8) Synthesis of 19,19,20,20-tetrafluoro-2,5,8,11,14,17-hexaoxatricosan-21-yl-trifluoromethane-sulfonate (A7-2)

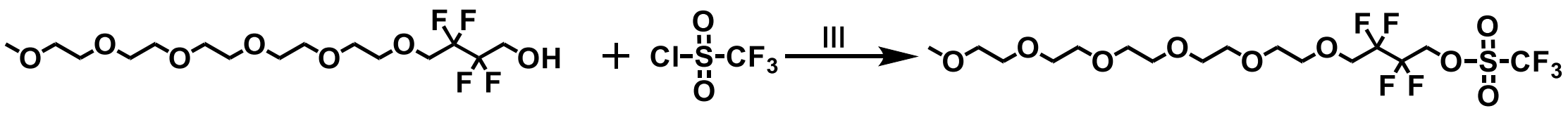

Fluorinated-2,5,8,11,14,17-hexaoxatricosan-ol for the synthesis of compound A7-1 was replaced with 19,19,20,20-tetrafluoro-2,5,8,11,14,17-hexaoxatricosan-21-ol (A5-2) using the same molar stoichiometry. The rest of synthetic procedure was same as the compound A7-1 (6.50g, 68.21% yield). ^{1}H NMR (400 MHz, $CDCl_3$) δ (ppm) = 4.90 (t, J = 14.0 Hz, 2H), 3.95 (t, J = 12.5 Hz, 2H), 3.74 – 3.71 (m, 2H), 3.68 – 3.58 (m, 16H), 3.55 – 3.51 (m, 2H), 3.36 (s, 3H). ^{19}F NMR (376 MHz, $CDCl_3$) δ (ppm) = –74.29 (s, 3F), –121.73 (s, 2F), –122.86 (s, 2F). ^{13}C NMR (101 MHz, $CDCl_3$) δ (ppm) = 123.34, 120.16, 116.98, 116.28- 111.18 (m), 115.56, 72.02, 71.93, 70.69, 70.66, 70.65, 70.61, 70.39, 70.16, 68.22, 67.93, 67.65, 59.11. HR-ESI-MS (m/z): calculated: $C_{16}H_{27}F_7O_9S$, $[M+H]^+$: 529.1337; found: 529.1335.

(9) Synthesis of 19,19,20,20,21,21-hexafluoro-2,5,8,11,14,17-hexaoxatricosan-22-yl-trifluoro-methanesulfonate (A7-3)

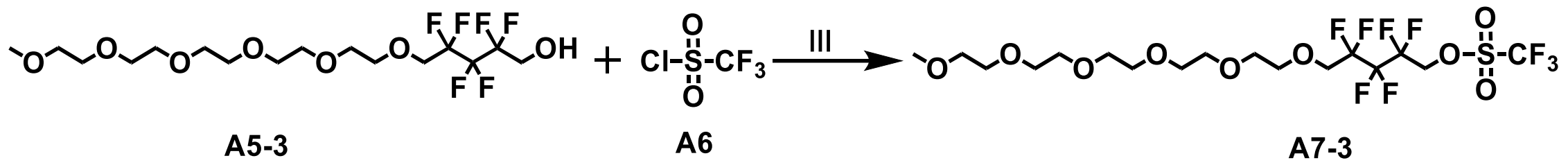

Fluorinated-2,5,8,11,14,17-hexaoxatricosan-ol for the synthesis of compound A7-1 was replaced with 19,19,20,20,21,21-hexafluoro-2,5,8,11,14,17-hexaoxatricosan-22-ol (A5-3) using the same molar stoichiometry. The rest of synthetic procedure was same as the compound A7-1 (10.50g, 79.13% yield). ^{1}H NMR (400 MHz, $CDCl_3$) δ (ppm) = 4.82 (t, J = 13.0 Hz, 2H), 4.01 (t, J = 14.0 Hz, 2H), 3.78 – 3.73 (m, 2H), 3.68 – 3.59 (m, 16H), 3.55 – 3.51 (m, 2H), 3.36 (s, 3H). ^{19}F NMR (376 MHz, $CDCl_3$) δ (ppm) = –74.12 (s, 3F), –119.74 (t, J = 10.3 Hz, 2F), –120.32 (t, J = 10.3 Hz, 2F), –125.94 (s, 2F). ^{13}C NMR (101 MHz, $CDCl_3$) δ (ppm) = 123.20, 120.02, 116.84, 116.84 – 109.43(m), 72.19, 71.92, 70.69, 70.62, 70.59, 70.57, 70.55, 70.51, 68.23, 67.98, 67.72, 59.01. HR-ESI-MS (m/z): calculated: $C_{17}H_{27}F_9O_9S$, $[M+H]^+$: 579.1305; found: 579.1306.

(10) Synthesis of 19,19,20,20,21,21,22,22-octafluoro-2,5,8,11,14,17-hexaoxatricosan-23-yl-tri-fluoromethanesulfonate (A7-4)

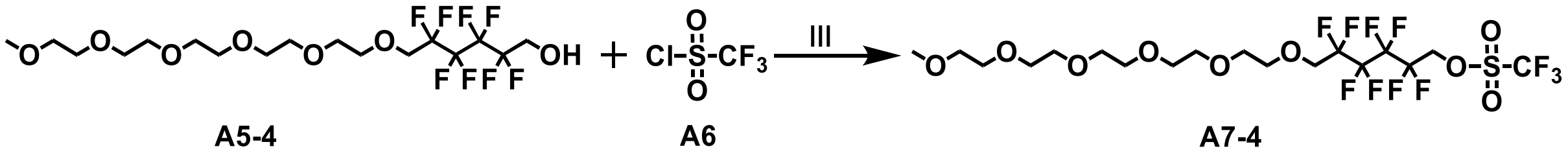

The compound was prepared following the synthetic protocol reported in Ref. [19].

(11) Synthesis of 19,19,20,20,21,21,22,22,23,23,24,24-dodecafluoro-2,5,8,11,14,17-hexaoxatricos -

an-25-yl-trifluoromethanesulfonate (A7-6)

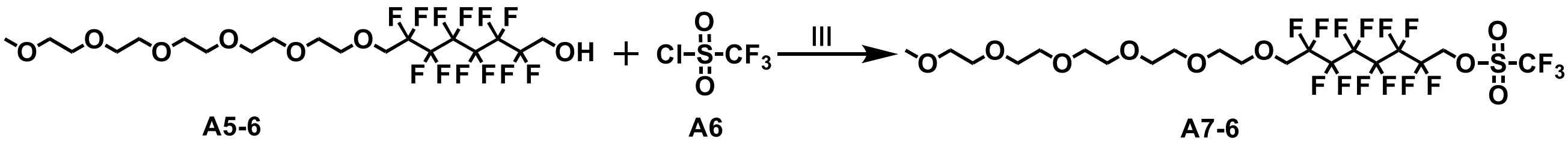

Fluorinated-2,5,8,11,14,17-hexaoxatricosan-ol for the synthesis of compound A5-1 was replaced with 19,19,20,20,21,21,22,22,23,23,24,24-dodecafluoro-2,5,8,11,14,17-hexaoxatricosan-25-ol (A5-6) using the same molar stoichiometry. The rest of synthetic procedure was same as the compound A7-1 (7.50g, 85.17% yield). ^{1}H NMR (400 MHz, $CDCl_3$) δ (ppm) = 4.81 (t, J = 12.4 Hz, 2H), 4.03 (t, J = 14.1 Hz, 2H), 3.77 (dd, J = 5.6, 3.5 Hz, 2H), 3.69 – 3.60 (m, 16H), 3.56 – 3.50 (m, 2H), 3.36 (s, 3H). ^{19}F NMR (376 MHz, $CDCl_3$) δ (ppm) = –74.02 (s, 3F), –119.75 – –119.87(m , 4F), –121.48 – –122.49 (m , 4F), –122.78 – –123.30 (m , 2F), –123.36 – –123.71 (m , 2F). ^{13}C NMR (101 MHz, $CDCl_3$) δ (ppm) = 123.29, 120.11,116.93, 116.05-107.99 (m), 72.44, 72.03, 70.84, 70.79, 70.71, 70.69, 70.67, 70.62, 68.65, 68.39, 68.14, 59.12. HR-ESI-MS (m/z): calculated: $C_{20}H_{27}F_{15}O_9S$, $[M+NH_4]^+$: 746.1475; found: 746.1475.

(12) Synthesis 2,5-bis(19,19-difluoro-2,5,8,11,14,17-hexaoxatricosan-20-yl)-3,6-di(thiophen-2-yl)-2,5-dihydropyrrolo[3,4-c]pyrrole-1,4-dione (A9-1)

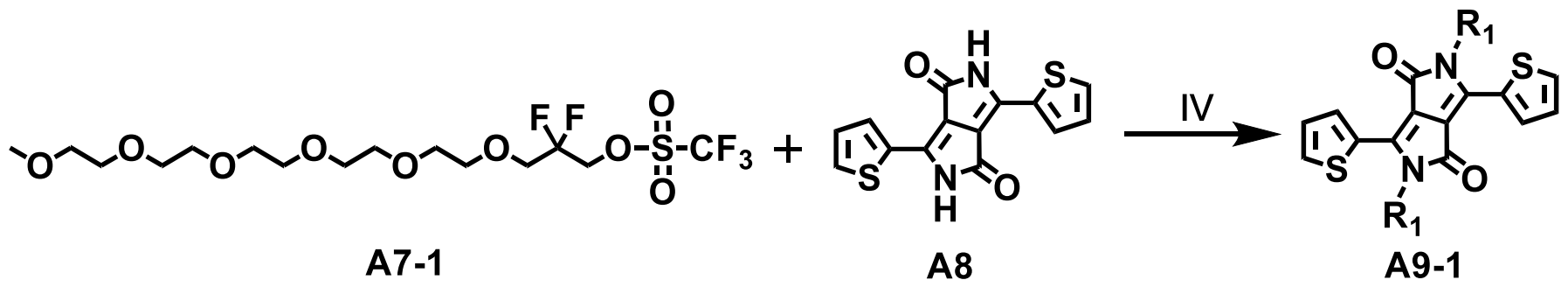

3,6-Dithienyl-pyrrolopyrroledione (A8, 1.51 g, 5.02 mmol, 1.00 eq) and K_2CO_3 (2.78 g, 20.08 mmol, 4.0 eq) were weighed into a 100 mL three-necked flask, which was degassed by three vacuum-nitrogen purge cycles to remove O_2 and H_2O. 40 mL of anhydrous N,N-dimethylformamide (DMF) was added via syringe, stirred at room temperature for 5 min, then heated to 90 °C and stirred for 1 h. 19,19-difluoro-2,5,8,11,14,17-hexaoxatricosan-20-yl-trifluoromethanesulfonate (A7-1) (6.00 g, 12.54 mmol, 2.50 eq) was weighed and added via syringe. The mixture was heated to 120 °C, stirred under reflux for 1 - 2 h, with reaction progress monitored by TLC. After completion, the system was cooled to room temperature and poured into 150 mL deionized water. The mixture was extracted with 300 mL dichloromethane, and the organic phase was washed repeatedly with saturated brine and deionized water. After concentration under reduced pressure, the residue was first purified by column

chromatography with ethyl acetate:methanol = 100:1 (v/v) to remove most impurities, then further purified with dichloromethane:methanol = 100:1 (v/v) as eluent, affording 1.66 g of a purple-black solid with a yield of 34.51%. ^{1}H NMR (400 MHz, $CDCl_3$) δ (ppm) = 8.71 (d, J = 3.9 Hz, 2H), 7.67 (d, J = 5.0 Hz, 2H), 7.27 – 7.25 (t, J = 4.0 Hz, 2H), 4.65 (t, J = 13.8 Hz, 4H), 3.83 (t, J = 12.5 Hz, 4H), 3.76 (m, 4H), 3.69 – 3.61 (m, 32H), 3.53 (m, 4H), 3.36 (s, 6H). ^{19}F NMR (376 MHz, $CDCl_3$) δ (ppm) = –107.36 (s, 4F). ^{13}C NMR (101 MHz, $CDCl_3$) δ (ppm) = 161.46, 140.73, 135.15, 131.25, 129.60, 128.48, 108.14, 72.02, 71.88, 71.52, 71.22, 70.93, 70.80, 70.70, 70.68, 70.65, 70.61, 59.15, 44.03, 43.77, 43.51. HR-ESI-MS (m/z): calculated: $C_{42}H_{60}F_4N_2O_{14}S_2$, $[M+H]^+$: 957.3495; found: 957.3496.

(13) Synthesis 2,5-bis(19,19,20,20-tetrafluoro-2,5,8,11,14,17-hexaoxatricosan-21-yl)-3,6-di(thio - phen-2-yl)-2,5-dihydropyrrolo[3,4-c]pyrrole-1,4-dione (A9-2)

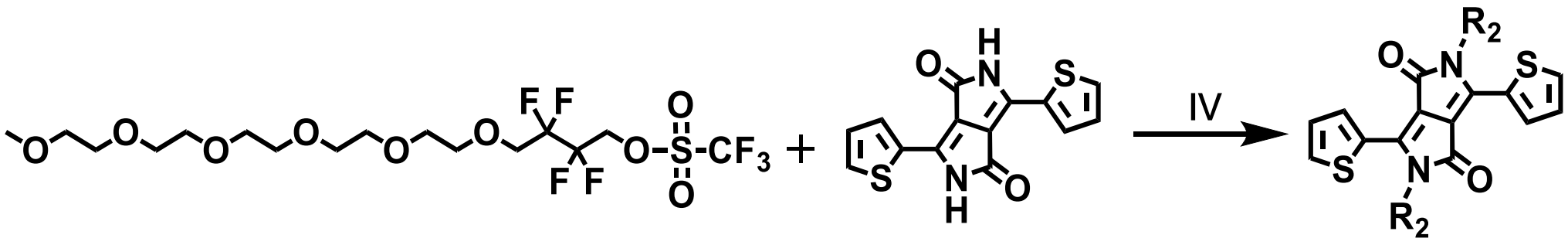

19,19-difluoro-2,5,8,11,14,17-hexaoxatricosan-20-yl-trifluoromethanesulfonate (A7-1) for the synthesis of compound A9-1 was replaced with 19,19,20,20-tetrafluoro-2,5,8,11,14,17-hexaoxatricosan-21-yl-trifluoromethanesulfonate (A7-2) using the same molar stoichiometry. The rest of synthetic procedure was the same as the compound A9-1 (0.99g, 24.72% yield). ^{1}H NMR (400 MHz, $CDCl_3$) δ (ppm) = 4.90 (t, J = 14.0 Hz, 2H), 3.95 (t, J = 12.5 Hz, 2H), 3.74 – 3.71 (m, 2H), 3.68 – 3.58 (m, 16H), 3.55 – 3.51 (m, 2H), 3.36 (s, 3H). ^{19}F NMR (376 MHz, $CDCl_3$) δ (ppm) = –116.77 (s, 4F), –121.48 (s, 4F). ^{13}C NMR (101 MHz, $CDCl_3$-d) δ (ppm) = 161.34, 140.63, 135.32, 131.35, 128.60, 108.30, 72.20, 72.03, 70.85, 70.80, 70.69, 70.67, 70.65, 70.62, 68.25, 67.98, 67.72, 59.16, 42.10, 41.52, 41.17. HR-ESI-MS (m/z): calculated: $C_{44}H_{60}F_8N_2O_{14}S_2$, $[M+H]^+$: 1057.3431; found: 1057.3428.

(14) Synthesis 2,5-bis(19,19,20,20,21,21-hexafluoro-2,5,8,11,14,17-hexaoxatricosan-22-yl)-3,6-di(thiophen-2-yl)-2,5-dihydropyrrolo[3,4-c]pyrrole-1,4-dione (A9-3)

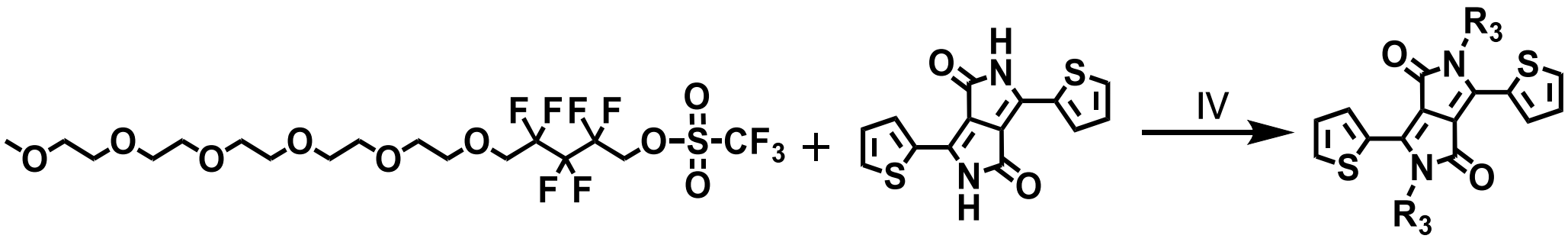

19,19-difluoro-2,5,8,11,14,17-hexaoxatricosan-20-yl-trifluoromethanesulfonate (A7-1) for the synthesis of compound A9-1 was replaced with 19,19,20,20,21,21-hexafluoro-2,5,8,11,14,17-hexaoxatricosan-22-yl-trifluoromethanesulfonate (A7-3) using the same molar stoichiometry. The rest of synthetic procedure was the same as the compound A9-1 (1.11g, 26.43% yield). 1H NMR (400 MHz, $CDCl_3$) δ (ppm) = 8.70 (d, J = 4.0 Hz, 2H), 7.69 (d, J = 5.0 Hz, 2H), 7.28 (t, J = 4.5 Hz, 2H), 4.83 (t, J = 15.6 Hz, 4H), 4.03 (t, J = 14.6 Hz, 4H), 3.78 (m, 4H), 3.68 – 3.61 (m, 32H), 3.53 (m, 4H), 3.36 (s, 6H). ^{19}F NMR (376 MHz, $CDCl_3$) δ (ppm) = –115.17 (t, J = 9.3 Hz, 4F), –119.51 (t, J = 9.6 Hz, 4F), –125.92 (s, 4F). ^{13}C NMR (101 MHz, $CDCl_3$) δ (ppm) = 161.19, 140.53, 135.55, 131.54, 129.01, 128.76, 108.32, 72.46, 72.05, 70.85, 70.75, 70.71, 70.69, 70.64, 68.62, 68.37, 68.14, 59.16, 41.7, 41.53, 41.32. HR-ESI-MS (m/z): calculated: $C_{46}H_{60}F_{12}N_2O_{14}S_2$, $[M+H]^+$: 1157.3368; found: 1157.3362.

(15) Synthesis 2,5-bis(19,19,20,20,21,21,22,22-octafluoro-2,5,8,11,14,17-hexaoxatricosan-23-yl)-3,6-di(thiophen-2-yl)-2,5-dihydropyrrolo[3,4-c]pyrrole-1,4-dione (A9-4)

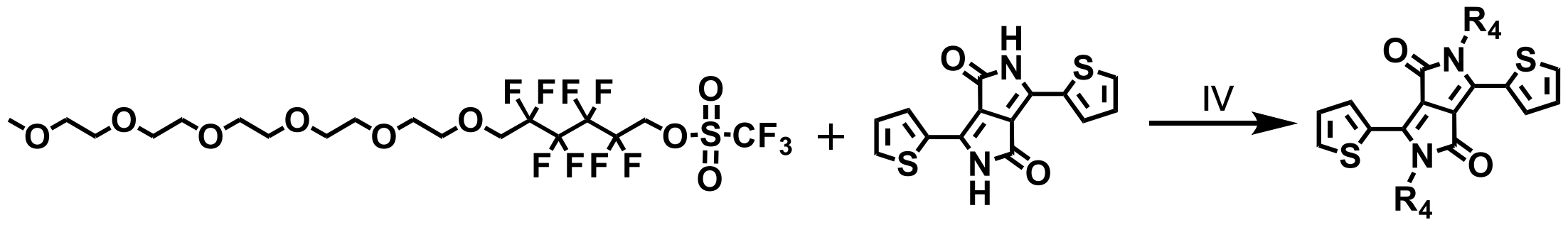

19,19-difluoro-2,5,8,11,14,17-hexaoxatricosan-20-yl-trifluoromethanesulfonate (A7-1) for the synthesis of compound A9-1 was replaced with 19,19,20,20,21,21,22,22-octafluoro-2,5,8,11,14,17-hexaoxatricosan-23-yl-trifluoromethanesulfonate (A7-4) using the same molar stoichiometry. The rest of synthetic procedure was the same as the compound A9-1. The compound was prepared following the synthetic protocol reported in Ref. [19].

(16) Synthesis 2,5-bis(19,19,20,20,21,21,22,22,23,23,24,24-dodecafluoro-2,5,8,11,14,17-hexaoxa -tricos-an-25-yl)-3,6-di(thiophen-2-yl)-2,5-dihydropyrrolo[3,4-c]pyrrole-1,4-dione (A9-6)

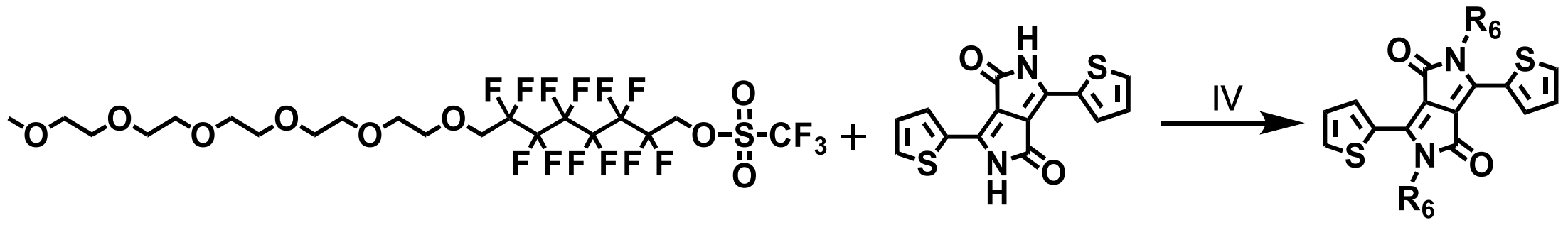

19,19-difluoro-2,5,8,11,14,17-hexaoxatricosan-20-yl-trifluoromethanesulfonate (A7-1) for the

synthesis of compound A9-1 was replaced with 19,19,20,20,21,21,22,22,23,23,24,24-dodecafluoro-2,5,8,11,14,17-hexaoxatricosan-25-yl-trifluoromethanesulfonate (A7-6) using the same molar stoichiometry. The rest of synthetic procedure was the same as the compound A9-1 (0.85g, 16.11% yield). ^{1}H NMR (400 MHz, $CDCl_3$) δ (ppm) = 8.71 (d, *J* = 3.9 Hz, 2H), 7.70 (d, *J* = 5.0 Hz, 2H), 7.33 – 7.27 (m, 2H), 4.86 (t, *J* = 15.3 Hz, 4H), 4.03 (t, *J* = 14.0 Hz, 4H), 3.78 (m, 4H), 3.65 (m, 32H), 3.56 – 3.52 (m, 4H), 3.37 (s, 6H). ^{19}F NMR (376 MHz, $CDCl_3$) δ (ppm) = –115.01(s, 4F), –119.79 (t, *J* = 13.8 Hz. 4F), –121.65 (m, 4F), –122.06 (m, 4F), –123.39 (m, 8F). ^{13}C NMR (101 MHz, $CDCl_3$) δ (ppm) = 161.10, 140.48, 135.74, 131.64, 128.88, 128.82, 108.35, 72.46, 72.05, 70.86, 70.80, 70.73, 70.71, 70.69, 70.64, 68.68, 68.43, 68.18, 59.16, 41.55, 41.46, 41.12. HR-ESI-MS (m/z): calculated: $C_{52}H_{60}F_{24}N_2O_{14}S_2$, $[M+NH_4]^+$: 1474.3441; found: 1474.3446.

(17) Synthesis of 3,6-bis(5-bromothiophen-2-yl)-2,5-bis(19,19-difluoro-2,5,8,11,14,17-hexaoxa-tricosan-20-yl)-2,5-dihydropyrrolo[3,4-c]pyrrole-1,4-dione (A10-1)

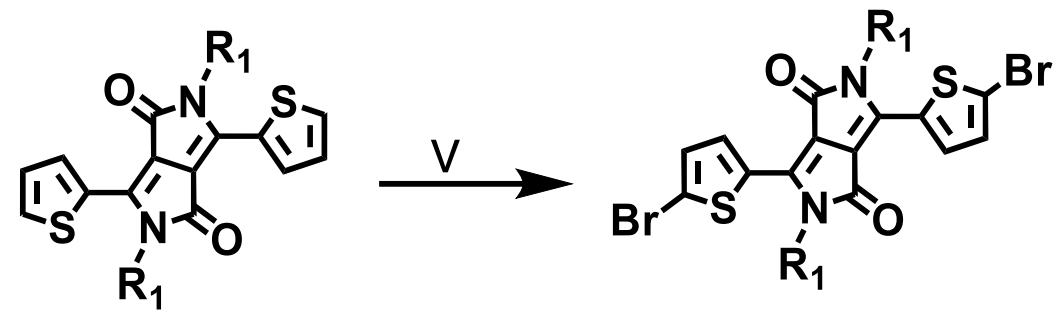

A9-1 A10-1

Compound A9-1 (1.66 g, 1.73 mmol, 1.00 eq) was weighed into a 100 mL single-necked flask, 50 mL chloroform was added, and stirred at room temperature for 15 min. The flask was then cooled to 0 °C in an ice-water bath. N-Bromosuccinimide (NBS, 0.65 g, 3.64 mmol, 2.1 eq) was weighed and added in portions, with reaction progress monitored by TLC. After completion, 25 mL deionized water was added to quench the reaction, and the mixture was separated with a separatory funnel. The organic phase was washed repeatedly with saturated brine and deionized water, concentrated under reduced pressure, and purified by column chromatography with dichloromethane:methanol = 100:1 (v/v) as eluent, affording 1.21 g of a purple solid, 62.69% yield. ^{1}H NMR (400 MHz, $CDCl_3$) δ (ppm) = 8.44 (d, *J* = 4.2 Hz, 2H), 7.21 (d, *J* = 4.2 Hz, 2H), 4.56 (t, *J* = 14.1 Hz, 4H), 3.83 (t, *J* = 12.3 Hz, 4H), 3.78 – 3.75 (m, 4H), 3.69 – 3.61 (m, 32H), 3.54 – 3.52 (m, 4H), 3.36 (s, 6H). ^{19}F NMR (376 MHz, $CDCl_3$) δ (ppm) = –107.34 (s, 4F). ^{13}C NMR (101 MHz, $CDCl_3$) δ (ppm) = 161.17, 139.63, 135.26, 131.61, 131.04, 119.81, 108.26, 72.07, 71.96, 71.67,71.45, 71.15, 70.88, 70.73, 70.71, 70.62, 59.26, 44.19, 43.93, 43.68. HR-ESI-MS (m/z): calculated: $C_{42}H_{58}Br_2F_4N_2O_{14}S_2$, $[M+H]^+$: 1115.1685; found:

1115.1701.

(18) Synthesis of 3,6-bis(5-bromothiophen-2-yl)-2,5-bis(19,19,20,20-tetrafluoro-2,5,8,11,14,17-hexaoxatricosan-21-yl)-2,5-dihydropyrrolo[3,4-c]pyrrole-1,4-dione (A10-2)

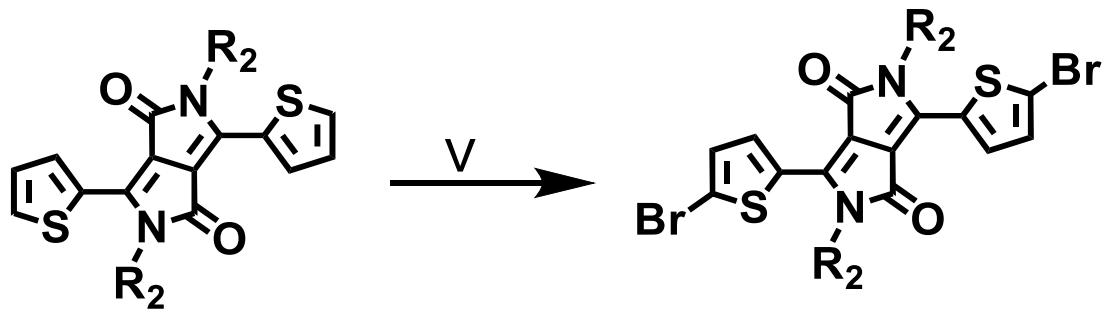

A9-2 A10-2

Compound A9-1 was replaced with compound A9-2 using the same molar stoichiometry. The rest of synthetic procedure was the same as that of compound A10-1 (0.51 g, 53.51% yield). ^{1}H NMR (400 MHz, $CDCl_3$) δ (ppm) = 8.42 (d, J = 4.2 Hz, 2H), 7.22 (d, J = 4.2 Hz, 2H), 4.76 (t, J = 16.1 Hz, 4H), 4.03 (t, J = 13.8 Hz, 4H), 3.82 – 3.79 (m, 4H), 3.69 – 3.62 (m, 32H), 3.54 – 3.52 (m, 4H), 3.36 (s, 6H). ^{19}F NMR (376 MHz, $CDCl_3$) δ (ppm) = –116.75 (s, 4F), –121.41 (s, 4F). ^{13}C NMR (101 MHz, $CDCl_3$) δ (ppm) = 161.34, 140.63, 135.32, 131.35, 128.60, 108.30, 72.20, 72.03, 70.85, 70.80, 70.69, 70.67, 70.65, 70.62, 68.25, 67.98, 67.72, 59.16, 42.10, 41.52, 41.17. HR-ESI-MS (m/z): calculated: $C_{44}H_{58}Br_2F_8N_2O_{14}S_2$, $[M+H]^+$: 1215.1621; found: 1215.1586.

(19) Synthesis of 3,6-bis(5-bromothiophen-2-yl)-2,5-bis(19,19,20,20,21,21-hexafluoro-2,5,8,11,14,17-hexaoxatricosan-22-yl)-2,5-dihydropyrrolo[3,4-c]pyrrole-1,4-dione (A10-3)

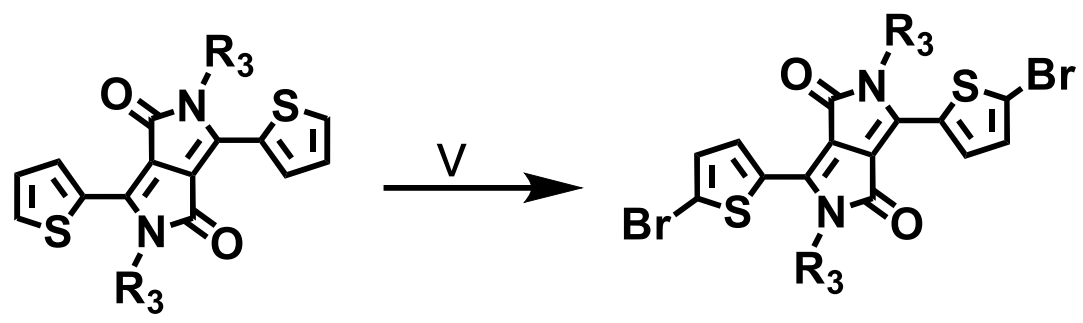

A9-3 A10-3

Compound A9-1 was replaced with compound A9-3 using the same molar stoichiometry. The rest of synthetic procedure was the same as that of compound A10-1 (0.77 g, 61.11% yield). ^{1}H NMR (400 MHz, $CDCl_3$) δ 8.40 (d, J = 4.2 Hz, 2H), 7.23 (d, J = 4.2 Hz, 2H), 4.72 (t, J = 15.6 Hz, 4H), 4.04 (t, J = 14.5 Hz, 4H), 3.81 – 3.77 (m, 4H), 3.68 – 3.62 (m, 32H), 3.53 (m, 4H), 3.36 (s, 6H). ^{19}F NMR (376 MHz, $CDCl_3$) δ -115.10 (s, 4F), -119.40 (t, J = 9.5 Hz, 4F), -125.84 (s, 4F). ^{13}C NMR (101 MHz, $CDCl_3$) δ 160.86, 139.34, 135.41, 131.84, 130.39, 120.26, 108.43, 77.48, 76.84, 72.45, 72.04, 70.84, 70.75, 70.70, 70.67, 70.63, 68.60, 68.36, 68.12, 59.16, 41.89, 41.58, 41.44. HR-ESI-MS (m/z): calculated: $C_{46}H_{58}Br_2F_{12}N_2O_{14}S_2$, $[M+H]^+$: 1315.1557; found: 1315.1527.

(20) Synthesis of 3,6-bis(5-bromothiophen-2-yl)-2,5-bis(19,19,20,20,21,21,22,22-octafluoro-2,5,8,11,14,17-hexaoxatricosan-23-yl)-2,5-dihydropyrrolo[3,4-c]pyrrole-1,4-dione (A10-4)

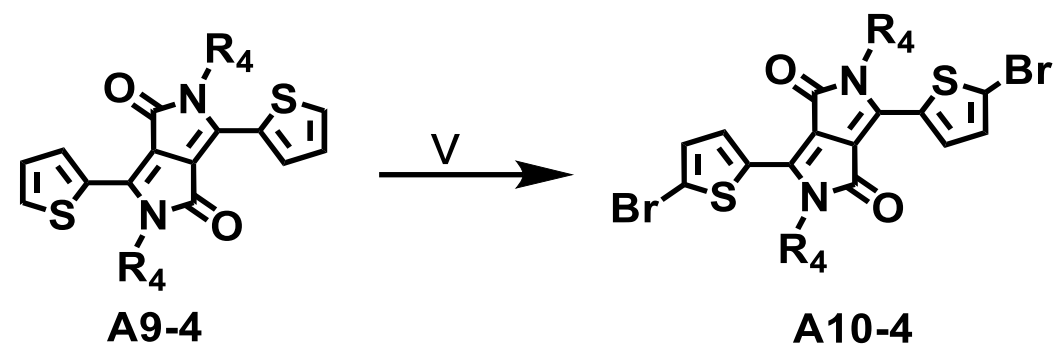

The compound was prepared following the synthetic protocol reported in Ref. [19].

(21) Synthesis of 3,6-bis(5-bromothiophen-2-yl)-2,5-bis(19,19,20,20,21,21,22,22-octafluoro-2,5,8,11,14,17-hexaoxatricosan-23-yl)-2,5-dihydropyrrolo[3,4-c]pyrrole-1,4-dione (A10-6)

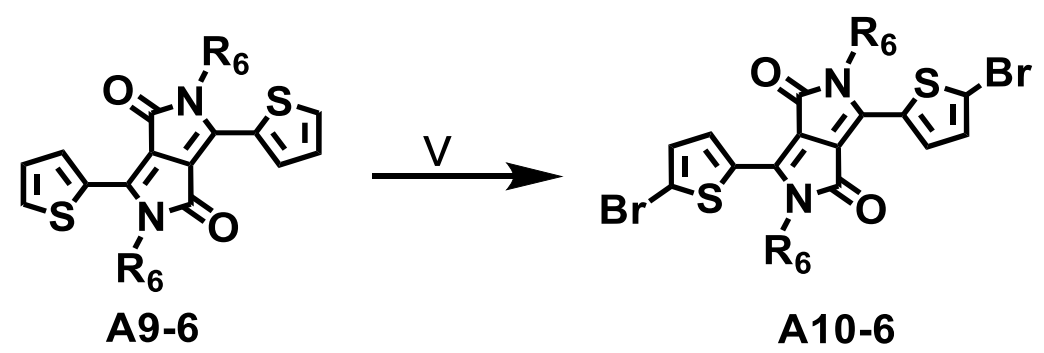

Compound A9-1 was replaced with compound A9-6 using the same molar stoichiometry. The rest of synthetic procedure was the same as that of compound A10-1 (0.65 g, 53.27% yield). 1H NMR (400 MHz, $CDCl_3$-d) δ (ppm) = 8.40 (d, J = 4.2 Hz, 2H), 7.25 (d, J = 4.2 Hz, 2H), 4.75 (t, J = 15.2 Hz, 4H), 4.04 (t, J = 14.0 Hz, 4H), 3.81 – 3.76 (m, 4H), 3.68 – 3.62 (m, 32H), 3.56 – 3.52 (m, 4H), 3.37 (s, 6H). ^{19}F NMR (376 MHz, $CDCl_3$-d) δ (ppm) = -114.91(s, 4F), -119.77 (t, J = 14.1 Hz, 4F), -121.60 (m, 4F), -122.02 (m, 4F), -123.34 (m, 8F). ^{13}C NMR (101 MHz, $CDCl_3$-d) δ (ppm) = 160.78, 139.31, 135.59, 131.96, 130.18, 120.44, 108.49, 72.47, 72.06, 70.87, 70.80, 70.72, 70.69, 70.65, 68.69, 68.44, 68.19, 59.16, 41.72, 41.48, 41.28. HR-ESI-MS (m/z): calculated: $C_{52}H_{58}Br_2F_{24}N_2O_{14}S_2$, $[M+NH_4]^+$: 1632.1631; found: 1632.1605.

(22) Synthesis of polymer: 2FG-P

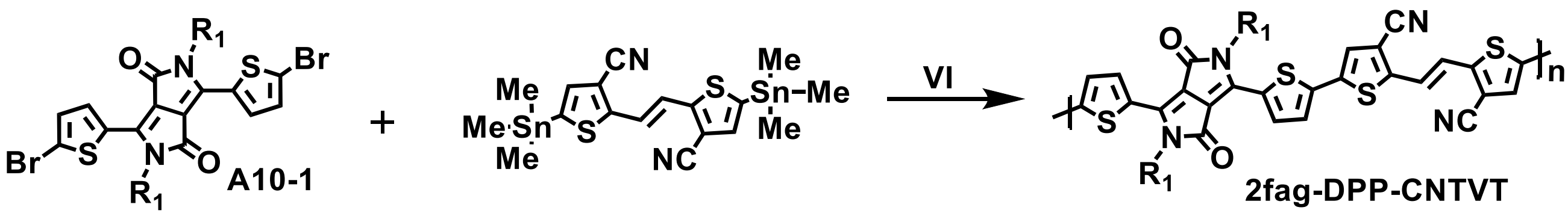

5 mL polymerization tube was charged sequentially with 113.3 mg (0.1017 mmol, 1.00 eq) of monomer (A10-1), 57.8 mg (0.1017 mmol, 1.00 eq) of (E)-2,2'-(ethene-1,2-diyl)bis(5-(trimethylstannyl)-thiophene-3-carbonitrile), 3.0 mg of tris(dibenzylideneacetone)dipalladium ($Pd_2(dba)_3$), and 7.98 mg

of tri(o-tolyl)phosphine (P(o-tol)$_3$). Chlorobenzene (2 mL) was added as the solvent, and the tube was sealed and frozen with liquid nitrogen. Under frozen conditions, the tube was evacuated for 15 min and then backfilled with nitrogen. This freeze-evacuation-nitrogen backfilling cycle was repeated three times. After the reaction mixture thawed, the polymerization tube was placed in an oil bath and heated to 135 °C. The degree of polymerization was controlled by adjusting the reaction time; upon completion, the tube was cooled to room temperature. The reaction product was dissolved in appropriate hexafluoroisopropanol and added dropwise to 100 mL of a methanol-hydrochloric acid mixture (v/v = 100:2.5) for precipitation, followed by suction filtration. The filter residue was wrapped in filter paper and subjected to Soxhlet extraction with methanol (8 h), acetone (8 h), n-hexane (8 h), and hexafluoroisopropanol (8 h) sequentially. After the final extraction with hexafluoroisopropanol, the polymer solution was concentrated to dryness under reduced pressure via rotary evaporation to afford a solid. Methanol was added to soak the solid overnight, and the polymer was collected by filtration, followed by drying under reduced pressure (112.9 mg, 92.98% yield).

(23) Synthesis of polymer: 4FG-P

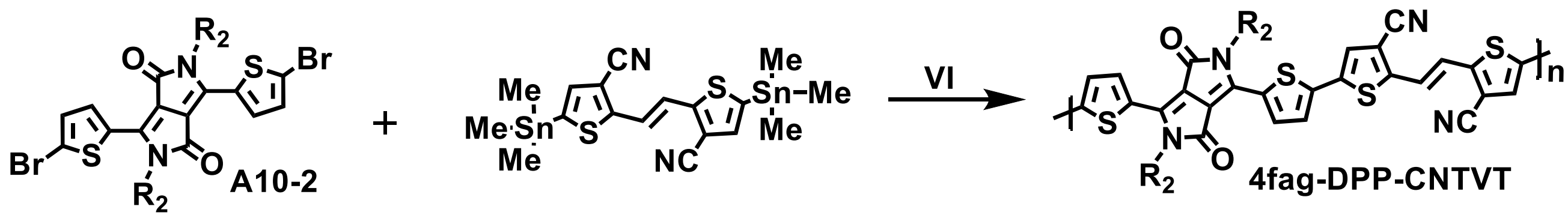

Compound A10-1 was replaced with compound A10-2 using the same molar stoichiometry. The rest of synthetic procedure was identical to that of polymer 4FG-P(115.2 mg, 97.43% yield).

(24) Synthesis of polymer: 6FG-P

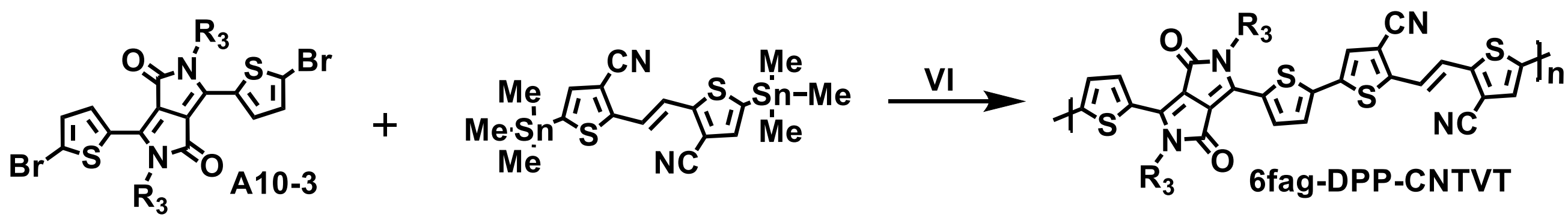

Compound A10-1 was replaced with compound A10-3 using the same molar stoichiometry. The rest of synthetic procedure was identical to that of polymer 6FG-P(101.7 mg, 95.27% yield).

(25) Synthesis of polymer: 8FG-P

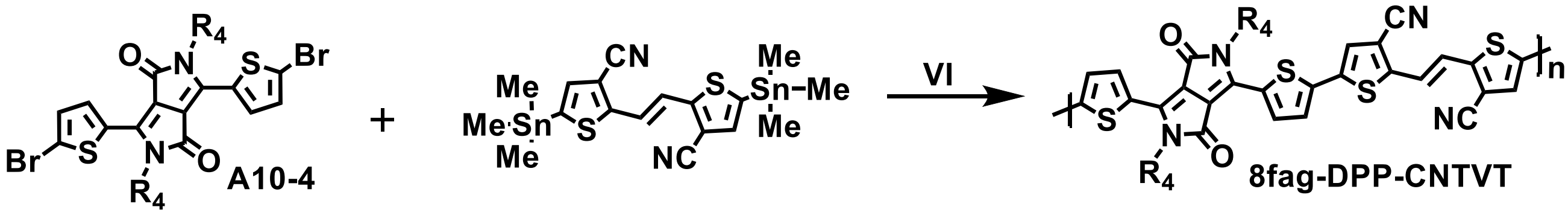

Compound A10-1 was replaced with compound A10-4 using the same molar stoichiometry. The rest of synthetic procedure was identical to that of polymer 8FG-P(104.7 mg, 96.77% yield).

(26) Synthesis of polymer: 12FG-P

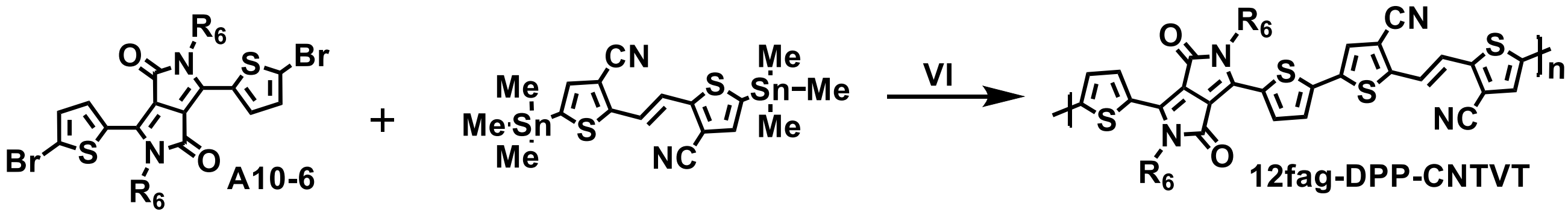

Compound A10-1 was replaced with compound A10-3 using the same molar stoichiometry. The rest of synthetic procedure was identical to that of polymer 12FG-P(105.6 g, 94.86% yield).

(27) Synthesis of polymer batches with different molecular mass: 8FG-P-MMn, 12FG-P-MMn, 8FG-P-LMn, and 12FG-P-LMn.

All four polymer batches were synthesized via the same Stille polycondensation route with precisely controlled monomer stoichiometry. The molecular mass of each batch was tuned exclusively by varying the polymerization reaction time, with all other reaction parameters (temperature, catalyst loading, monomer concentration, and solvent) kept strictly identical. The medium-molecular-weight batches (8FG-P-MMn and 12FG-P-MMn) were prepared by terminating the polymerization prior to the gel point (~ 4 h), while the low-molecular-weight batches (8FG-P-LMn and 12FG-P-LMn) were obtained via earlier reaction quenching to prematurely terminate chain propagation (~ 2.5 h). All batches were subjected to the same purification workflow, including sequential Soxhlet extraction and repeated precipitation, to ensure consistent chemical purity. The number-average molecular weights (M_n) and dispersities (Đ) of the final products are as follows: 8FG-P-MMn (100.7 g, 93.07% yield, Mn = 39.8 kDa, Đ = 1.9), 12FG-P-MMn (101.6 g, 91.28% yield, Mn = 27.2 kDa, Đ = 1.5), 8FG-P-LMn (81.8 g, 75.63% yield, Mn = 21.3 kDa, Đ = 1.8), and 12FG-P-LMn (56.2 g, 50.45% yield, Mn = 15.6 kDa, Đ = 1.3).

21.NMR Spectra

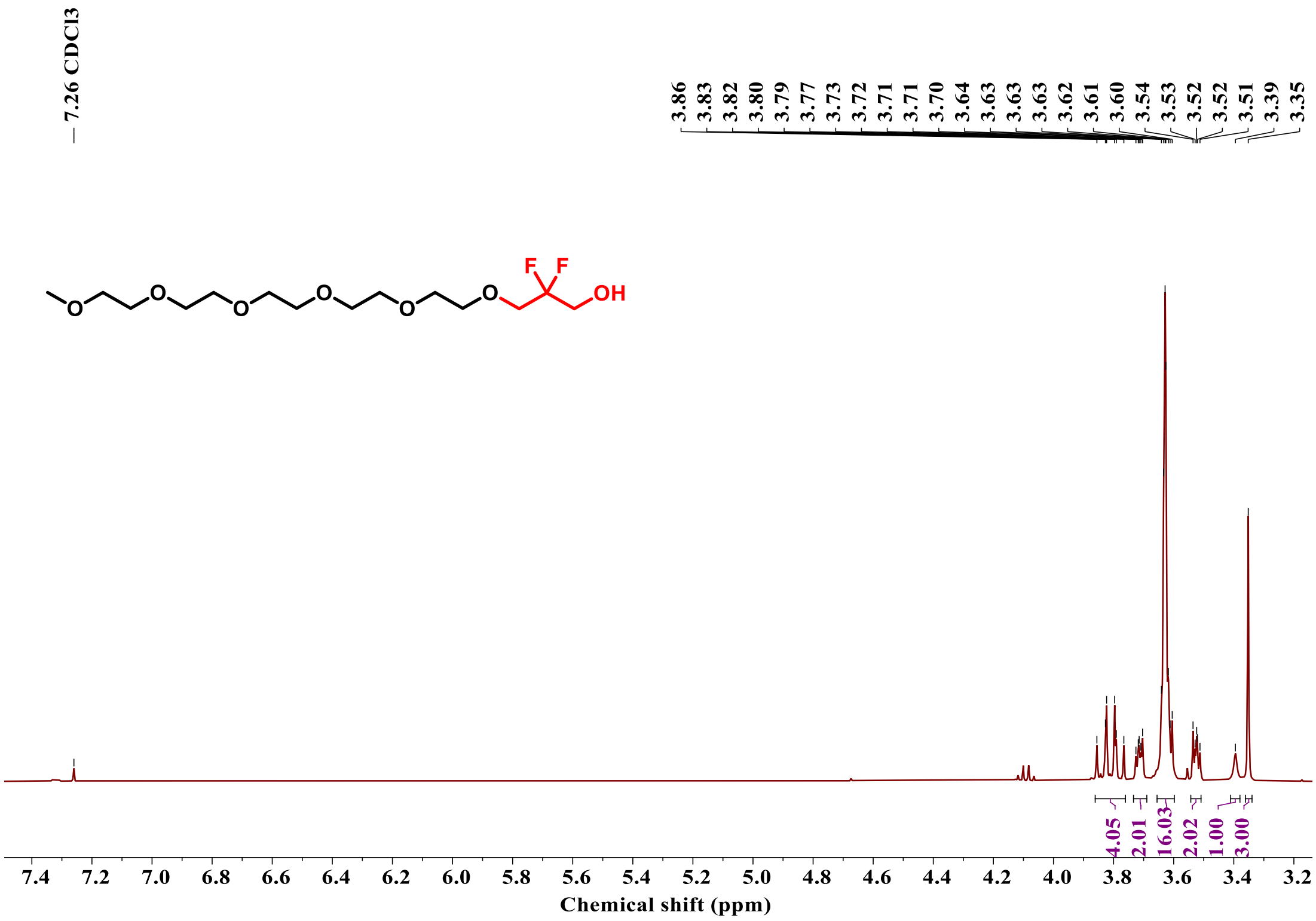

Figure S35. ^{1}H NMR spectrum of compound A5-1.

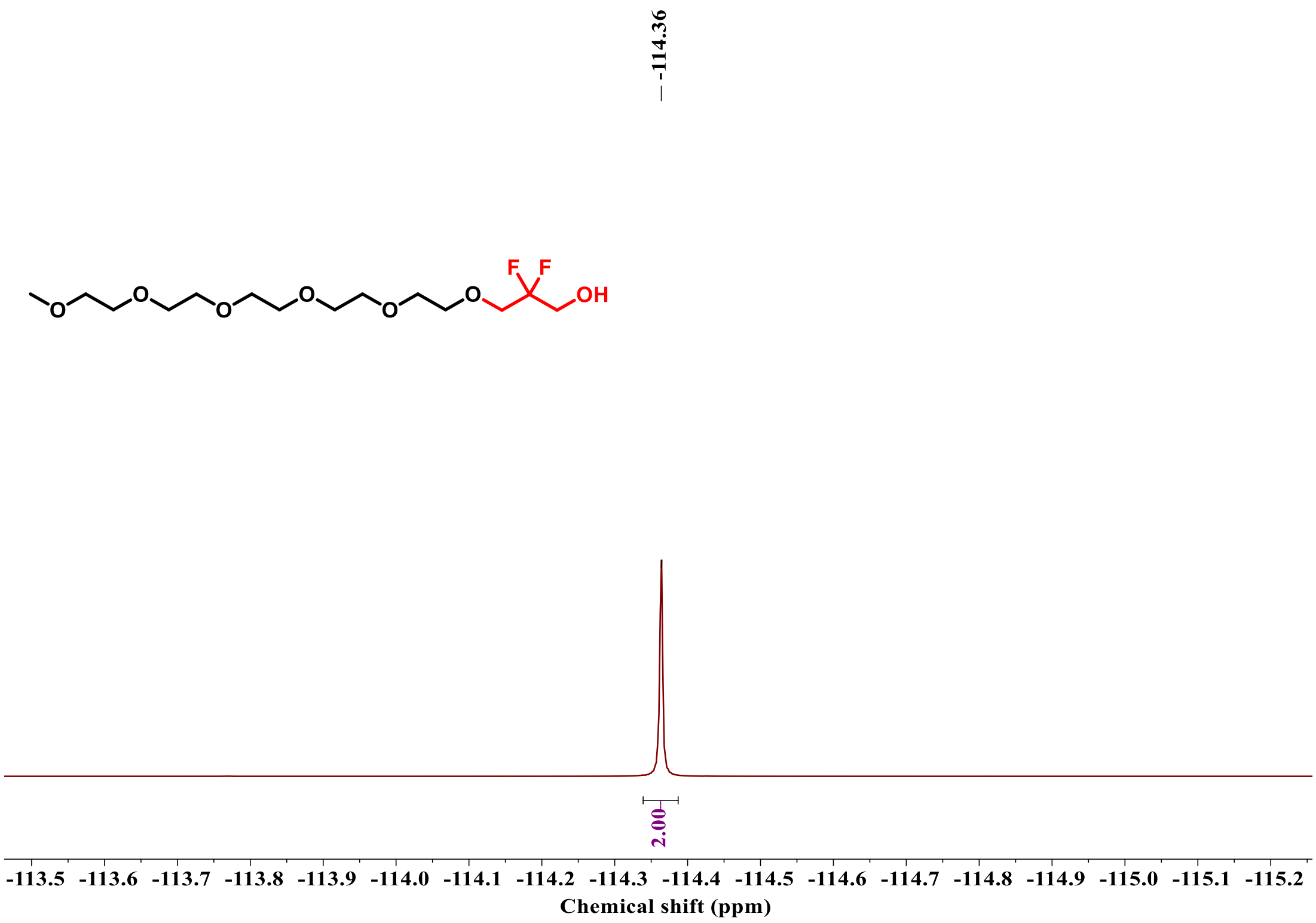

Figure S36 ^{19}F NMR spectrum of compound A5-1.

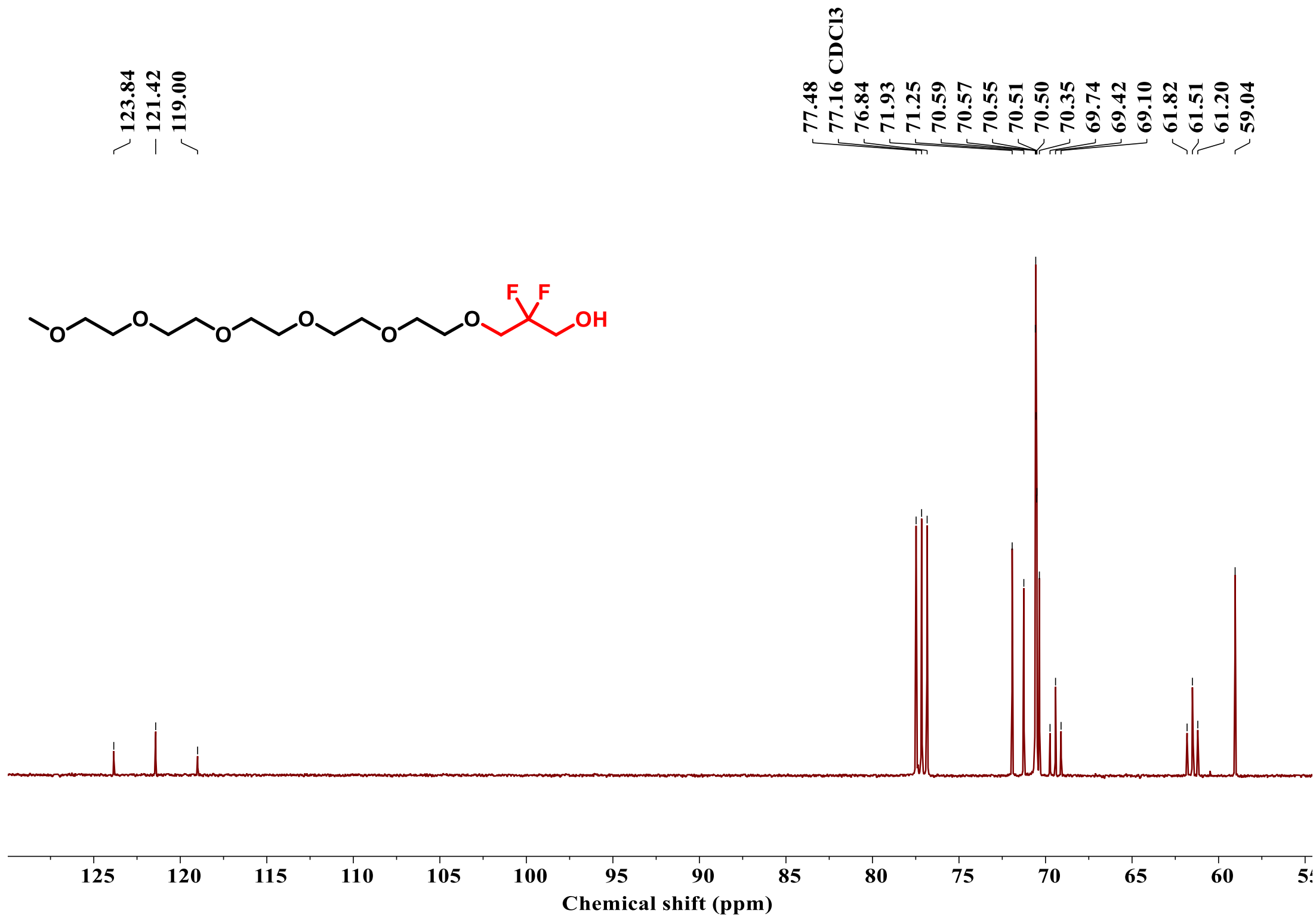

Figure S37. ^{13}C NMR spectrum of compound A5-1.

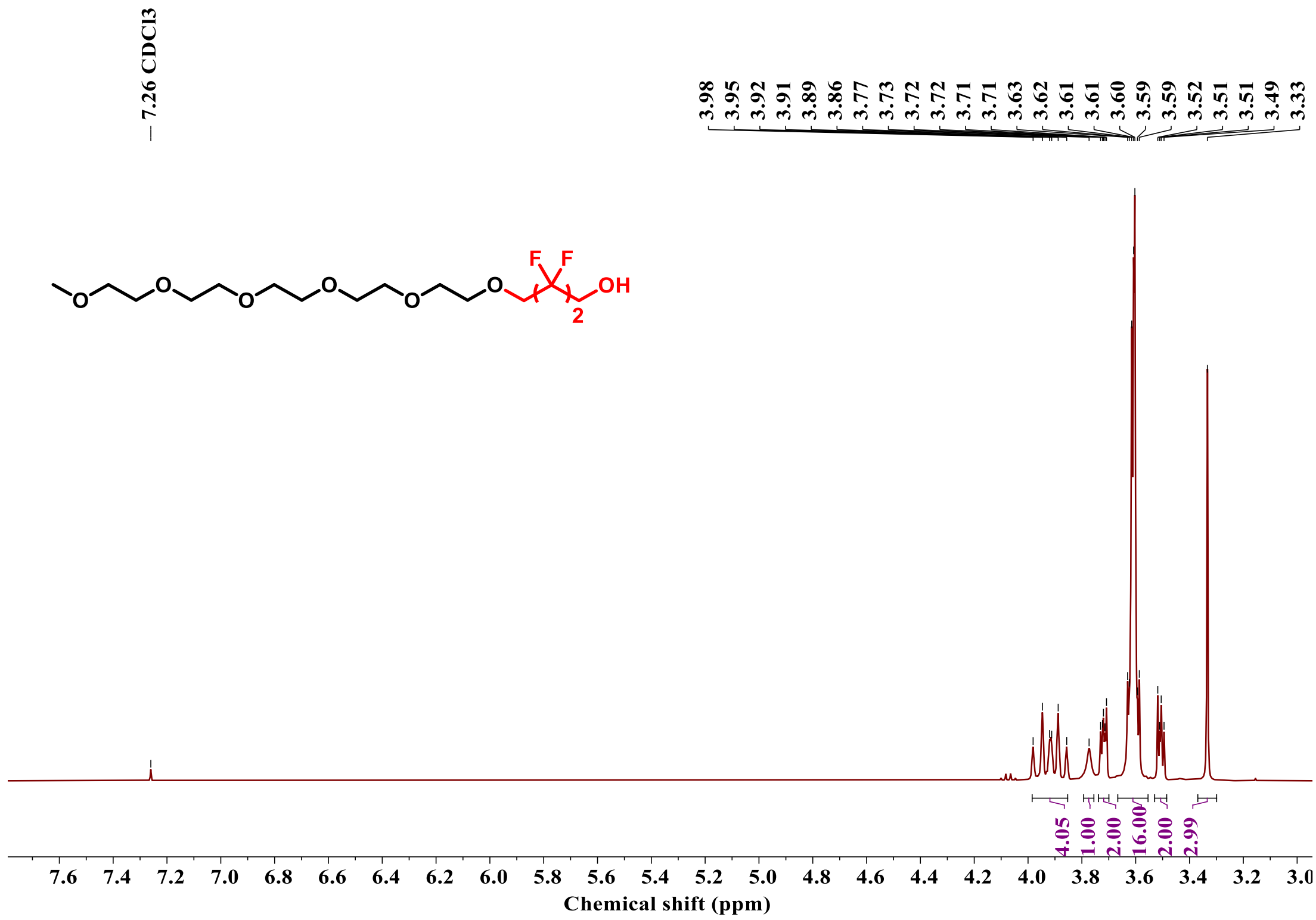

Figure S38. ^{1}H NMR spectrum of compound A5-2.

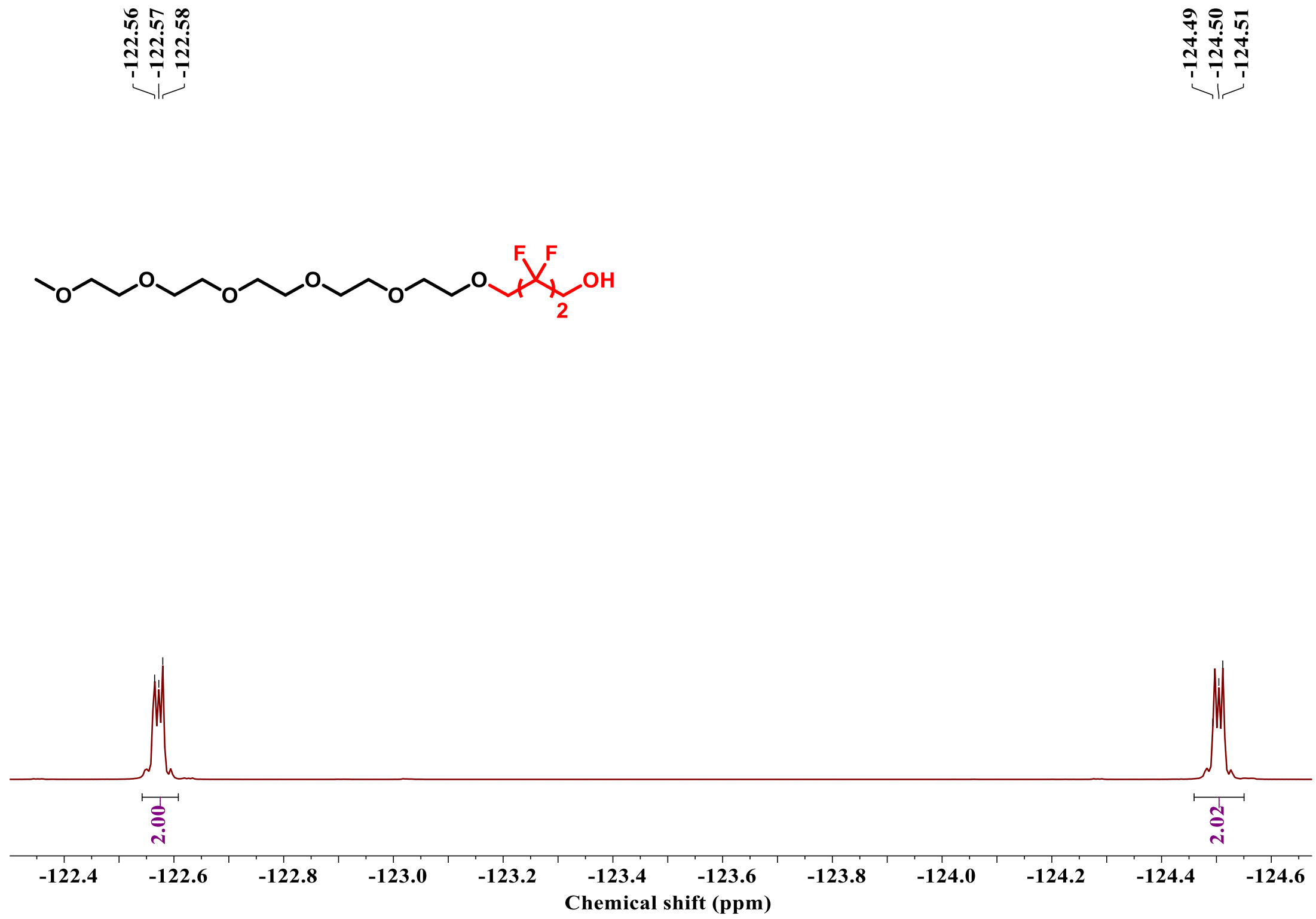

Figure S39. ^{19}F NMR spectrum of compound A5-2.

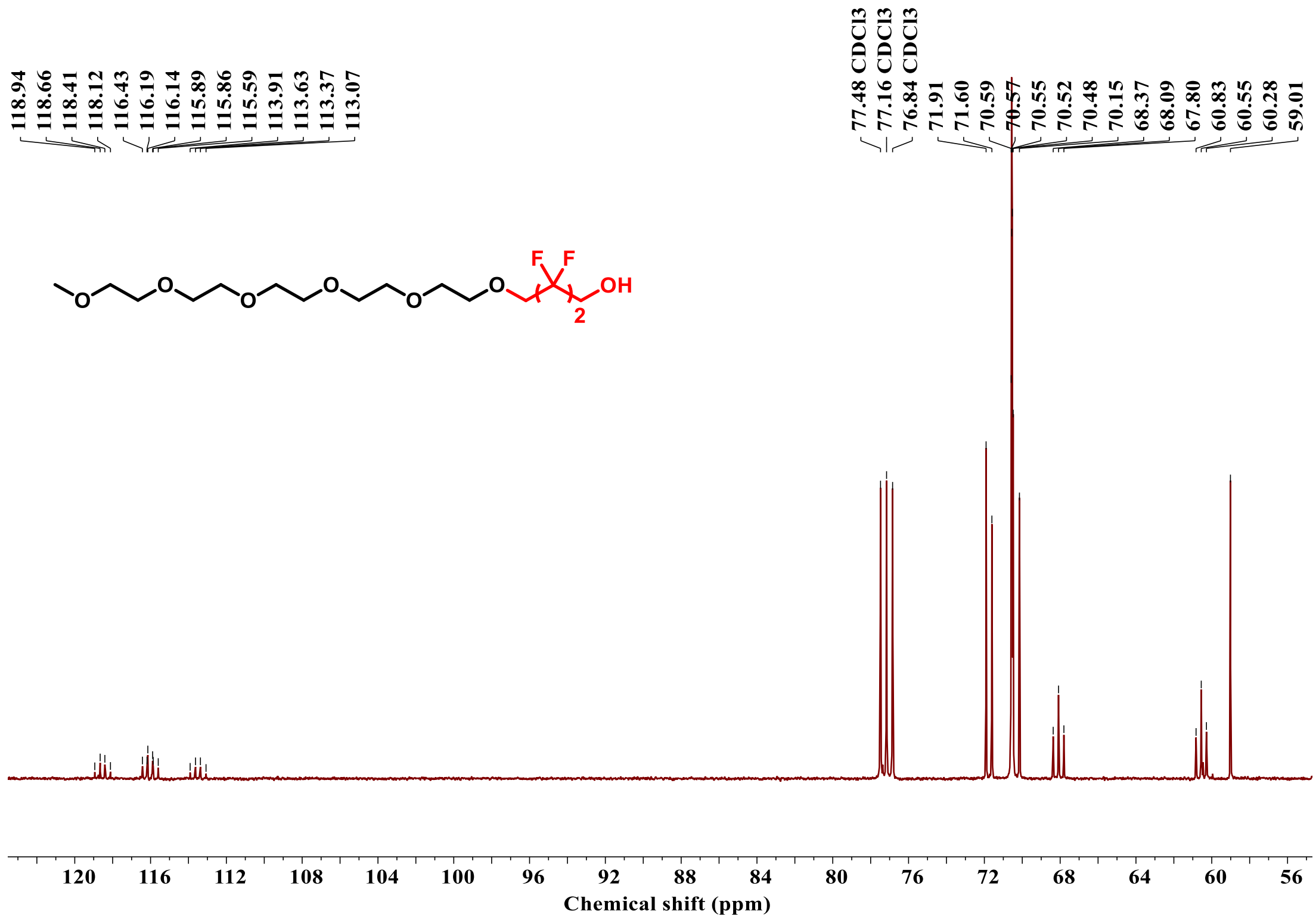

Figure S40. ^{13}C NMR spectrum of compound A5-2.

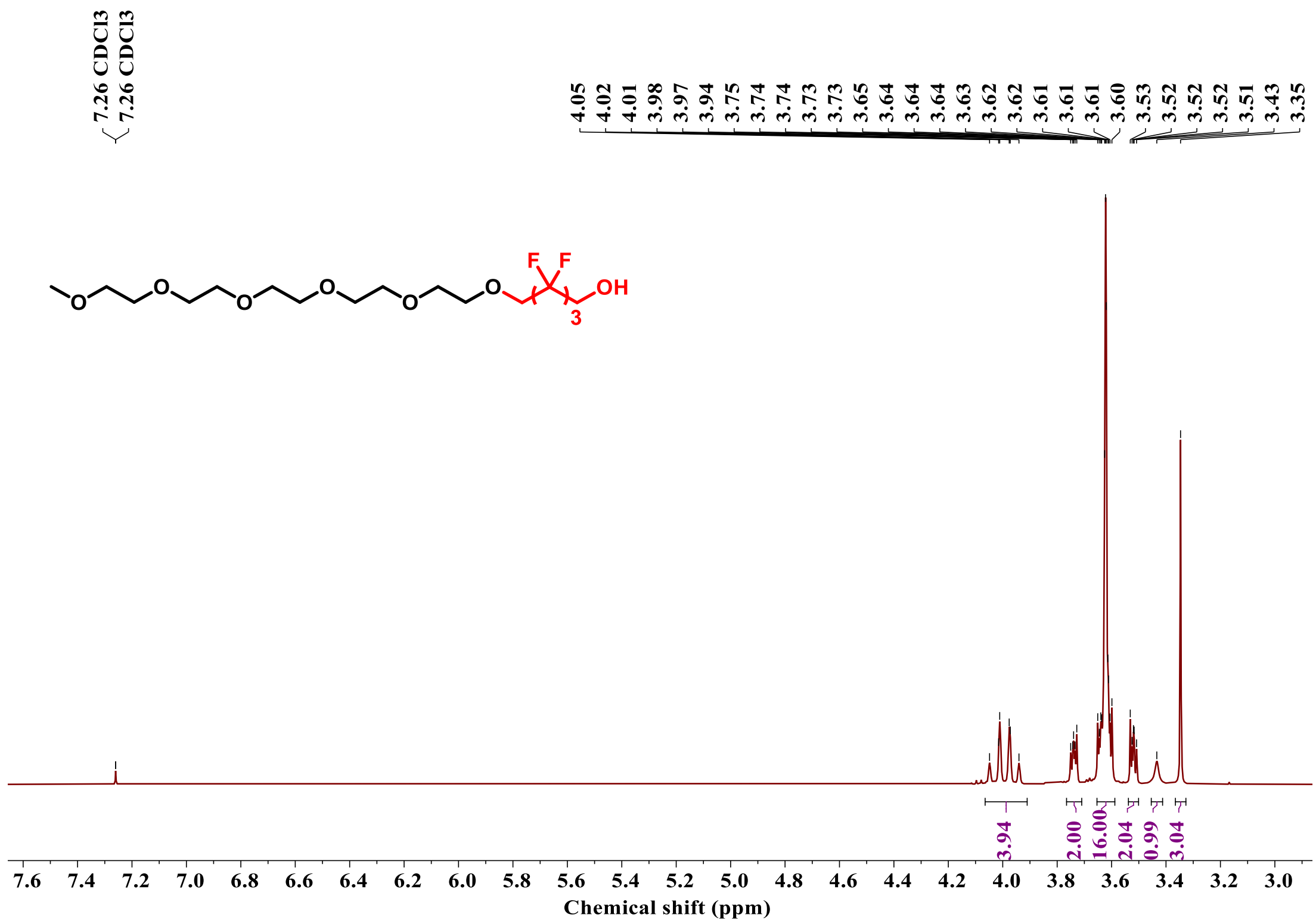

Figure S41. ^{1}H NMR spectrum of compound A5-3.

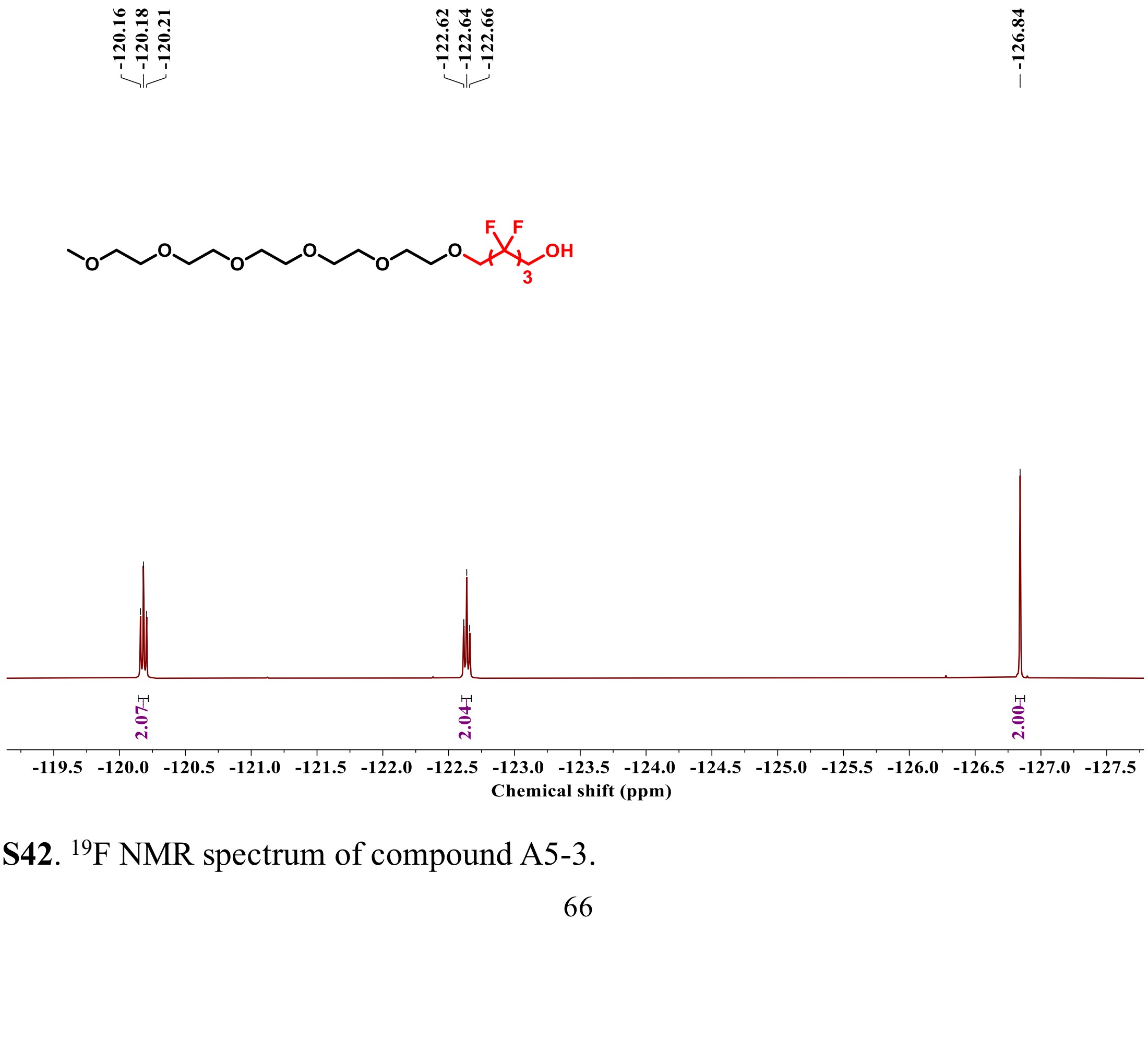

Figure S42. ^{19}F NMR spectrum of compound A5-3.

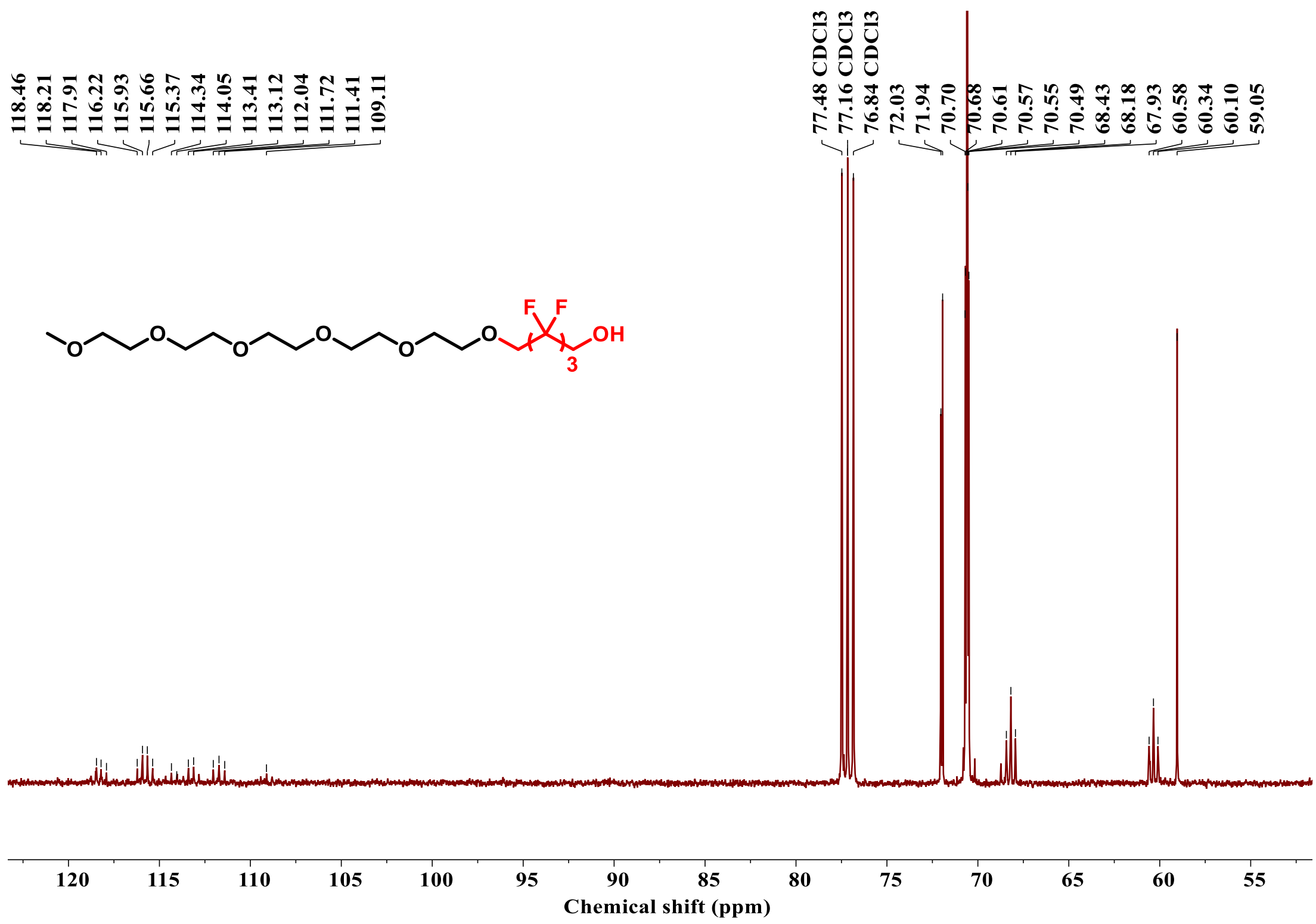

Figure S43. ^{13}C NMR spectrum of compound A5-3.

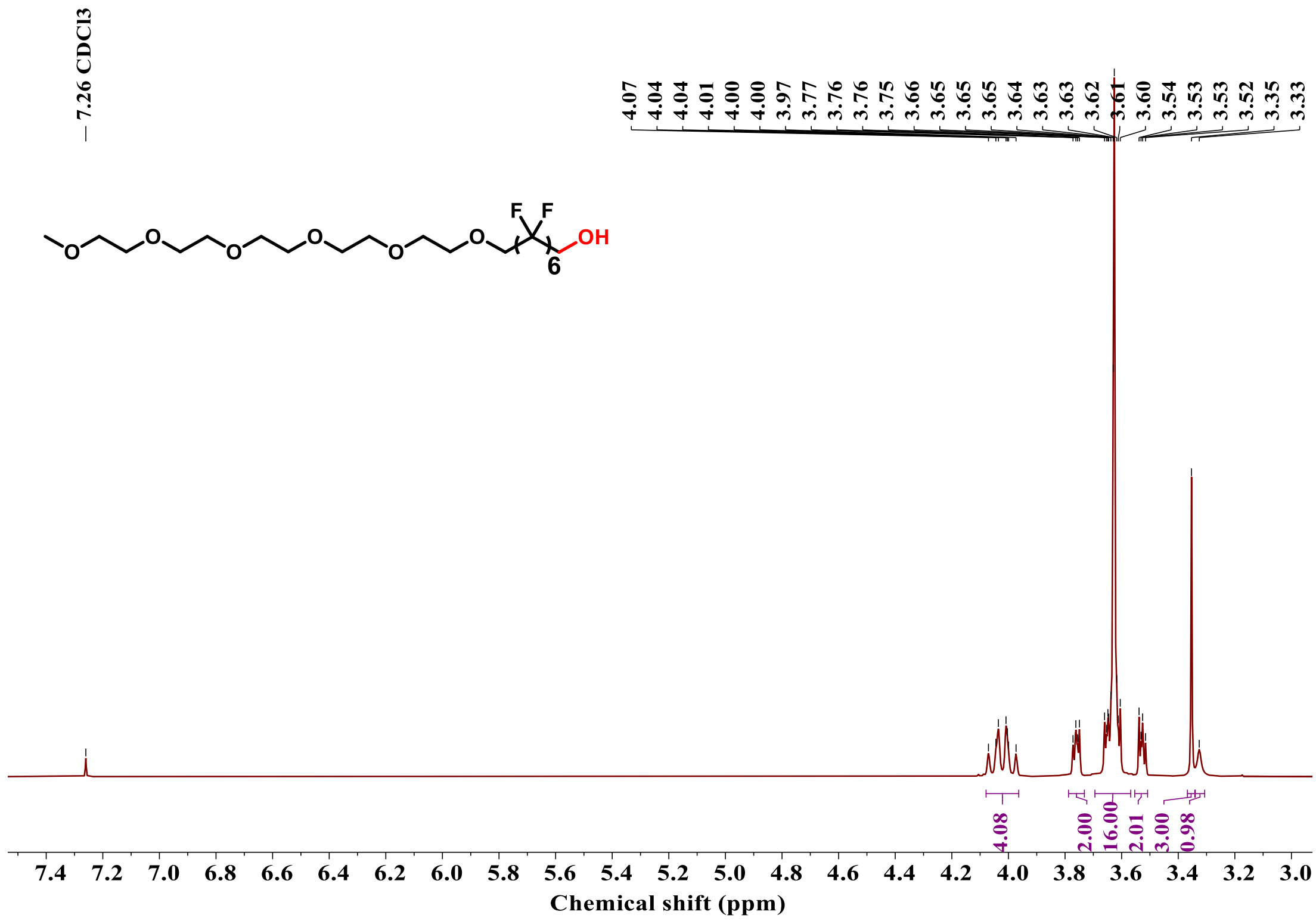

Figure S44. ^{1}H NMR spectrum of compound A5-6.

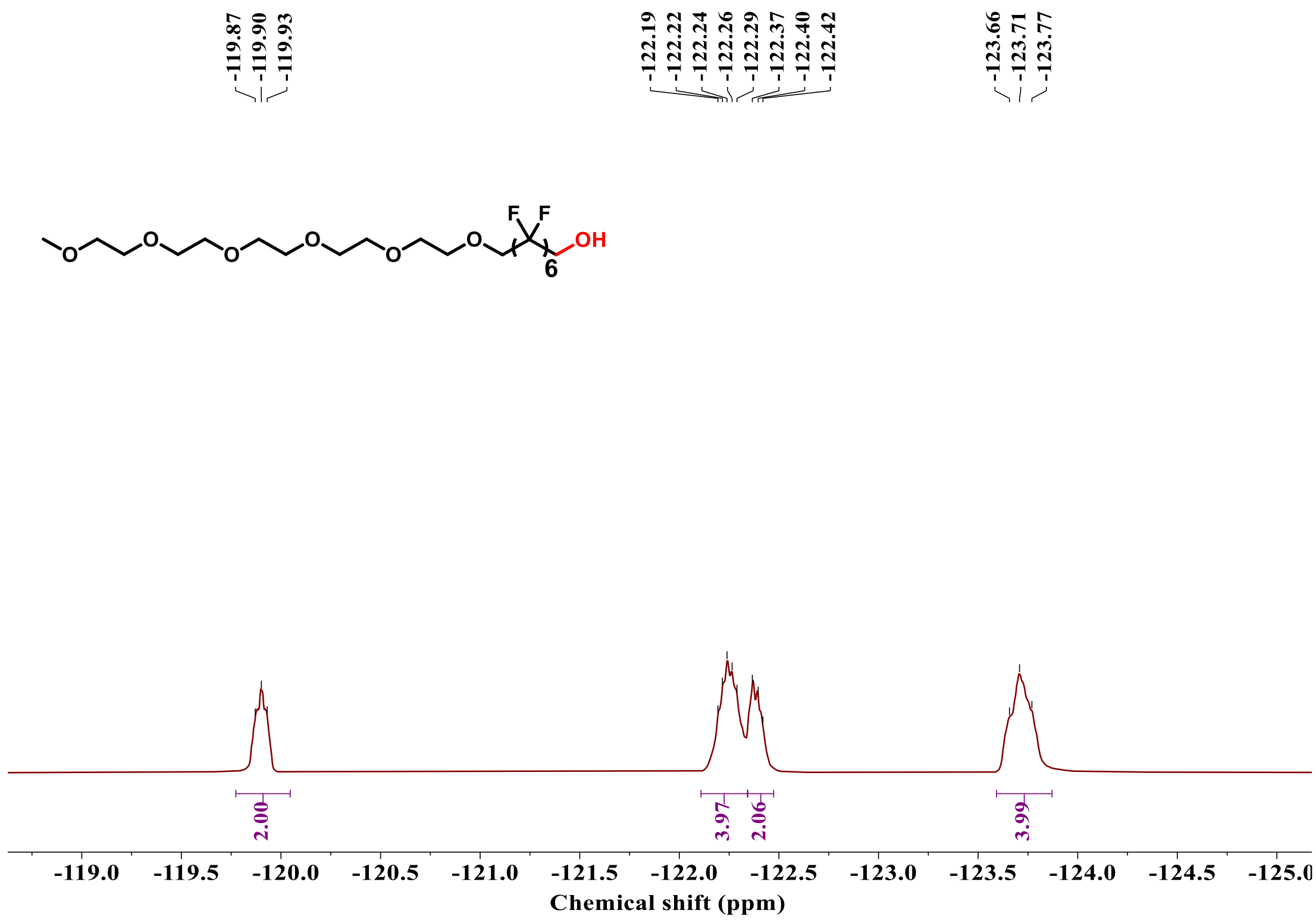

Figure S45. ^{19}F NMR spectrum of compound A5-6.

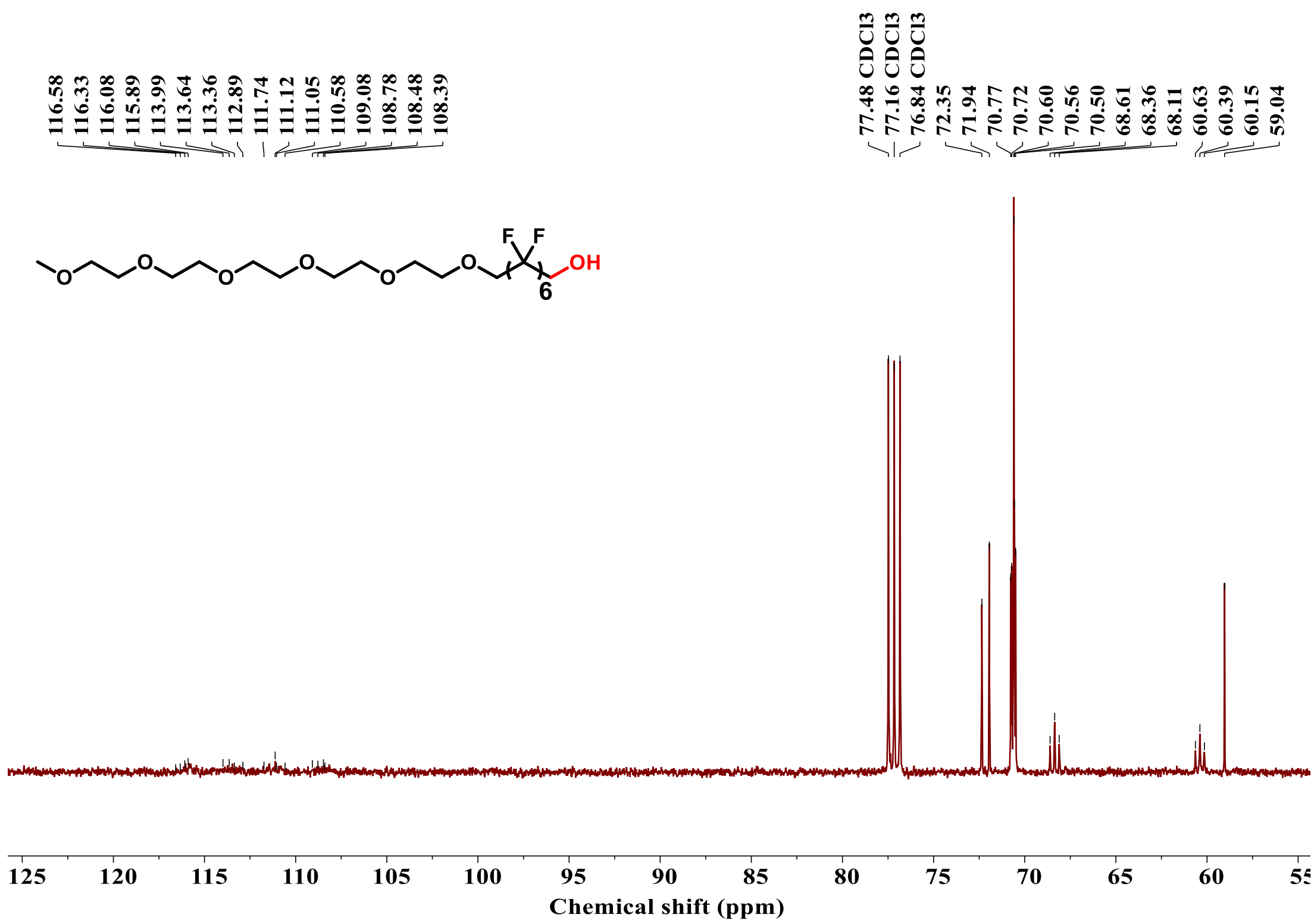

Figure S46. ^{13}C NMR spectrum of compound A5-6.

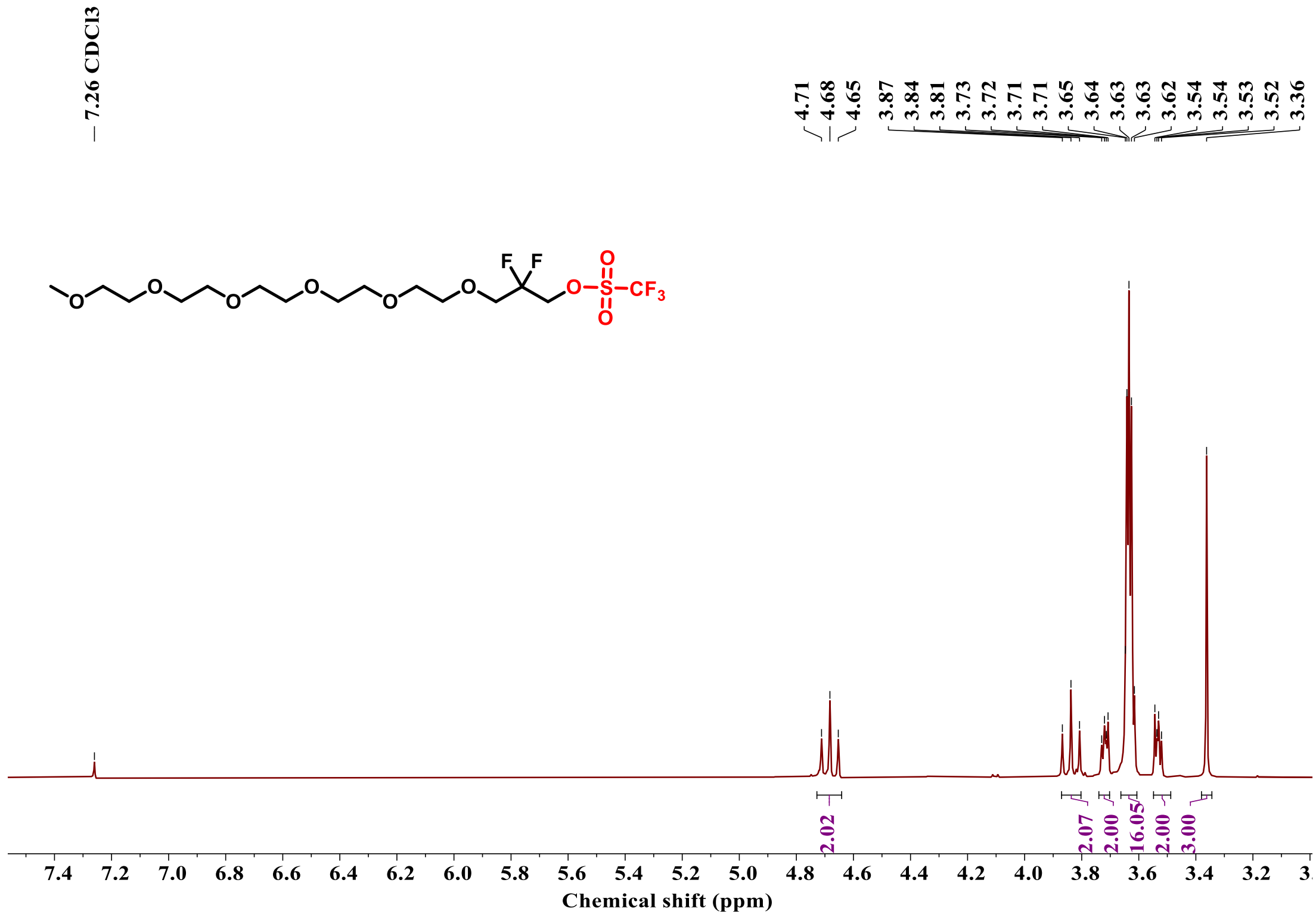

Figure S47. ^{1}H NMR spectrum of compound A7-1.

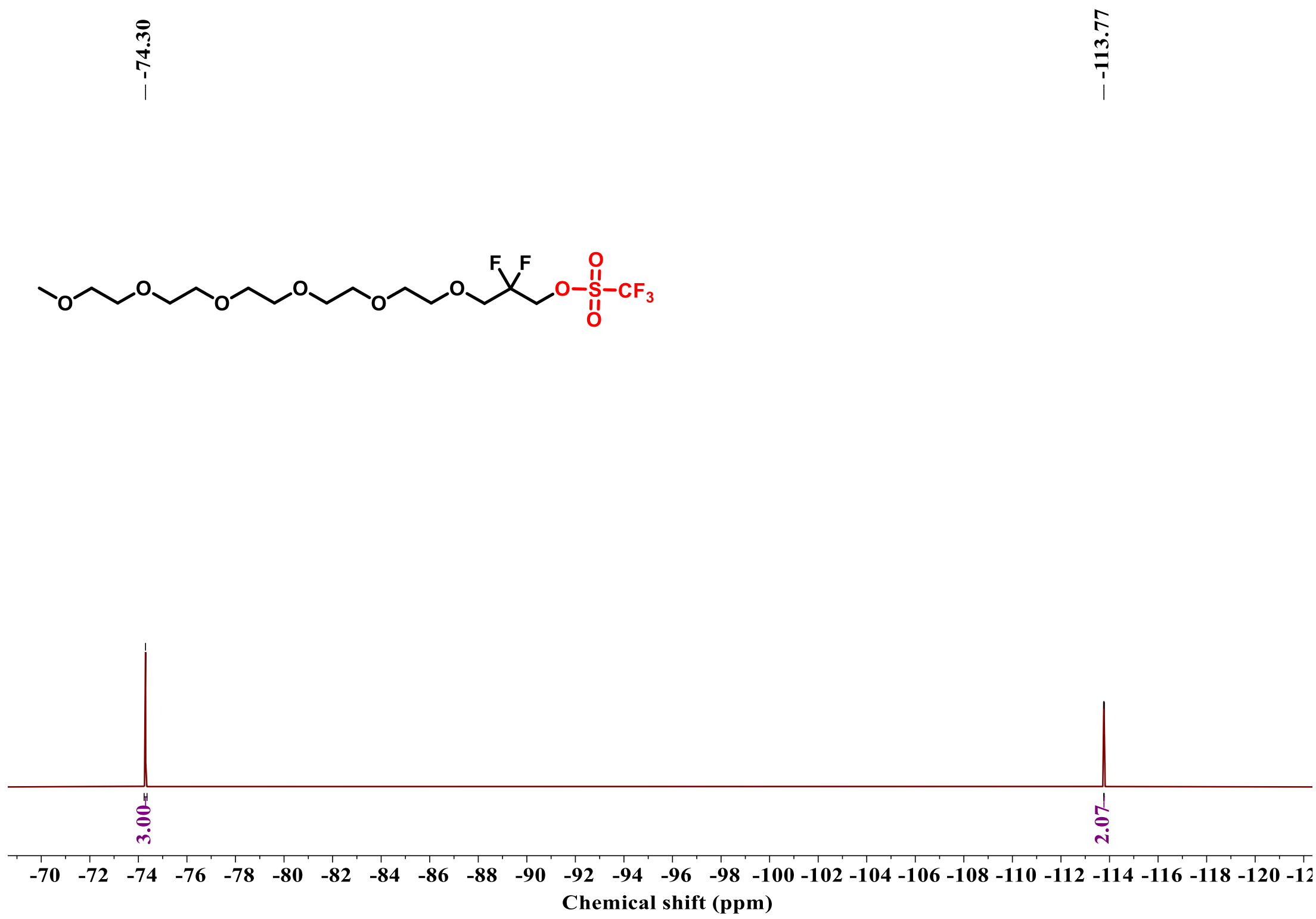

Figure S48. ^{19}F NMR spectrum of compound A7-1.

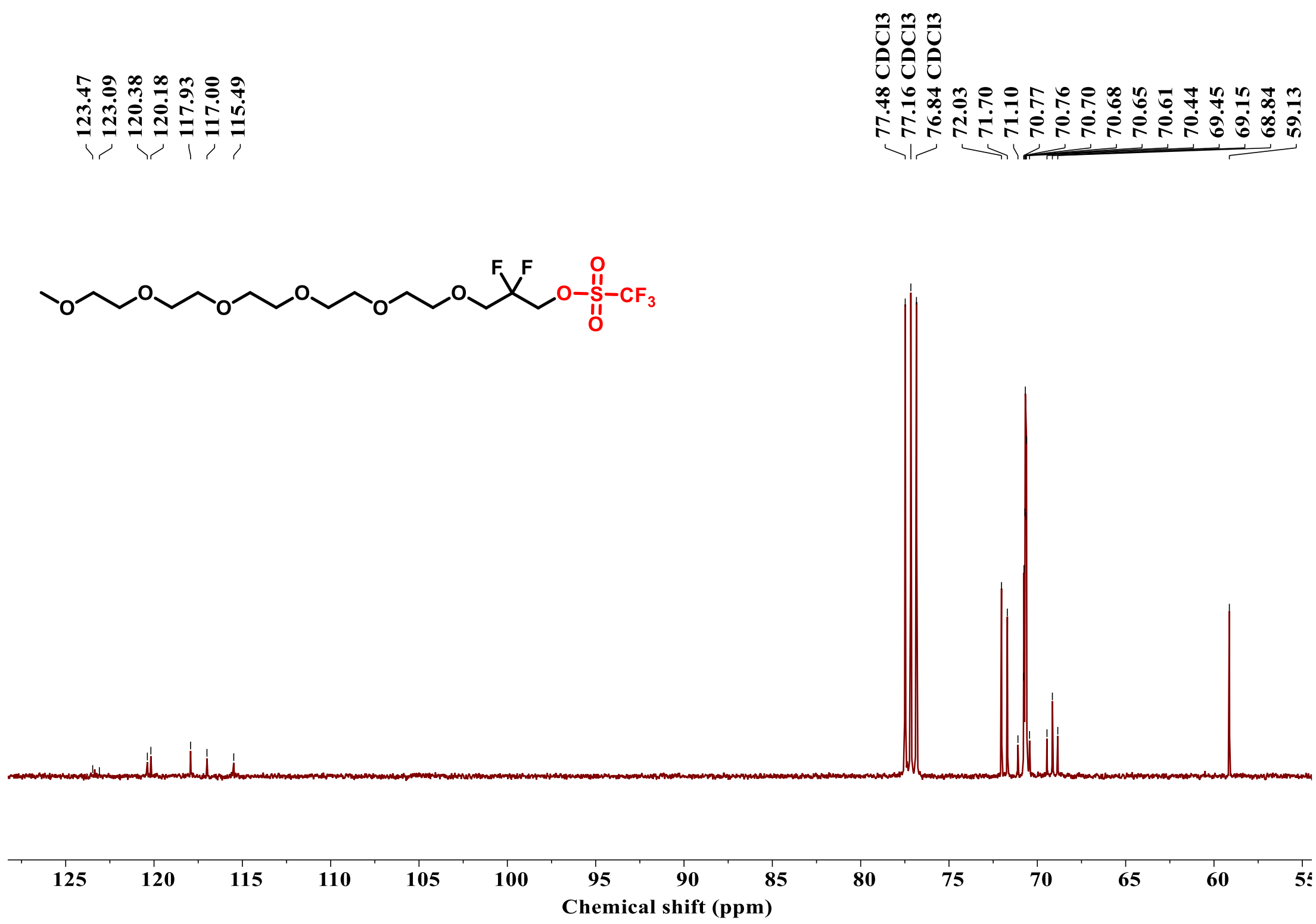

Figure S49. ^{13}C NMR spectrum of compound A7-1.

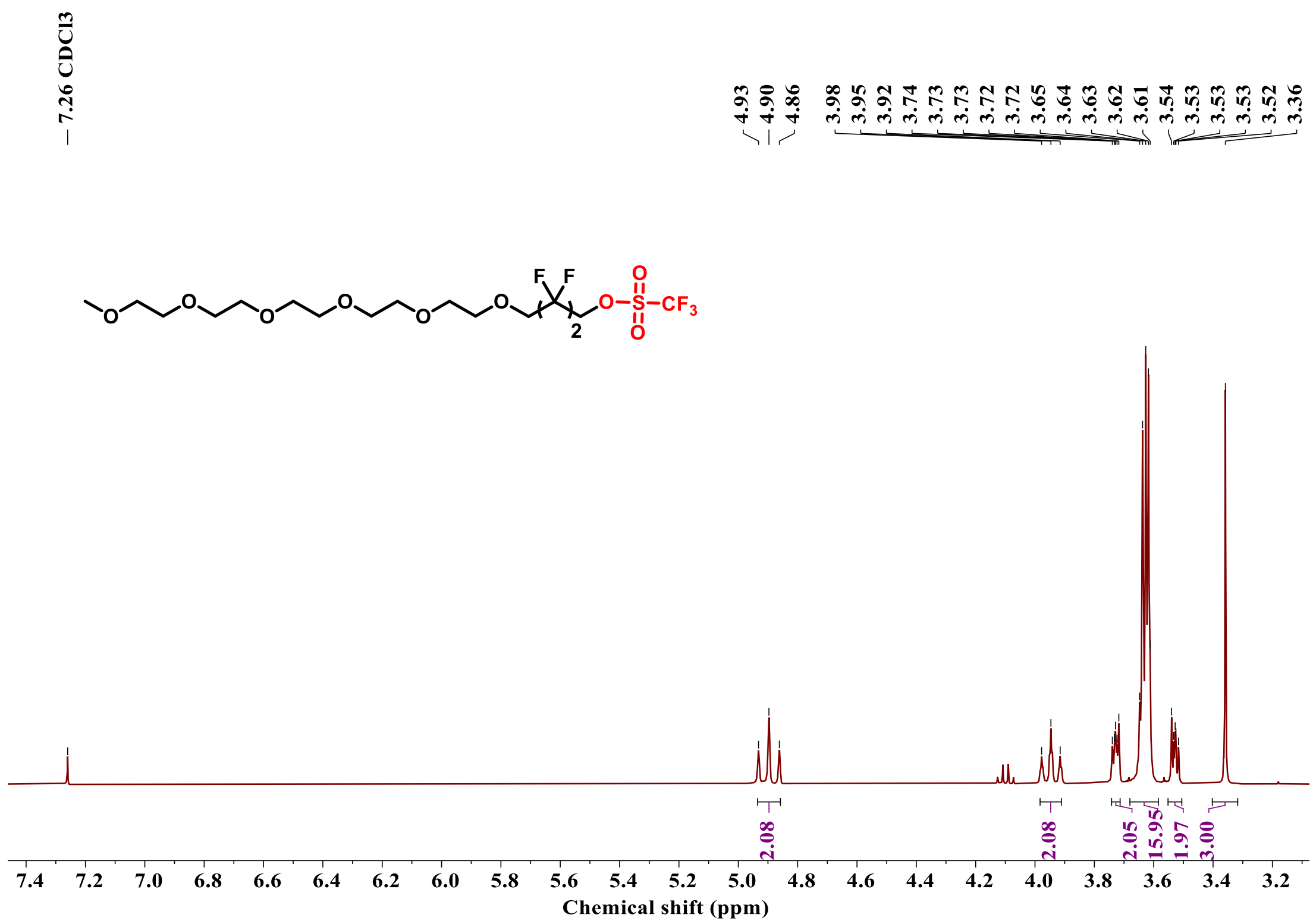

Figure S50. ^{1}H NMR spectrum of compound A7-2.

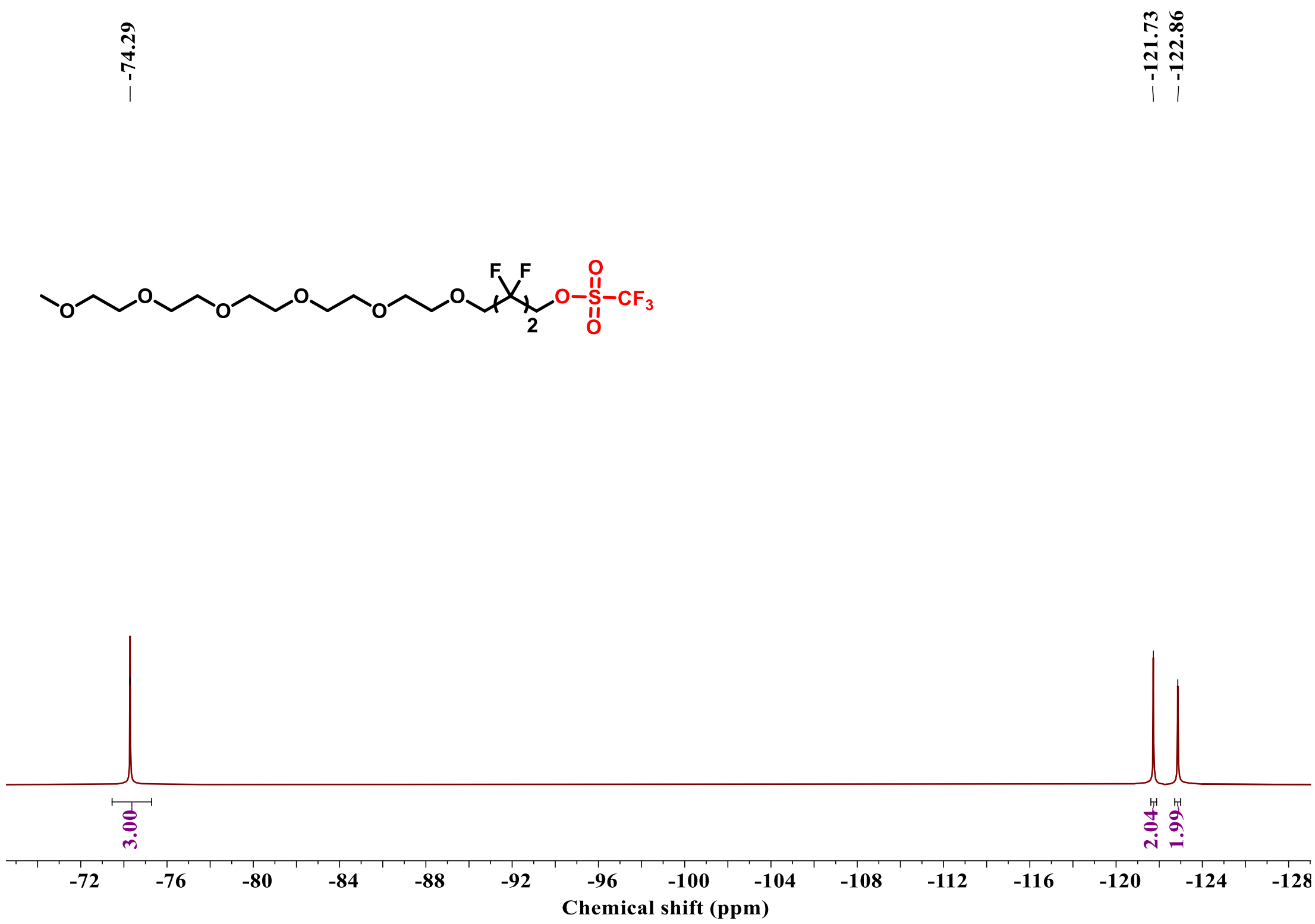

Figure S51. ^{19}F NMR spectrum of compound A7-2.

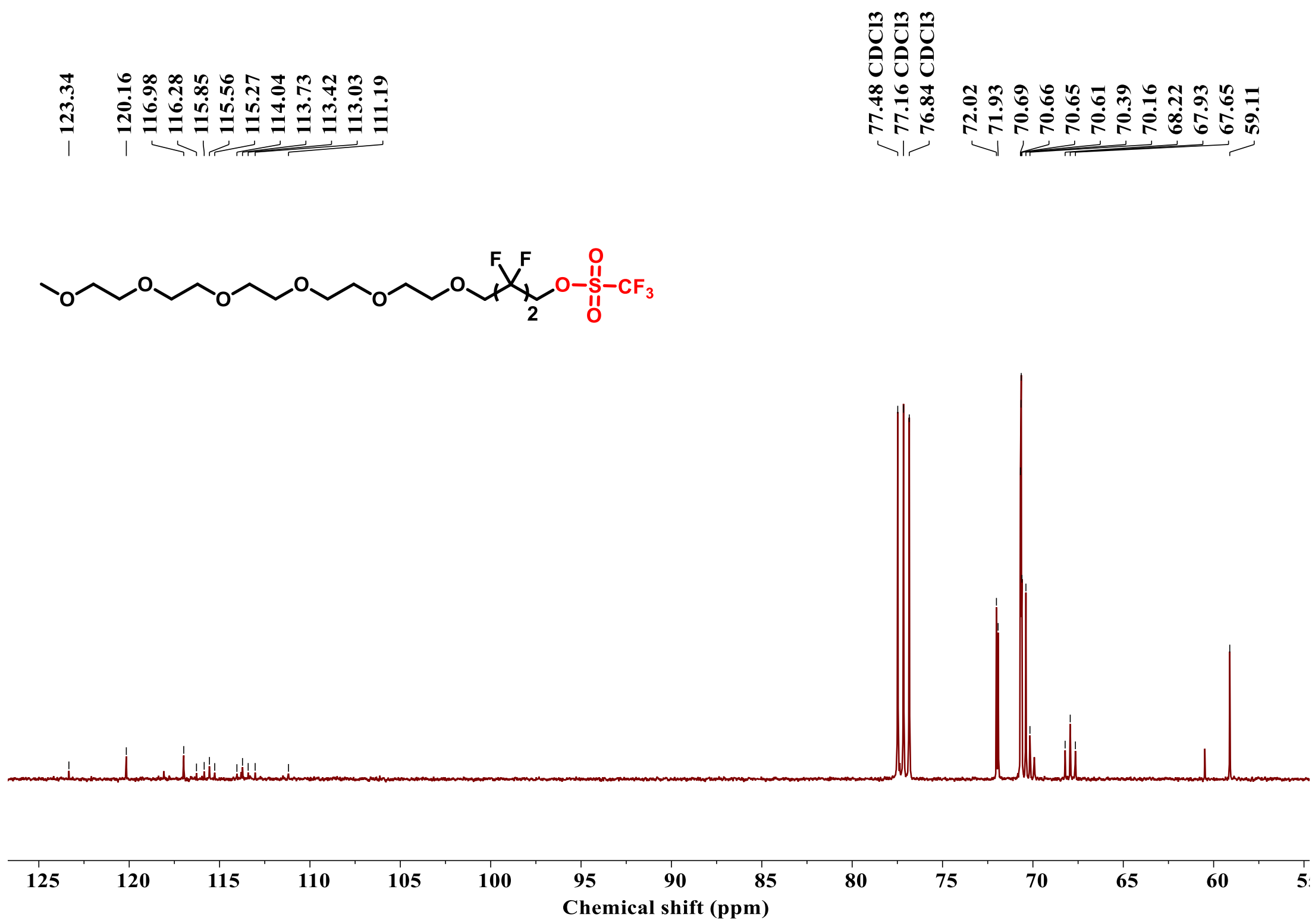

Figure S52. ^{13}C NMR spectrum of compound A7-2.

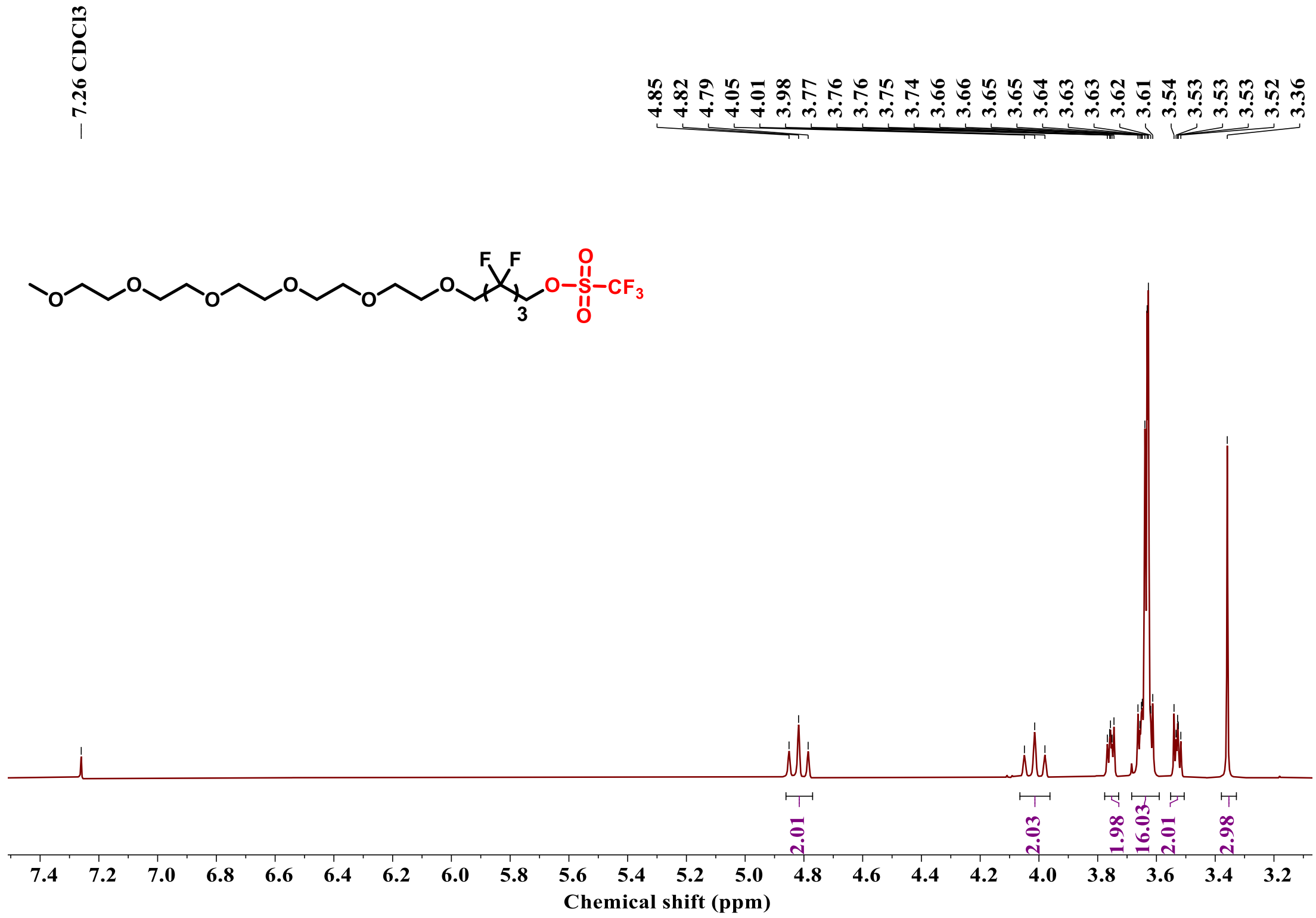

Figure S53. ¹H NMR spectrum of compound A7-3.

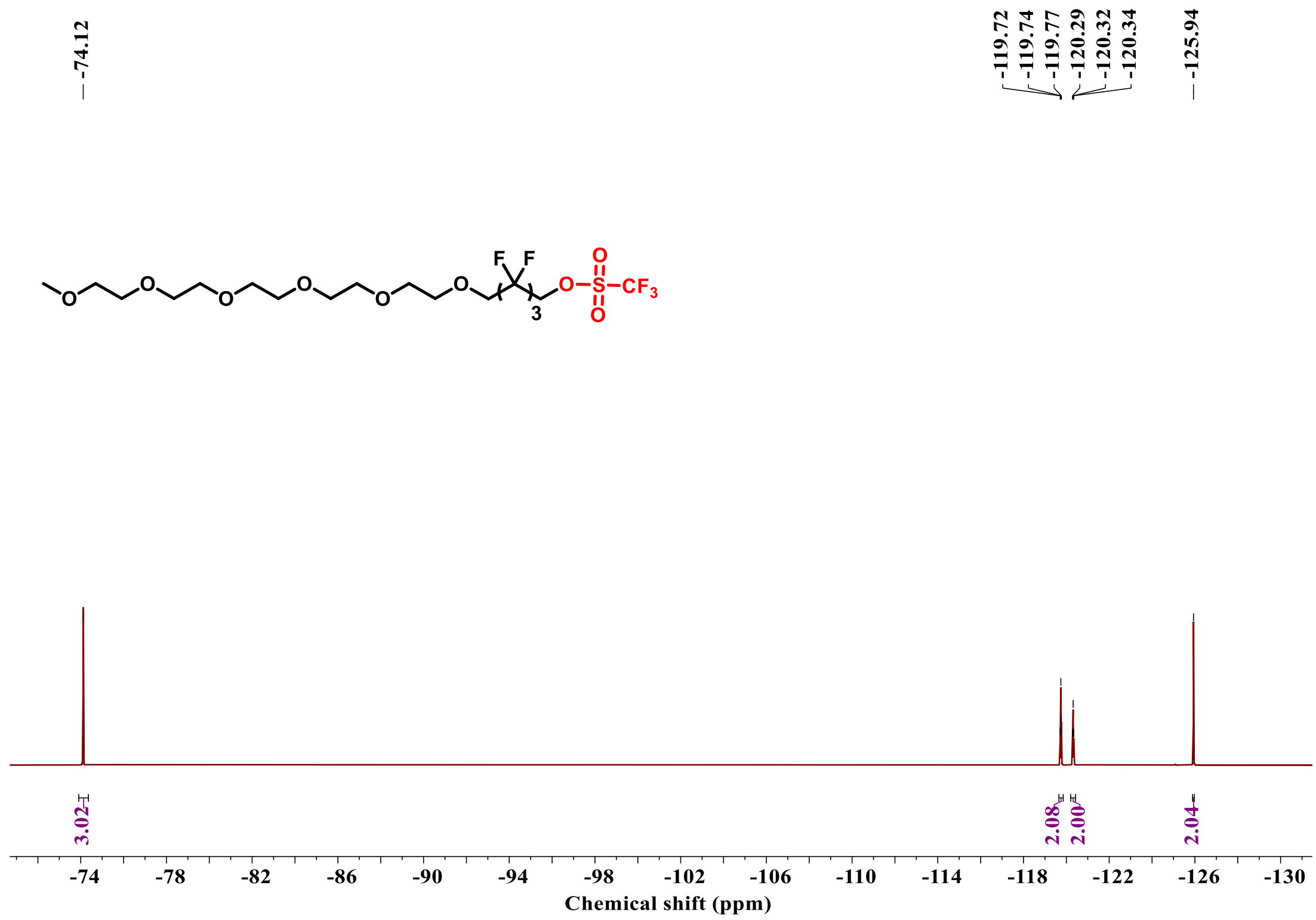

Figure S54. ¹⁹F NMR spectrum of compound A7-3.

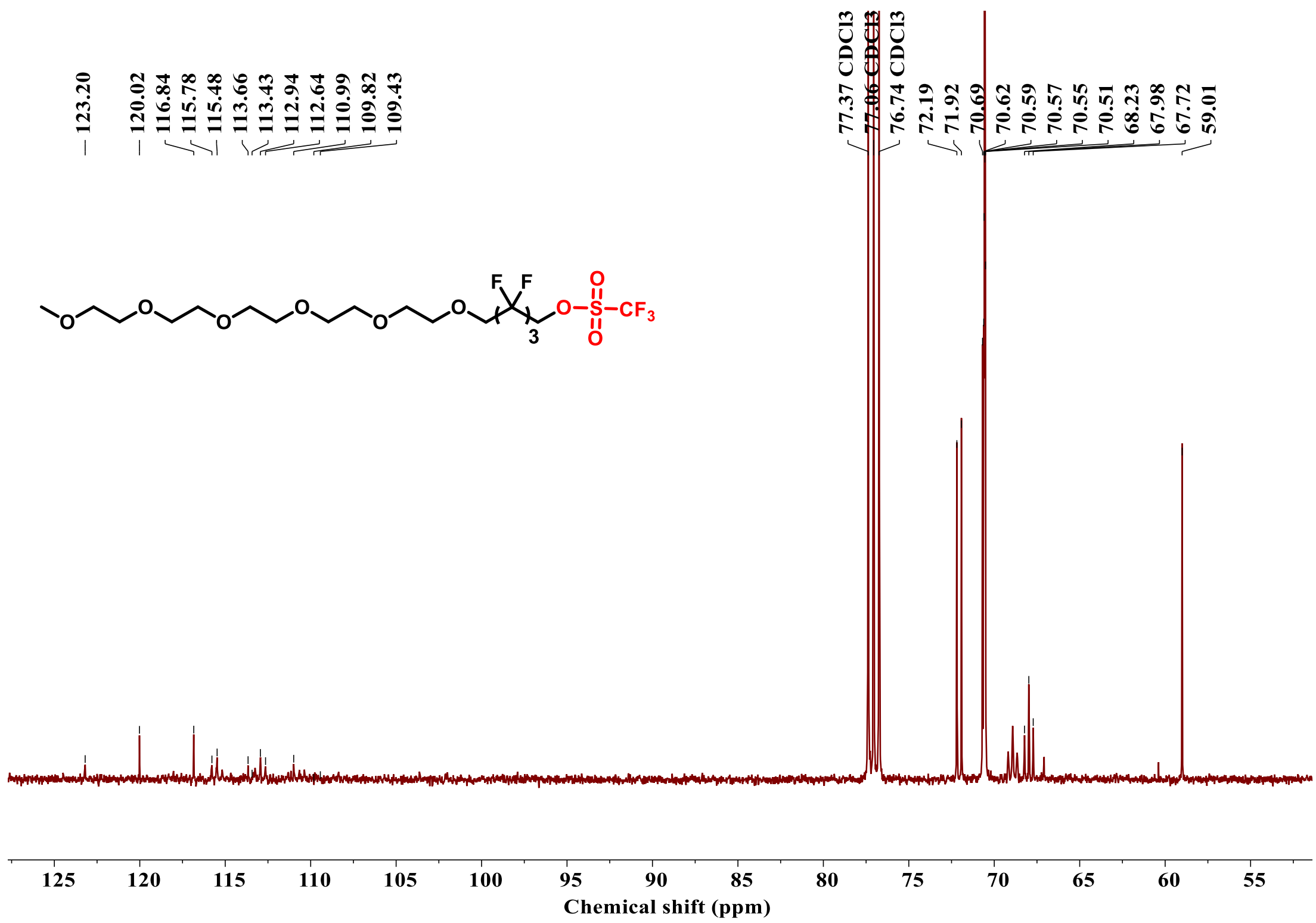

Figure S55. ^{13}C NMR spectrum of compound A7-3.

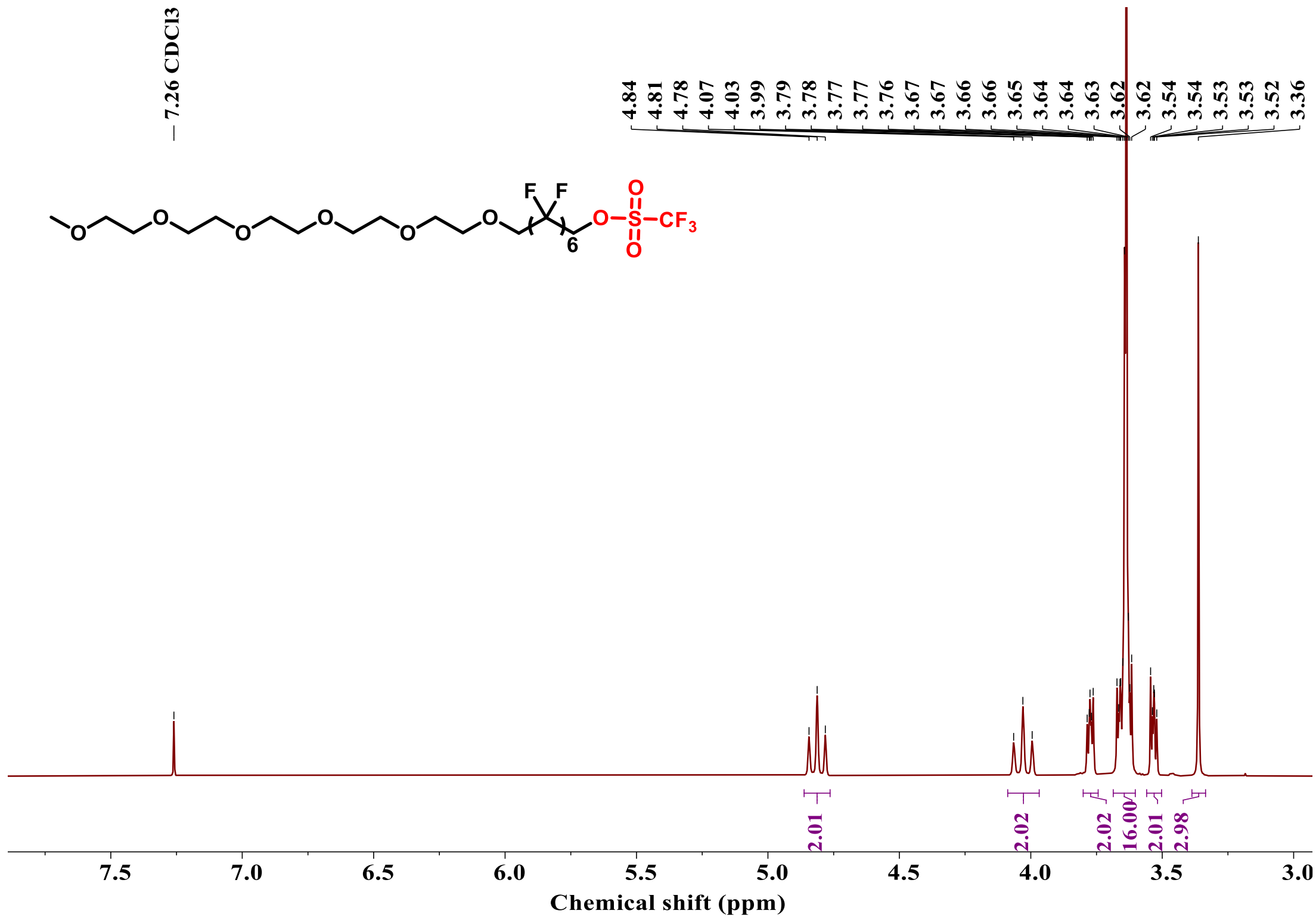

Figure S56. ^{1}H NMR spectrum of compound A7-6.

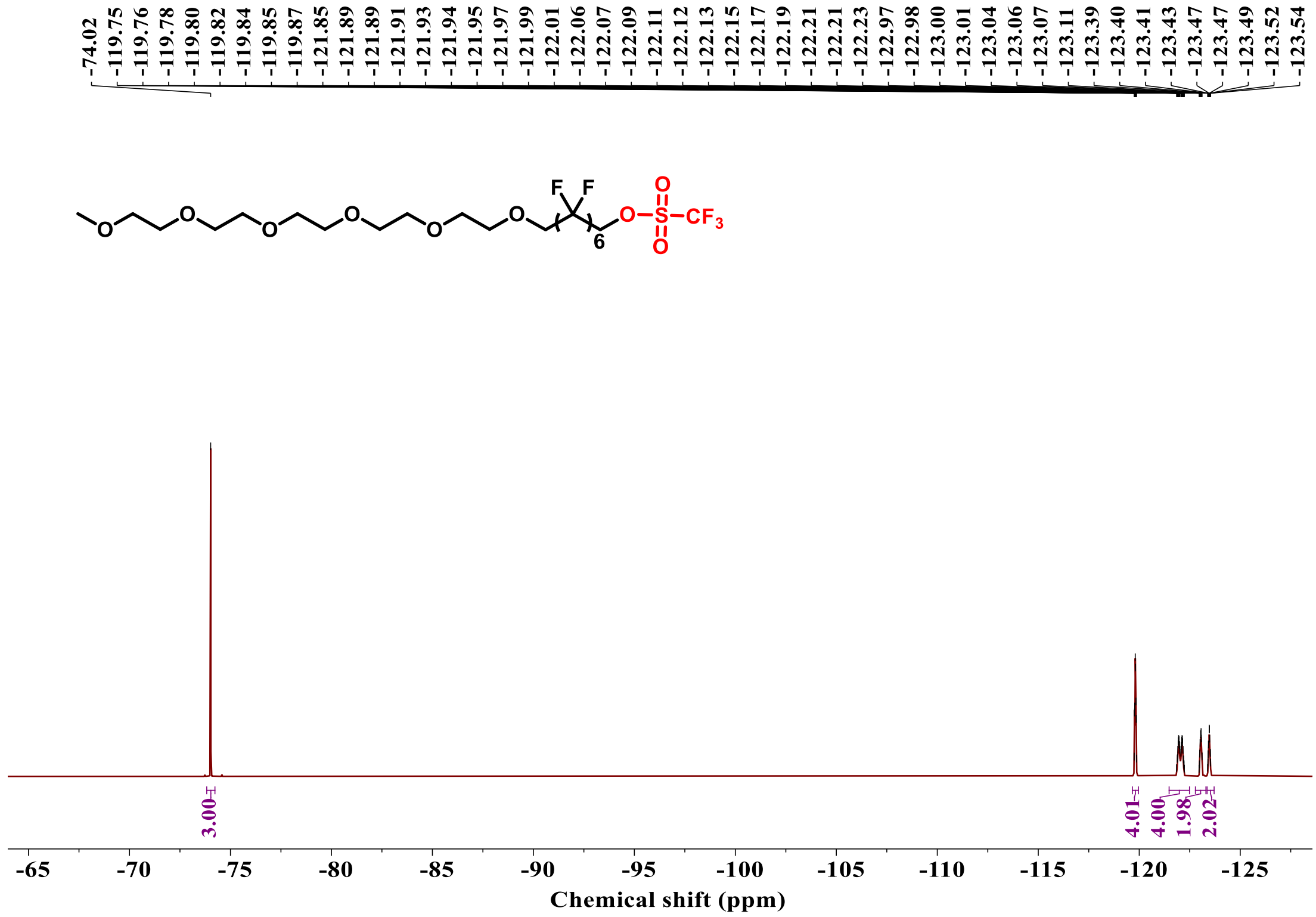

Figure S57. ^{19}F NMR spectrum of compound A7-6.

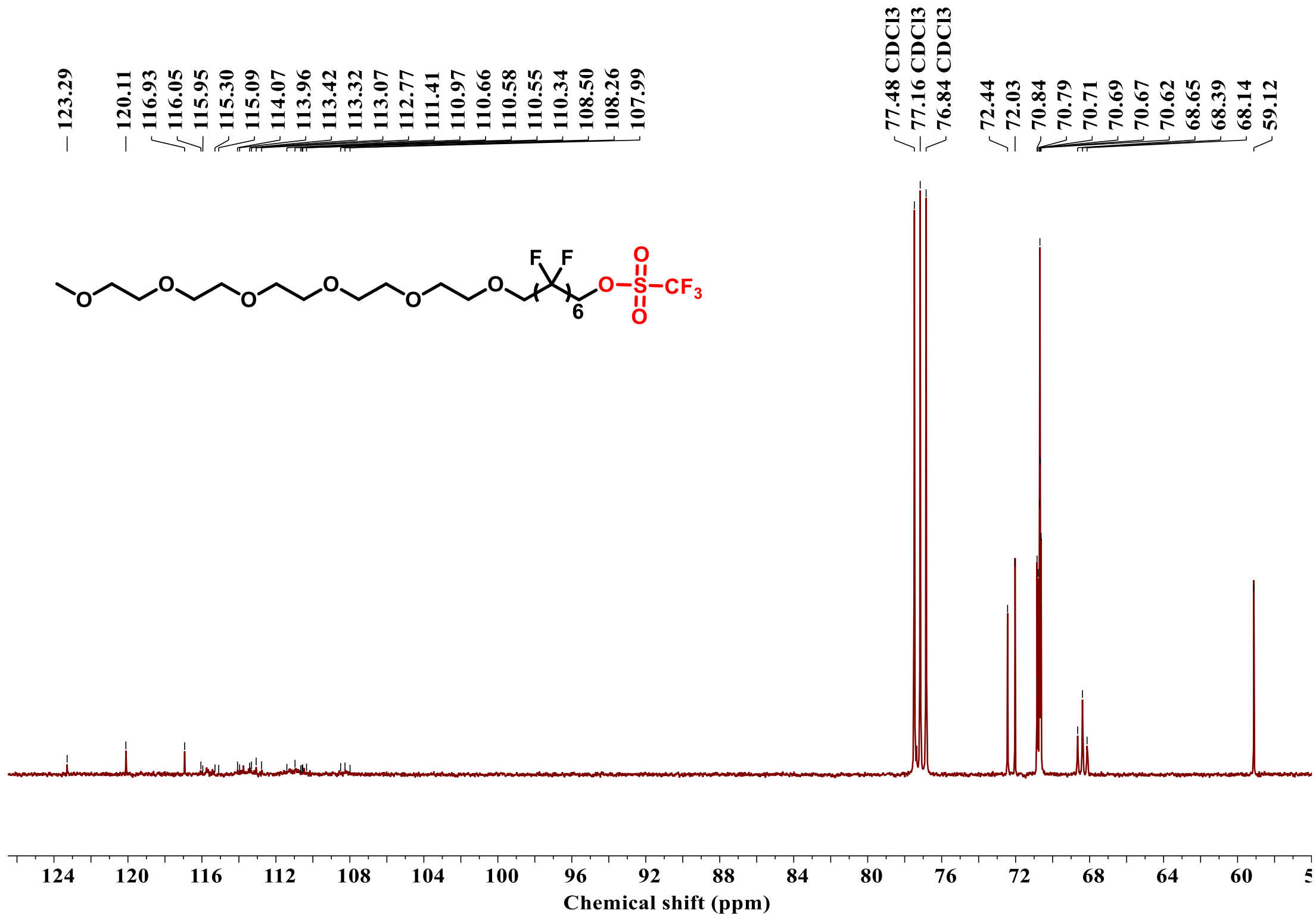

Figure S58. ^{13}C NMR spectrum of compound A7-6.

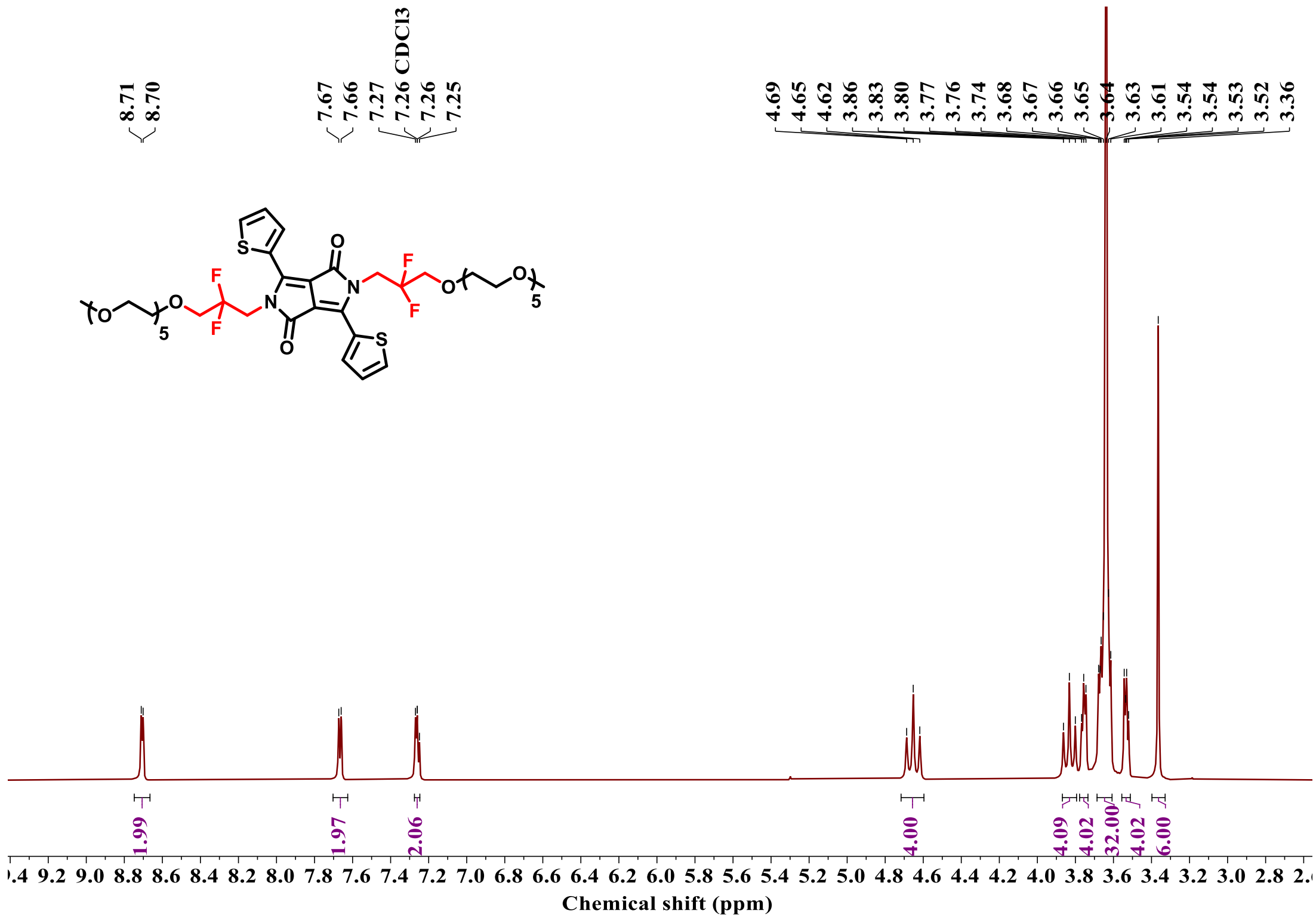

Figure S59. ^{1}H NMR spectrum of compound A9-1.

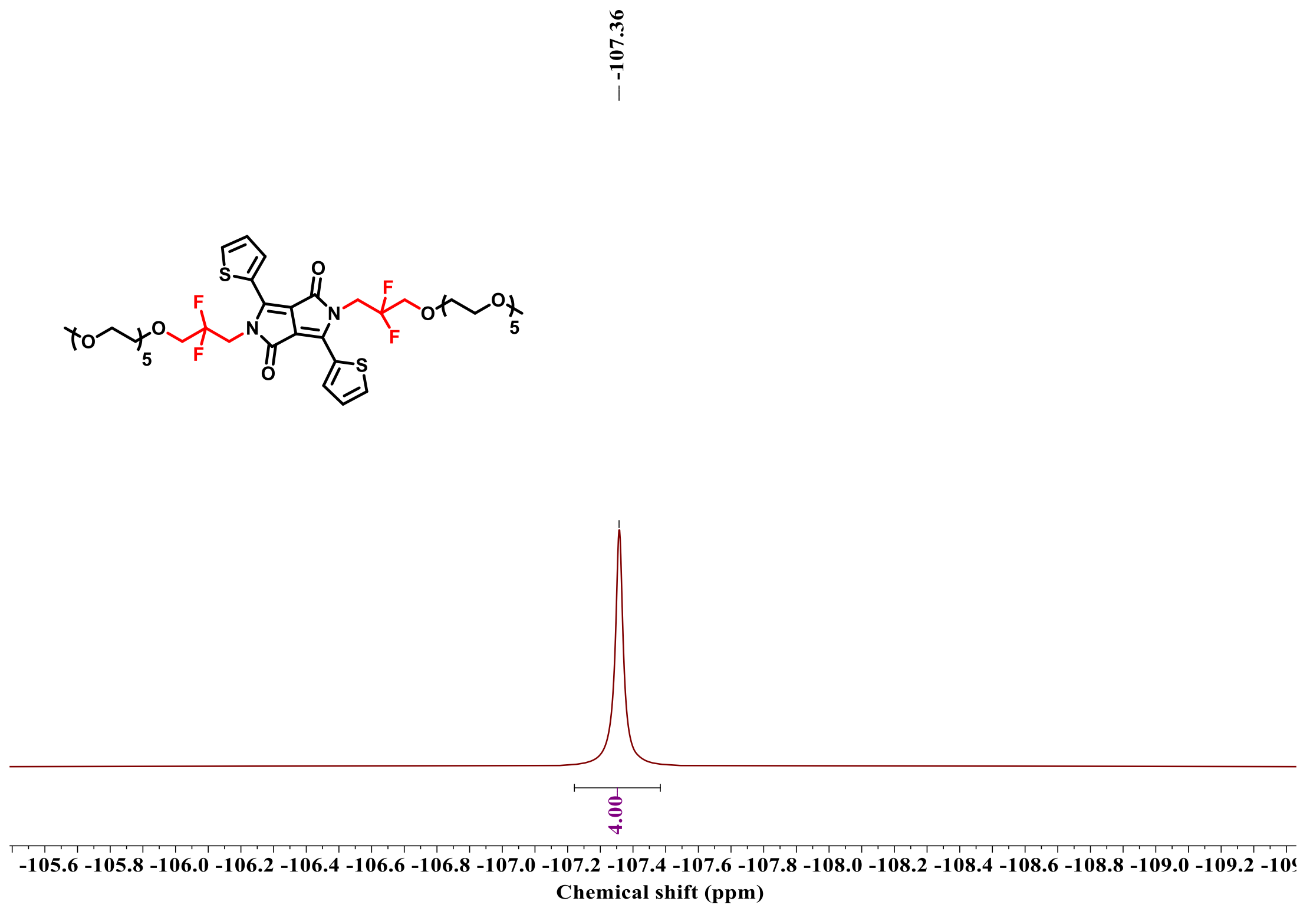

Figure S60. ^{19}F NMR spectrum of compound A9-1.

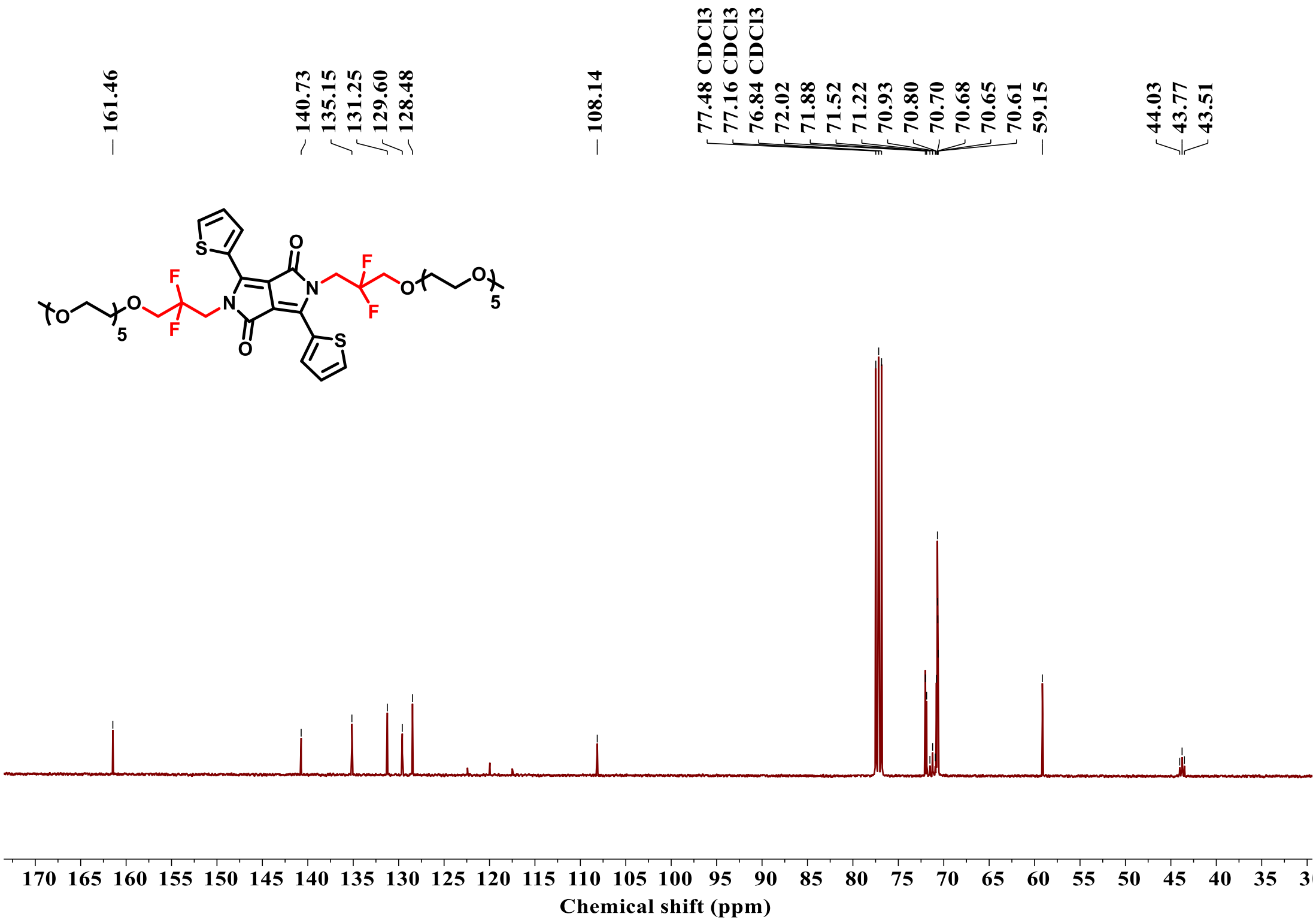

Figure S61. ^{13}C NMR spectrum of compound A9-1.

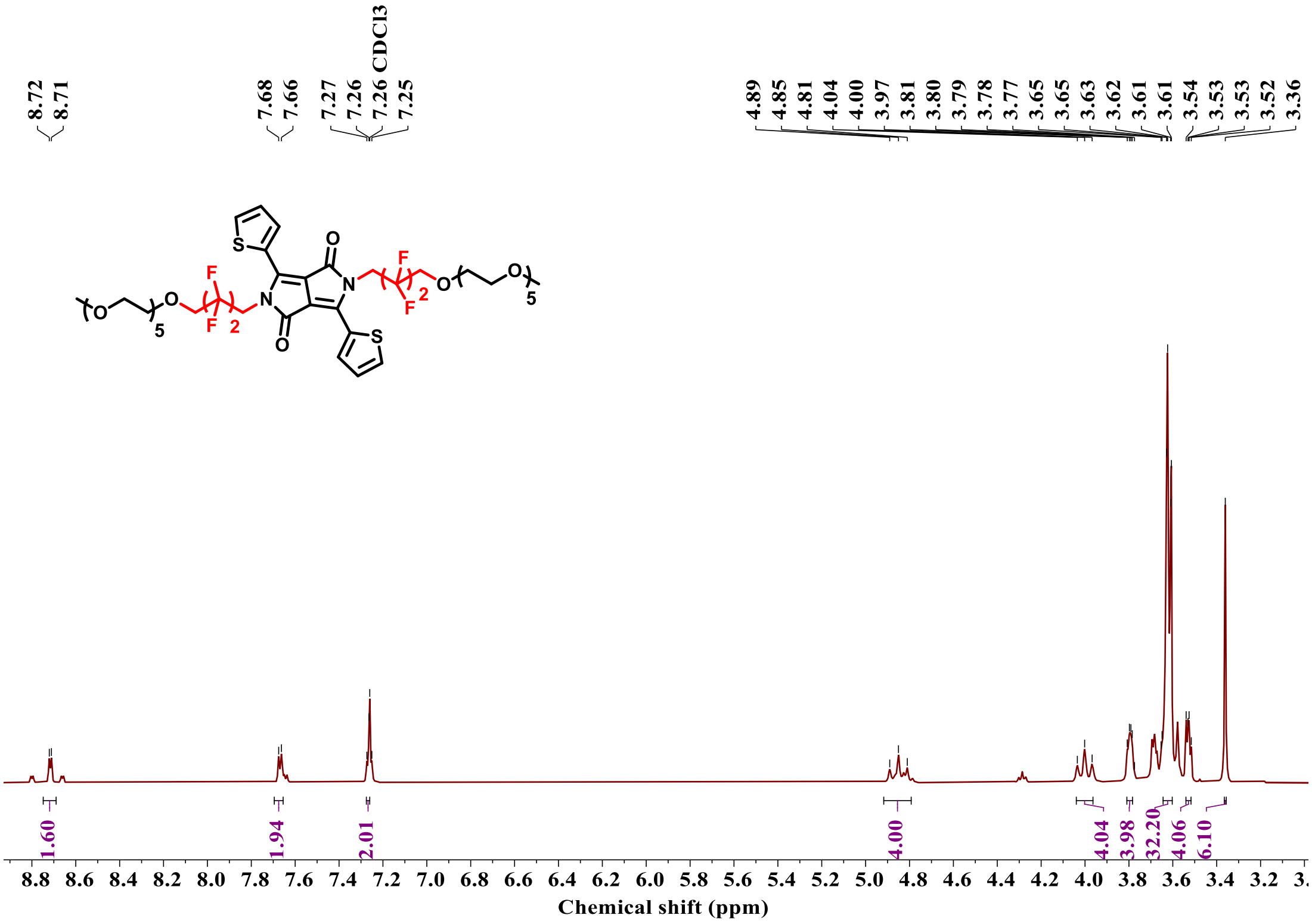

Figure S62. ^{1}H NMR spectrum of compound A9-2.

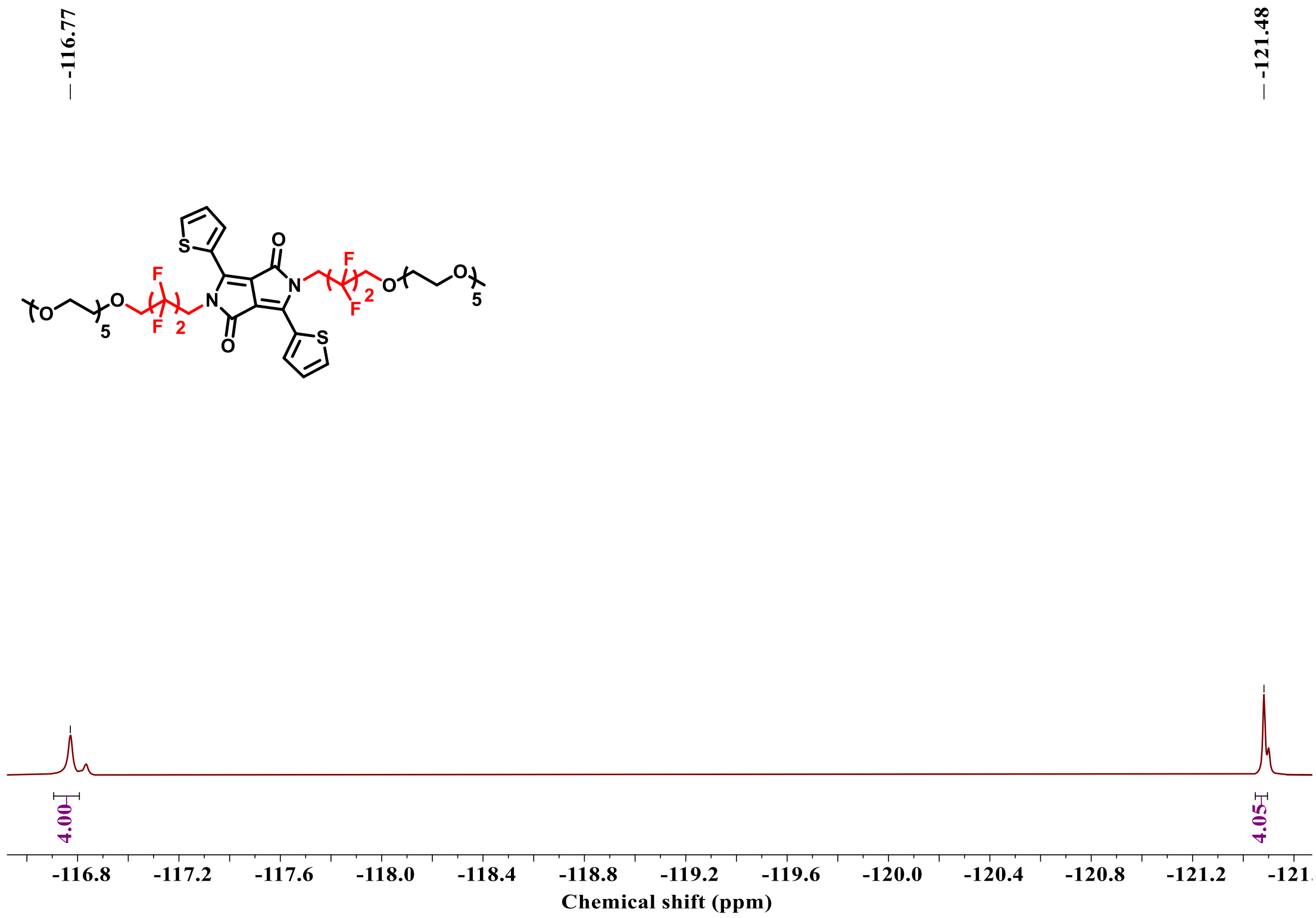

Figure S63. ^{19}F NMR spectrum of compound A9-2.

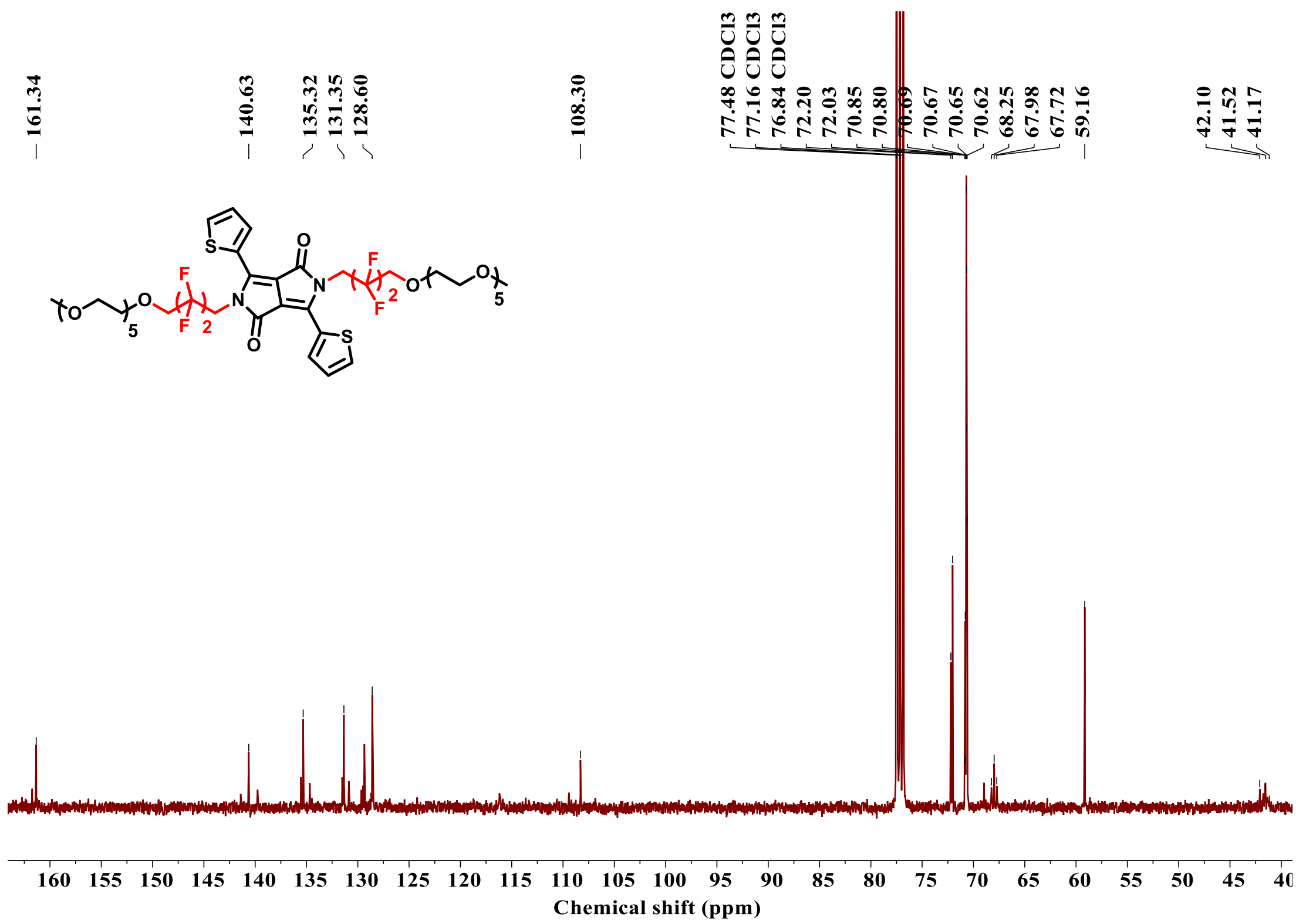

Figure S64. ^{13}C NMR spectrum of compound A9-2.

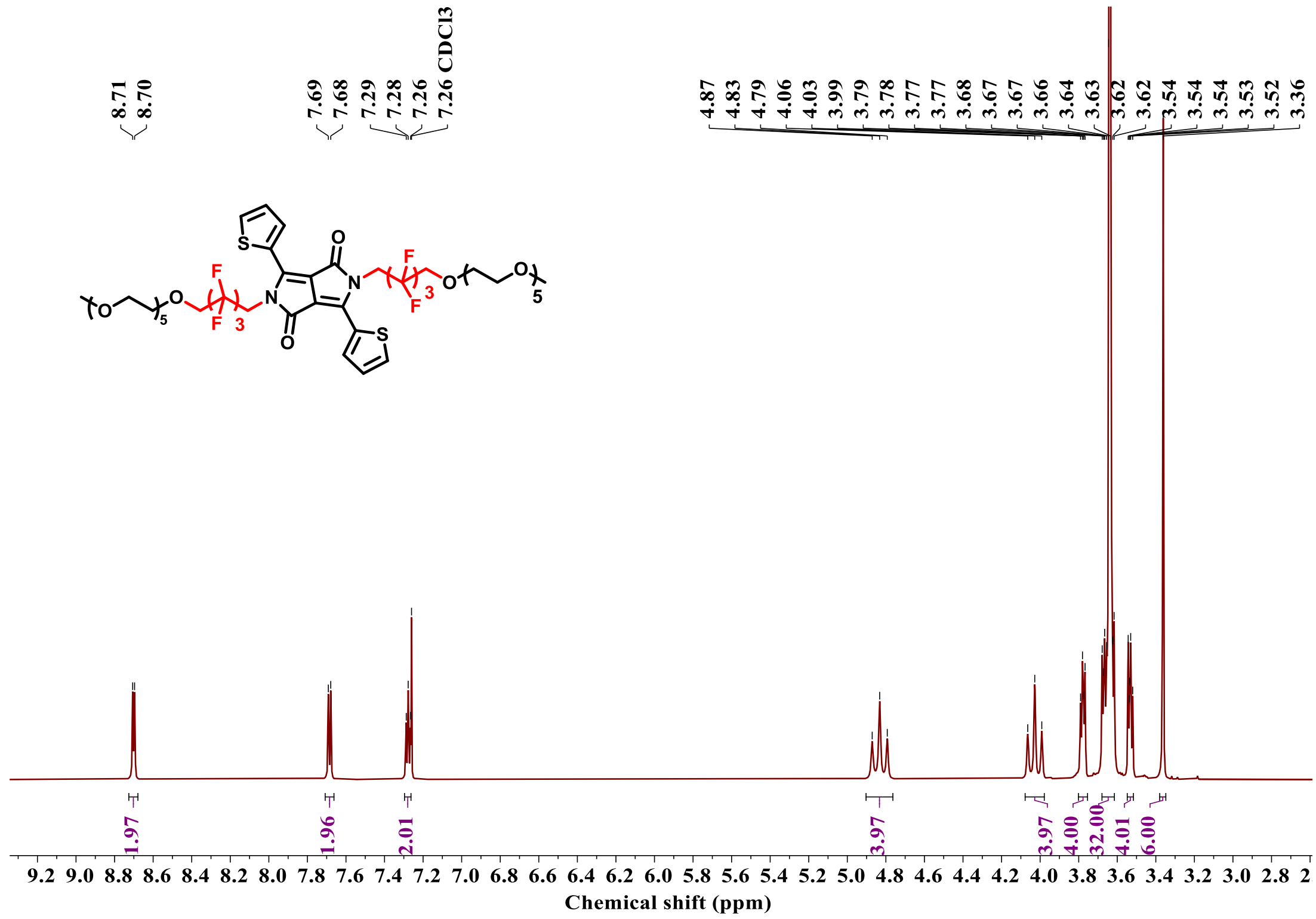

Figure S65. ^{1}H NMR spectrum of compound A9-3.

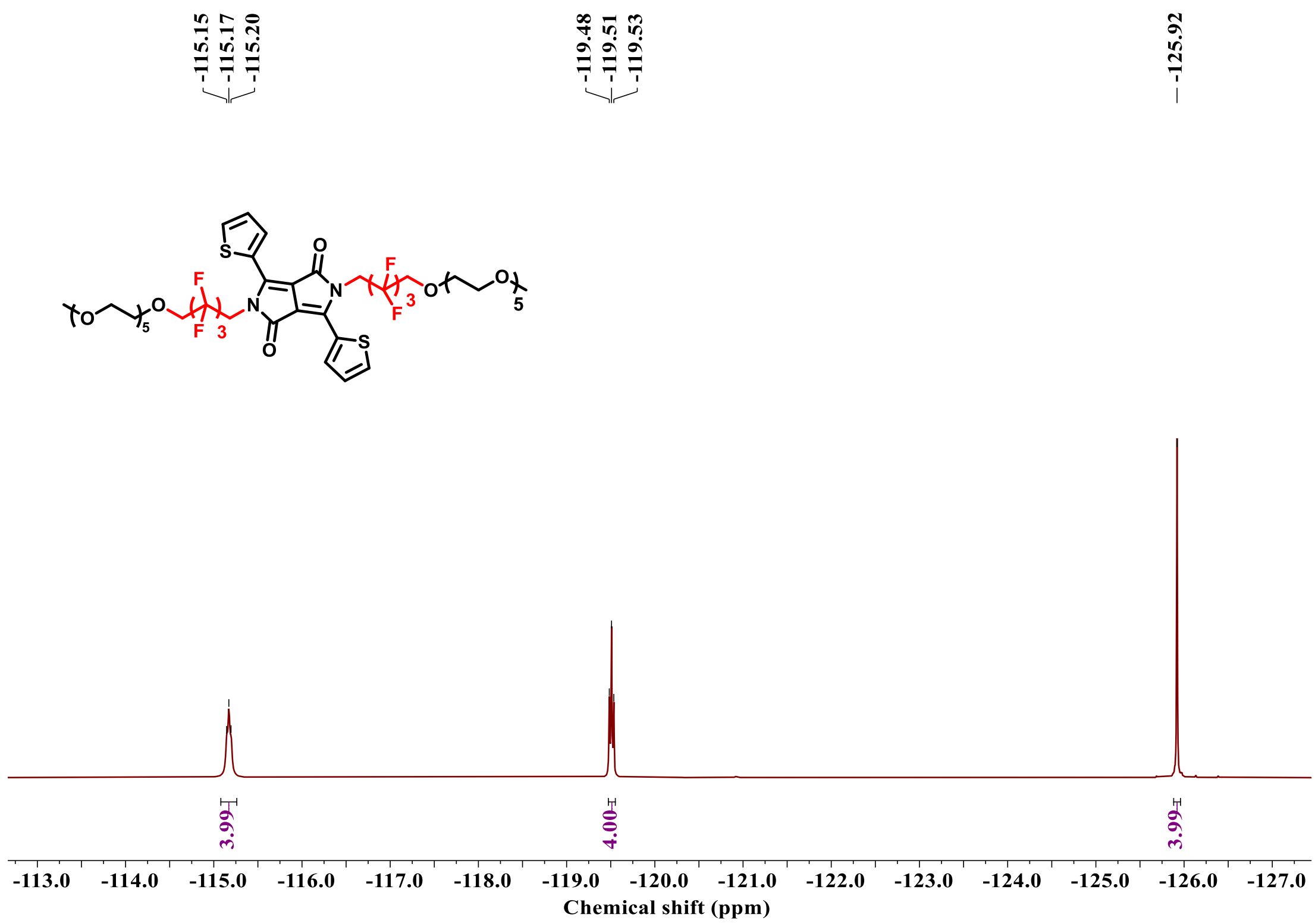

Figure S66. ^{19}F NMR spectrum of compound A9-3.

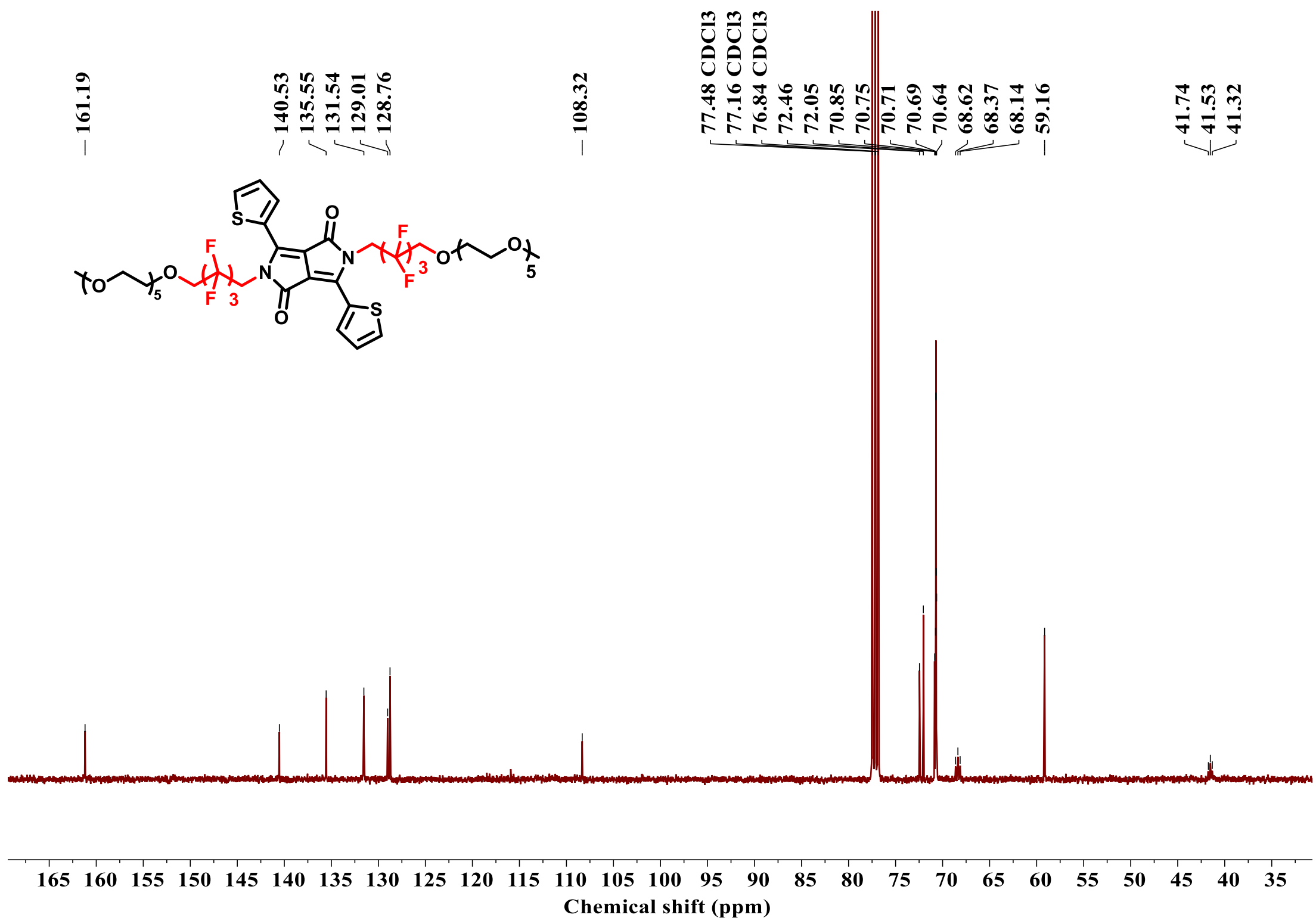

Figure S67. ^{13}C NMR spectrum of compound A9-3.

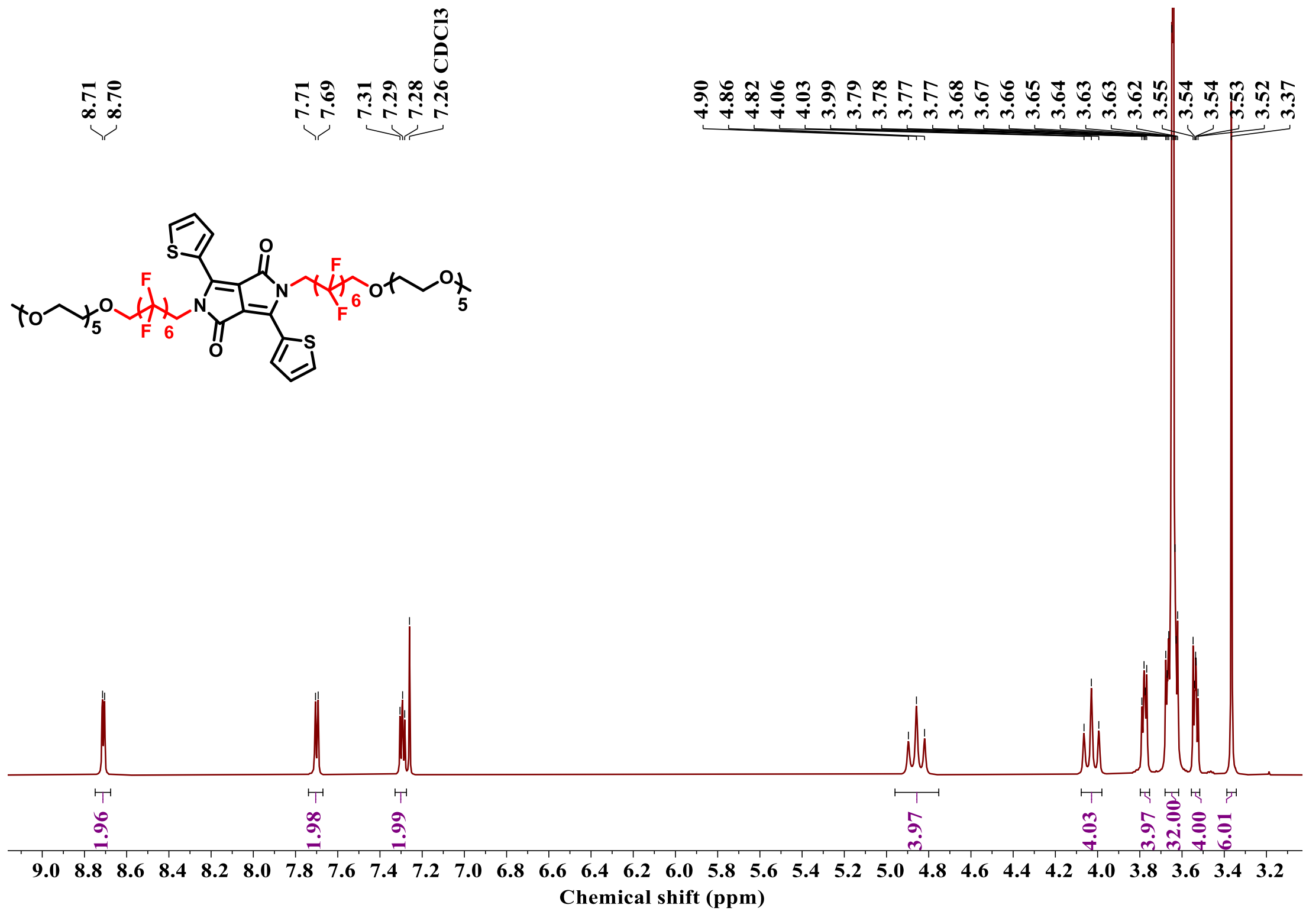

Figure S68. ^{1}H NMR spectrum of compound A9-6.

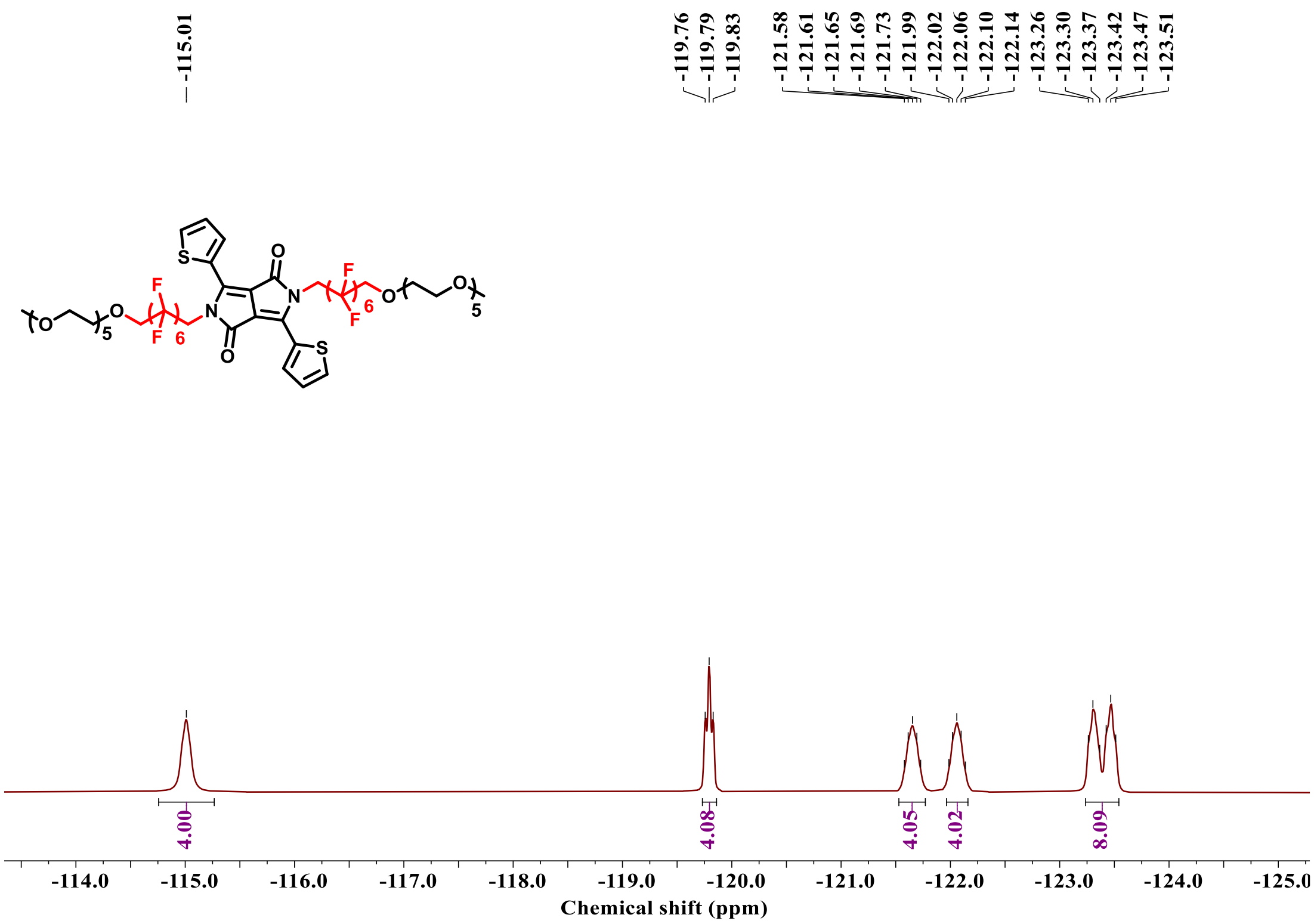

Figure S69. ^{19}F NMR spectrum of compound A9-6.

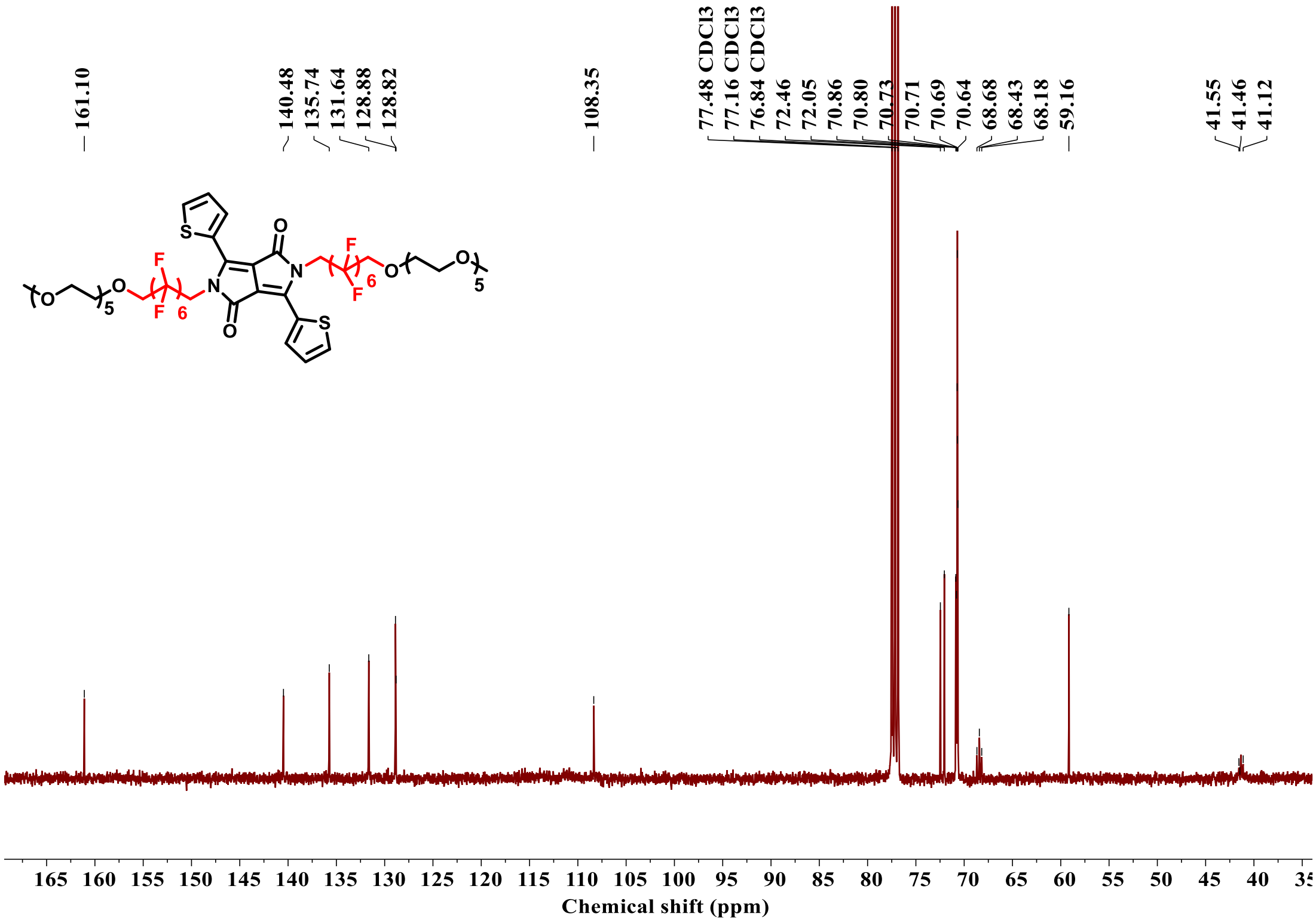

Figure S70. ^{13}C NMR spectrum of compound A9-6.

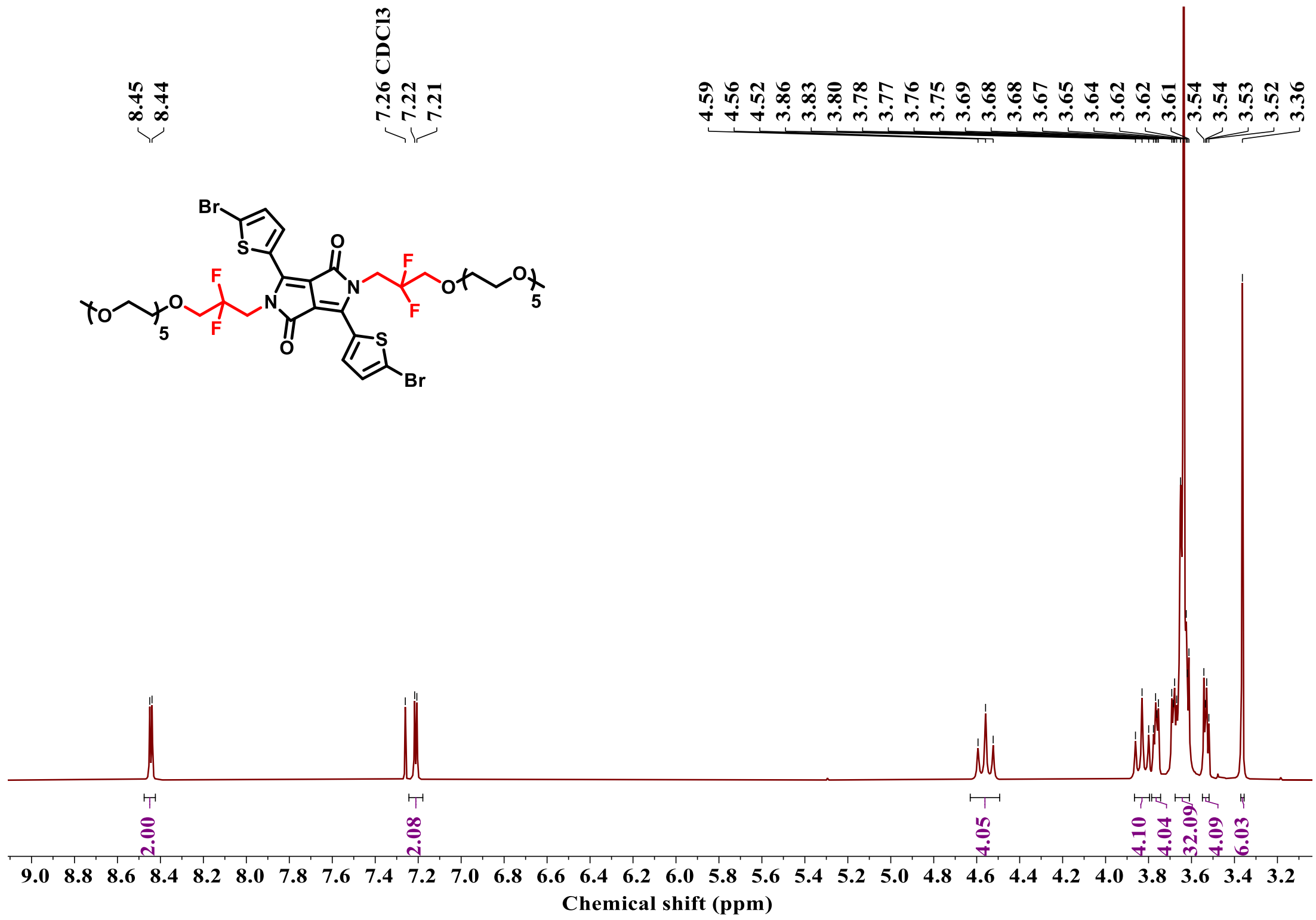

Figure S71. ^{1}H NMR spectrum of compound A10-1.

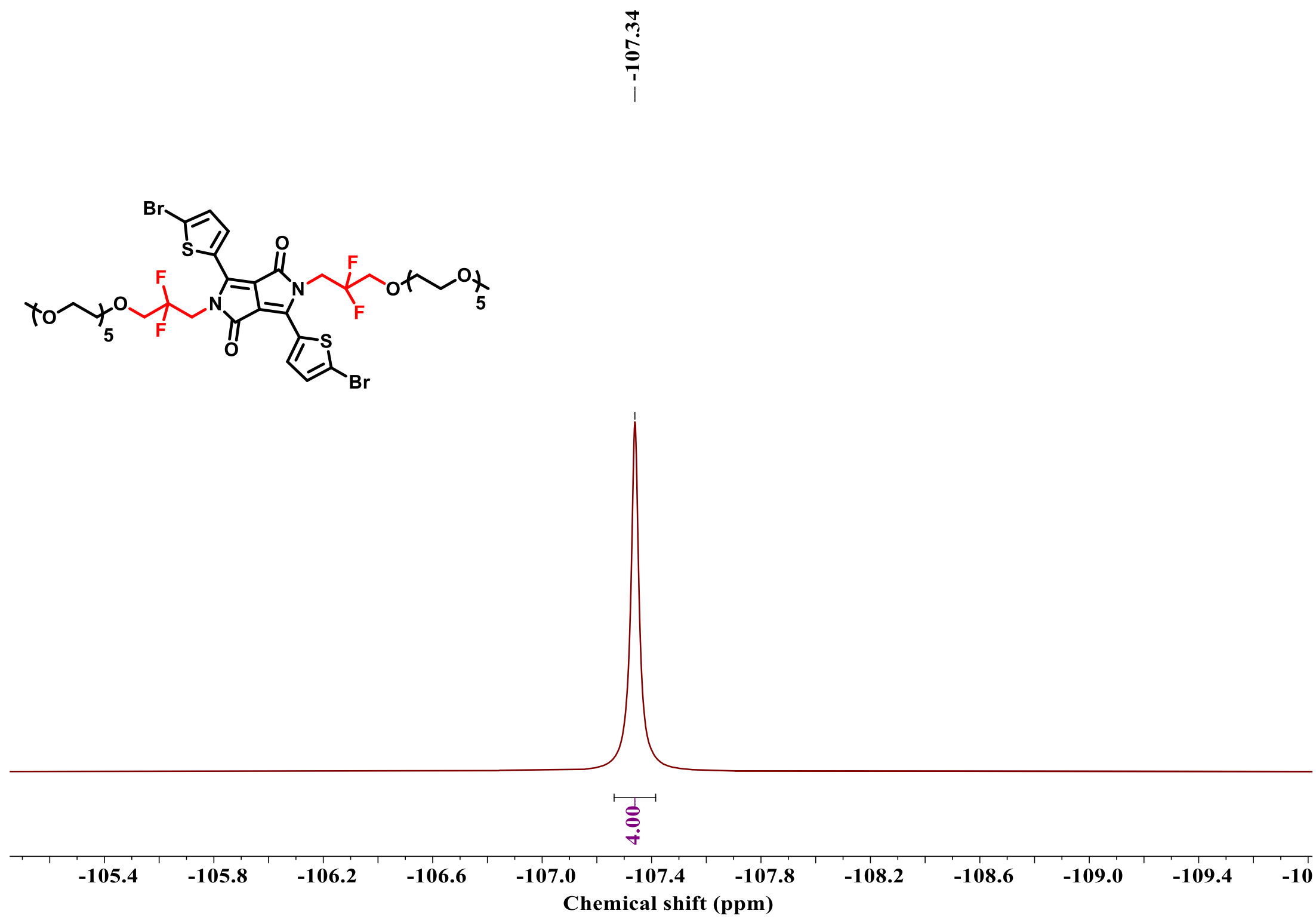

Figure S72. ^{19}F NMR spectrum of compound A10-1.

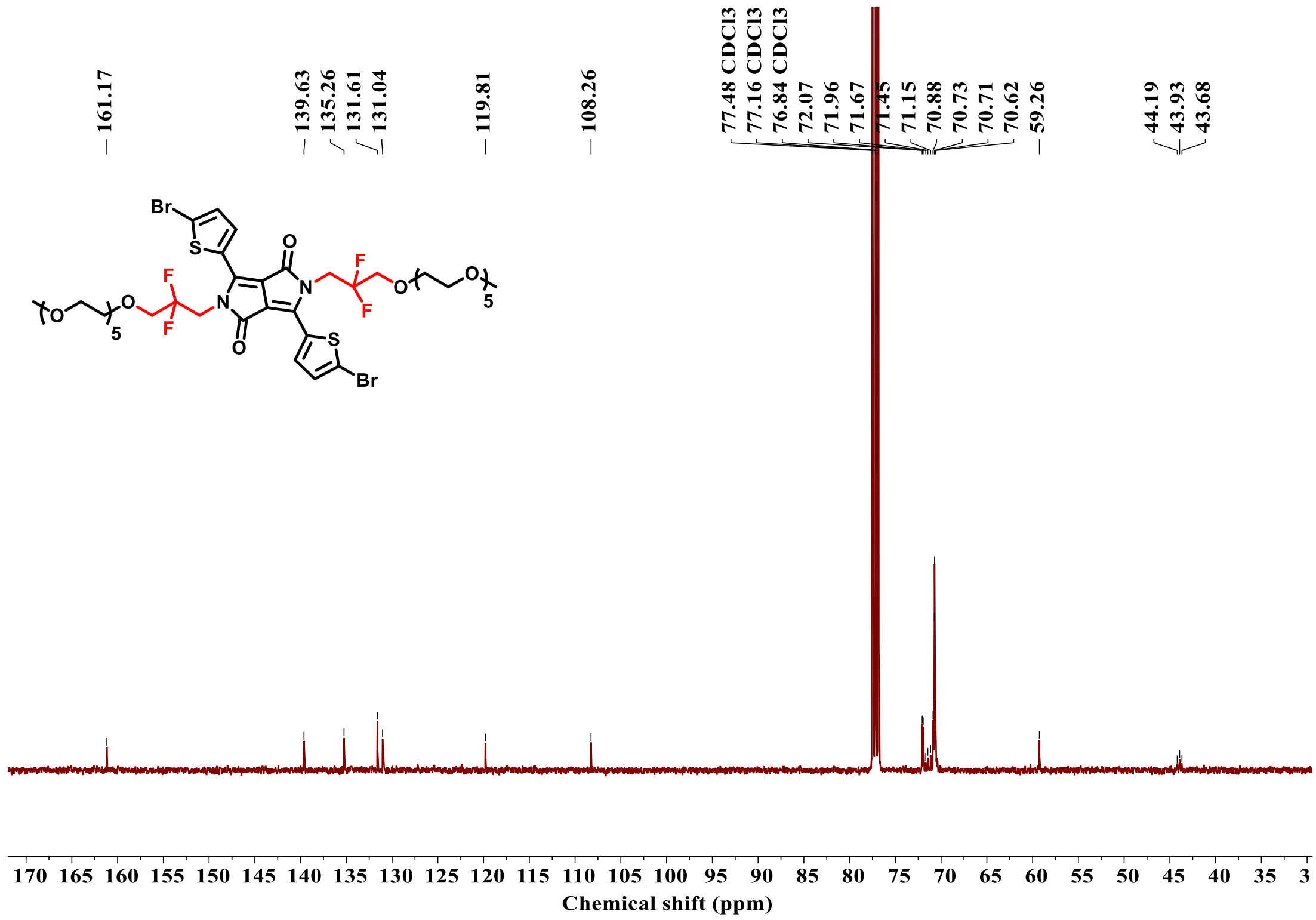

Figure S73. ^{13}C NMR spectrum of compound A10-1.

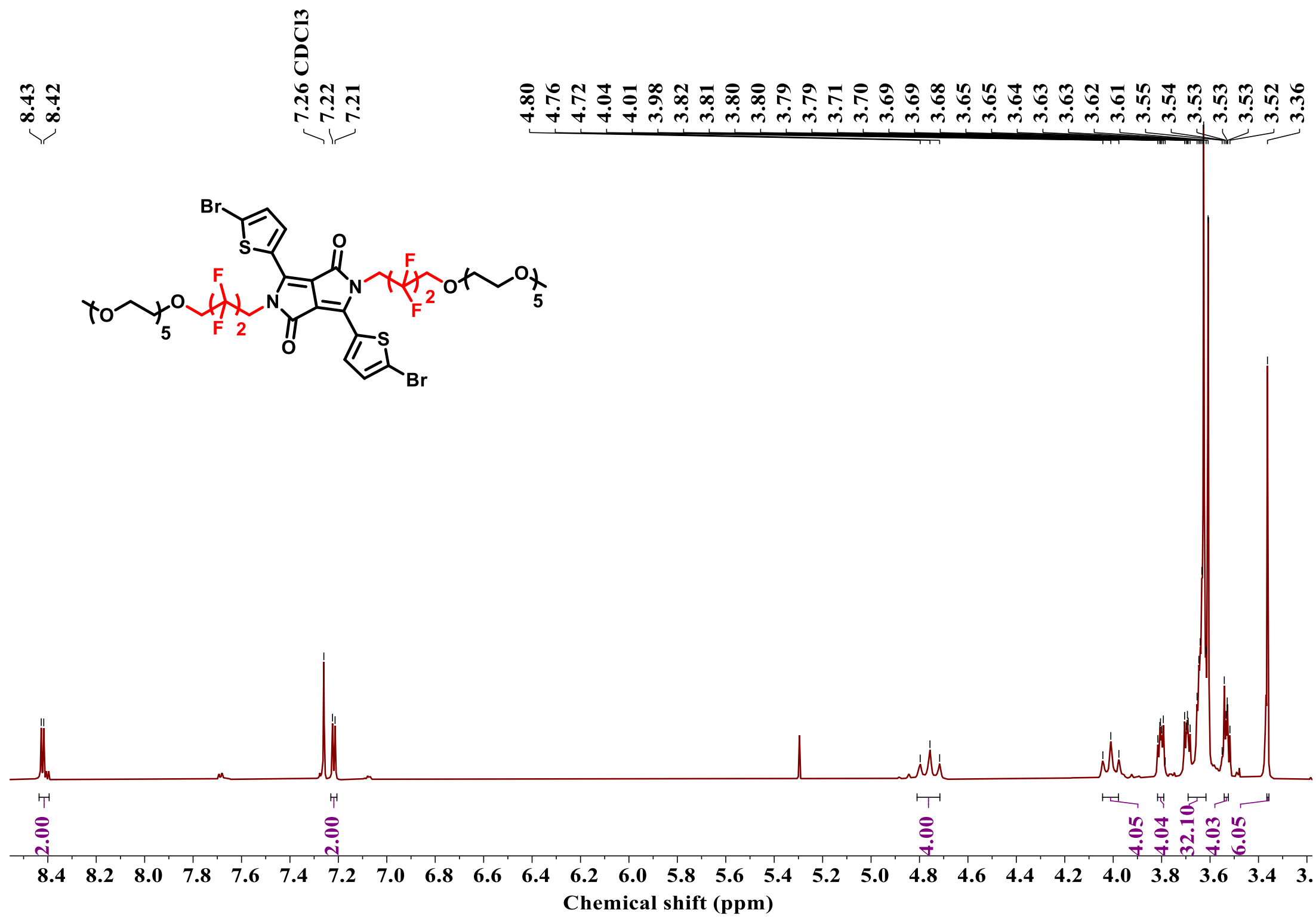

Figure S74. ^{1}H NMR spectrum of compound A10-2.

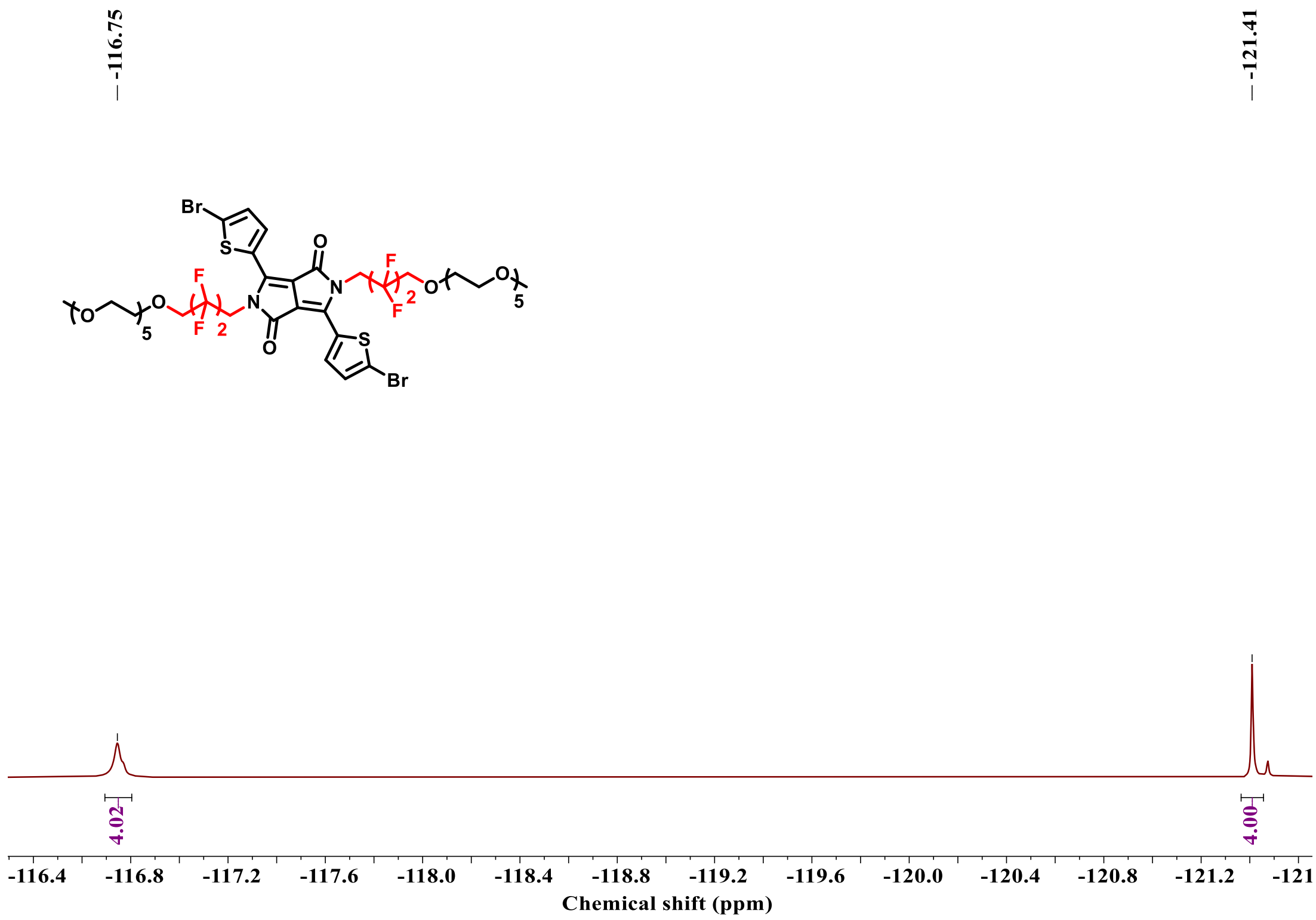

Figure S75. ^{19}F NMR spectrum of compound A10-2.

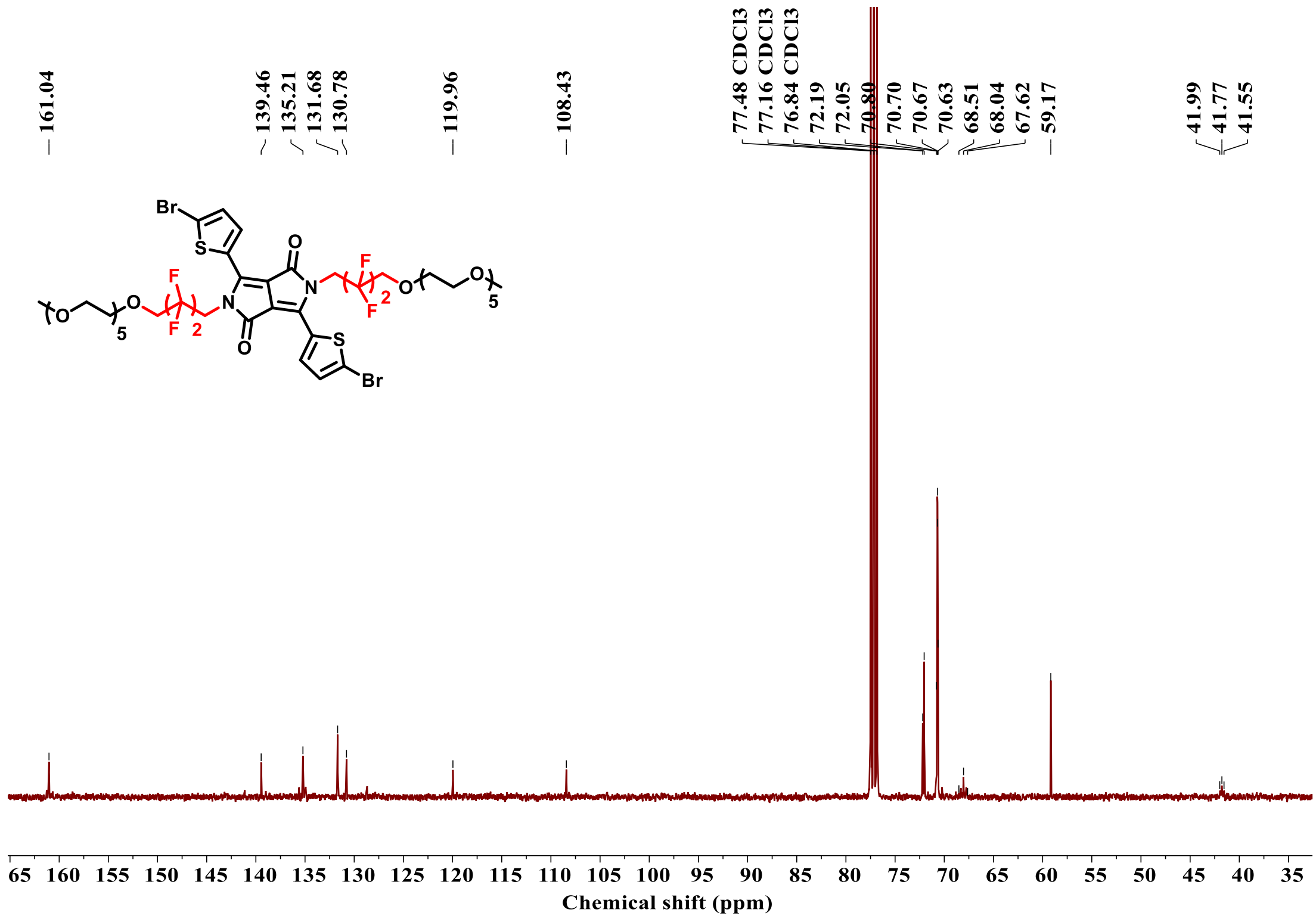

Figure S76. ^{13}C NMR spectrum of compound A10-2.

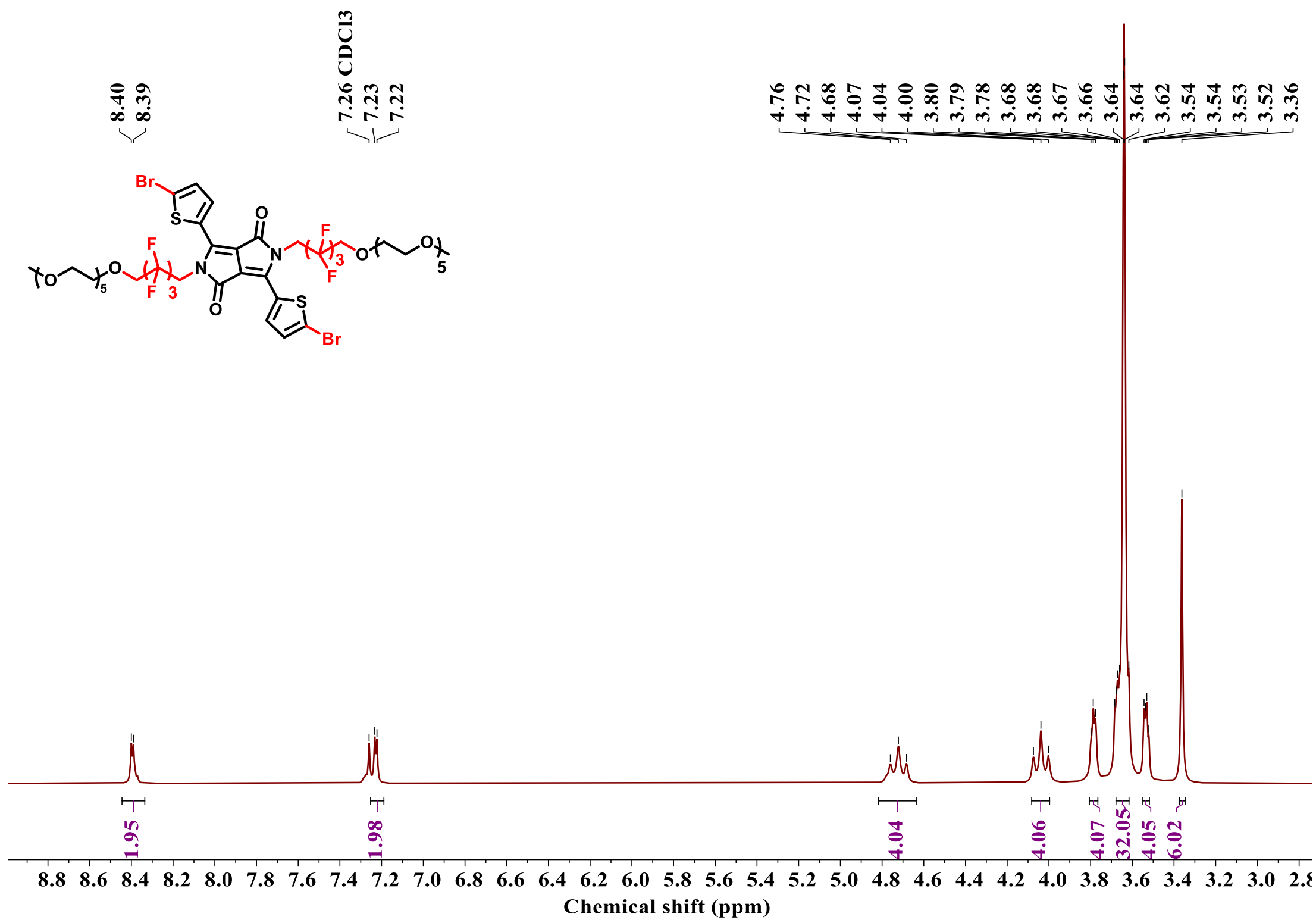

Figure S77. ^{1}H NMR spectrum of compound A10-3.

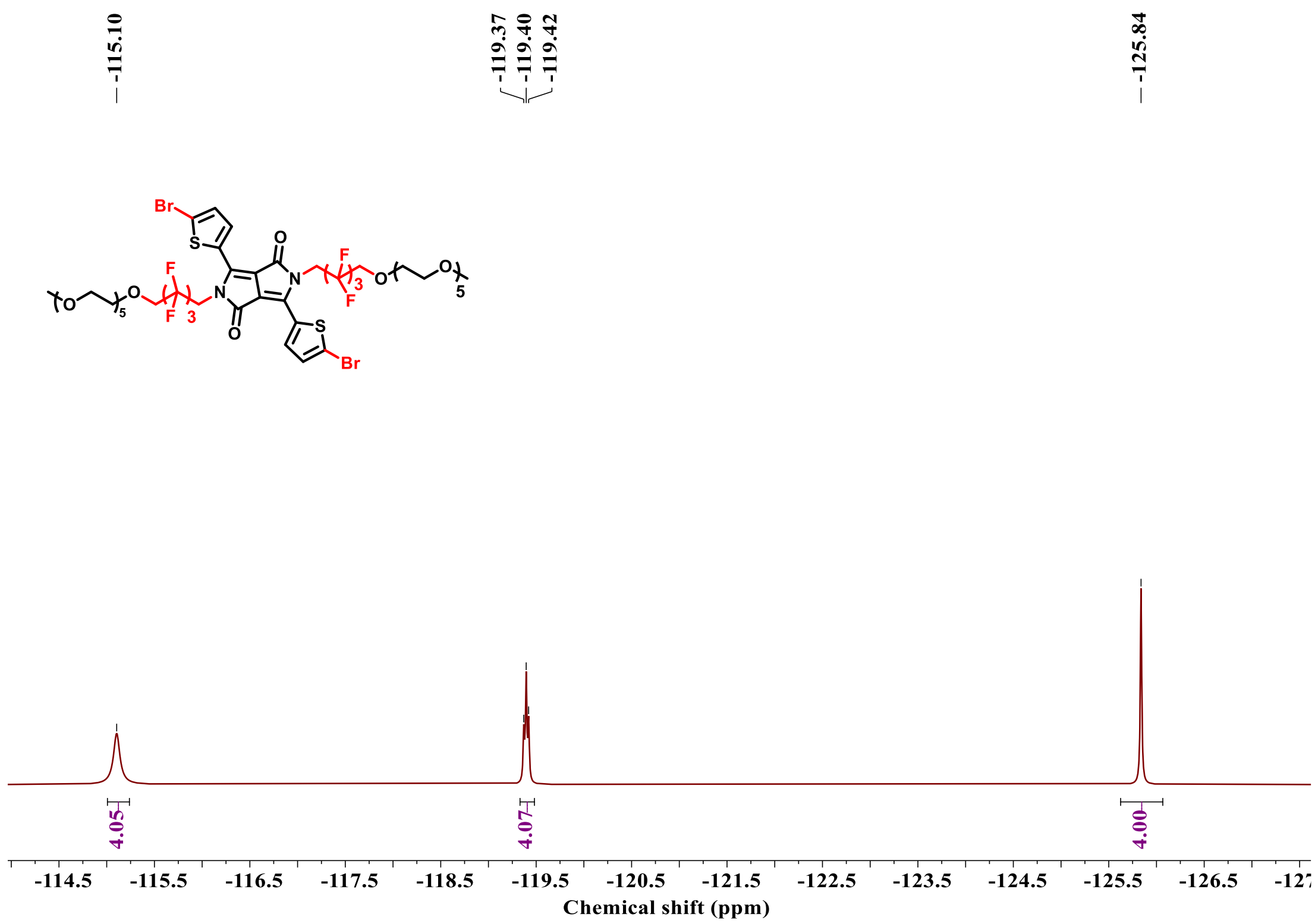

Figure S78. ^{19}F NMR spectrum of compound A10-3.

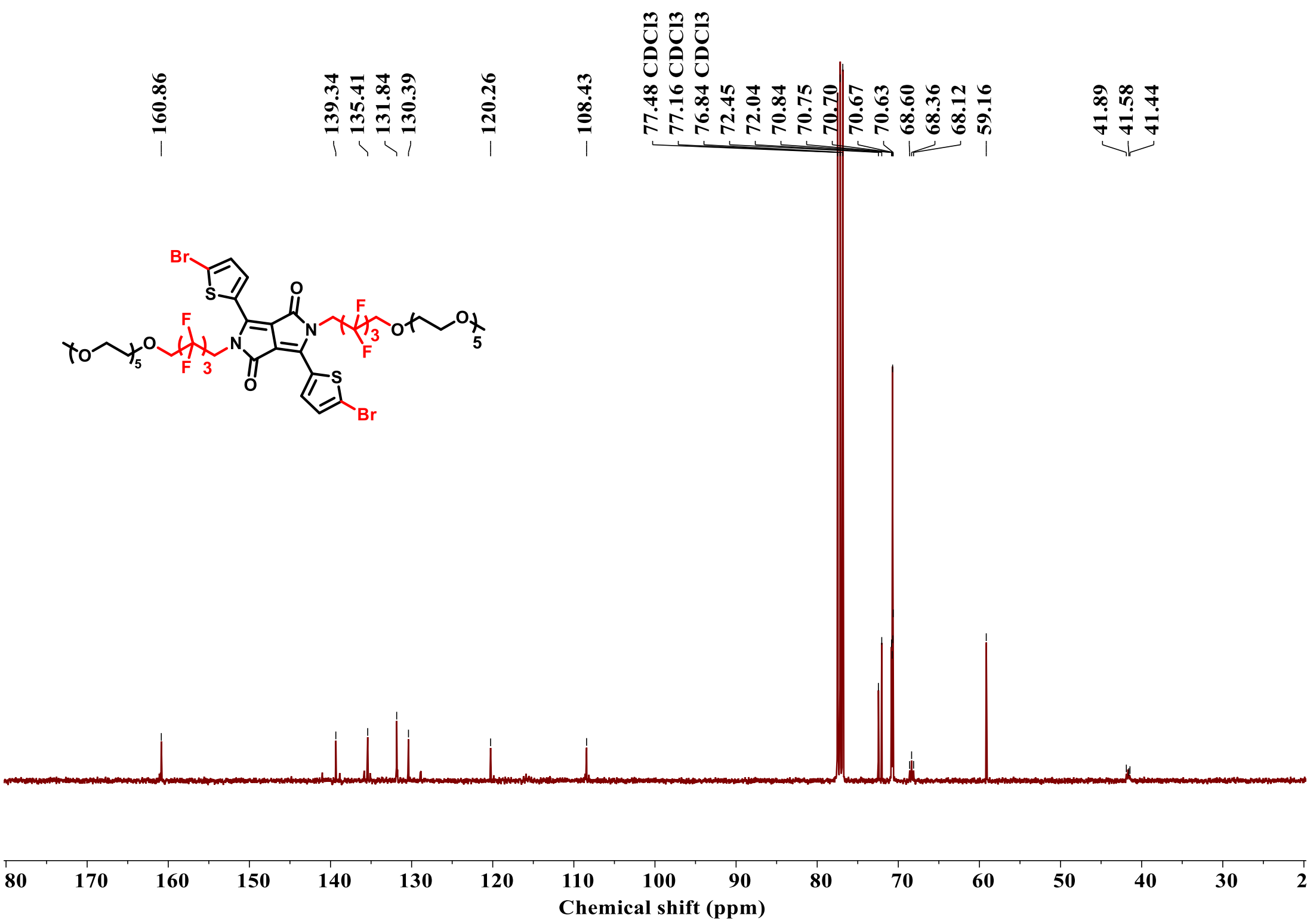

Figure S79. ^{13}C NMR spectrum of compound A10-3.

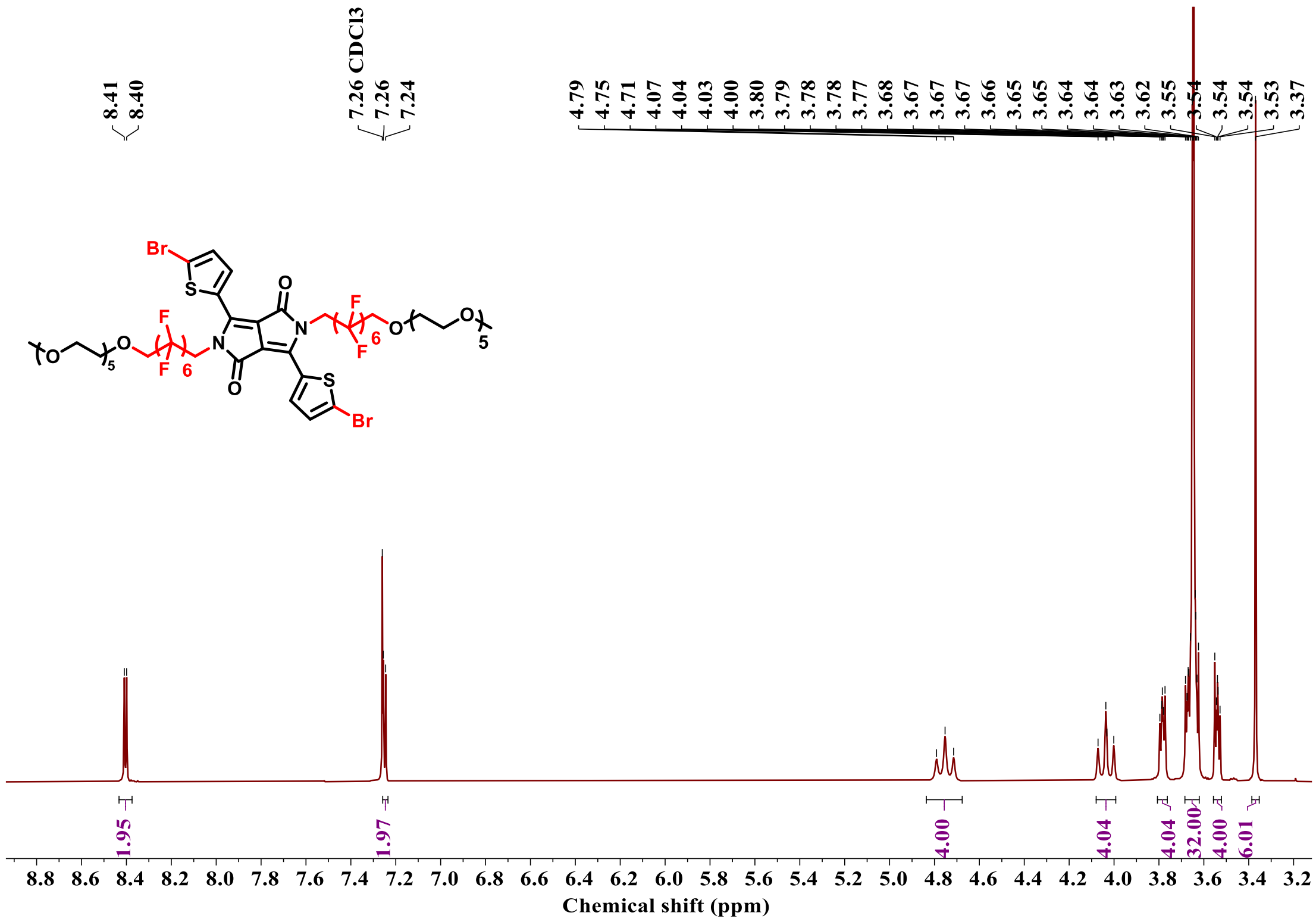

Figure S80. ^{1}H NMR spectra of compound A10-6.

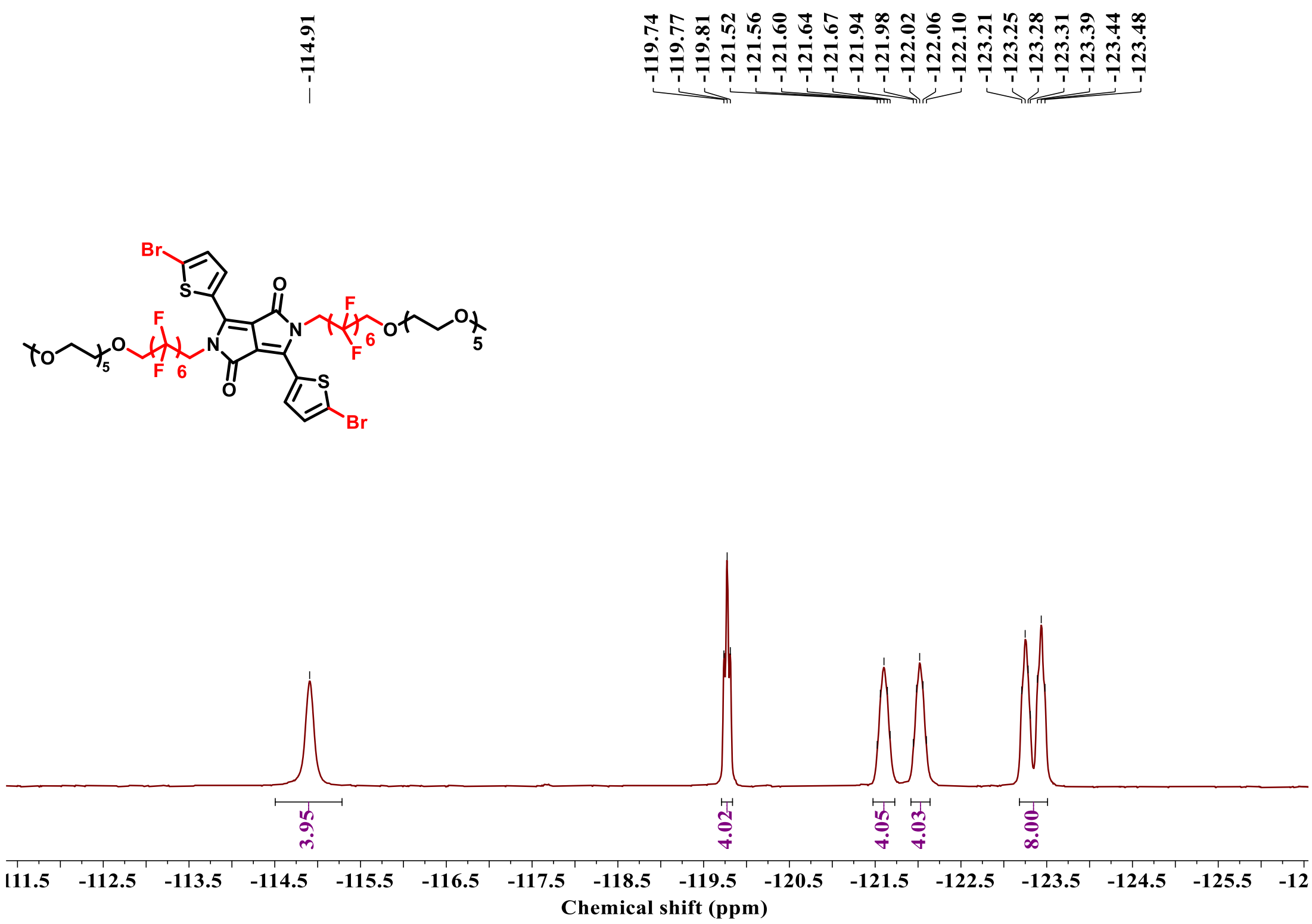

Figure S81. ^{19}F NMR spectra of compound A10-6.

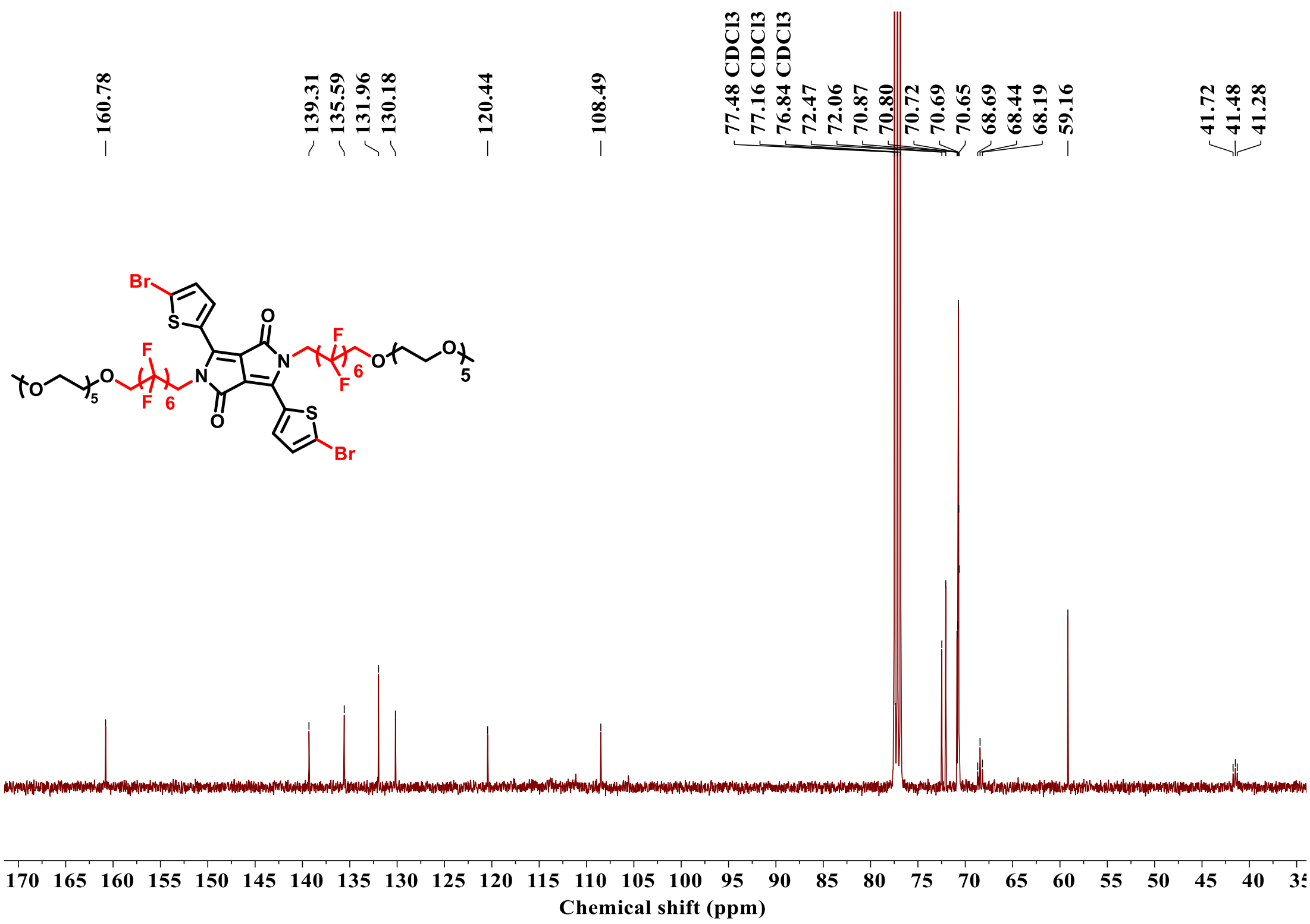

Figure S82. ^{13}C NMR spectra of compound A10-6.

22.High Resolution Mass Spectrometry

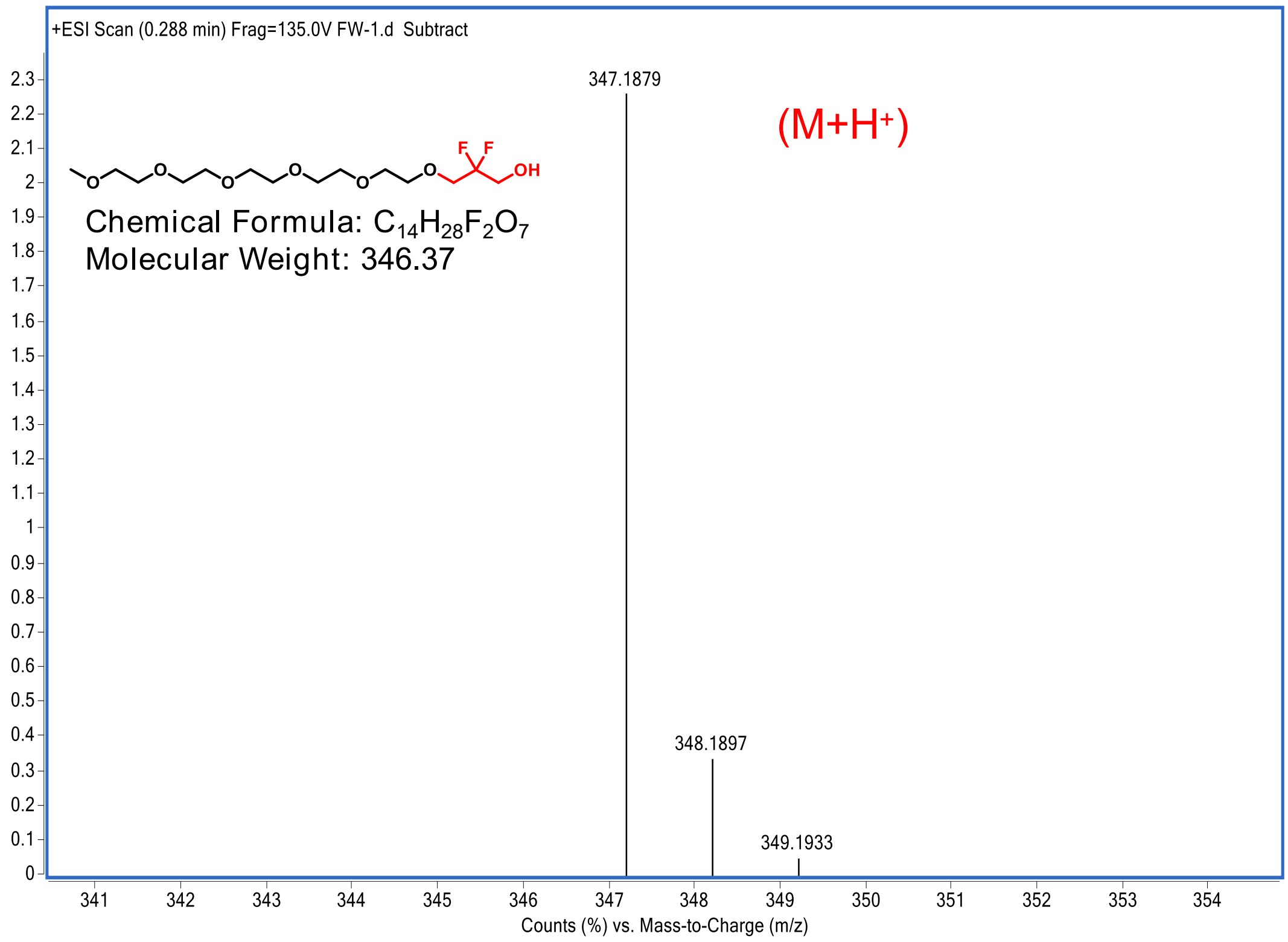

Figure S83. High resolution mass spectrometry of compound A5-1.

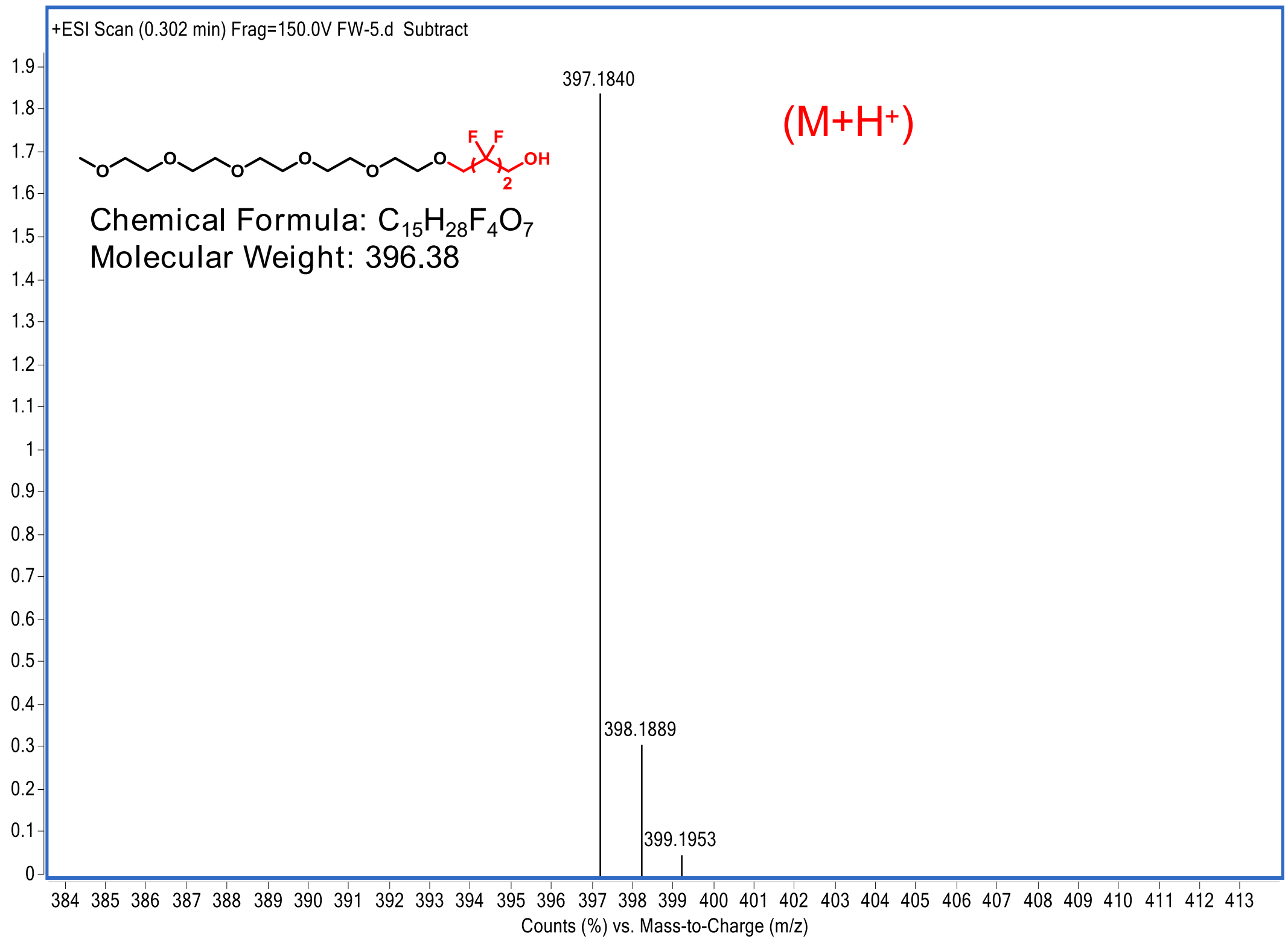

Figure S84. High resolution mass spectrometry of compound A5-2.

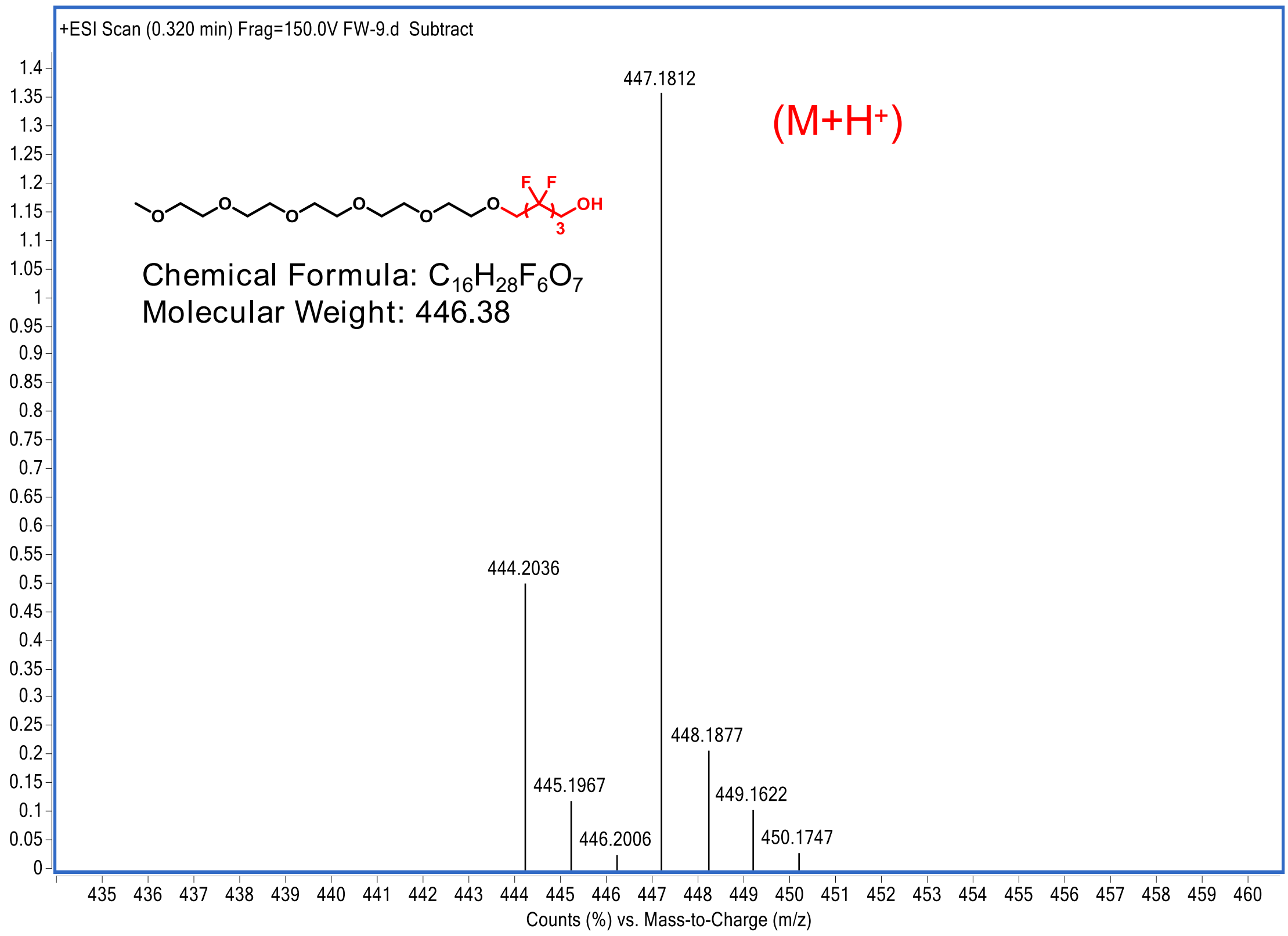

Figure S85. High resolution mass spectrometry of compound A5-3.

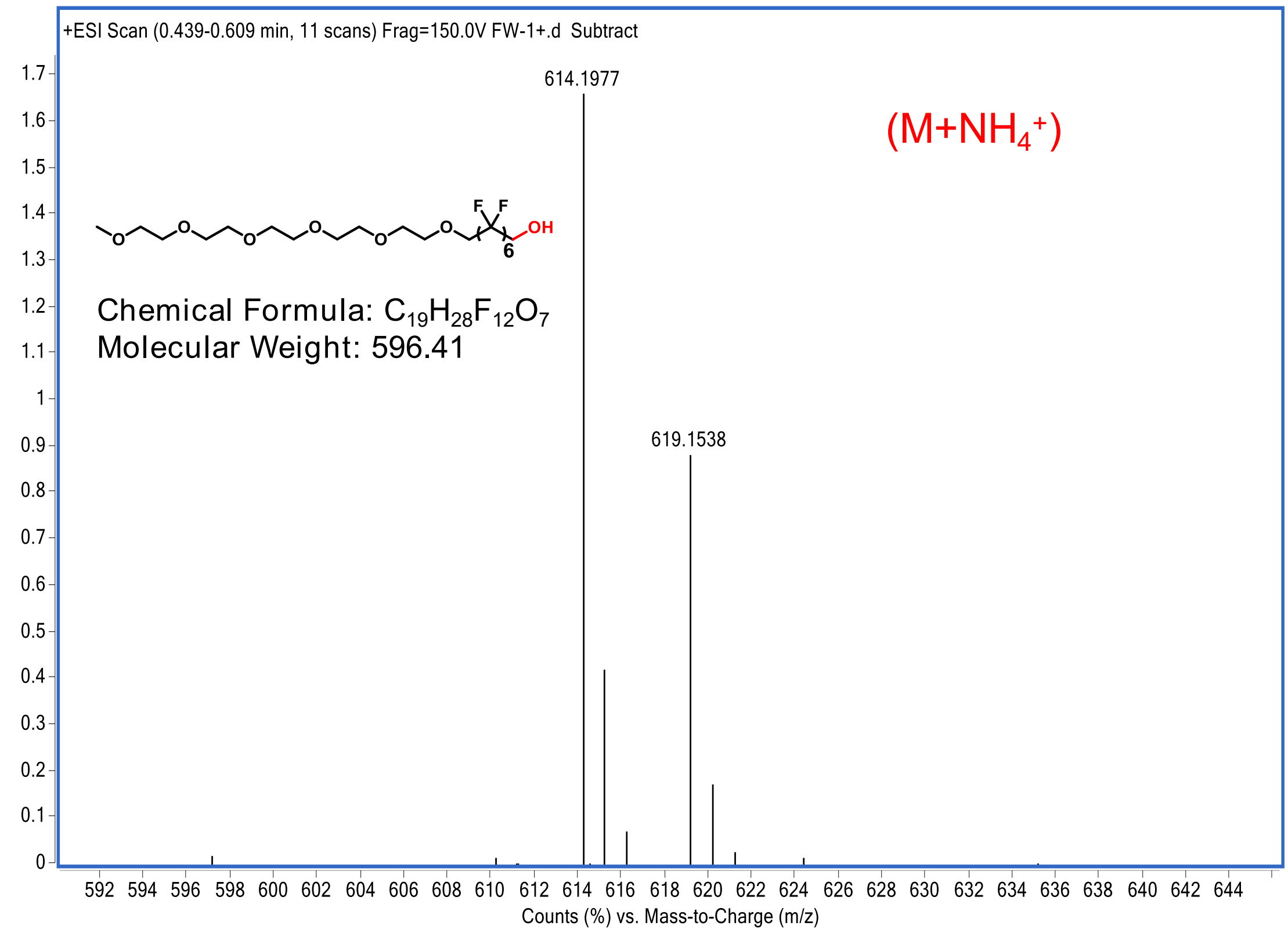

Figure S86. High resolution mass spectrometry of compound A5-6.

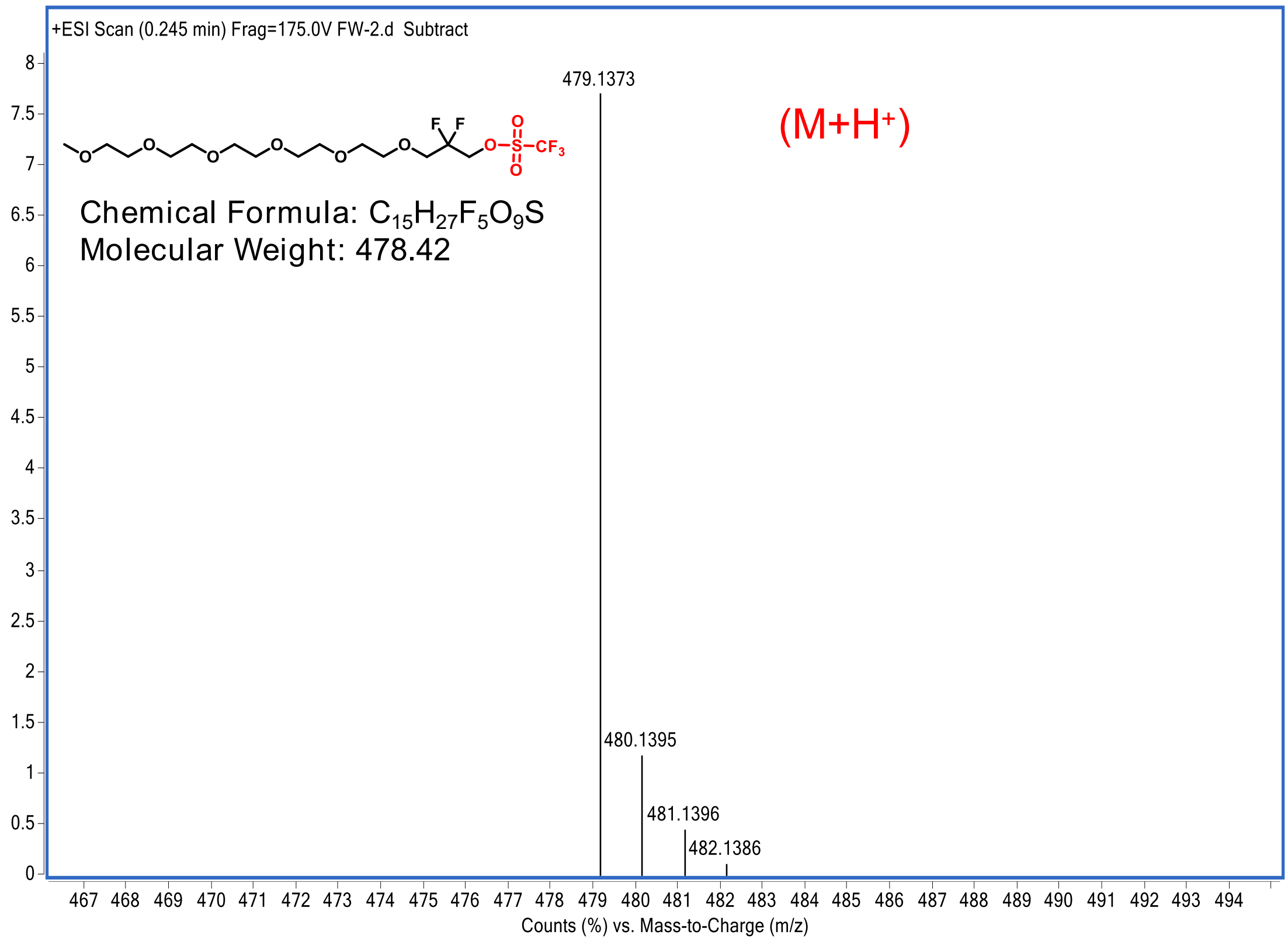

Figure S87. High resolution mass spectrometry of compound A7-1.

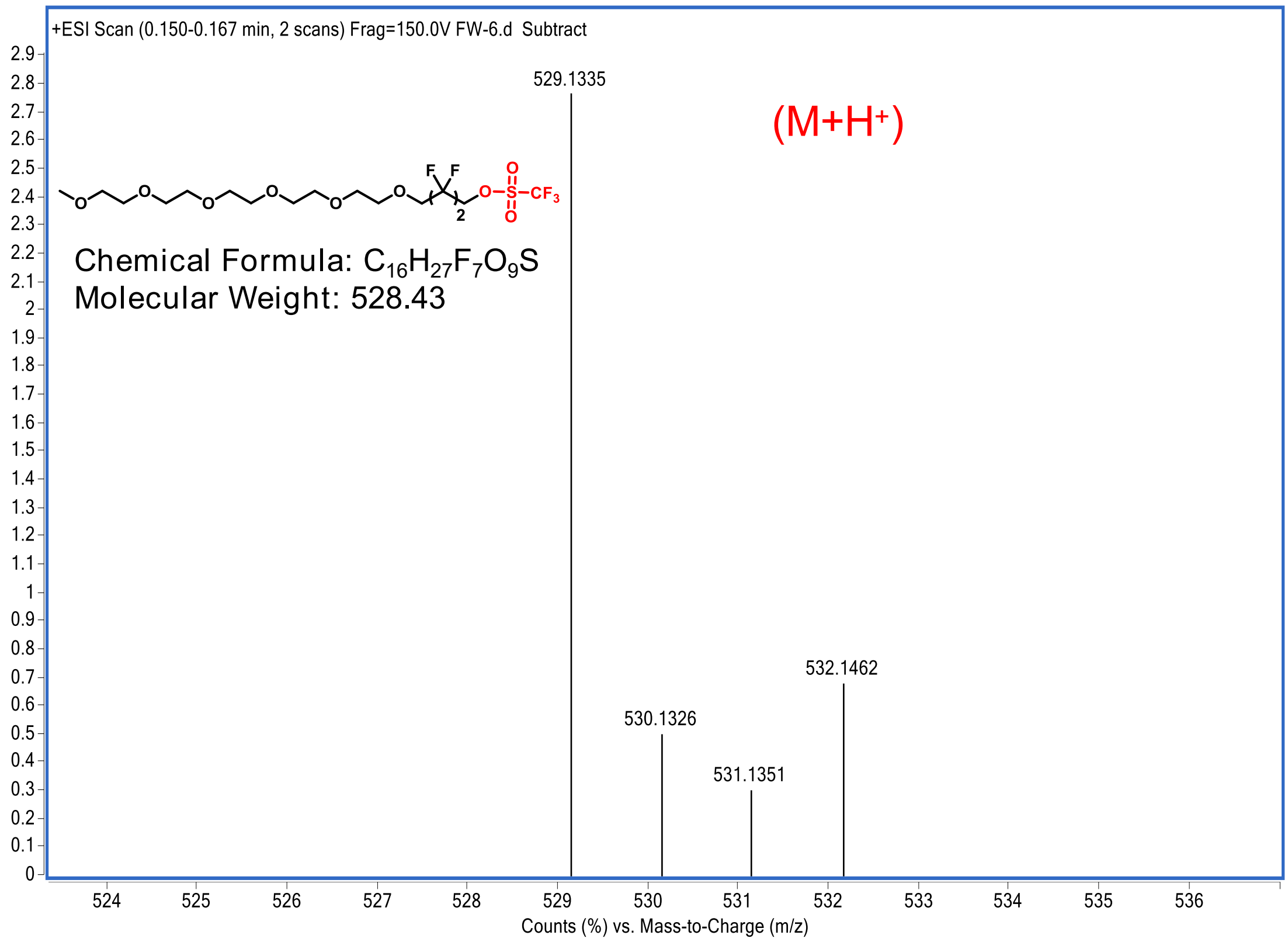

Figure S88. High resolution mass spectrometry of compound A7-2.

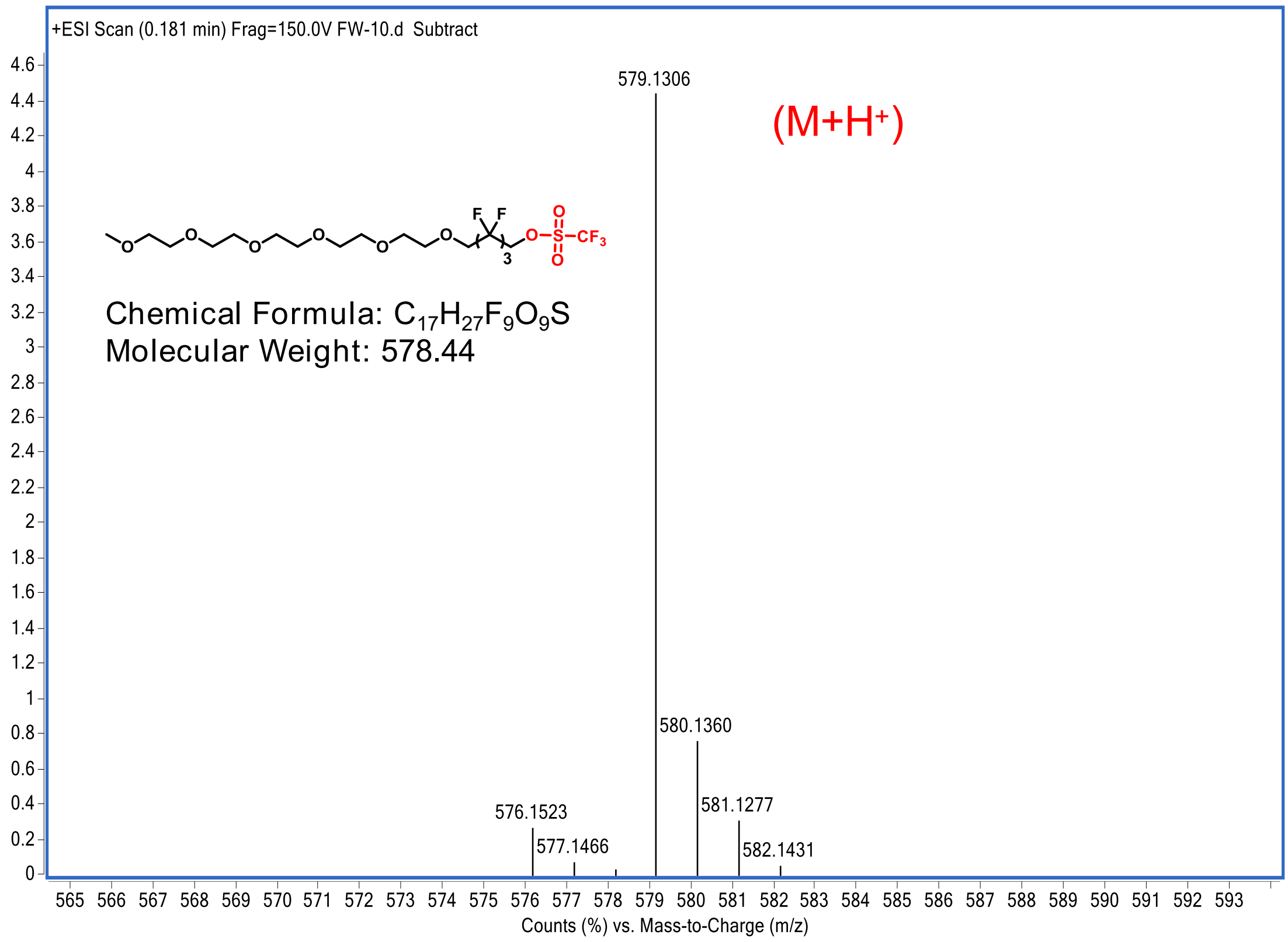

Figure S89. High resolution mass spectrometry of compound A7-3.

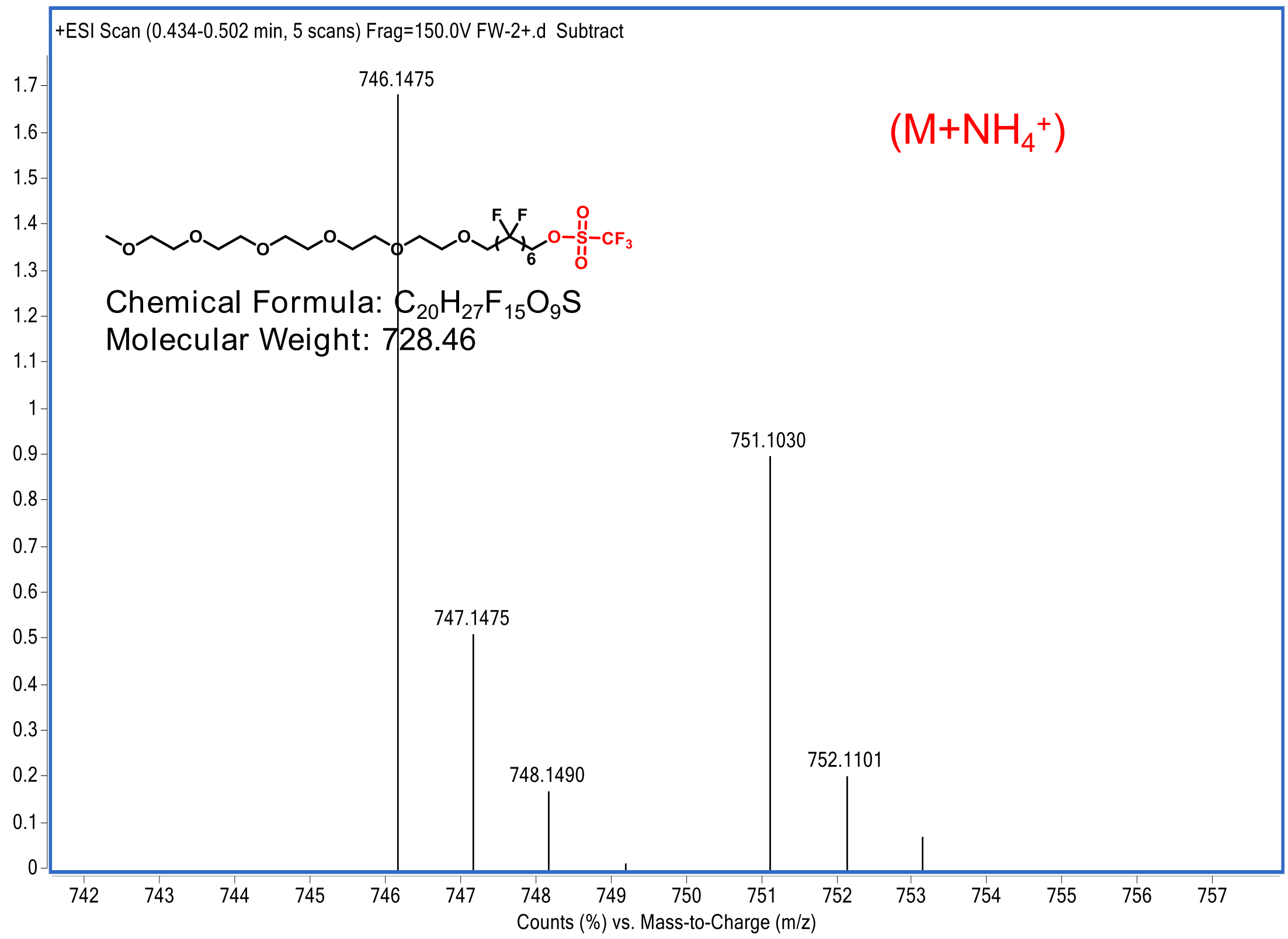

Figure S90. High resolution mass spectrometry of compound A7-6.

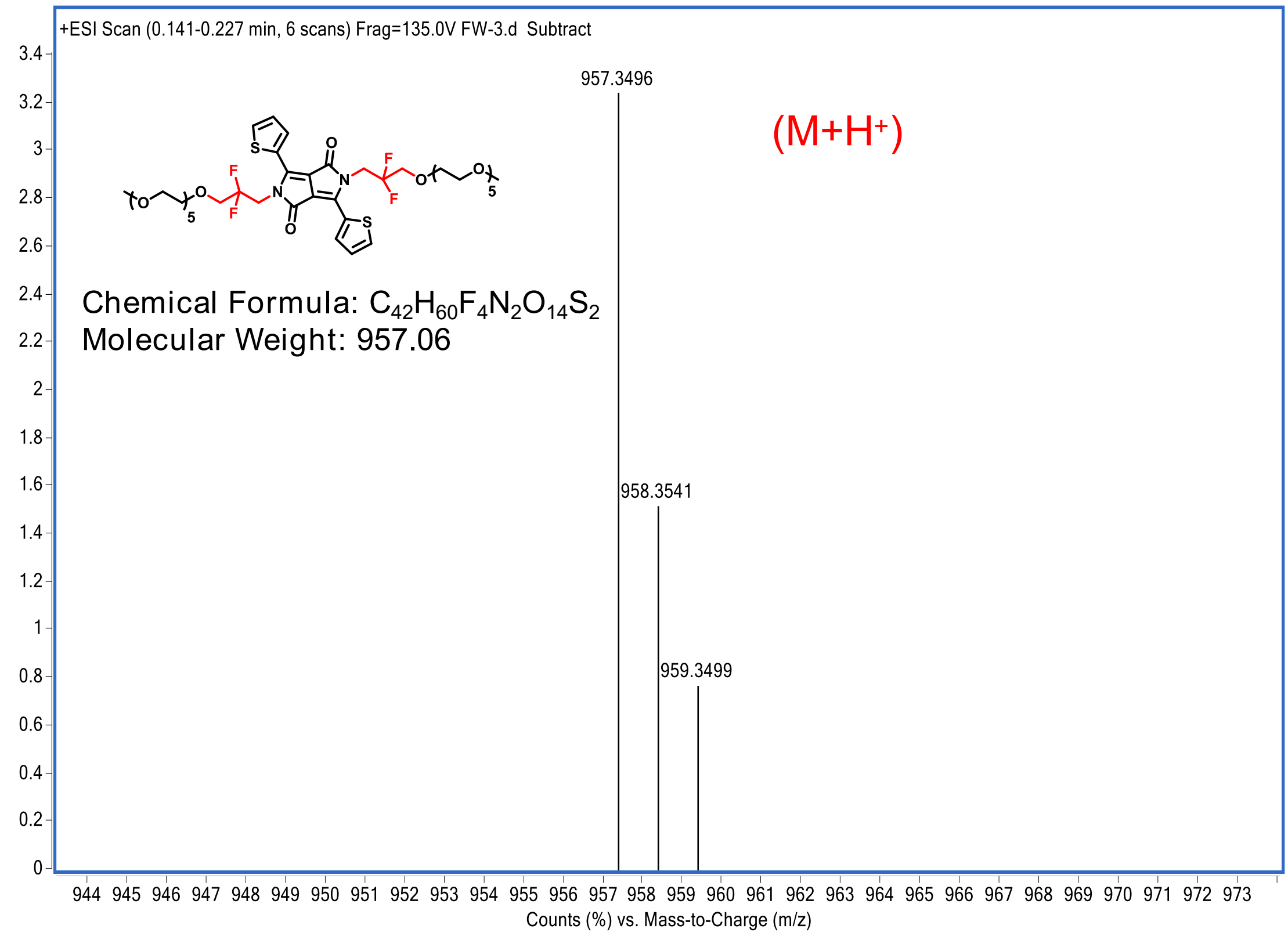

Figure S91. High resolution mass spectrometry of compound A9-1.

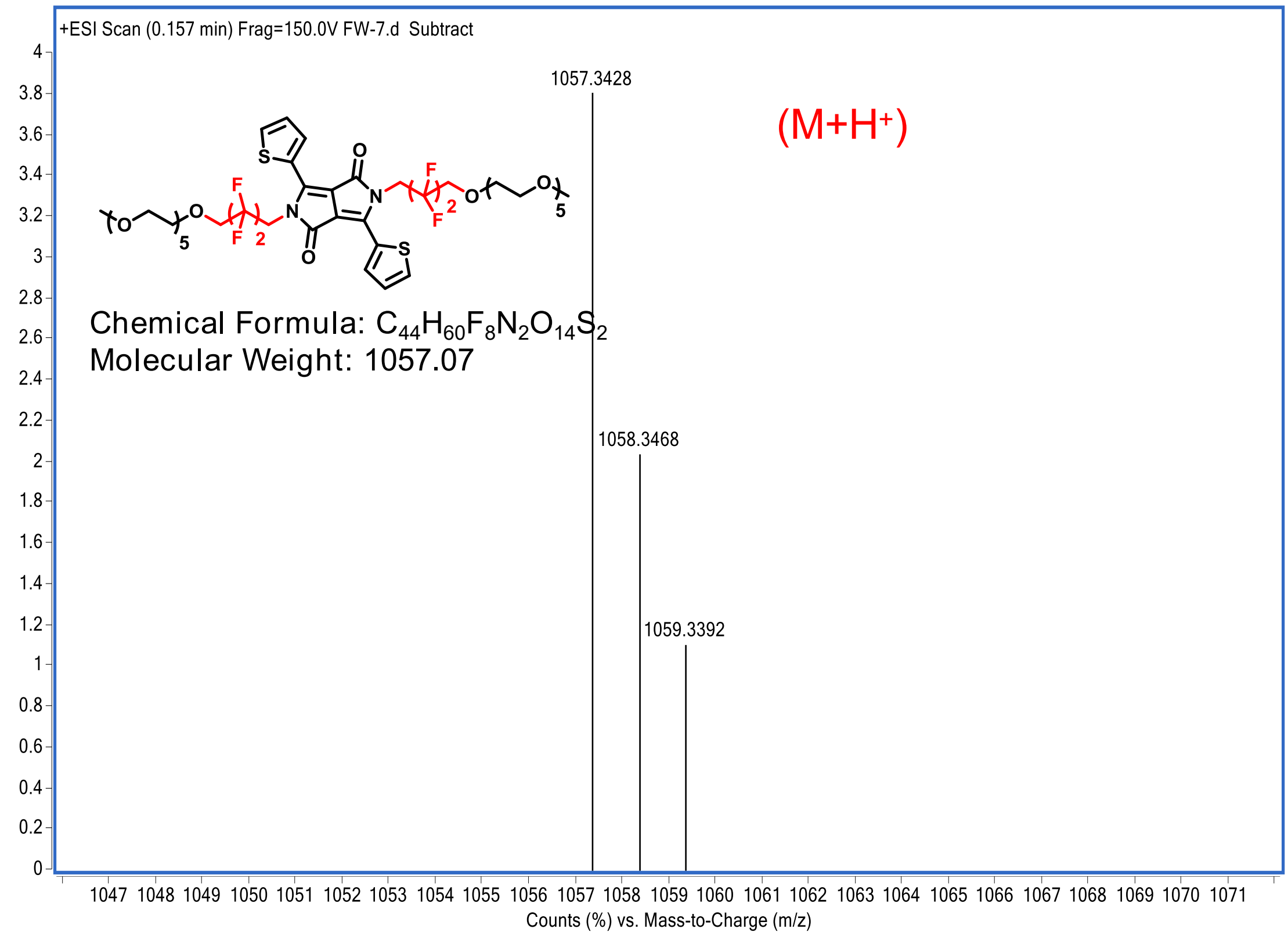

Figure S92. High resolution mass spectrometry of compound A9-2.

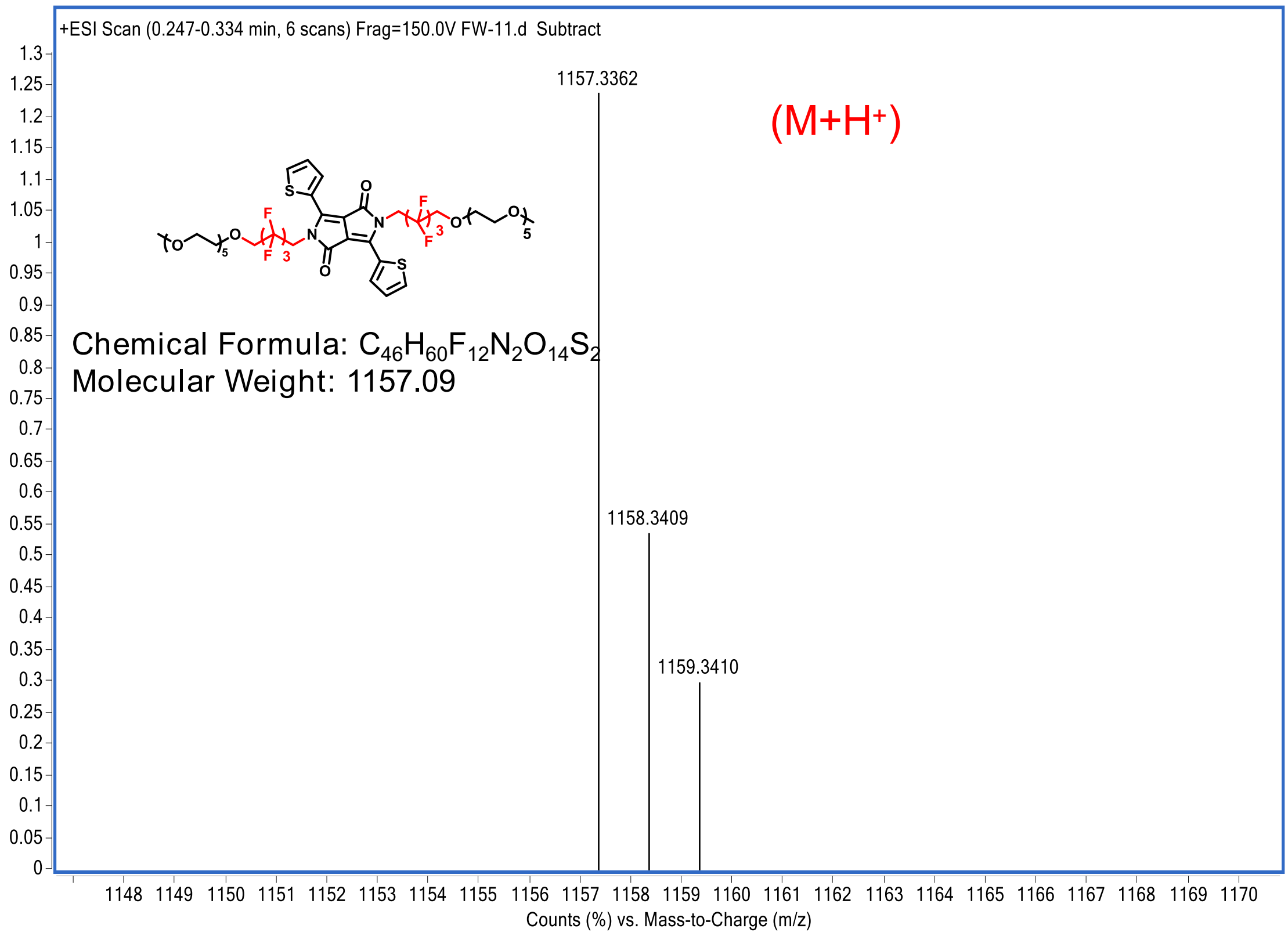

Figure S93. High resolution mass spectrometry of compound A9-3.

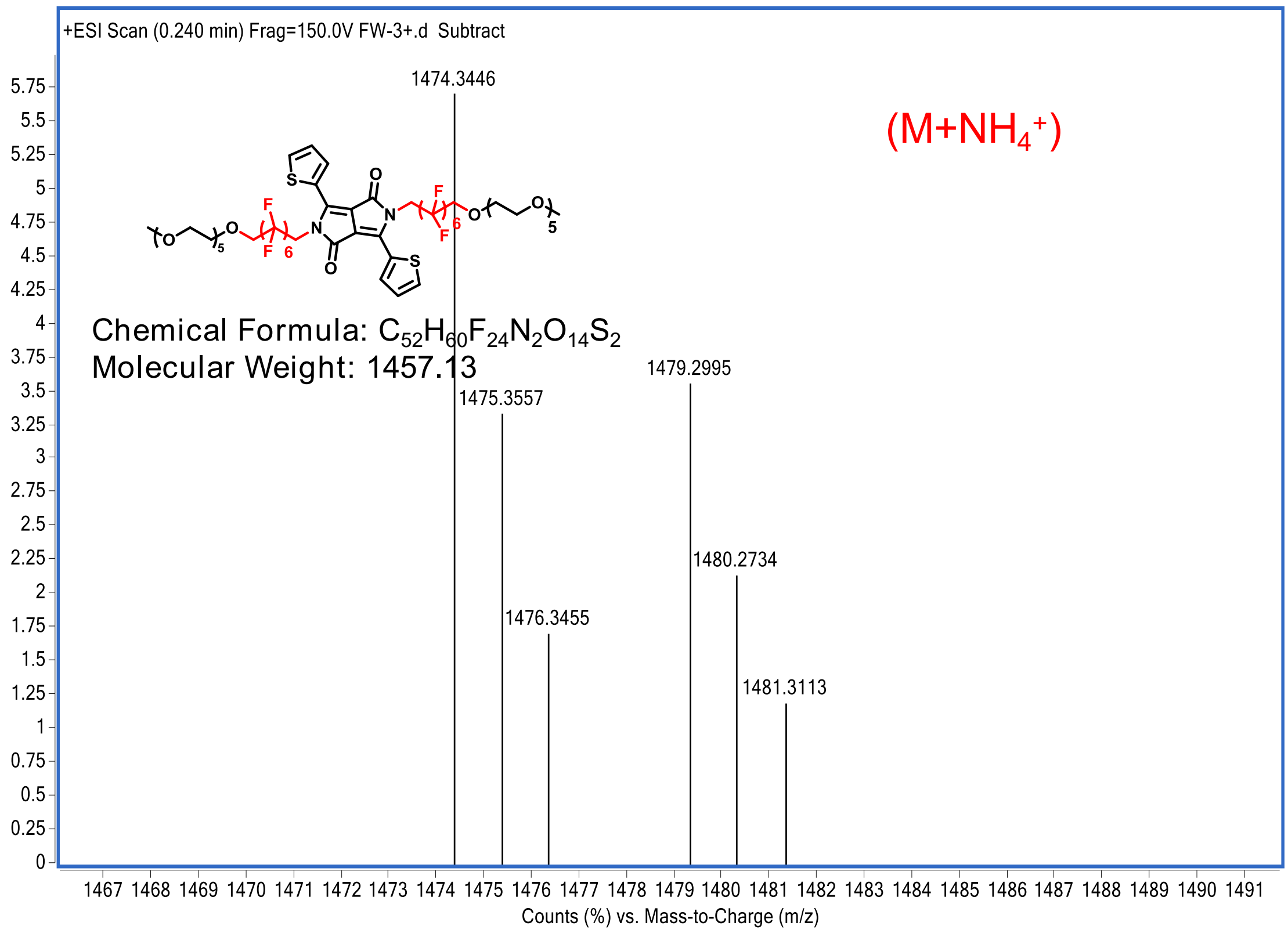

Figure S94. High resolution mass spectrometry of compound A9-6.

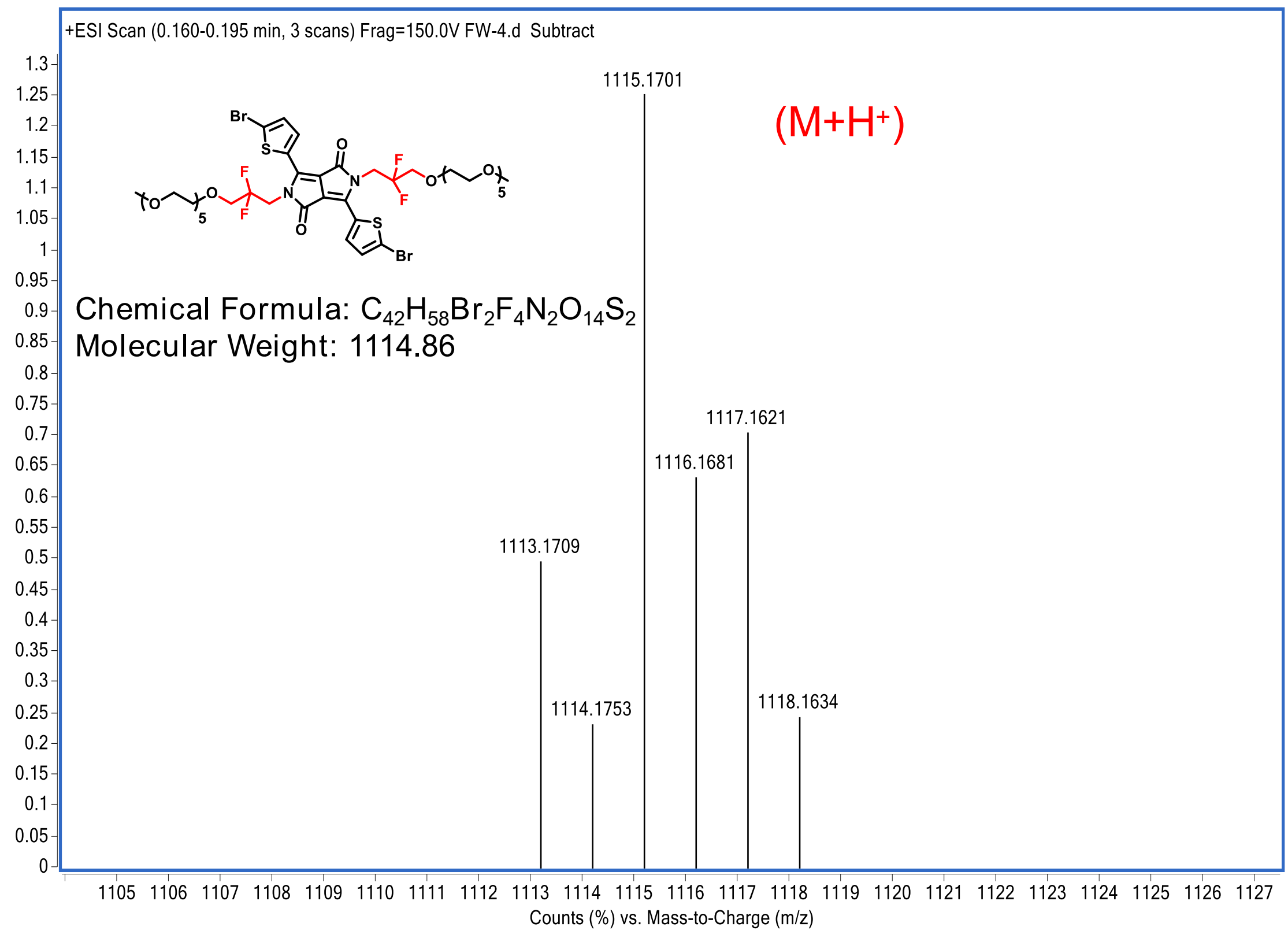

Figure S95. High resolution mass spectrometry of compound A10-1.

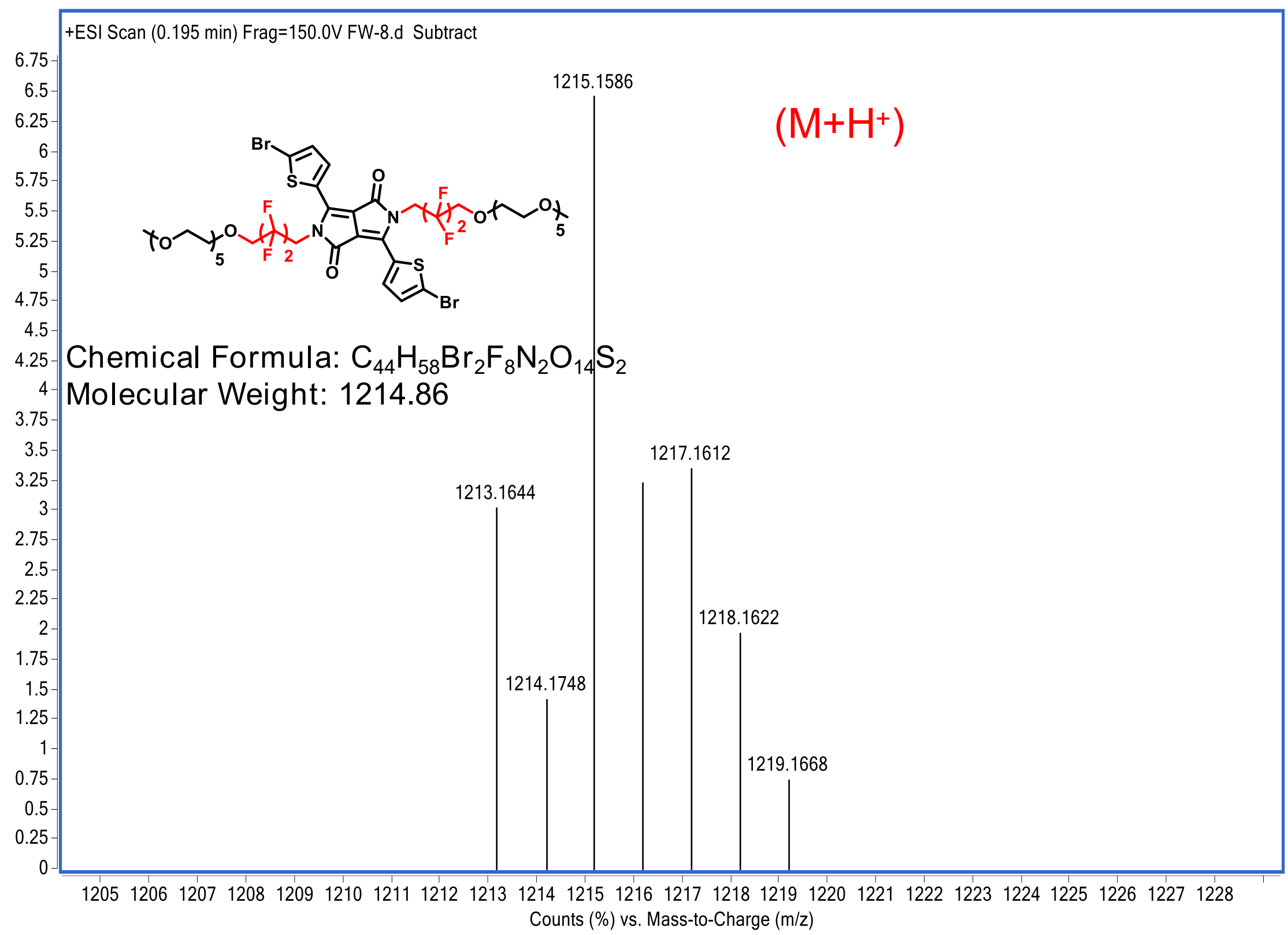

Figure S96. High resolution mass spectrometry of compound A10-2.

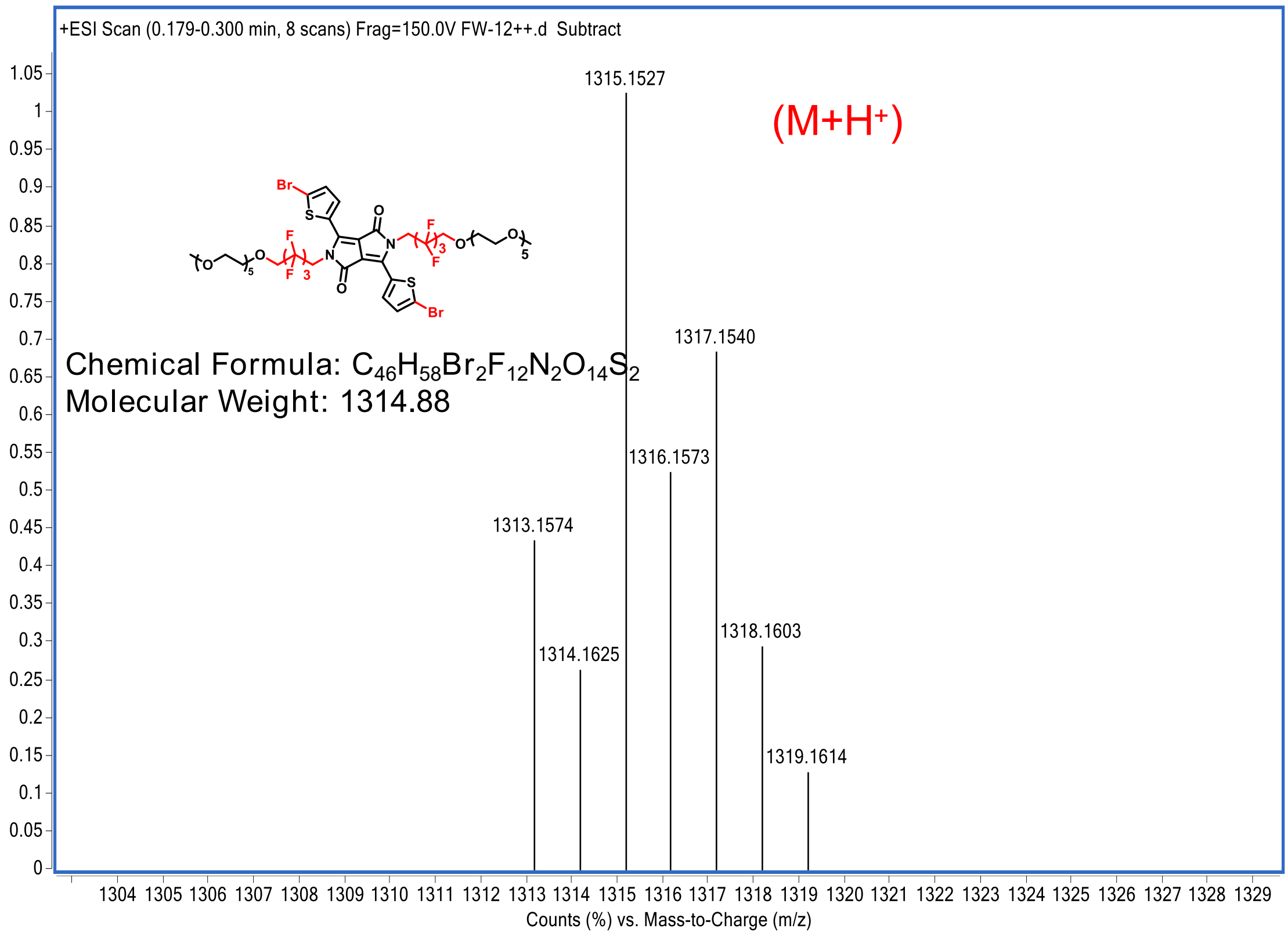

Figure S97. High resolution mass spectrometry of compound A10-3.

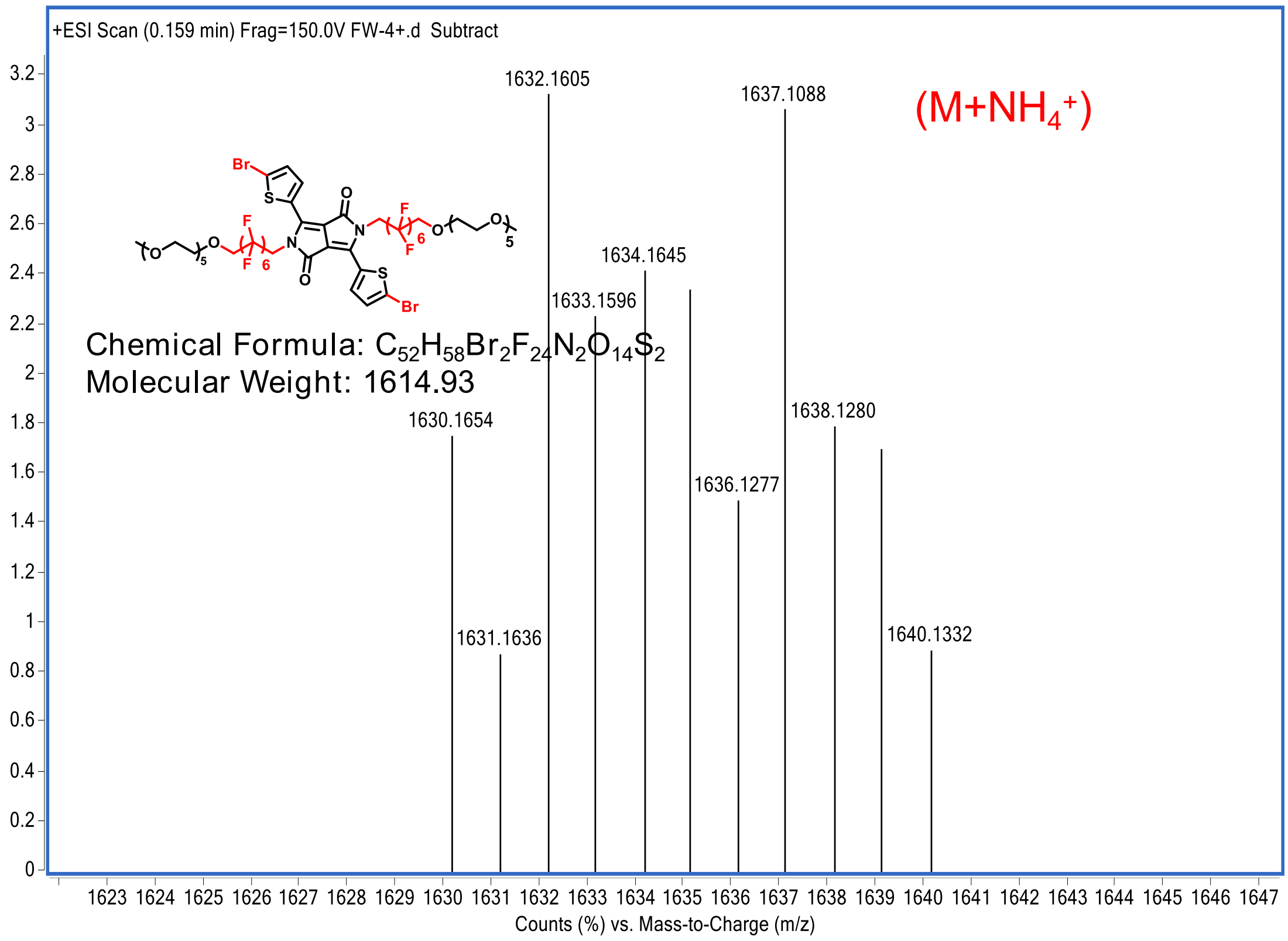

Figure S98. High resolution mass spectrometry of compound A10-6.

23.GPC Trace

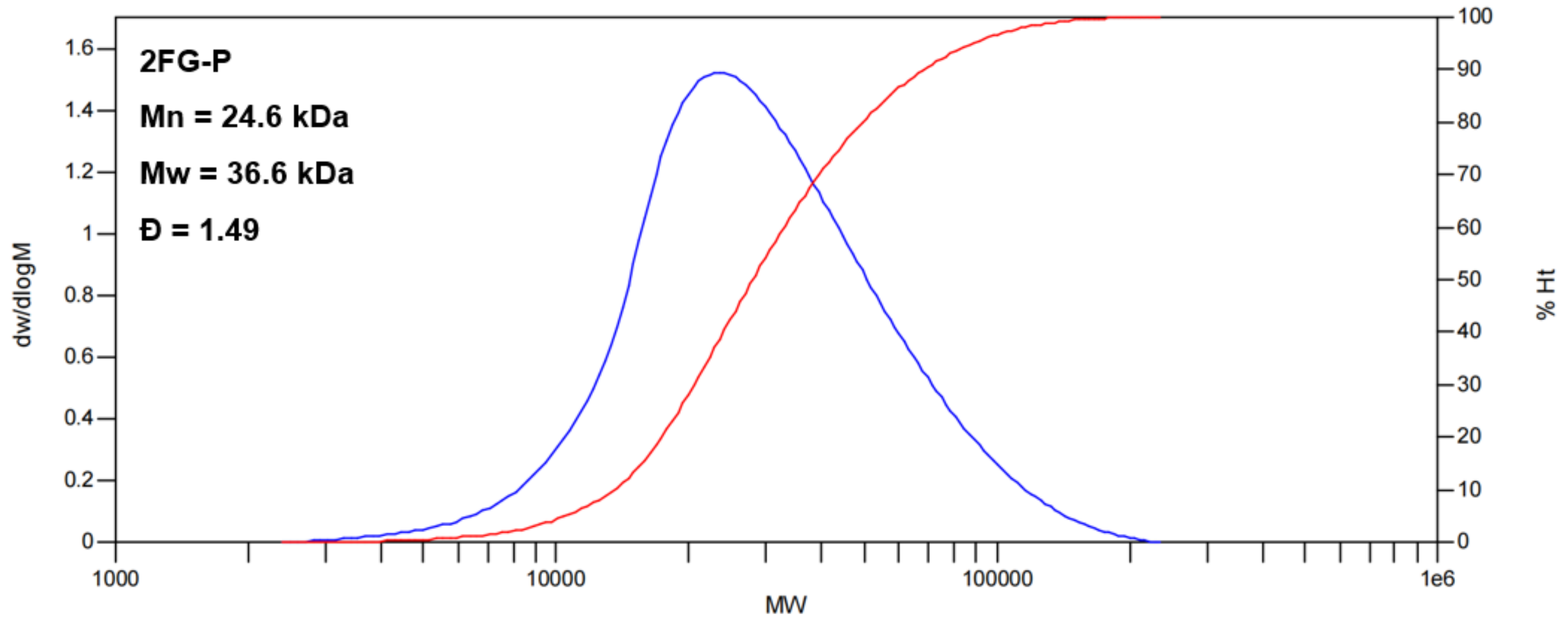

Figure S99. GPC trace of polymer 2FG-P.

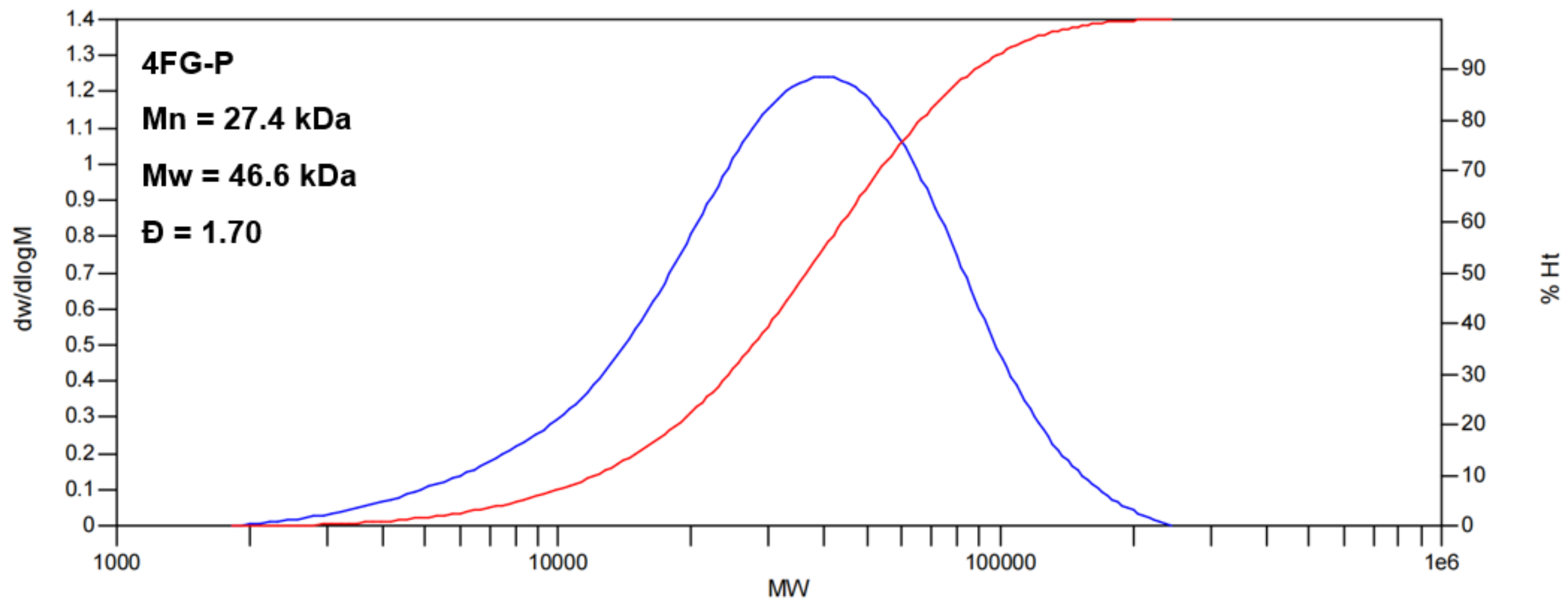

Figure S100. GPC trace of polymer 4FG-P.

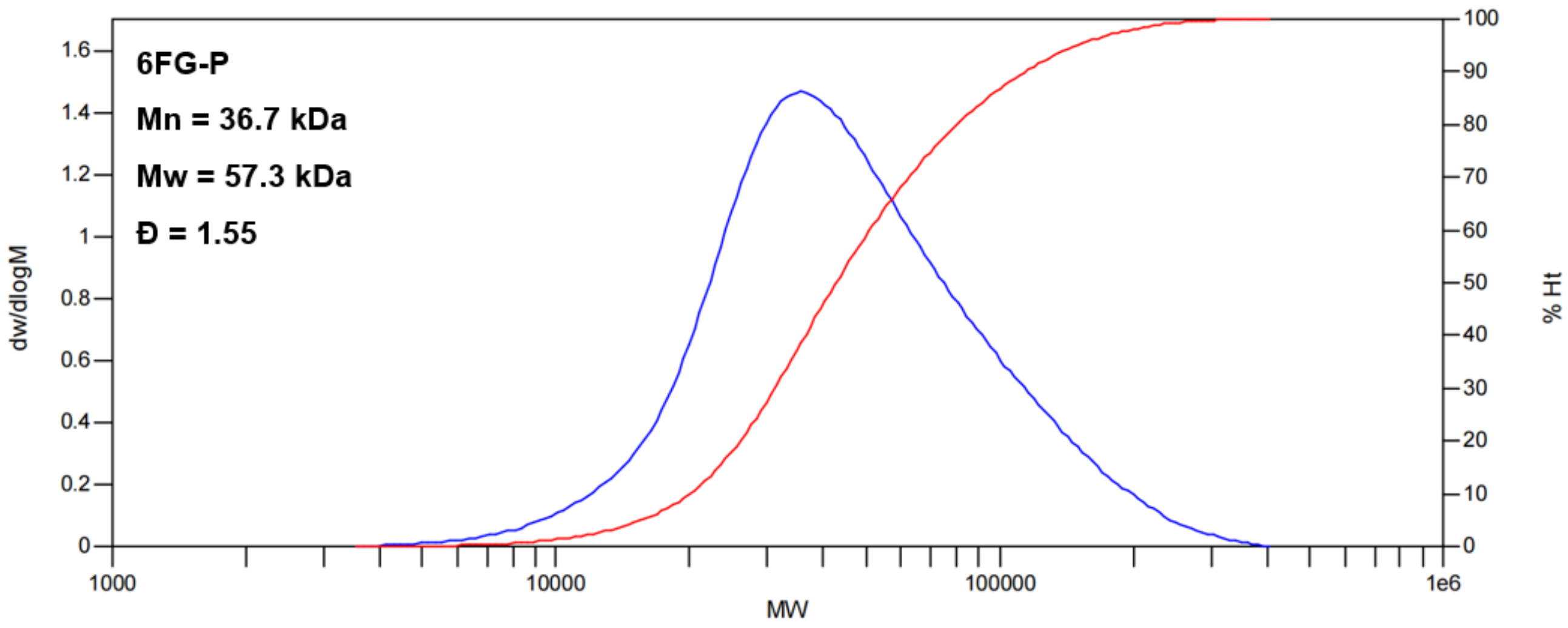

Figure S101. GPC trace of polymer 6FG-P.

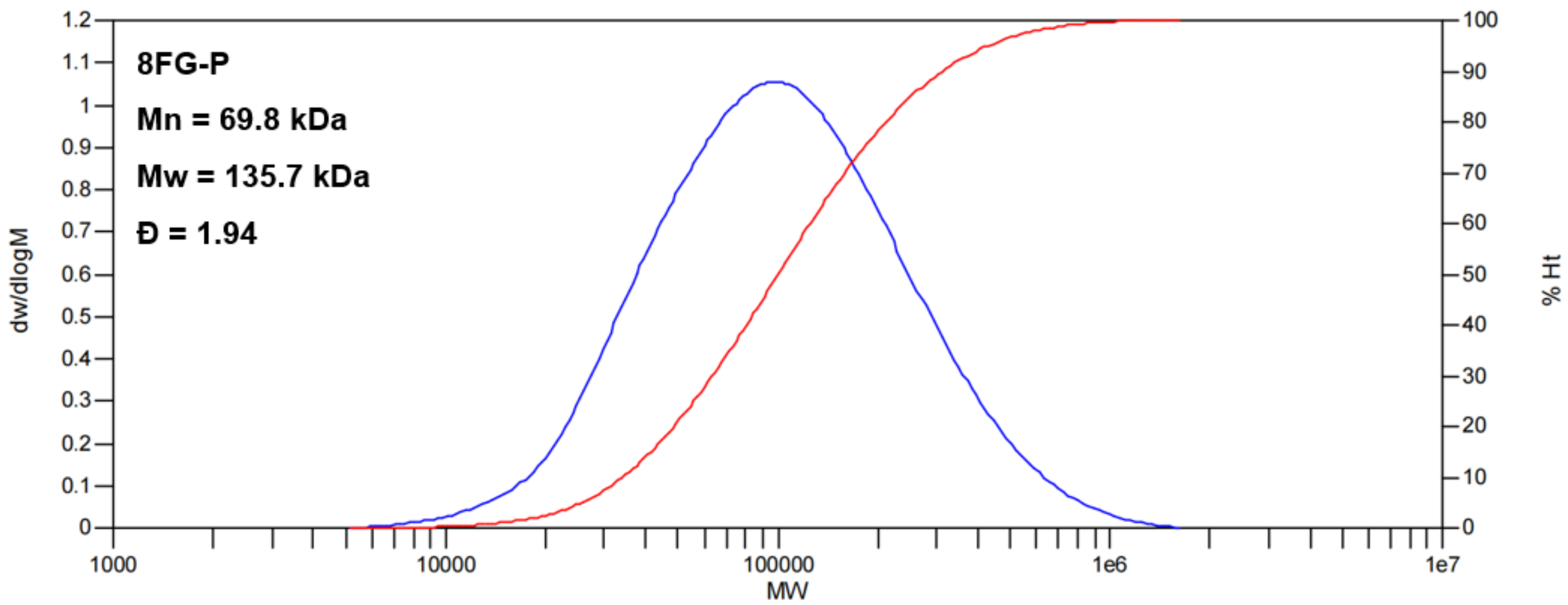

Figure S102. GPC trace of polymer 8FG-P.

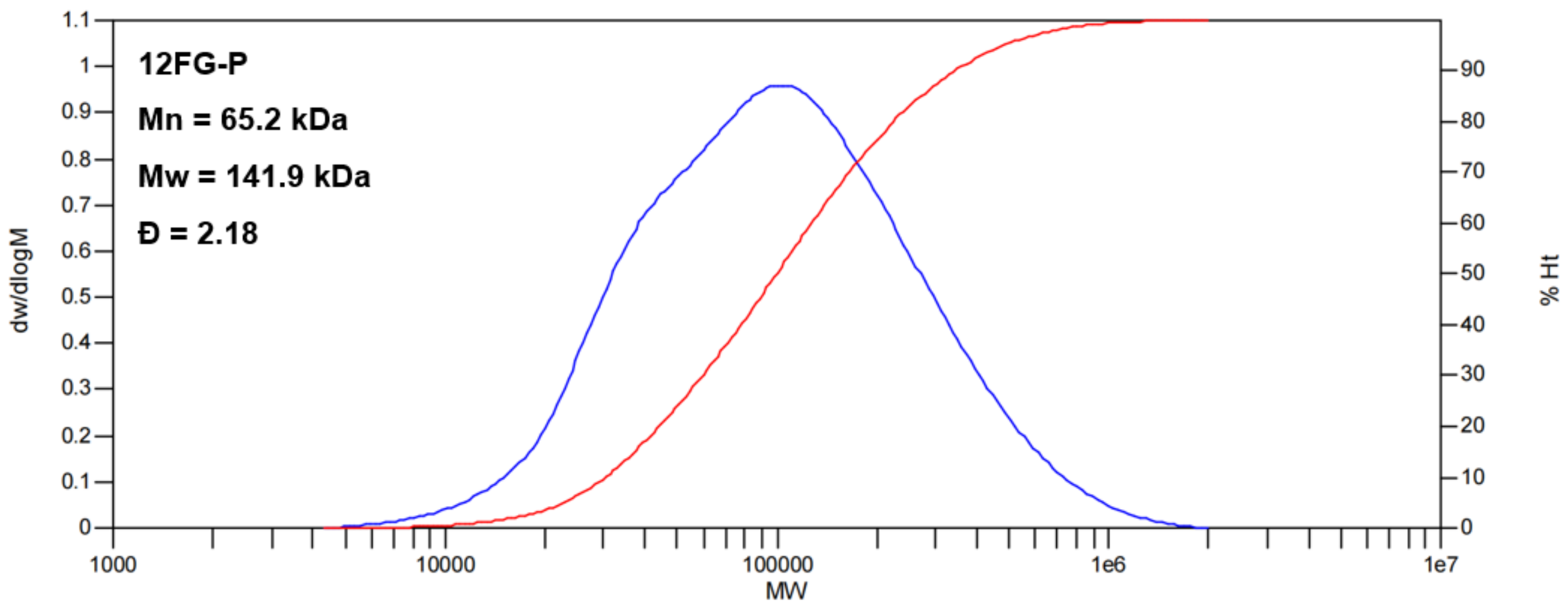

Figure S103. GPC trace of polymer 12FG-P.

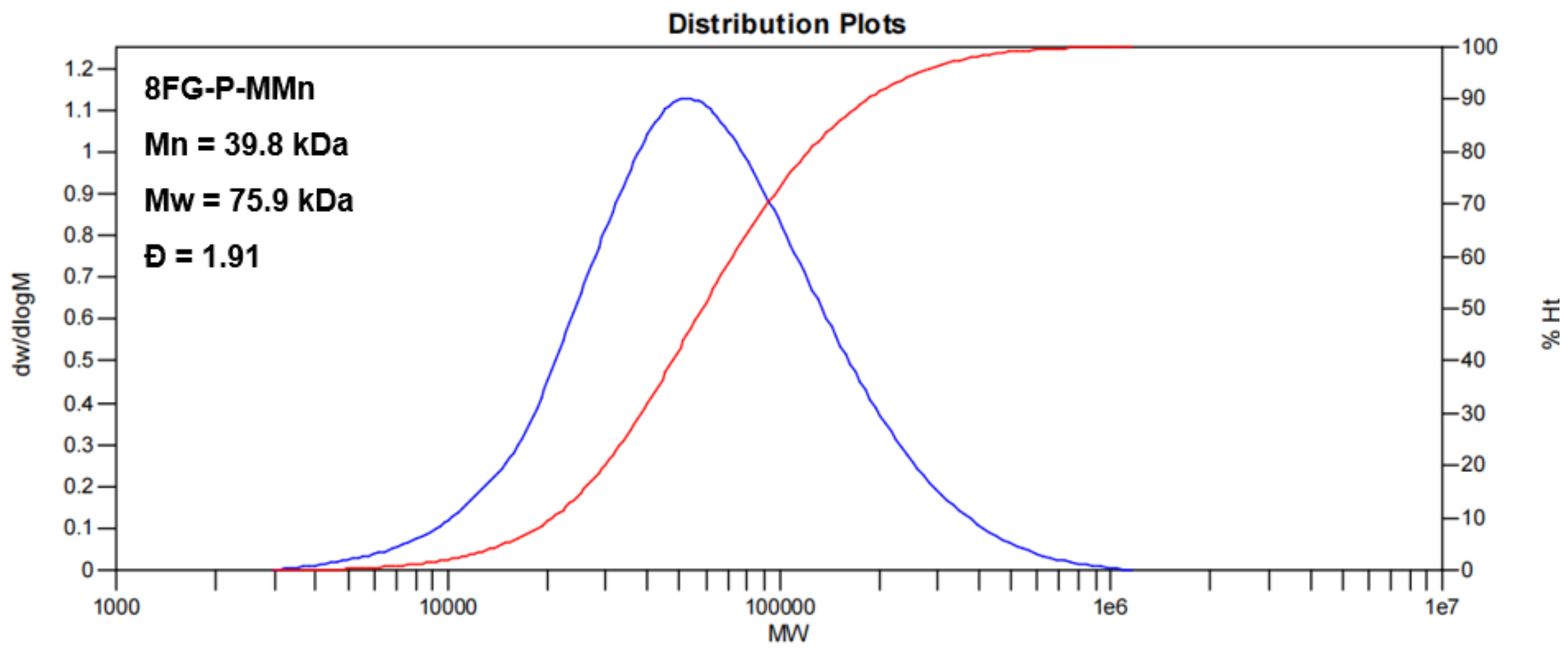

Figure S104. GPC trace of polymer 8FG-P-MMn.

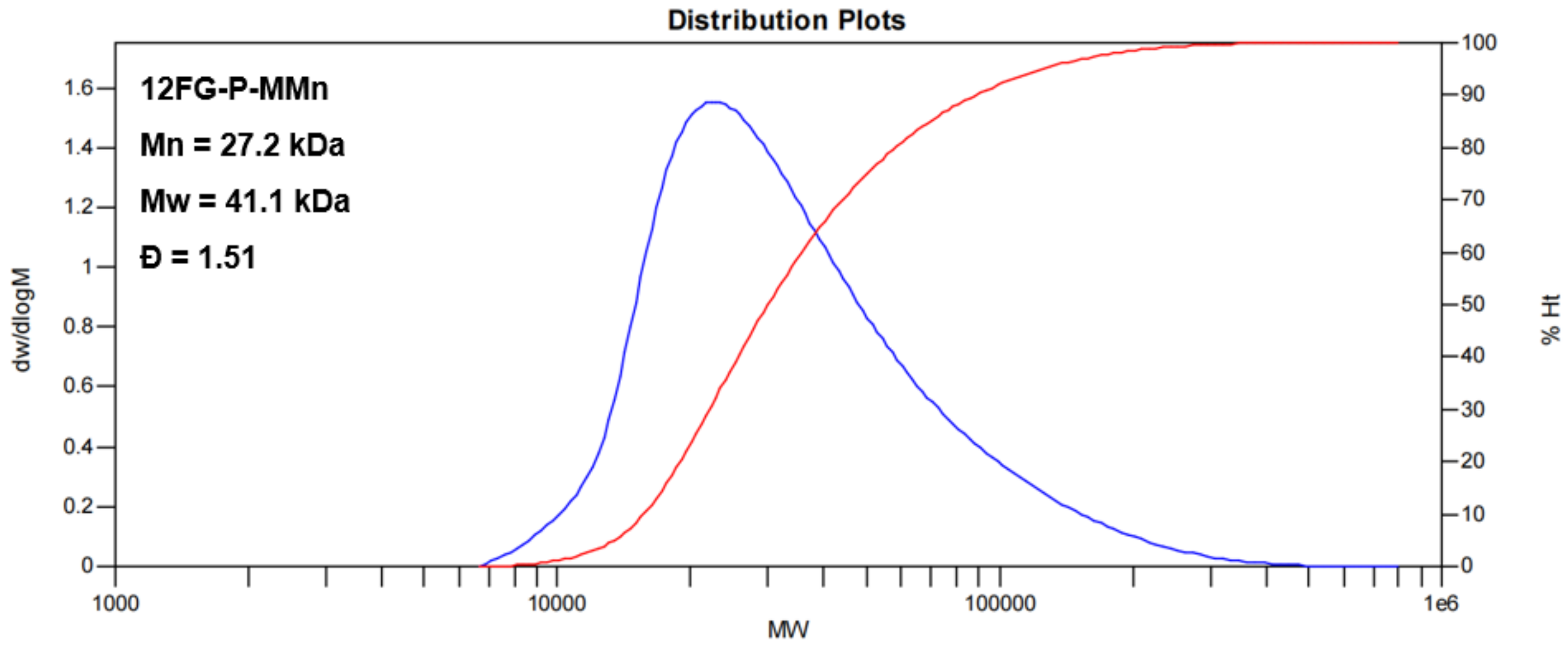

Figure S105. GPC trace of polymer 12FG-P-MMn.

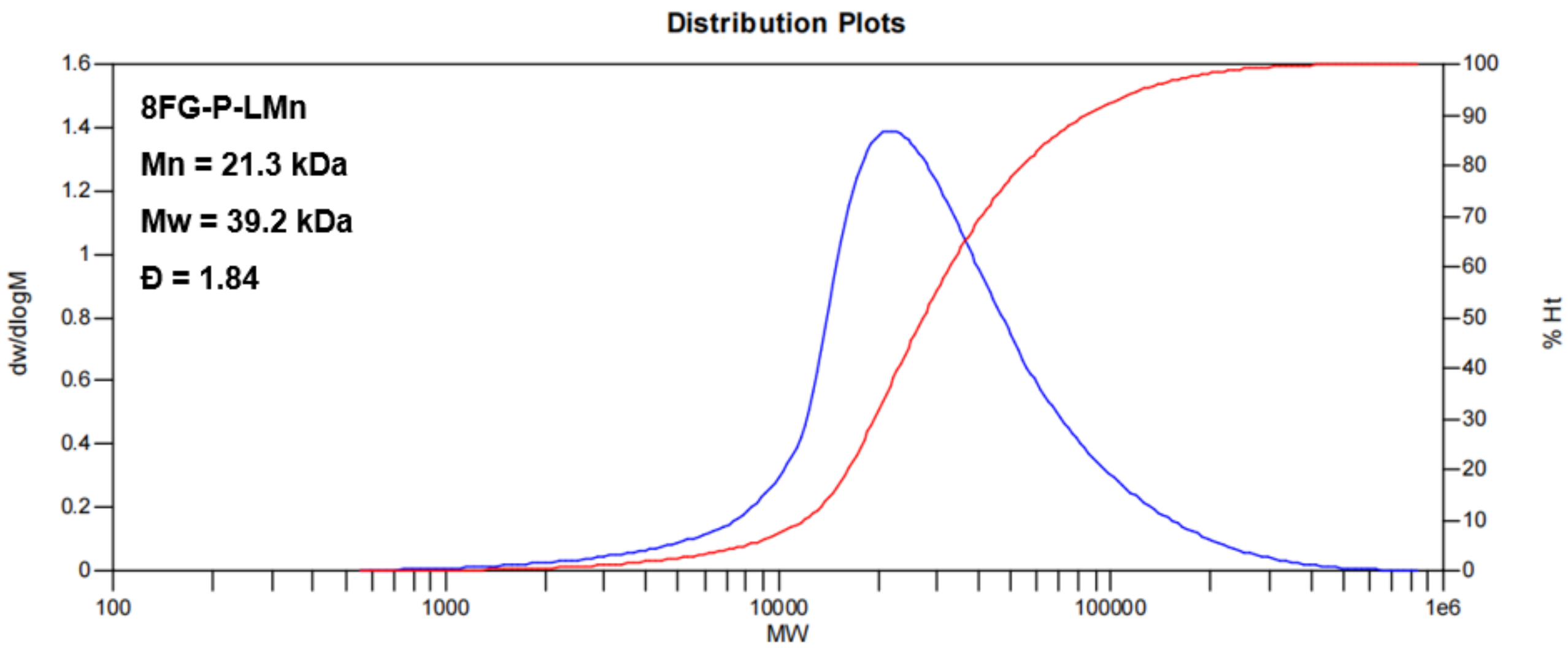

Figure S106. GPC trace of polymer 8FG-P-LMn.

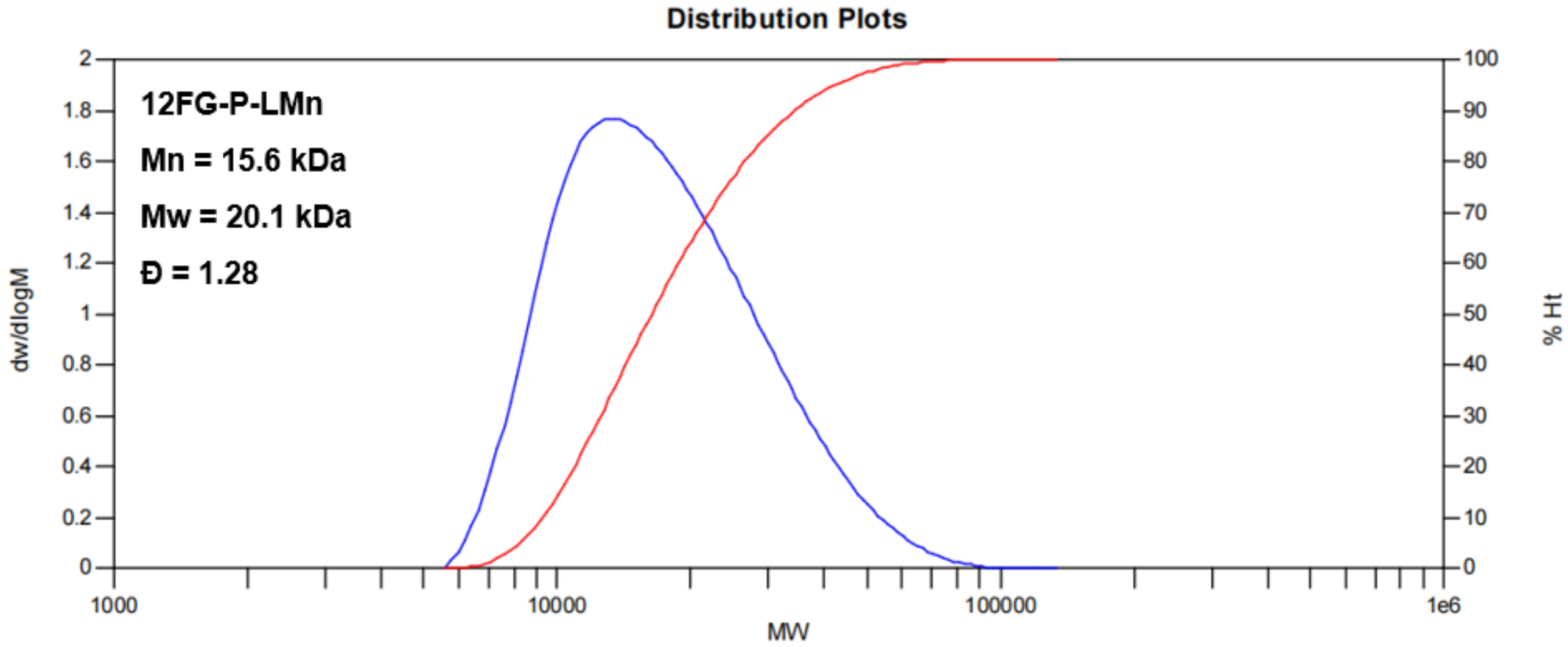

Figure S107. GPC trace of polymer 12FG-P-LMn.

24. References

(1) *Gaussian 16 Rev. C.01*; Wallingford, CT, 2016. (accessed.

(2) Stewart, K.; Tan, E.; Kim, J.; Kim, Y.-H.; Kim, J.-S. Polarons in DPP Polymers – How Glycol Side Chains and Elongated Conjugated Backbone Influence the Formation and Transport. *Adv. Electron. Mater.* **2026**, *12* (7), e00731.

(3) Savva, A.; Cendra, C.; Giugni, A.; Torre, B.; Surgailis, J.; Ohayon, D.; Giovannitti, A.; McCulloch, I.; Di Fabrizio, E.; Salleo, A.; Rivnay, J.; Inal, S. Influence of Water on the Performance of Organic Electrochemical Transistors. *Chem. Mater.* **2019**, *31* (3), 927-937.

(4) Meli, D.; Thomas, Q.; Rolland, N.; Freychet, G.; Kousseff, C. J.; Cavassin, P.; Flagg, L. Q.; Lemaur, V.; Surendran, A.; Hamid, Z.; Griggs, S.; Wu, R.; Huerta, R. A.; Duplessis, I. D.; Paulsen, B. D.; Marks, T. J.; Lauhon, L. J.; McCulloch, I.; Richter, L. J.; Beljonne, D.; Rivnay, J. Charge state-dependent ion condensation near conjugated polymer backbones. *Mater. Horiz.* **2026**, *13* (4), 1877-1886.

(5) Huang, Z.; Li, P.; Lei, Y.; Deng, X.-Y.; Chen, Y.-N.; Tian, S.; Pan, X.; Lei, X.; Song, C.; Zheng, Y.; Wang, J.-Y.; Zhang, Z.; Lei, T. Azonia-Naphthalene: A Cationic Hydrophilic Building Block for Stable N-Type Organic Mixed Ionic-Electronic Conductors. *Angew. Chem. Int. Ed.* **2024**, *63* (6), e202313260.

(6) Hallani, R. K.; Paulsen, B. D.; Petty, A. J., II; Sheelamanthula, R.; Moser, M.; Thorley, K. J.; Sohn, W.; Rashid, R. B.; Savva, A.; Moro, S.; Parker, J. P.; Drury, O.; Alsufyani, M.; Neophytou, M.; Kosco, J.; Inal, S.; Costantini, G.; Rivnay, J.; McCulloch, I. Regiochemistry-Driven Organic Electrochemical Transistor Performance Enhancement in Ethylene Glycol-Functionalized Polythiophenes. *J. Am. Chem. Soc.* **2021**, *143* (29), 11007-11018.

(7) Surgailis, J.; Flagg, L. Q.; Richter, L. J.; Druet, V.; Griggs, S.; Wu, X.; Moro, S.; Ohayon, D.; Kousseff, C. J.; Marks, A.; Maria, I. P.; Chen, H.; Moser, M.; Costantini, G.; McCulloch, I.; Inal, S. The Role of Side Chains and Hydration on Mixed Charge Transport in n-Type Polymer Films. *Adv. Mater.* **2024**, *36* (51), 2313121.

(8) Savva, A.; Hallani, R.; Cendra, C.; Surgailis, J.; Hidalgo, T. C.; Wustoni, S.; Sheelamanthula, R.; Chen, X.; Kirkus, M.; Giovannitti, A.; Salleo, A.; McCulloch, I.; Inal, S. Balancing Ionic and Electronic Conduction for High-Performance Organic Electrochemical Transistors. *Adv. Funct. Mater.* **2020**, *30* (11), 1907657.

(9) Jeong, D.; Jo, I.-Y.; Lee, S.; Kim, J. H.; Kim, Y.; Kim, D.; Reynolds, J. R.; Yoon, M.-H.; Kim, B. J. High-Performance n-Type Organic Electrochemical Transistors Enabled by Aqueous Solution Processing of Amphiphilicity-Driven Polymer Assembly. *Adv. Funct. Mater.* **2022**, *32* (16), 2111950.

(10) Moser, M.; Hidalgo, T. C.; Surgailis, J.; Gladisch, J.; Ghosh, S.; Sheelamanthula, R.; Thiburce, Q.; Giovannitti, A.; Salleo, A.; Gasparini, N.; Wadsworth, A.; Zozoulenko, I.; Berggren, M.; Stavrinidou, E.; Inal, S.; McCulloch, I. Side Chain Redistribution as a Strategy to Boost Organic Electrochemical Transistor Performance and Stability. *Adv. Mater.* **2020**, *32* (37), 2002748.

(11) Hidalgo Castillo, T. C.; Ponder Jr., J. F.; Feng, K.; Magni, A.; Marks, A.; Arcangeli, D.; Uribe, J.; Emwas, A.-H.; Sheelraamanthula, R.; Cowen, L. M.; Schroeder, B. C.; McCulloch, I.; Guo, X.; Salleo, A.; Inal, S. Side Chains Override Crystallinity in n-Type Organic Mixed Conductors. *Adv. Mater.* **2026**, *38* (31), e21048.

(12) Liao, H.; Luo, L.; Wang, Y.; Shan, W.; Yang, Y.-Y.; Luke, J.; Marsh, A. V.; Castillo, T. C. H.; Hama, A.; Faber, H.; Rodríguez-Martínez, X.; Martín, J.; Savva, A.; Anthopoulos, T. D.; Inal, S.; Heeney, M. Tunable Stability and Performance of Fused Thienothiophene Based Polymers for Organic Electrochemical Transistors and Artificial Synapse Based on A Side Chain Reorganization Strategy. *Adv. Mater.* *n/a* (n/a), e12687.

(13) Hidalgo Castillo, T. C.; Shan, W.; Ma, G.; Zhao, H.; Wang, Y.; Druet, V.; Saleh, A.; Gu, X.; Inal, S. Thermal Annealing for High Performance and Memory Behavior in n-Type Organic Electrochemical Transistors. *Adv.*

Mater. **2025**, *37* (5), 2411214.
(14) Szumska, A. A.; Maria, I. P.; Flagg, L. Q.; Savva, A.; Surgailis, J.; Paulsen, B. D.; Moia, D.; Chen, X.; Griggs, S.; Mefford, J. T.; Rashid, R. B.; Marks, A.; Inal, S.; Ginger, D. S.; Giovannitti, A.; Nelson, J. Reversible Electrochemical Charging of n-Type Conjugated Polymer Electrodes in Aqueous Electrolytes. *J. Am. Chem. Soc.* **2021**, *143* (36), 14795-14805.
(15) Ohayon, D.; Flagg, L. Q.; Giugni, A.; Wustoni, S.; Li, R.; Hidalgo Castillo, T. C.; Emwas, A.-H.; Sheelamanthula, R.; McCulloch, I.; Richter, L. J.; Inal, S. Salts as Additives: A Route to Improve Performance and Stability of n-Type Organic Electrochemical Transistors. *ACS Mater. Au* **2023**, *3* (3), 242-254.
(16) Surgailis, J.; Savva, A.; Druet, V.; Paulsen, B. D.; Wu, R.; Hamidi-Sakr, A.; Ohayon, D.; Nikiforidis, G.; Chen, X.; McCulloch, I.; Rivnay, J.; Inal, S. Mixed Conduction in an N-Type Organic Semiconductor in the Absence of Hydrophilic Side-Chains. *Adv. Funct. Mater.* **2021**, *31* (21), 2010165.
(17) Ohayon, D.; Hamidi-Sakr, A.; Surgailis, J.; Wustoni, S.; Dereli, B.; Wehbe, N.; Nastase, S.; Chen, X.; McCulloch, I.; Cavallo, L.; Inal, S. Impact of Noncompensating Ions on the Electrochemical Performance of n-Type Polymeric Mixed Conductors. *J. Am. Chem. Soc.* **2025**, *147* (15), 12523-12533.
(18) Bynens, L.; Zhang, K.; Cavassin, P.; Goossens, A.; Vanderspikken, J.; Castillo, T. C. H.; Tsokkou, D.; Marks, A.; Magni, A.; Weaver, K.; Lutsen, L.; Inal, S.; Vandewal, K.; Banerji, N.; Maes, W. Organic Electrochemical Transistor Channel Materials: Copolymerization Versus Physical Mixing of Glycolated and Alkoxylated Polymers. *Adv. Funct. Mater.* **2026**, *36* (20), 2423913.
(19) Feng, W.; Mo, S.; Wang, T.; Li, H.; Chen, J. Perfluoroalkyl Hybrid Ethylene Glycol Side Chains: A Solution to Water Related Challenges in n-Type Organic Mixed Ionic-Electronic Conductors. *Angew. Chem. Int. Ed.* **2025**.
(20) Feng, K.; Shan, W.; Wang, J.; Lee, J. W.; Yang, W.; Wu, W.; Wang, Y.; Kim, B. J.; Guo, X.; Guo, H. Cyano-Functionalized n-Type Polymer with High Electron Mobility for High-Performance Organic Electrochemical Transistors. *Adv. Mater.* **2022**, *34* (24).
(21) Yang, W.; Feng, K.; Ma, S.; Liu, B.; Wang, Y.; Ding, R.; Jeong, S. Y.; Woo, H. Y.; Chan, P. K. L.; Guo, X. High-Performance n-Type Polymeric Mixed Ionic-Electronic Conductors: The Impacts of Halogen Functionalization. *Adv. Mater.* **2024**, *36* (4), e2305416.
(22) Feng, K.; Shan, W.; Ma, S.; Wu, Z.; Chen, J.; Guo, H.; Liu, B.; Wang, J.; Li, B.; Woo, H. Y.; Fabiano, S.; Huang, W.; Guo, X. Fused Bithiophene Imide Dimer-Based n-Type Polymers for High-Performance Organic Electrochemical Transistors. *Angew. Chem. Int. Ed.* **2021**, *60* (45), 24198-24205.
(23) Zhang, X.; Zhu, R.; Yang, W.; Wang, K.; Ding, R.; Jeong, S. Y.; Woo, H. Y.; Feng, K.; Guo, X. Backbone Engineering of Bithiophene Imide Dimer-Based Polymeric Mixed Ionic-Electronic Conductors for High-Performance n-Type Organic Electrochemical Transistors. *Small* **2025**, *21* (8), e2408716.
(24) Ding, R.; Zhang, X.; Yan, R.; Peng, M.; Su, S.; Jeong, S. Y.; Woo, H. Y.; Guo, X.; Feng, K.; Guo, Z.-H. Ultra-Low Threshold Voltage in N-Type Organic Electrochemical Transistors Enabled by Organic Mixed Ionic-Electronic Conductors with Dual Electron-Withdrawing Substitutions. *Adv. Funct. Mater.* **2025**, *35* (2), 2412181.
(25) Wu, W.; Feng, K.; Wang, Y.; Wang, J.; Huang, E.; Li, Y.; Jeong, S. Y.; Woo, H. Y.; Yang, K.; Guo, X. Selenophene Substitution Enabled High-Performance n-Type Polymeric Mixed Ionic-Electronic Conductors for Organic Electrochemical Transistors and Glucose Sensors. *Adv. Mater.* **2024**, *36* (1), e2310503.
(26) Yang, W.; Ma, S.; Gamez-Valenzuela, S.; Jeong, S. Y.; Lee, J. W.; Cai, H.; Zhu, R.; Liu, B.; Woo, H. Y.; Kim, B. J.; Wang, S. J.; Chan, P. K. L.; Guo, X.; Feng, K. Backbone Tailoring Enables High-Performance and Stable n-Type Organic Mixed Ionic-Electronic Conductors for Synaptic Simulation and Biosensor. *Adv. Mater.* **2026**, *38* (3), e12070.

(27) Wang, Y.; Koklu, A.; Zhong, Y.; Chang, T.; Guo, K.; Zhao, C.; Castillo, T. C. H.; Bu, Z.; Xiao, C.; Yue, W.; Ma, W.; Inal, S. Acceptor Functionalization via Green Chemistry Enables High-Performance n-Type Organic Electrochemical Transistors for Biosensing, Memory Applications. *Adv. Funct. Mater.* **2024**, *34* (15), 2304103.
(28) Wang, Y.; Shan, W.; Li, H.; Zhong, Y.; Wustoni, S.; Uribe, J.; Chang, T.; Musteata, V. E.; Yue, W.; Ling, H.; El-Atab, N.; Inal, S. An optoelectrochemical synapse based on a single-component n-type mixed conductor. *Nat. Commun.* **2025**, *16* (1), 1615.
(29) Parr, Z. S.; Borges-Gonzalez, J.; Rashid, R. B.; Thorley, K. J.; Meli, D.; Paulsen, B. D.; Strzalka, J.; Rivnay, J.; Nielsen, C. B. From p- to n-Type Mixed Conduction in Isoindigo-Based Polymers through Molecular Design. *Adv. Mater.* **2022**, *34* (14), e2107829.
(30) Wang, Y.; Zhu, G.; Zeglio, E.; Castillo, T. C. H.; Haseena, S.; Ravva, M. K.; Cong, S.; Chen, J.; Lan, L.; Li, Z.; Herland, A.; McCulloch, I.; Inal, S.; Yue, W. n-Type Organic Electrochemical Transistors with High Transconductance and Stability. *Chem. Mater.* **2023**, *35* (2), 405-415.
(31) Chen, X.; Marks, A.; Paulsen, B. D.; Wu, R.; Rashid, R. B.; Chen, H.; Alsufyani, M.; Rivnay, J.; McCulloch, I. n-Type Rigid Semiconducting Polymers Bearing Oligo(Ethylene Glycol) Side Chains for High-Performance Organic Electrochemical Transistors. *Angew Chem Int Ed Engl* **2021**, *60* (17), 9368-9373.
(32) Gunturkun, D.; Meli, D.; Thorley, K. J.; Kuhn, M.; Herzig, E. M.; Rivnay, J.; Nielsen, C. B. Impact of donor modification on azaisoindigo-based polymers for n- and p-type organic electrochemical transistors. *J. Mater. Chem. C* **2025**, *13* (44), 22244-22252.
(33) Tan, J.; Wang, Y.; Zhu, X.; Duan, J.; Liu, R.; Chen, C.; Ran, C.; Li, Z.; Ai, B.; Yue, W. Polymeric mixed ionic–electronic conductors based on quinoid–azaisoindigo for n-type organic electrochemical transistors. *Mater. Chem. Front.* **2025**, *9* (4), 725-734.
(34) Shi, J.; Li, P.; Deng, X.-Y.; Xu, J.; Huang, Z.; Lei, Y.; Wang, Y.; Wang, J.-Y.; Gu, X.; Lei, T. Revealing the Role of Polaron Distribution on the Performance of n-Type Organic Electrochemical Transistors. *Chem. Mater.* **2022**, *34* (2), 864-872.
(35) Erhardt, A.; Hochgesang, A.; McNeill, C. R.; Thelakkat, M. A Competitive n-Type OECT Material via Copolymerization of Electron Deficient Building Blocks. *Adv. Electron. Mater.* **2023**, *9* (7).
(36) Li, P.; Shi, J.; Lei, Y.; Huang, Z.; Lei, T. Switching p-type to high-performance n-type organic electrochemical transistors via doped state engineering. *Nat. Commun.* **2022**, *13* (1), 5970.
(37) Yu, Z.; Jiang, X.; Shi, C.; Shi, Y.; Huang, L.; Han, Y.; Deng, Y.; Geng, Y. Anisole Processible n-Type Conjugated Polymers Synthesized via C horizontal line H/C horizontal line H Oxidative Direct Arylation Polycondensation for Organic Electrochemical Transistors. *Macromol. Rapid Commun.* **2025**, *46* (4), e2400757.
(38) Ma, M.; Zhang, L.; Huang, M.; Kuang, Y.; Li, H.; Yang, H.; Yao, T.; Ye, G.; Shao, S.; Yoon, M. H.; Liu, J. Regiochemistry and Side-Chain Engineering Enable Efficient N-Type Mixed Conducting Polymers. *Angew Chem Int Ed Engl* **2025**, *64* (21), e202424820.
(39) Giovannitti, A.; Nielsen, C. B.; Sbircea, D.-T.; Inal, S.; Donahue, M.; Niazi, M. R.; Hanifi, D. A.; Amassian, A.; Malliaras, G. G.; Rivnay, J.; McCulloch, I. N-type organic electrochemical transistors with stability in water. *Nat. Commun.* **2016**, *7* (1).
(40) Maria, I. P.; Paulsen, B. D.; Savva, A.; Ohayon, D.; Wu, R.; Hallani, R.; Basu, A.; Du, W.; Anthopoulos, T. D.; Inal, S.; Rivnay, J.; McCulloch, I.; Giovannitti, A. The Effect of Alkyl Spacers on the Mixed Ionic-Electronic Conduction Properties of N-Type Polymers. *Adv. Funct. Mater.* **2021**, *31* (14).
(41) Ohayon, D.; Savva, A.; Du, W.; Paulsen, B. D.; Uguz, I.; Ashraf, R. S.; Rivnay, J.; McCulloch, I.; Inal, S. Influence of Side Chains on the n-Type Organic Electrochemical Transistor Performance. *ACS Appl. Mater. Interfaces* **2021**, *13* (3), 4253-4266.

(42) Jeong, D.; Jo, I. Y.; Lee, S.; Kim, J. H.; Kim, Y.; Kim, D.; Reynolds, J. R.; Yoon, M. H.; Kim, B. J. High-Performance n-Type Organic Electrochemical Transistors Enabled by Aqueous Solution Processing of Amphiphilicity-Driven Polymer Assembly. *Adv. Funct. Mater.* **2022**, *32* (16).
(43) Cong, S.; Chen, J.; Wang, L.; Lan, L.; Wang, Y.; Dai, H.; Liao, H.; Zhou, Y.; Yu, Y.; Duan, J.; Li, Z.; McCulloch, I.; Yue, W. Donor Functionalization Tuning the N-Type Performance of Donor–Acceptor Copolymers for Aqueous-Based Electrochemical Devices. *Adv. Funct. Mater.* **2022**, *32* (29).
(44) Zhang, C.; Zheng, Y.; Li, Y.; Xue, Z.; Zhu, X.; Chen, J.; Ma, J.; Zhang, Z.; Zhong, H.; Yue, W.; Lei, T.; Fei, Z. Polythiophenes for High-Performance N-type Organic Electrochemical Transistors. *Adv. Funct. Mater.* **2025**, *35* (23).
(45) Ma, S.; Wang, J.; Wu, W.; Wu, Z.; Zhang, H.; Chen, R.; Liu, B.; Feng, K.; Woo, H. Y.; Guo, X. Sequential Cyanation of Polythiophenes: Tuning Charge Carrier Polarity in Organic Electrochemical Transistors. *Adv. Electron. Mater.* **2023**, *9* (11).
(46) Li, P.; Sun, W.; Li, J.; Chen, J.; Wang, X.; Mei, Z.; Jin, G.; Lei, Y.; Xin, R.; Yang, M.; Xu, J.; Pan, X.; Song, C.; Deng, X.; Lei, X.; Liu, K.; Wang, X.; Zheng, Y.; Zhu, J.; Lv, S.; Zhang, Z.; Dai, X.; Lei, T. N-type semiconducting hydrogel. *Science* **2024**, *384*, 30.
(47) Wang, Y.; Tan, J.; Hou, H.; Maricherla, H.; Ravva, M. K.; Zhu, X.; Liu, R.; He, J.; Lin, Y.; McCulloch, I.; Li, Z.; Yue, W. High Performing Ambipolar Organic Electrochemical Transistors and Solid-State Inverters Enabled by Hydrophilic/Hydrophobic Side Chains Integration. *Adv. Mater.* **2026**, *38* (8), e15186.
(48) Yao, Y.; Shah, M. B.; Lu, W.; Li, X. e.; Vasant, R.; Hamid, Z.; Ai, K.; Tian, J.; Alsufyani, M.; Rawle, J.; Gaşpar, M.; Wan, Q.; Found, R.; Chen, W.; Kotnik, T.; Nguyen, T.-Q.; Savva, A.; Durrant, J.; McCulloch, I. Side-Chain Free Semiconducting Polymer for High-Performance n-Type Organic Electrochemical Transistors. *J. Am. Chem. Soc.* **2026**, *148* (9), 9494-9503.
(49) Chen, J.; Duan, J.; Wang, R.; Tan, J.; Li, Z.; McCulloch, I.; Yue, W. Complementary Hydrogen─Bonded Functionalized Mixed Conducting Terpolymers for High-Performance n-type Organic Electrochemical Transistors and Healable Inverters. *Angew. Chem. Int. Ed.* **2025**, *64* (31), e202505011.
(50) Wang, Y.; Zeglio, E.; Wang, L.; Cong, S.; Zhu, G.; Liao, H.; Duan, J.; Zhou, Y.; Li, Z.; Mawad, D.; Herland, A.; Yue, W.; McCulloch, I. Green Synthesis of Lactone-Based Conjugated Polymers for n-Type Organic Electrochemical Transistors. *Adv. Funct. Mater.* **2022**, *32* (16), 2111439.
(51) Maria, I. P.; Griggs, S.; Rashid, R. B.; Paulsen, B. D.; Surgailis, J.; Thorley, K.; Le, V. N.; Harrison, G. T.; Combe, C.; Hallani, R.; Giovannitti, A.; Paterson, A. F.; Inal, S.; Rivnay, J.; McCulloch, I. Enhancing the Backbone Coplanarity of n-Type Copolymers for Higher Electron Mobility and Stability in Organic Electrochemical Transistors. *Chem. Mater.* **2022**, *34* (19), 8593-8602.
(52) Chen, J.; Cong, S.; Wang, L.; Wang, Y.; Lan, L.; Chen, C.; Zhou, Y.; Li, Z.; McCulloch, I.; Yue, W. Backbone coplanarity manipulation via hydrogen bonding to boost the n-type performance of polymeric mixed conductors operating in aqueous electrolyte. *Mater. Horiz.* **2023**, *10* (2), 607-618.
(53) Ding, R.; Zhang, X.; Luan, Y.; Peng, M.; Chen, W.; Wang, S.; Su, S.; Lu, S.; Jeong, S. Y.; Woo, H. Y.; Guo, X.; Feng, K.; Guo, Z. H. Donor Engineering for High Performance n-Type OECT Materials with Exceptional Operational Stability. *Angew Chem Int Ed Engl* **2025**, *64* (36), e202513182.
(54) Zhang, Y.; Ye, G.; van der Pol, T. P. A.; Dong, J.; van Doremaele, E. R. W.; Krauhausen, I.; Liu, Y.; Gkoupidenis, P.; Portale, G.; Song, J.; Chiechi, R. C.; van de Burgt, Y. High-Performance Organic Electrochemical Transistors and Neuromorphic Devices Comprising Naphthalenediimide-Dialkoxybithiazole Copolymers Bearing Glycol Ether Pendant Groups. *Adv. Funct. Mater.* **2022**, *32* (27).
